# Accurate analysis of genuine CRISPR editing events with ampliCan

**DOI:** 10.1101/gr.244293.118

**Published:** 2019-05

**Authors:** Kornel Labun, Xiaoge Guo, Alejandro Chavez, George Church, James A. Gagnon, Eivind Valen

**Affiliations:** 1 Department of Informatics/Computational Biology Unit, University of Bergen, Bergen 5008, Norway;; 2 Wyss Institute for Biologically Inspired Engineering, Harvard University, Cambridge, Massachusetts 02115, USA;; 3 Department of Genetics, Harvard Medical School, Boston, Massachusetts 02115, USA;; 4 Department of Pathology and Cell Biology, Columbia University, New York, New York 10032, USA;; 5 Department of Biology, University of Utah, Salt Lake City, Utah 84112, USA;; 6 Sars International Centre for Marine Molecular Biology, University of Bergen, Bergen 5008, Norway

## Abstract

We present ampliCan, an analysis tool for genome editing that unites highly precise quantification and visualization of genuine genome editing events. ampliCan features nuclease-optimized alignments, filtering of experimental artifacts, event-specific normalization, and off-target read detection and quantifies insertions, deletions, HDR repair, as well as targeted base editing. It is scalable to thousands of amplicon sequencing–based experiments from any genome editing experiment, including CRISPR. It enables automated integration of controls and accounts for biases at every step of the analysis. We benchmarked ampliCan on both real and simulated data sets against other leading tools, demonstrating that it outperformed all in the face of common confounding factors.

With the introduction of CRISPR (Jinek et al. 2012; Cong et al. 2013), researchers obtained an inexpensive and effective tool for targeted mutagenesis. Despite some limitations, CRISPR has been widely adopted in research settings and has made inroads into medical applications (Courtney et al. 2016). Successful genome editing relies on the ability to confidently identify induced mutations after repair through nonhomologous end-joining (NHEJ) or homology directed repair (HDR). Insertions or deletions (indels) are often identified by sequencing the targeted loci and comparing the sequenced reads to a reference sequence. Deep sequencing has the advantage of both capturing the nature of the indel, readily identifying frameshift mutations or disrupted regulatory elements, and characterizing the heterogeneity of the introduced mutations in a population. This is of particular importance when the aim is allele-specific editing or the experiment can result in mosaicism.

The reliability of a sequencing-based approach is dependent on the processing and interpretation of the sequenced reads and is contingent on factors such as the inclusion of controls, the alignment algorithm, and the filtering of experimental artifacts. To date, no tool considers and controls for the whole range of biases that can influence this interpretation and, therefore, distort the estimate of the mutation efficiency and lead to erroneous conclusions. Here we introduce a fully automated tool, ampliCan, designed to determine the true mutation frequencies of CRISPR experiments from high-throughput DNA amplicon sequencing. It scales to genome-wide experiments and can be used alone or integrated with the CHOPCHOP (Montague et al. 2014; Labun et al. 2016) guide RNA (gRNA) design tool.

## Results

### ampliCan accurately determines the true mutation efficiency

**Figure 1.**
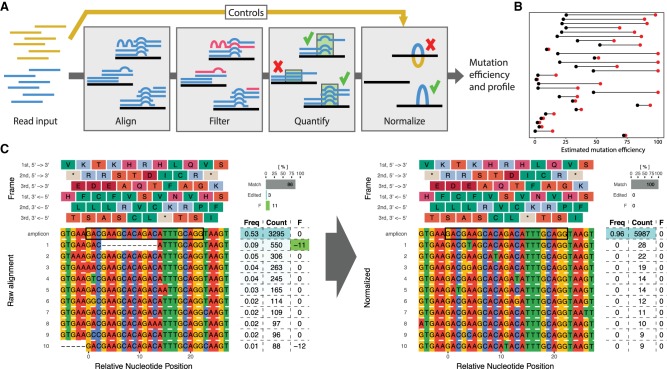
Overview of ampliCan pipeline and normalization. (*A*) Estimation of mutation efficiency consists of multiple steps. At each of these steps, biases can be introduced. Controls are processed identically to the main experiment and used for normalization. (*B*) Overview of the change in estimated mutation efficiency on real CRISPR experiments when using controls that account for natural genetic variance in 29 experiments (mean change of 30%). Red dots show initial estimates based on unnormalized data, whereas black dots show the values after normalization. (*C*) Alignment plot showing the top 10 most abundant reads in a real experiment. The table shows relative efficiency (Freq) of read, absolute number of reads (Count), and the summed size of the indel(s) (F), colored green when inducing a frameshift. The bars (*top right*) show the fraction of reads that contain no indels (Match), those having an indel without inducing frameshift (Edited), and frameshift-inducing indels (F). The *left* panel shows the estimated mutation efficiency from raw reads, which is 14% (11% with frameshift, 3% without). The *right* panel shows the same genomic loci after normalization with controls, resulting in a mutation efficiency of 0%. The deletion of 11 bp in 9% of the reads could not be found in the GRCz10.88 Ensembl Variation database and would, in the absence of controls, give the impression of a real editing event.

Estimation of the true mutation efficiency depends on multiple steps all subject to different biases (Lindsay et al. 2016). Following sequencing, reads have to be aligned to the correct reference and filtered for artifacts, and then the mutation efficiency has to be quantified and normalized (Fig. 1A). In most existing tools, many of the choices made during these steps are typically hidden from the user, leading to potential misinterpretation of the data. These hidden steps can lead to widely different estimates of mutation efficiency (in up to 67% of all experiments) when run on data from real experiments (Supplemental Note S1; Supplemental Fig. S1). Furthermore, steps are frequently relegated to other tools that have not been optimized for CRISPR experiments. ampliCan instead implements a complete pipeline from alignment to interpretation and can therefore control for biases at every step.

Despite being arguably the most important step in any experiment, the use of controls is frequently overlooked in CRISPR assays. Discrepancies between a reference genome and the genetic variation in an organism of interest often lead to false positives and the false impression that mutations have been introduced (Gagnon et al. 2014). Although the use of controls is (in principle) possible with any tool, it commonly requires running the treated and control samples separately followed by a manual inspection and comparison. In ampliCan, controls are an integrated part of the pipeline, and mutation frequencies are normalized and estimated automatically. ampliCan accomplishes this by normalizing at the level of editing events (insertion, deletion, or mismatch) rather than at the level of whole reads. This means that any putative editing event detected in the reads from the target sample that also occurs in the reads from the control sample, above the level of noise, is ignored when calculating mutation frequencies. Importantly, this normalization process does not remove any reads from the calculation; it only refrains from counting the specific editing events that are also present in the controls (Supplemental Figs. S2–S4; Supplemental Table S1). Therefore, it also does not filter any genuine editing events that may co-occur on the same read as a normalized event (see Supplemental Note S2). This process is blind to the source of the event, which may include genetic variance as well as experimental and sequencing artifacts. To assess the impact of controls, we generated 112 CRISPR data sets and pooled them with data we previously generated (Gagnon et al. 2014) for a total of 263 experiments (Methods; Supplemental Note S1; Supplemental Table S2). These consisted of pools of CRISPR-injected zebrafish using wild-type fish as a control. This experimental setup presents a challenging task to pipelines because the genetic background may not be identical across all fish and because the injected fish can be highly mosaic in their mutational outcomes. This benchmark revealed that accounting for the genetic background in the wild-type fish reduced the estimated mutation frequencies substantially in several experiments and is a necessary step to ensure accurate results (Fig. 1B,C; Supplemental Fig. S5).

**Figure 2.**
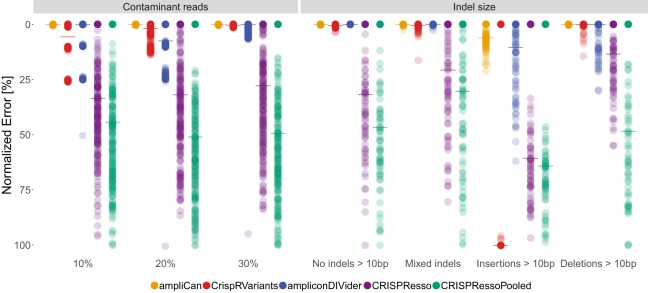
Benchmark of leading tools when estimating mutation efficiency under different data set conditions. Each dot shows the error of the estimate to the correct value for a single experiment normalized to a 0–100 scale. The median performance (mixed indels) is indicated by the horizontal line. The *left* panel shows comparison of tools when data sets contain contaminant reads (see text and Methods). The *x*-axis denotes how dissimilar the contaminant reads are to the correct reads. In cases in which the contaminants are from homologous regions, this may be low (10%); for other contaminants, this is likely to be higher (30%). The *right* panel shows performance of tools as a function of the length of indel events. The sets in the first column contain no indels >10 bp; the second column (Mixed indels) contains a mix of shorter and longer events; the sets in the third and fourth columns contain insertions and deletions >10 bp, respectively.

Estimating mutation efficiency starts with the alignment of the sequenced reads (Fig. 1A). A common strategy is to use standard genomic alignment tools. However, these tools do not align using knowledge about the known mechanisms of CRISPR-induced double-stranded breaks and DNA repair. Genome editing typically results in a single deletion and/or insertion of variable length. Hence, correctly aligned reads will often have a low number of events (optimally one deletion and/or one insertion after normalization for controls) overlapping the cut site, whereas misaligned reads will result in a high number of events throughout the read owing to discrepancies to the correct loci. Therefore an alignment strategy that penalizes multiple indel events (see Methods) is more consistent with DNA repair mechanisms and the CRISPR mode of action. ampliCan uses the Needleman–Wunsch algorithm with tuned parameters to ensure optimal alignments of the reads to their loci and models the number of indel and mismatch events to ensure that the reads originated from that loci (see Methods; Supplemental Note S3). In contrast, nonoptimized aligners can create fragmented alignments, resulting in misleading mutation profiles and possible distortion of downstream analyses and frameshift estimation (Supplemental Fig. S6). In assessments, ampliCan outperforms the tools CrispRVariants, CRISPResso, and ampliconDIVider on the synthetic benchmarking previously used to assess these tools (Lindsay et al. 2016), in which experiments were contaminated with simulated off-target reads that resemble the real on-target reads but have a mismatch rate of 30% per base pair (Supplemental Fig. S7). A cause for concern is that the mapping strategy used in the pipelines of several tools (Supplemental Table S3) is not robust to small perturbations of this mismatch rate, and when we simulated contaminant off-target data with varying degrees of mismatches to the on-target loci (see Supplemental Note S4), it led to a significant reduction in performance (Fig. 2, left). In contrast, ampliCan's strategy of modeling editing events to ascertain whether a read originated from the on-target or the off-target loci resulted in consistently high performance across a broad range of mismatch rates (Fig. 2, left; Supplemental Figs. S7, S8).

### ampliCan can detect long indels and estimate HDR efficiency

Targeted insertion of shorter fragments through co-opting of the homology directed repair (HDR) pathway is becoming increasingly popular (Lackner et al. 2015; Kuscu et al. 2017). This, together with long indels occurring in regular CRISPR experiments (Supplemental Figs. S9, S10), presents a challenge for most CRISPR analysis tools. To assess the ability of the leading tools in recognizing long indels, we simulated data using the strategy from Lindsay et al. (2016), but restricted to indels of ≥10 bp. This revealed an inability of current pipelines to process these longer events (Fig. 2, right), typically stemming from alignment strategies that are unable to assign reads with long indels to the correct loci. In previous assessments, simulated data have often been restricted to short indels in which this weakness would not be apparent (Supplemental Note S5). By using a localized alignment strategy, based on primer matching (see Methods), ampliCan knows a priori which loci the reads are supposed to originate from. This alignment strategy therefore outperforms all other tools and robustly handles these longer indels (>10 bp) when they occur unintentionally (Fig. 2, right; Supplemental Fig. S11).

Intentional introduction of specific edits using donor templates is supported in ampliCan through an HDR mode in which it first aligns the donor template to the reference in order to identify editing events that are expected to take place in a successful integration. The presence of these success-events is then quantified in the edited samples, obtaining the frequency of integration. To assess this strategy, we simulated experiments with different levels of donor integration (a result of HDR) in the presence of different levels of cut loci but with donor introduction (a result of nonhomologous end-joining [NHEJ]). This revealed that only ampliCan can consistently recover both the true HDR and NHEJ efficiency (Supplemental Note S6; Supplemental Fig. S12). An identical strategy also makes it possible to quantify the efficiency of base editors (Komor et al. 2016; Gaudelli et al. 2017) by supplying ampliCan with templates in which the target bases have been altered.

### ampliCan summarizes and aggregates results over thousands of experiments

To aid analysis of heterogeneous outcomes, ampliCan quantifies the heterogeneity of reads (Supplemental Fig. S13), the complete mutation efficiency for an experiment, and the proportion of mutations resulting in a frameshift (Fig. 1C, top right). It also aggregates and quantifies mutation events of a specific type if a particular outcome is desired (Supplemental Fig. S14). In addition, ampliCan provides overviews of the impact of all filtering steps (Supplemental Figs. S15, S16). Reports can be generated in several formats (Supplemental Tables S4, S5) and aggregated at multiple levels such as sequencing barcodes, gRNA, gene, loci, or any user-specified grouping (Supplemental Note S7). This enables exploration of questions beyond mutation efficiency such as the rules of gRNA design, whether a particular researcher is better at designing gRNAs than others (Supplemental Fig. S17), whether a given barcode is not working, or determining the stochasticity in the mutation outcome from a given gRNA (Supplemental Fig. S18).

## Discussion

ampliCan offers a complete pipeline for genome engineering controlling for biases at every step of evaluation. When used with CRISPR, it can be integrated with the CHOPCHOP tool for gRNA design to incorporate all computational steps necessary for a CRISPR experiment. It scales from a single experiment to genome-wide screens and can be run with a single command. For more advanced users, it provides a complete and adaptable framework, enabling further exploration of the data. Collectively, these advances will minimize misinterpretation of genome editing experiments and allow effective analysis of the outcome in an automated fashion.

## Methods

### ampliCan pipeline

ampliCan is completely automated and accepts a configuration file describing the experiment(s) and FASTQ files of sequenced reads as input. The configuration file contains information about barcodes, gRNAs, forward and reverse primers, amplicons, and paths to corresponding FASTQ files (Supplemental Table S6). From here, ampliCan generates reports summarizing the key features of the experiments.

In the first step, ampliCan filters low-quality reads that have either ambiguous nucleotides, an average quality, or individual base quality under a default or user-specified threshold (Supplemental Note S8). After quality filtering, ampliCan assigns reads to the particular experiment by searching for matching primers (default up to two mismatches, but ampliCan supports different stringency) (Supplemental Note S9). Unassigned reads are summarized and reported separately for troubleshooting. After read assignment, ampliCan uses the Biostrings (https://bioconductor.org/packages/release/bioc/html/Biostrings.html) implementation of the Needleman–Wunsch algorithm with optimized parameters (gap opening = −25, gap extension = 0, match = 5, mismatch = −4, no end gap penalty) to align all assigned reads to the loci/amplicon sequence. Subsequently, primer dimer reads are removed by detecting deletions larger than the size of the amplicon, subtracting the length of the two primers and a short buffer. Additionally, sequences that contain a high number of indels or mismatch events compared with the remainder of the reads are filtered as these are potential sequencing artifacts or originate from off-target amplification (Supplemental Note S8; Supplemental Fig. S19). Mutation frequencies are calculated from the remaining reads using the frequency of indels that (Supplemental Fig. S14) overlap a region (±5 bp) around the expected cut site. If paired-end sequencing is used, ampliCan follows consensus rules for the paired forward and reverse read, generally picking the read with the best alignment in case of disagreement (for description, see Supplemental Figs. S20, S21). The alternative strategy of merging the paired reads is supported by ampliCan but has been shown to be detrimental to performance (Lindsay et al. 2016). The expected cut site can be specified as a larger region for nickase or TALEN experiments in which the exact site is not known. Any indel or mismatch also observed above a 1% threshold in the control is removed. Frameshifts are identified by summing the impact of deletions and insertions on the amplicon.

A series of automated reports is prepared in form of “.Rmd” files, which can be converted to multiple formats but also immediately transformed into HTML reports with knitr (https://yihui.name/knitr/) for convenience. There are six different default reports prepared by ampliCan with statistics grouped at the corresponding level: identifier, barcode, gRNA, amplicon, summary, and group (user-specified, but typically signifies the researcher conducting the experiment, treatment of sample, or other grouping of interest). In addition to alignments of top reads (Fig. 1C; Supplemental Fig. S5), reports contain plots summarized over all deletions, insertions, and variants (Supplemental Fig. S14). In addition, a number of plots showing the general state of the experiments is shown, including the heterogeneity of reads to investigate mosaicism or sequencing issues (Supplemental Figs. S13, S22, S23) and overviews of how many reads were filtered/assigned at each step (Supplemental Fig. S24). In addition to the default plots, ampliCan produces R objects that contain all alignments and read information; these can be manipulated, extended, and visualized through the R statistical package.

ampliCan provides a versatile tool that can be used out-of-the-box or as a highly flexible framework that can be extended to more complex analysis. The default pipeline consists of a single convenient wrapper, amplicanPipeline, which generates all default reports. More advanced users can gain complete control over all processing steps (Supplemental Fig. S25) and produce novel plots for more specialized use cases. Compatibility with the most popular plotting packages ggplot2 (https://ggplot2.tidyverse.org) and ggbio (Yin et al. 2012), as well as the most popular data processing packages dplyr (https://dplyr.tidyverse.org) and data.table, provides a full-fledged and elastic framework. Output files are encoded as GenomicRanges (Lawrence et al. 2013) tables of aligned read events for easy parsing (Supplemental Table S5) and human-readable alignment results (Supplemental Table S4) and FASTA. We would like to encourage users to communicate their needs and give us feedback for future development.

### Running parameters

Supplemental Code S1 and https://github.com/valenlab/amplican_manuscript both contain all code related to reproducibility of benchmark and analyses. For benchmarking, all the tools were used with their default options; specific versions of the tools and software can be found in the description file.

### Software availability

ampliCan is developed as an R package (R Core Team 2018) under GNU General Public License version 3 and is available through Bioconductor under http://bioconductor.org/packages/amplican or https://github.com/valenlab/amplican. Supplemental Code S2 contains ampliCan source for installation, version 1.5.6.

## Data access

All real data sets from this study come from the zebrafish TLAB strain and have been submitted to the NCBI BioProject database (BioProject; https://www.ncbi.nlm.nih.gov/bioproject/) under accession number PRJNA245510 (run 1 and run 5). Other data sets used in this study, published previously, are described in the Supplemental Material. Descriptions, treatments, and other details of those data sets were previously described (Gagnon et al. 2014). Synthetic data sets can be reconstructed with the use of code from https://github.com/valenlab/amplican_manuscript (Supplemental Code S1). Synthetic data sets were created in a similar fashion to the sets previously described (Lindsay et al. 2016) using 20 different loci edited at variable efficiency (0%, 33.3%, 66.7%, and 90%) and with the possibility of adding HDR. Further details can be found in the Supplemental Material.

## Supplementary Material

Supplemental Material
