# Supplementary material for: Accurate analysis of genuine CRISPR editing events with ampliCan
Source: Genome Res. 2019 May;29(5):843–7. doi: 10.1101/gr.244293.118 (PMC6499316; doi:10.1101/gr.244293.118)
Supplement: Supplemental Material [file supp_29_5_843__index.html]

Accurate analysis of genuine CRISPR editing events with ampliCan — Accurate analysis of genuine CRISPR editing events with ampliCan — Supplemental Material 

# Accurate analysis of genuine CRISPR editing events with ampliCan

## Supplemental Material

- Supplemental\_Code\_S1.zip
- Supplemental\_Code\_S2.tar.gz
- Supplemental\_Material.pdf
