# Supplementary material for: Accurate analysis of genuine CRISPR editing events with ampliCan
Source: Genome Res. 2019 May;29(5):843–7. doi: 10.1101/gr.244293.118 (PMC6499316; doi:10.1101/gr.244293.118)
Supplement: Supplemental Material [file supp_gr.244293.118_Supplemental_Code_S1.zip › amplican_manuscript/analysis/make_comparison_normalized.html]

Before and After Normalization


# Before and After Normalization

#### *ampliCan*

#### *12 September 2018*

---

# 1 Normalization function

---

|  |  |
| --- | --- |
| amplicanNormalize {amplican} | R Documentation |

## Remove events that can be found in Controls.

### Description

This function can adjust events for small differences between known annotations (amplicon sequences) and real DNA of the strain that was sequenced. Events from the control are grouped by `add` and their frequencies are calculated in respect to number of total reads in that groups. In next step events from the control are filtered according to `min_freq`, all events below are treated as sequencing errors and rejected. Finally, all events that can be found in treatment group that find their exact match (by non skipped columns) in control group are removed. All events from control group are returned back.

### Usage

```
amplicanNormalize(aln, cfgT, add = c("guideRNA", "Group"),
  skip = c("counts", "score", "seqnames", "read_id", "strand",
  "overlaps", "consensus"), min_freq = 0.01)
```

### Arguments

|  |  |
| --- | --- |
| `aln` | (data.frame) Contains events from alignments. |
| `cfgT` | (data.frame) Config table with information about experiments. |
| `add` | (character vector) Columns from cfgT that should be included in event table for normalization matching. Defaults to c(“guideRNA”, “Group”) , which means that only those events created by the same guideRNA in the same Group will be removed if found in Control. |
| `skip` | (character vector) Specifies which columns of aln to skip. |
| `min_freq` | (numeric) All events from control group below this frequency will be not included in filtering. Use this to filter out background noise and sequencing errors. |

### Value

(data.frame) Same as aln, but events are normalized. Events from Control are not changed. Additionally columns from add are added to the data.frame.

### See Also

Other analysis steps: `amplicanAlign`, `amplicanConsensus`, `amplicanFilter`, `amplicanMap`, `amplicanOverlap`, `amplicanPipeline`, `amplicanReport`, `amplicanSummarize`

### Examples

```
aln <- data.frame(seqnames = 1:5, start = 1, end = 2, width = 2,
                  counts = 101:105)
cfgT <- data.frame(ID = 1:5, guideRNA = rep("ACTG", 5),
                   Reads_Filtered = c(2, 2, 3, 3, 4),
                   Group = c("A", "A", "B", "B", "B"),
                   Control = c(TRUE, FALSE, TRUE, FALSE, FALSE))
# all events are same as in the control group, therefore are filtered out
# events from control groups stay
amplicanNormalize(aln, cfgT)
# events that are different from control group are preserved
aln[2, "start"] <- 3
amplicanNormalize(aln, cfgT)
```

---

[Package *amplican* version 1.3.3 Index]

---

# 2 Before and after normalization

---

## 2.1 Injected\_NC1b\_megamind\_MiSeq\_run1

### 2.1.1 Raw

### 2.1.2 Control

### 2.1.3 Normalized

## 2.2 Injected\_NC4\_Cingulin\_intronic\_MiSeq\_run1

### 2.2.1 Raw

### 2.2.2 Control

### 2.2.3 Normalized

## 2.3 Injected\_NP005\_MiSeq\_run1

### 2.3.1 Raw

### 2.3.2 Control

### 2.3.3 Normalized

## 2.4 Injected\_SP1\_MiSeq\_run1

### 2.4.1 Raw

### 2.4.2 Control

### 2.4.3 Normalized

## 2.5 Injected\_SP18\_MiSeq\_run1

### 2.5.1 Raw

### 2.5.2 Control

### 2.5.3 Normalized

## 2.6 Injected\_SP8\_updated2\_MiSeq\_run1

### 2.6.1 Raw

### 2.6.2 Control

### 2.6.3 Normalized

## 2.7 Injected\_aplnrB\_MiSeq\_run1

### 2.7.1 Raw

### 2.7.2 Control

### 2.7.3 Normalized

## 2.8 Injected\_cartb\_MiSeq\_run1

### 2.8.1 Raw

### 2.8.2 Control

### 2.8.3 Normalized

## 2.9 Injected\_cartc\_MiSeq\_run1

### 2.9.1 Raw

### 2.9.2 Control

### 2.9.3 Normalized

## 2.10 Injected\_eomesb\_MiSeq\_run1

### 2.10.1 Raw

### 2.10.2 Control

### 2.10.3 Normalized

## 2.11 Injected\_fgf13b\_MiSeq\_run1

### 2.11.1 Raw

### 2.11.2 Control

### 2.11.3 Normalized

## 2.12 Injected\_NC7\_cyranoC2\_sg76+77\_1\_MiSeq\_run5\_2013\_09\_25

### 2.12.1 Raw

### 2.12.2 Control

### 2.12.3 Normalized

## 2.13 Injected\_NC24\_mboatSall3\_MiSeq\_run5\_2013\_09\_25

### 2.13.1 Raw

### 2.13.2 Control

### 2.13.3 Normalized

## 2.14 Injected\_Toddler\_u1\_1\_MiSeq\_run5\_2013\_09\_25

### 2.14.1 Raw

### 2.14.2 Control

### 2.14.3 Normalized

## 2.15 Injected\_NCorf4\_Cingulinintronic\_MiSeq\_run5\_2013\_09\_25

### 2.15.1 Raw

### 2.15.2 Control

### 2.15.3 Normalized

## 2.16 elovl6\_e1-1-2\_7071\_inj\_MiSeq\_run6\_2013\_11\_19

### 2.16.1 Raw

### 2.16.2 Control

### 2.16.3 Normalized

## 2.17 insert\_ctgfa\_my96ds\_3\_MiSeq\_run6\_2013\_11\_19

### 2.17.1 Raw

### 2.17.2 Control

### 2.17.3 Normalized

## 2.18 SP1\_inj\_MiSeq\_run7\_2014\_01\_02

### 2.18.1 Raw

### 2.18.2 Control

### 2.18.3 Normalized

## 2.19 spaw-lnc\_e1\_1\_inj\_MiSeq\_run7\_2014\_01\_02

### 2.19.1 Raw

### 2.19.2 Control

### 2.19.3 Normalized

## 2.20 elovl6\_e1\_1-2\_inj\_MiSeq\_run7\_2014\_01\_02

### 2.20.1 Raw

### 2.20.2 Control

### 2.20.3 Normalized

## 2.21 old\_g118\_1\_MiSeq\_run8\_2014\_01\_30

### 2.21.1 Raw

### 2.21.2 Control

### 2.21.3 Normalized

## 2.22 atp1a1.1\_e4\_1\_inj\_MiSeq\_run8\_2014\_01\_30

### 2.22.1 Raw

### 2.22.2 Control

### 2.22.3 Normalized

## 2.23 7879\_2\_MiSeq\_run10\_2014\_05\_16

### 2.23.1 Raw

### 2.23.2 Control

### 2.23.3 Normalized

## 2.24 GFPnoSTOP\_gRNA-\_MiSeq\_run9\_2014\_03\_26

### 2.24.1 Raw

### 2.24.2 Control

### 2.24.3 Normalized

## 2.25 GFPnoSTOP\_MiSeq\_run9\_2014\_03\_26

### 2.25.1 Raw

### 2.25.2 Control

### 2.25.3 Normalized

## 2.26 my96ss\_MiSeq\_run9\_2014\_03\_26

### 2.26.1 Raw

### 2.26.2 Control

### 2.26.3 Normalized

## 2.27 invit96ss\_MiSeq\_run9\_2014\_03\_26

### 2.27.1 Raw

### 2.27.2 Control

### 2.27.3 Normalized

## 2.28 213ss\_MiSeq\_run9\_2014\_03\_26

### 2.28.1 Raw

### 2.28.2 Control

### 2.28.3 Normalized

## 2.29 213ds\_MiSeq\_run9\_2014\_03\_26

### 2.29.1 Raw

### 2.29.2 Control

### 2.29.3 Normalized

---

# 3 Fig 1b

---

---

# 4 Mean

---

```
# % of experiments with changed estimated indel rates after normalization
round(sum(big_norm_exp$Raw != big_norm_exp$Normalized)*100/dim(big_norm_exp)[1], 0)
```

```
## [1] 10
```

```
# mean change of experiment rates
bg <- big_norm_exp[big_norm_exp$Raw != big_norm_exp$Normalized, ]
round(median(abs(bg$Raw - bg$Normalized)), 0)
```

```
## [1] 30
```
