# Supplementary material for: Accurate analysis of genuine CRISPR editing events with ampliCan
Source: Genome Res. 2019 May;29(5):843–7. doi: 10.1101/gr.244293.118 (PMC6499316; doi:10.1101/gr.244293.118)
Supplement: Supplemental Material [file supp_gr.244293.118_Supplemental_Code_S1.zip › amplican_manuscript/figures/normalization/MiSeq_run1/Injected_eomesb_control.pdf]

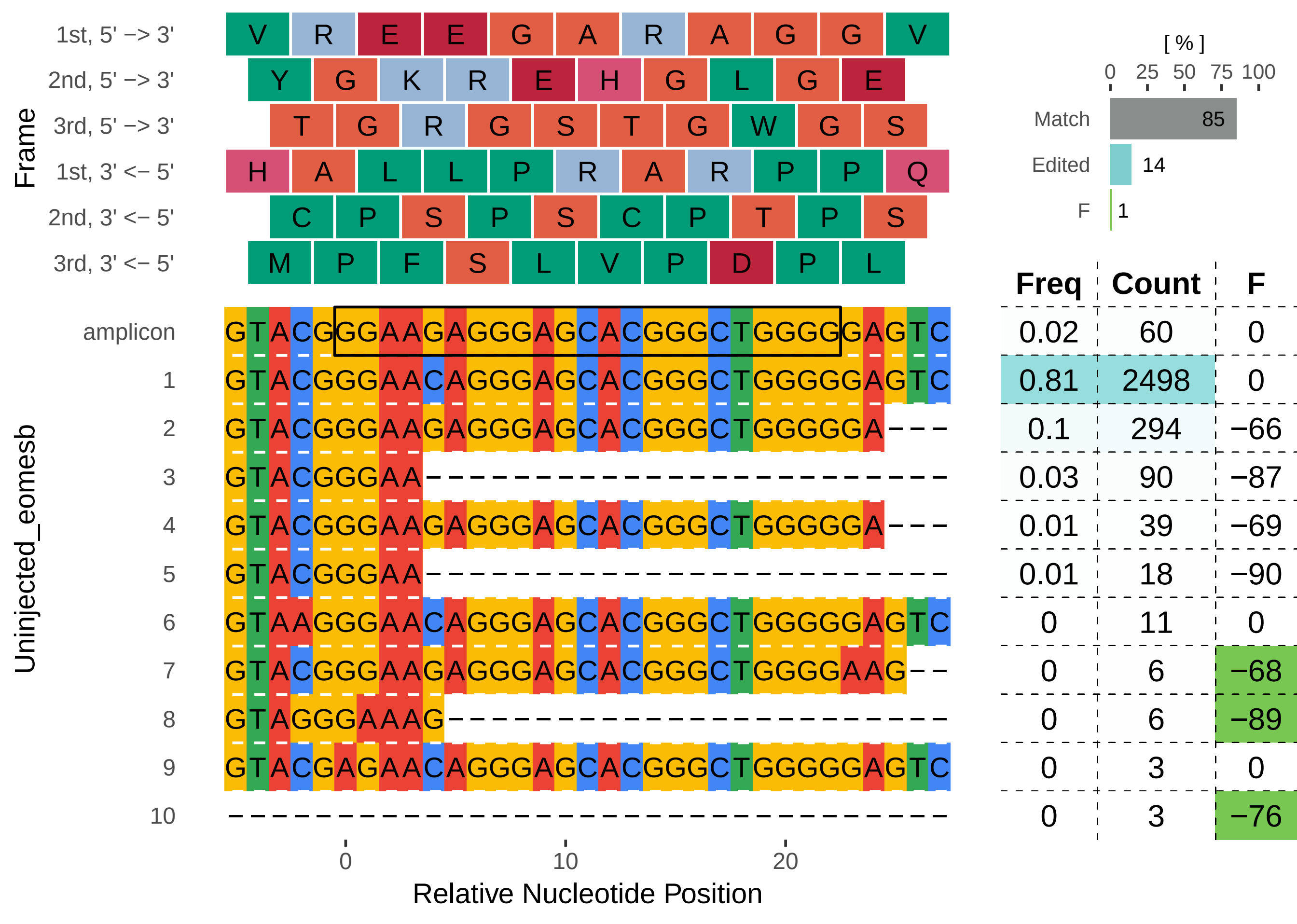
Frame
1st, 5' -> 3'
V R E E G A R A G G V
2nd, 5' -> 3'
Y G K R E H G L G E
3rd, 5' -> 3'
T G R G S T G W G S
1st, 3' <- 5'
H A L L P R A R P P Q
2nd, 3' <- 5'
C P S P S C P T P S
3rd, 3' <- 5'
M P F S L V P D P L
[%]
0 25 50 75 100
Match 85
Edited 14
F 1
Freq Count F
amplicon GTACGGGAAGAGGGAGCACGGGCTGGGGGAGTC 0.02 60 0
1 GTACGGGAACAGGGAGCACGGGCTGGGGGAGTC 0.81 2498 0
2 GTACGGGAAGAGGGAGCACGGGCTGGGGGA--- 0.1 294 −66
3 GTACGGGAA------------------------ 0.03 90 −87
4 GTACGGGAAGAGGGAGCACGGGCTGGGGGA--- 0.01 39 −69
5 GTACGGGAA------------------------ 0.01 18 −90
6 GTAAGGGAACAGGGAGCACGGGCTGGGGGAGTC 0 11 0
7 GTACGGGAAGAGGGAGCACGGGCTGGGGAAG-- 0 6 −68
8 GTAGGGAAAG----------------------- 0 6 −89
9 GTACGAGAACAGGGAGCACGGGCTGGGGGAGTC 0 3 0
10 --------------------------------- 0 3 −76
Uninjected_eomesb
0 10 20
Relative Nucleotide Position
