# Supplementary material for: Accurate analysis of genuine CRISPR editing events with ampliCan
Source: Genome Res. 2019 May;29(5):843–7. doi: 10.1101/gr.244293.118 (PMC6499316; doi:10.1101/gr.244293.118)
Supplement: Supplemental Material [file supp_gr.244293.118_Supplemental_Code_S1.zip › amplican_manuscript/figures/normalization/MiSeq_run1/Injected_fgf13b_normalized.pdf]

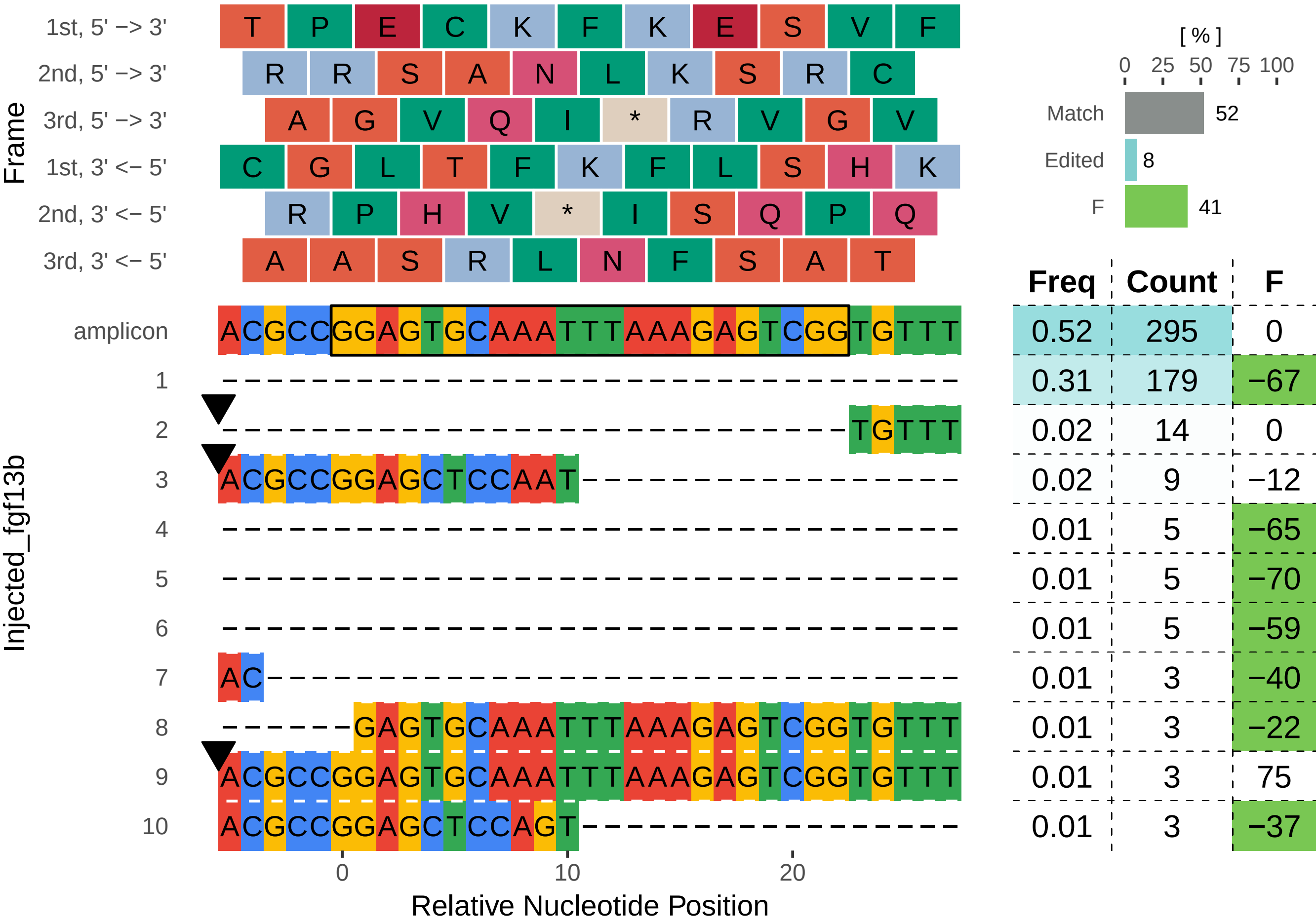

Frame
1st, 5' –> 3'
T P E C K F K E S V F
2nd, 5' –> 3'
R R S A N L K S R C
3rd, 5' –> 3'
A G V Q I * R V G V
1st, 3' <– 5'
C G L T F K F L S H K
2nd, 3' <– 5'
R P H V * I S Q P Q
3rd, 3' <– 5'
A A S R L N F S A T
[%]
0 25 50 75 100
Match 52
Edited 8
F 41
Freq Count F
amplicon ACGCCGGAGTGCAAATTTAAAGAGTCGGTGTTT 0.52 295 0
1 0.31 179 −67
2 TGTTT 0.02 14 0
3 ACGCCGGAGCTCCAAT 0.02 9 −12
4 0.01 5 −65
5 0.01 5 −70
6 0.01 5 −59
7 AC 0.01 3 −40
8 GAGTGCAAATTTAAAGAGTCGGTGTTT 0.01 3 −22
9 ACGCCGGAGTGCAAATTTAAAGAGTCGGTGTTT 0.01 3 75
10 ACGCCGGAGCTCCAGT 0.01 3 −37
Injected_fgf13b
0 10 20
Relative Nucleotide Position
