# Supplementary material for: Accurate analysis of genuine CRISPR editing events with ampliCan
Source: Genome Res. 2019 May;29(5):843–7. doi: 10.1101/gr.244293.118 (PMC6499316; doi:10.1101/gr.244293.118)
Supplement: Supplemental Material [file supp_gr.244293.118_Supplemental_Code_S1.zip › amplican_manuscript/figures/normalization/MiSeq_run1/Injected_NC1b_megamind_control.pdf]

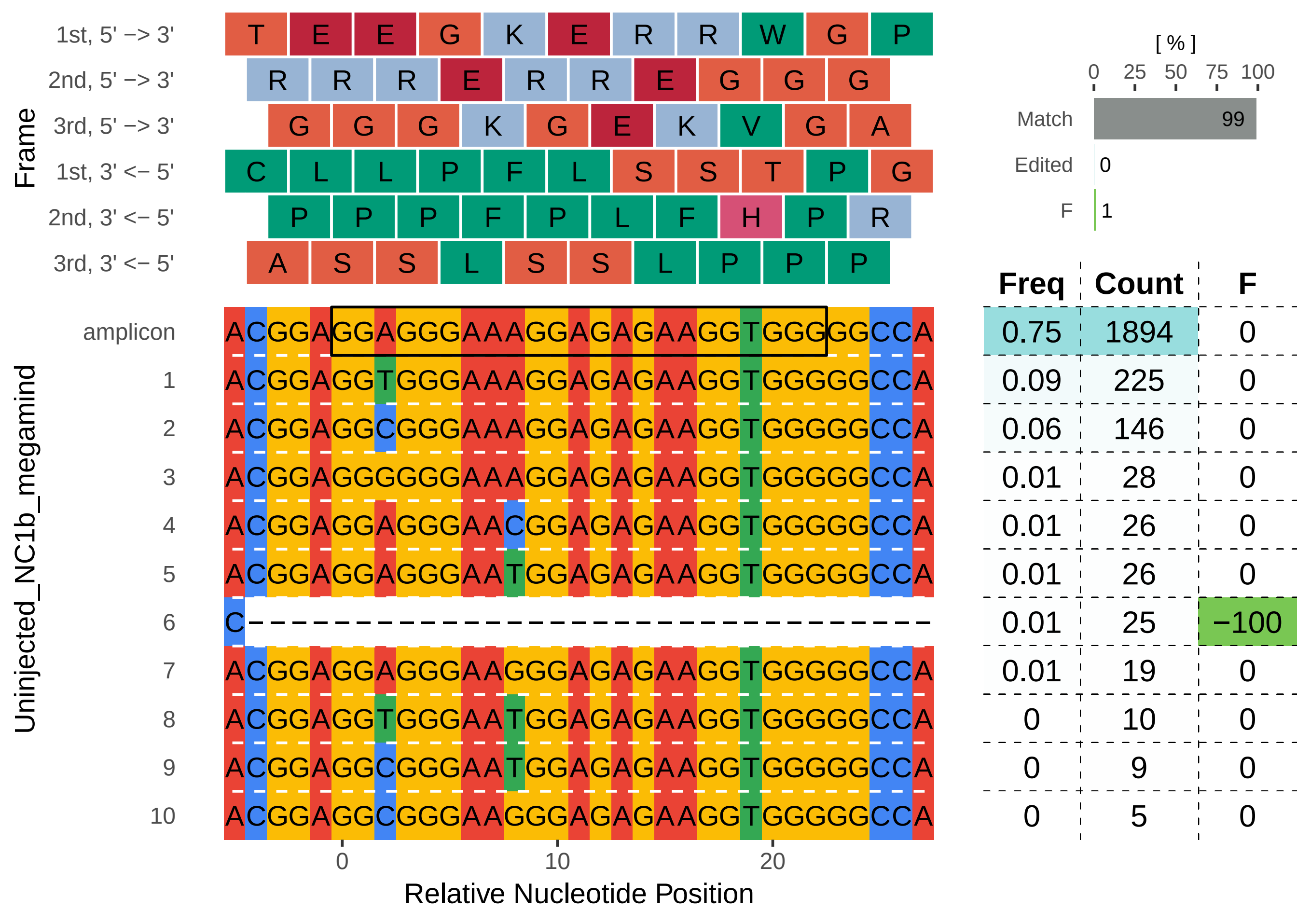

Frame
1st, 5' –> 3'
T E E G K E R R W G P
2nd, 5' –> 3'
R R R E R R E G G G
3rd, 5' –> 3'
G G G K G E K V G A
1st, 3' <– 5'
C L L P F L S S T P G
2nd, 3' <– 5'
P P P F P L F H P R
3rd, 3' <– 5'
A S S L S S L P P P
[%]
0 25 50 75 100
Match 99
Edited 0
F 1
Uninjected_NC1b_megamind
Freq Count F
amplicon ACGGAGGAGGGAAAGGAGAAGGTGGGGGCCA 0.75 1894 0
1 ACGGAGGTGGGAAAGGAGAAGGTGGGGGCCA 0.09 225 0
2 ACGGAGGCGGGAAAGGAGAAGGTGGGGGCCA 0.06 146 0
3 ACGGAGGGGGGAAAGGAGAAGGTGGGGGCCA 0.01 28 0
4 ACGGAGGAGGGAACGGAGAAGGTGGGGGCCA 0.01 26 0
5 ACGGAGGAGGGAATGGAGAAGGTGGGGGCCA 0.01 26 0
6 C------------------------------ 0.01 25 −100
7 ACGGAGGAGGGAAGGGAGAAGGTGGGGGCCA 0.01 19 0
8 ACGGAGGTGGGAATGGAGAAGGTGGGGGCCA 0 10 0
9 ACGGAGGCGGGAATGGAGAAGGTGGGGGCCA 0 9 0
10 ACGGAGGCGGGAAGGGAGAAGGTGGGGGCCA 0 5 0
0 10 20
Relative Nucleotide Position
