# Supplementary material for: Accurate analysis of genuine CRISPR editing events with ampliCan
Source: Genome Res. 2019 May;29(5):843–7. doi: 10.1101/gr.244293.118 (PMC6499316; doi:10.1101/gr.244293.118)
Supplement: Supplemental Material [file supp_gr.244293.118_Supplemental_Code_S1.zip › amplican_manuscript/figures/normalization/MiSeq_run1/Injected_NC4_Cingulin_intronic_raw.pdf]

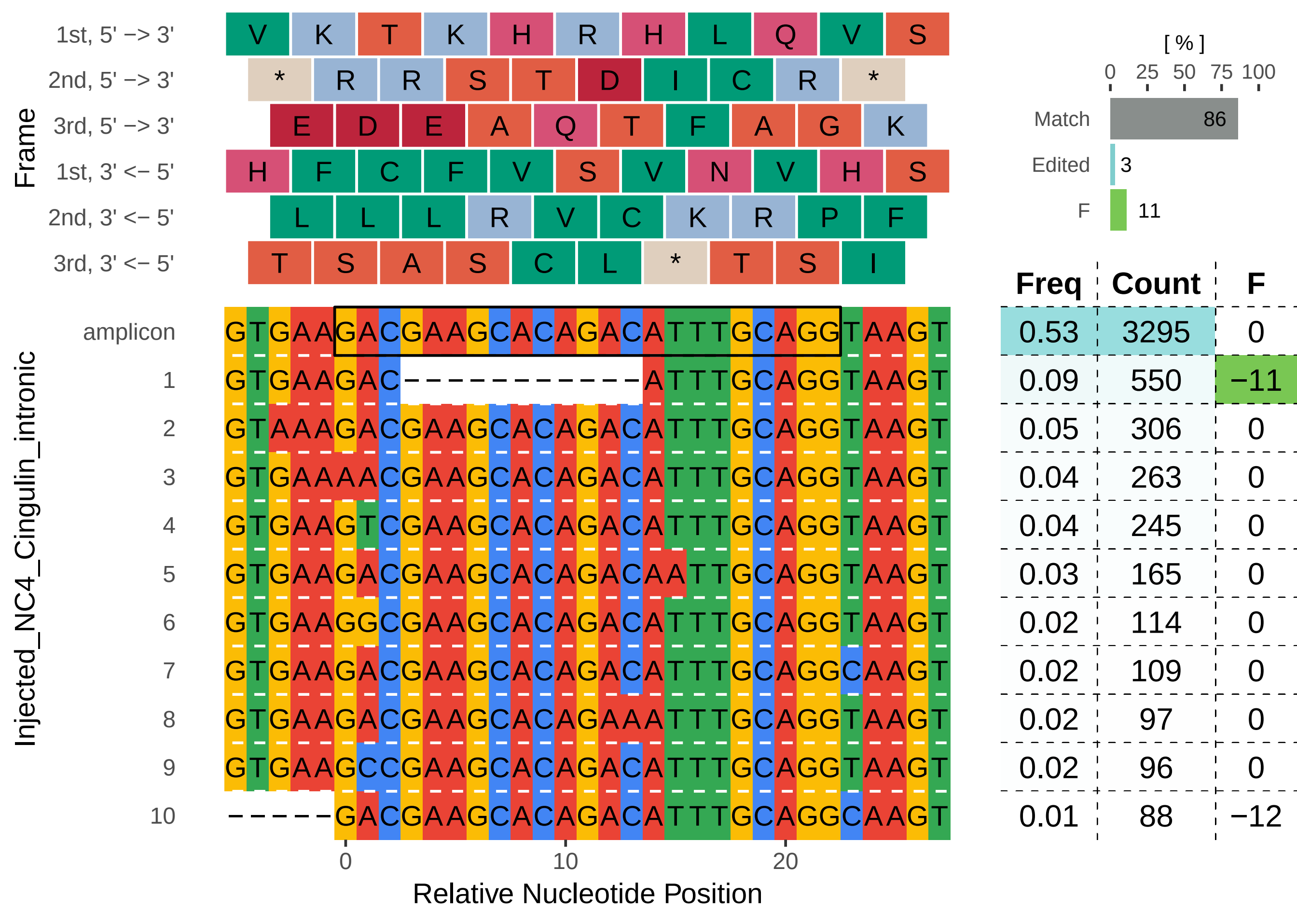
Frame
1st, 5' –> 3'
V K T K H R H L Q V S
2nd, 5' –> 3'
* R R S T D I C R *
3rd, 5' –> 3'
E D E A Q T F A G K
1st, 3' <– 5'
H F C F V S V N V H S
2nd, 3' <– 5'
L L L R V C K R P F
3rd, 3' <– 5'
T S A S C L * T S I
[%]
0 25 50 75 100
Match 86
Edited 3
F 11
Injected_NC4_Cingulin_intronic
Freq Count F
amplicon GTGAAGACGAAGCACAGACATTTGCAGGTAAGT 0.53 3295 0
1 GTGAAGAC------------ATTTGCAGGTAAGT 0.09 550 −11
2 GTAAAGACGAAGCACAGACATTTGCAGGTAAGT 0.05 306 0
3 GTGAAAACGAAGCACAGACATTTGCAGGTAAGT 0.04 263 0
4 GTGAAGTCGAAGCACAGACATTTGCAGGTAAGT 0.04 245 0
5 GTGAAGACGAAGCACAGACAATTGCAGGTAAGT 0.03 165 0
6 GTGAAGGCGAAGCACAGACATTTGCAGGTAAGT 0.02 114 0
7 GTGAAGACGAAGCACAGACATTTGCAGGCAAGT 0.02 109 0
8 GTGAAGACGAAGCACAGAAATTTGCAGGTAAGT 0.02 97 0
9 GTGAAGCCGAAGCACAGACATTTGCAGGTAAGT 0.02 96 0
10 -----GACGAAGCACAGACATTTGCAGGCAAGT 0.01 88 −12
0 10 20
Relative Nucleotide Position
