# Supplementary material for: Accurate analysis of genuine CRISPR editing events with ampliCan
Source: Genome Res. 2019 May;29(5):843–7. doi: 10.1101/gr.244293.118 (PMC6499316; doi:10.1101/gr.244293.118)
Supplement: Supplemental Material [file supp_gr.244293.118_Supplemental_Code_S1.zip › amplican_manuscript/figures/normalization/MiSeq_run1/Injected_NP005_control.pdf]

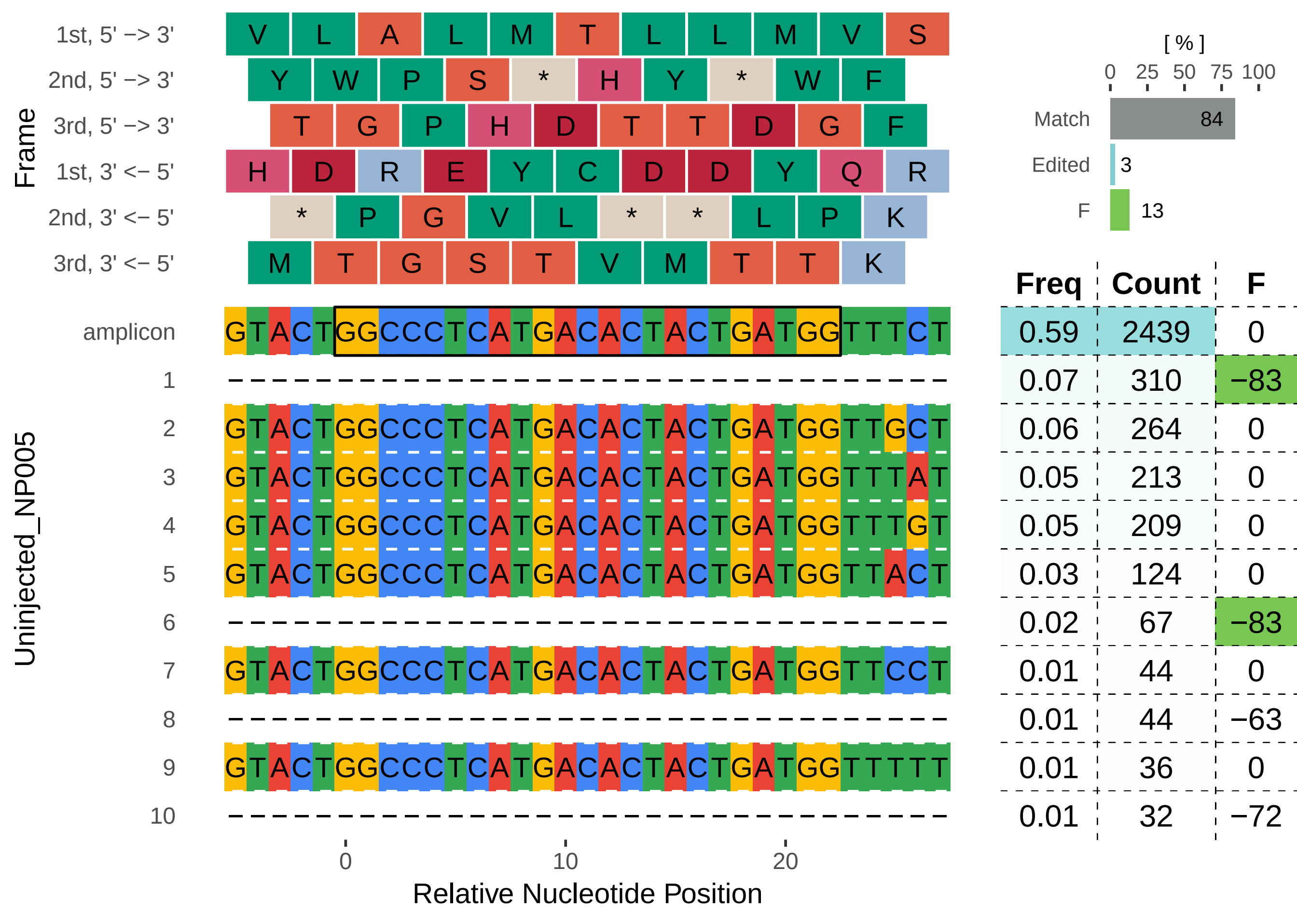
Frame
1st, 5' -> 3'
V L A L M T L L M V S
2nd, 5' -> 3'
Y W P S * H Y * W F
3rd, 5' -> 3'
T G P H D T T D G F
1st, 3' <- 5'
H D R E Y C D D Y Q R
2nd, 3' <- 5'
* P G V L * * L P K
3rd, 3' <- 5'
M T G S T V M T T K
[%]
0 25 50 75 100
Match 84
Edited 3
F 13
Freq Count F
amplicon GTACTGGCCCTCATGACACTACTGATGGTTTCT 0.59 2439 0
1 0.07 310 −83
2 GTACTGGCCCTCATGACACTACTGATGGTTGCT 0.06 264 0
3 GTACTGGCCCTCATGACACTACTGATGGTTTAT 0.05 213 0
4 GTACTGGCCCTCATGACACTACTGATGGTTTGT 0.05 209 0
5 GTACTGGCCCTCATGACACTACTGATGGTTACT 0.03 124 0
6 0.02 67 −83
7 GTACTGGCCCTCATGACACTACTGATGGTTCCT 0.01 44 0
8 0.01 44 −63
9 GTACTGGCCCTCATGACACTACTGATGGTTTTT 0.01 36 0
10 0.01 32 −72
Uninjected_NP005
0 10 20
Relative Nucleotide Position
