# Supplementary material for: Accurate analysis of genuine CRISPR editing events with ampliCan
Source: Genome Res. 2019 May;29(5):843–7. doi: 10.1101/gr.244293.118 (PMC6499316; doi:10.1101/gr.244293.118)
Supplement: Supplemental Material [file supp_gr.244293.118_Supplemental_Code_S1.zip › amplican_manuscript/figures/normalization/MiSeq_run1/Injected_SP18_control.pdf]

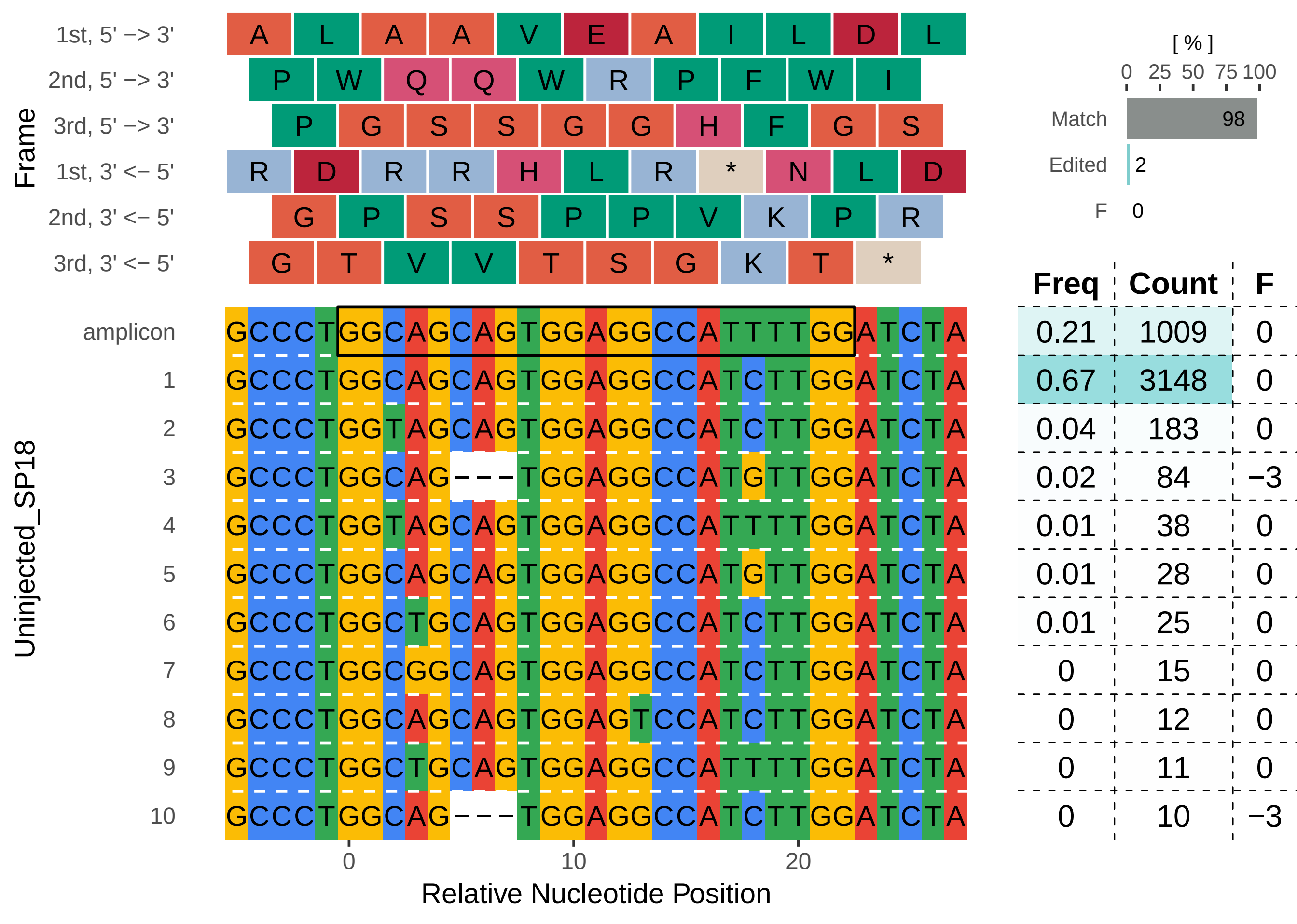
Frame
1st, 5' -> 3' A L A A V E A I L D L
2nd, 5' -> 3' P W Q Q W R P F W I
3rd, 5' -> 3' P G S S G G H F G S
1st, 3' <- 5' R D R R H L R * N L D
2nd, 3' <- 5' G P S S P P V K P R
3rd, 3' <- 5' G T V V T S G K T *
[%]
0 25 50 75 100
Match 98
Edited 2
F 0
Freq Count F
Uninjected_SP18
amplicon GCCCTGGCAGCAGTGGAGGCCATTTTGGATCTA 0.21 1009 0
1 GCCCTGGCAGCAGTGGAGGCCATCTTGGATCTA 0.67 3148 0
2 GCCCTGGTAGCAGTGGAGGCCATCTTGGATCTA 0.04 183 0
3 GCCCTGGCAG---TGGAGGCCATGTTGGATCTA 0.02 84 −3
4 GCCCTGGTAGCAGTGGAGGCCATTTTGGATCTA 0.01 38 0
5 GCCCTGGCAGCAGTGGAGGCCATGTTGGATCTA 0.01 28 0
6 GCCCTGGCTGCAGTGGAGGCCATCTTGGATCTA 0.01 25 0
7 GCCCTGGCGGCAGTGGAGGCCATCTTGGATCTA 0 15 0
8 GCCCTGGCAGCAGTGGAGTCCATCTTGGATCTA 0 12 0
9 GCCCTGGCTGCAGTGGAGGCCATTTTGGATCTA 0 11 0
10 GCCCTGGCAG---TGGAGGCCATCTTGGATCTA 0 10 −3
0 10 20
Relative Nucleotide Position
