# Supplementary material for: Accurate analysis of genuine CRISPR editing events with ampliCan
Source: Genome Res. 2019 May;29(5):843–7. doi: 10.1101/gr.244293.118 (PMC6499316; doi:10.1101/gr.244293.118)
Supplement: Supplemental Material [file supp_gr.244293.118_Supplemental_Code_S1.zip › amplican_manuscript/figures/normalization/MiSeq_run1/Injected_SP18_raw.pdf]

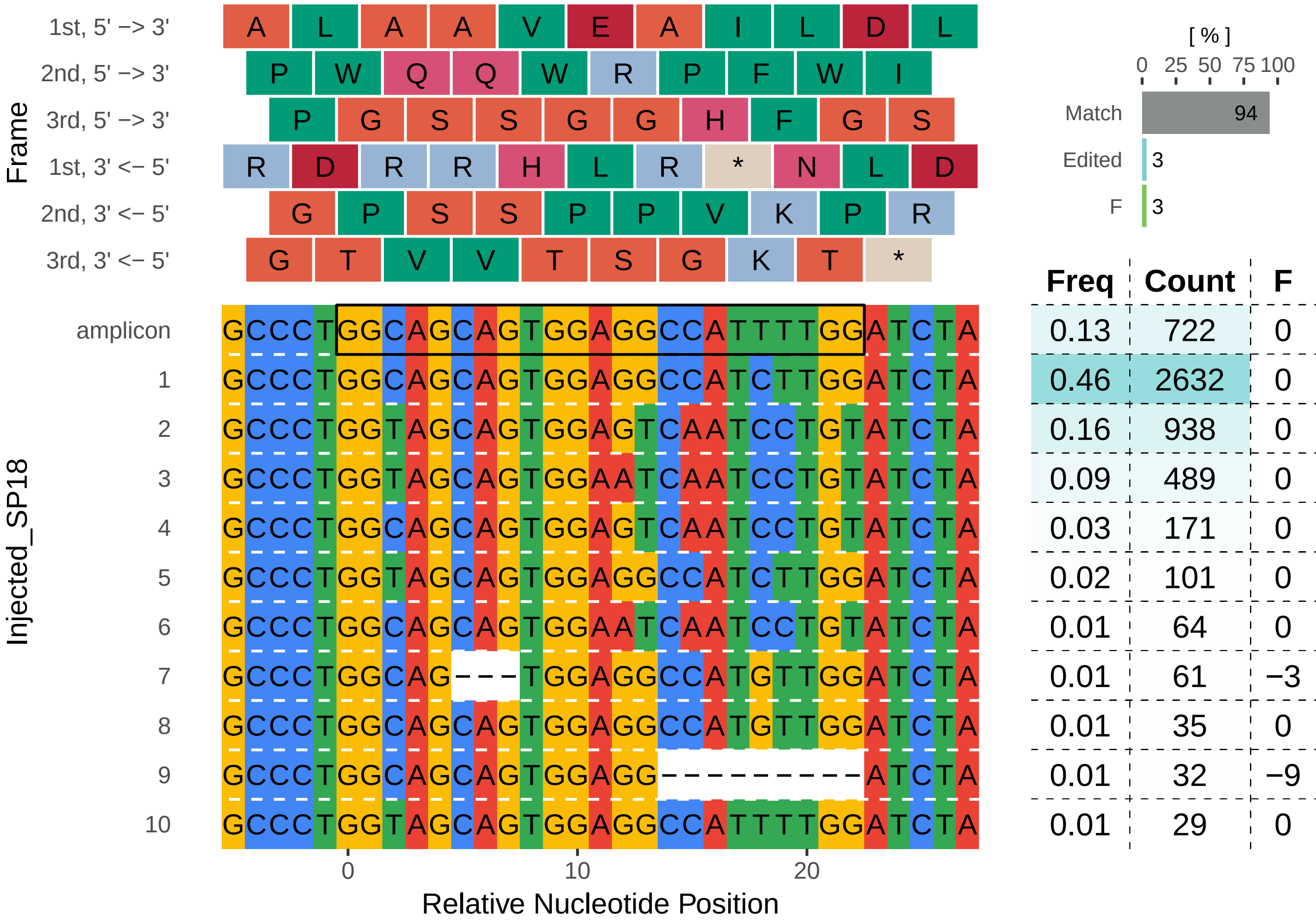
Frame
1st, 5' –> 3'
A L A A V E A I L D L
2nd, 5' –> 3'
P W Q Q W R P F W I
3rd, 5' –> 3'
P G S S G G H F G S
1st, 3' <– 5'
R D R R H L R * N L D
2nd, 3' <– 5'
G P S S P P V K P R
3rd, 3' <– 5'
G T V V T S G K T *
[%]
0 25 50 75 100
Match 94
Edited 3
F 3
Injected_SP18
amplicon GCCCTGGCAGCAGTGGAGGCCATTTTGGATCTA
1 GCCCTGGCAGCAGTGGAGGCCATCTTGGATCTA
2 GCCCTGGTAGCAGTGGAGTCAATCCTGTATCTA
3 GCCCTGGTAGCAGTGGAATCAATCCTGTATCTA
4 GCCCTGGCAGCAGTGGAGTCAATCCTGTATCTA
5 GCCCTGGTAGCAGTGGAGGCCATCTTGGATCTA
6 GCCCTGGCAGCAGTGGAATCAATCCTGTATCTA
7 GCCCTGGCAG---TGGAGGCCATGTTGGATCTA
8 GCCCTGGCAGCAGTGGAGGCCATGTTGGATCTA
9 GCCCTGGCAGCAGTGGAGG---------ATCTA
10 GCCCTGGTAGCAGTGGAGGCCATTTTGGATCTA
Freq Count F
0.13 722 0
0.46 2632 0
0.16 938 0
0.09 489 0
0.03 171 0
0.02 101 0
0.01 64 0
0.01 61 −3
0.01 35 0
0.01 32 −9
0.01 29 0
0 10 20
Relative Nucleotide Position
