# Supplementary material for: Accurate analysis of genuine CRISPR editing events with ampliCan
Source: Genome Res. 2019 May;29(5):843–7. doi: 10.1101/gr.244293.118 (PMC6499316; doi:10.1101/gr.244293.118)
Supplement: Supplemental Material [file supp_gr.244293.118_Supplemental_Code_S1.zip › amplican_manuscript/figures/normalization/MiSeq_run1/Injected_SP1_control.pdf]

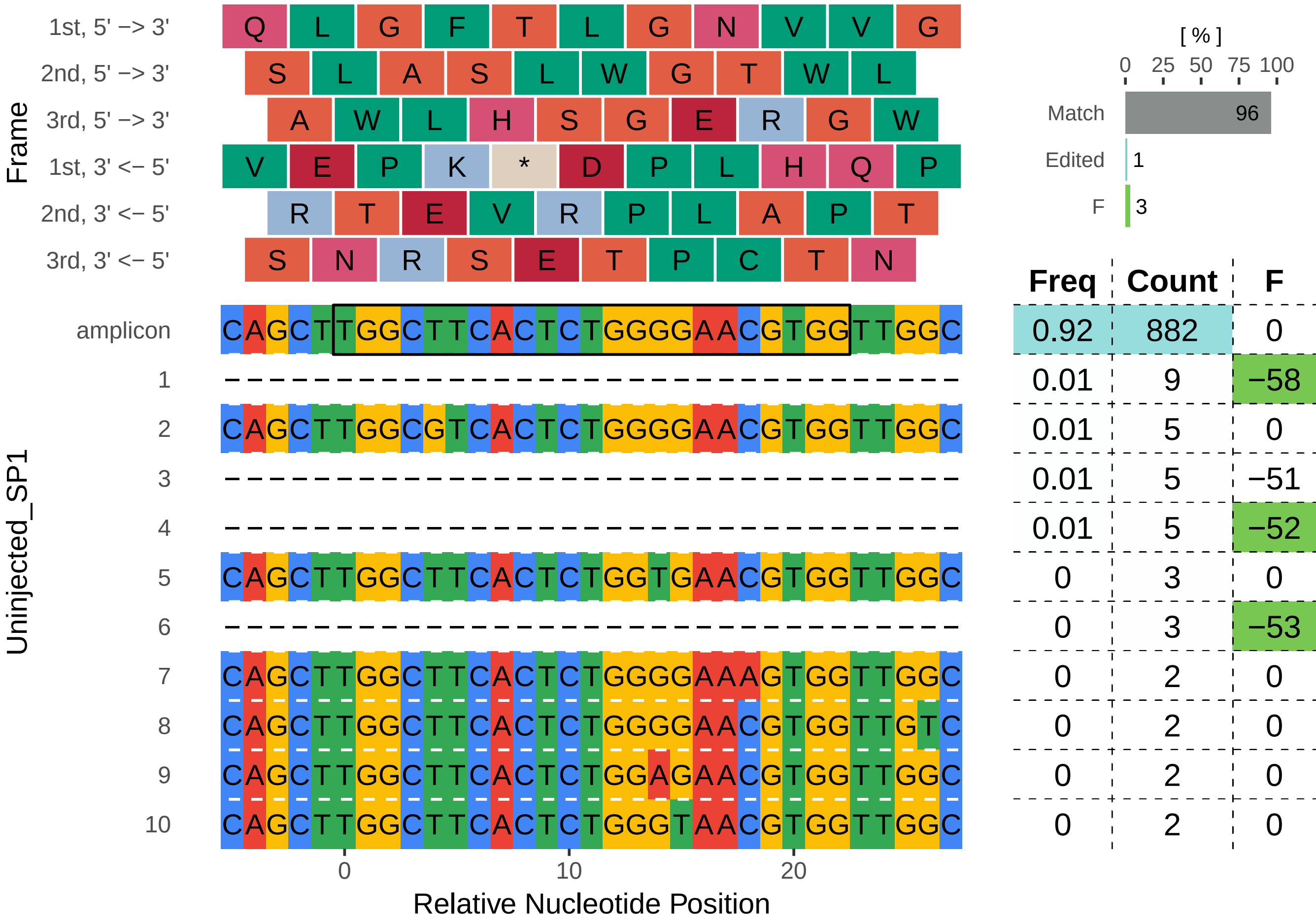

Frame
1st, 5' –> 3'
Q L G F T L G N V V G
2nd, 5' –> 3'
S L A S L W G T W L
3rd, 5' –> 3'
A W L H S G E R G W
1st, 3' <– 5'
V E P K * D P L H Q P
2nd, 3' <– 5'
R T E V R P L A P T
3rd, 3' <– 5'
S N R S E T P C T N
[%]
0 25 50 75 100
Match 96
Edited 1
F 3
Freq Count F
amplicon CAGCTTGGCTTCACTCTGGGGAACGTGGTTGGC 0.92 882 0
1 0.01 9 −58
2 CAGCTTGGCGTCACTCTGGGGAACGTGGTTGGC 0.01 5 0
3 0.01 5 −51
4 0.01 5 −52
5 CAGCTTGGCTTCACTCTGGTGAACGTGGTTGGC 0 3 0
6 0 3 −53
7 CAGCTTGGCTTCACTCTGGGGAAAGTGGTTGGC 0 2 0
8 CAGCTTGGCTTCACTCTGGGGAACGTGGTTGTC 0 2 0
9 CAGCTTGGCTTCACTCTGGAGAACGTGGTTGGC 0 2 0
10 CAGCTTGGCTTCACTCTGGGTAACGTGGTTGGC 0 2 0
Uninjected_SP1
0 10 20
Relative Nucleotide Position
