# Supplementary material for: Accurate analysis of genuine CRISPR editing events with ampliCan
Source: Genome Res. 2019 May;29(5):843–7. doi: 10.1101/gr.244293.118 (PMC6499316; doi:10.1101/gr.244293.118)
Supplement: Supplemental Material [file supp_gr.244293.118_Supplemental_Code_S1.zip › amplican_manuscript/figures/normalization/MiSeq_run5_2013_09_25/Injected_Toddler_u1_1_normalized.pdf]

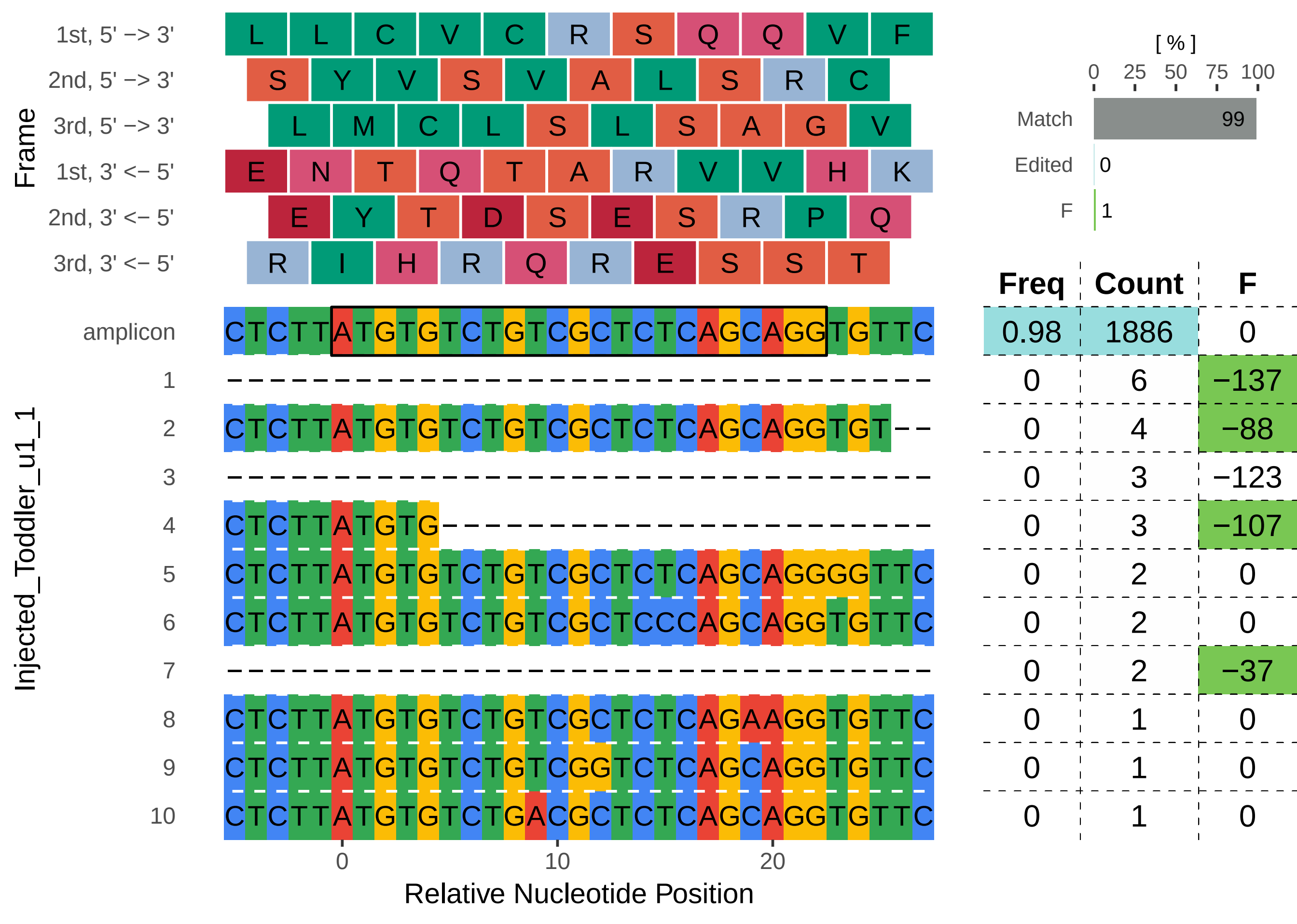
Frame
1st, 5' -> 3'
L L C V C R S Q Q V F
2nd, 5' -> 3'
S Y V S V A L S R C
3rd, 5' -> 3'
L M C L S L S A G V
1st, 3' <- 5'
E N T Q T A R V V H K
2nd, 3' <- 5'
E Y T D S E S R P Q
3rd, 3' <- 5'
R I H R Q R E S S T
[%]
0 25 50 75 100
Match 99
Edited 0
F 1
Freq Count F
amplicon CTCTTATGTGTCTGTCGCTCTCAGCAGGTGTTC 0.98 1886 0
1 0 6 −137
2 CTCTTATGTGTCTGTCGCTCTCAGCAGGTGT 0 4 −88
3 0 3 −123
4 CTCTTATGTG 0 3 −107
5 CTCTTATGTGTCTGTCGCTCTCAGCAGGGGTTC 0 2 0
6 CTCTTATGTGTCTGTCGCTCCCAGCAGGTGTTC 0 2 0
7 0 2 −37
8 CTCTTATGTGTCTGTCGCTCTCAGAAGGTGTTC 0 1 0
9 CTCTTATGTGTCTGTCGGTCTCAGCAGGTGTTC 0 1 0
10 CTCTTATGTGTCTGACGCTCTCAGCAGGTGTTC 0 1 0
Injected_Toddler_u1_1
0 10 20
Relative Nucleotide Position
