# Supplementary material for: Accurate analysis of genuine CRISPR editing events with ampliCan
Source: Genome Res. 2019 May;29(5):843–7. doi: 10.1101/gr.244293.118 (PMC6499316; doi:10.1101/gr.244293.118)
Supplement: Supplemental Material [file supp_gr.244293.118_Supplemental_Code_S1.zip › amplican_manuscript/figures/normalization/MiSeq_run7_2014_01_02/elovl6_e1_1-2_inj_control.pdf]

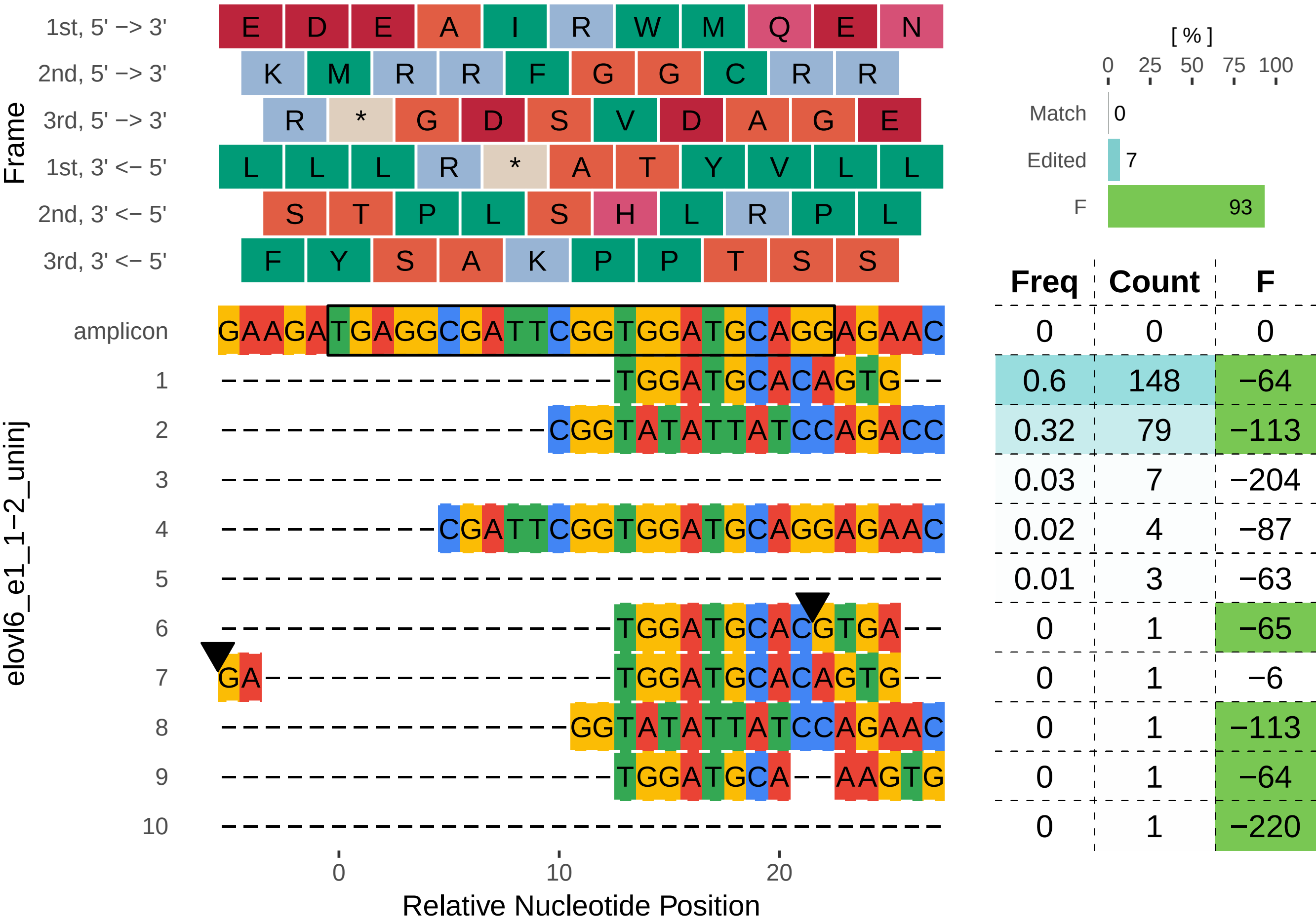

Frame
1st, 5' -> 3'
E D E A I R W M Q E N
2nd, 5' -> 3'
K M R R F G G C R R
3rd, 5' -> 3'
R * G D S V D A G E
1st, 3' <- 5'
L L L R * A T Y V L L
2nd, 3' <- 5'
S T P L S H L R P L
3rd, 3' <- 5'
F Y S A K P P T S S
[%]
0 25 50 75 100
Match 0
Edited 7
F 93
elovl6_e1_1−2_uninj
amplicon GAAGATGAGGCGATTCGGTGGATGCAGGAGAAC
1 TGGATGCACAGTG
2 CGGTATATTATCCAGACC
3
4 CGATTCGGTGGATGCAGGAGAAC
5
6 TGGATGCACGTGA
7 GA TGGATGCACAGTG
8 GGTATATTATCCAGAAC
9 TGGATGCA--AAGTG
10
Freq Count F
0 0 0
0.6 148 −64
0.32 79 −113
0.03 7 −204
0.02 4 −87
0.01 3 −63
0 1 −65
0 1 −6
0 1 −113
0 1 −64
0 1 −220
0 10 20
Relative Nucleotide Position
