# Supplementary material for: Accurate analysis of genuine CRISPR editing events with ampliCan
Source: Genome Res. 2019 May;29(5):843–7. doi: 10.1101/gr.244293.118 (PMC6499316; doi:10.1101/gr.244293.118)
Supplement: Supplemental Material [file supp_gr.244293.118_Supplemental_Code_S1.zip › amplican_manuscript/figures/normalization/MiSeq_run7_2014_01_02/SP1_inj_control.pdf]

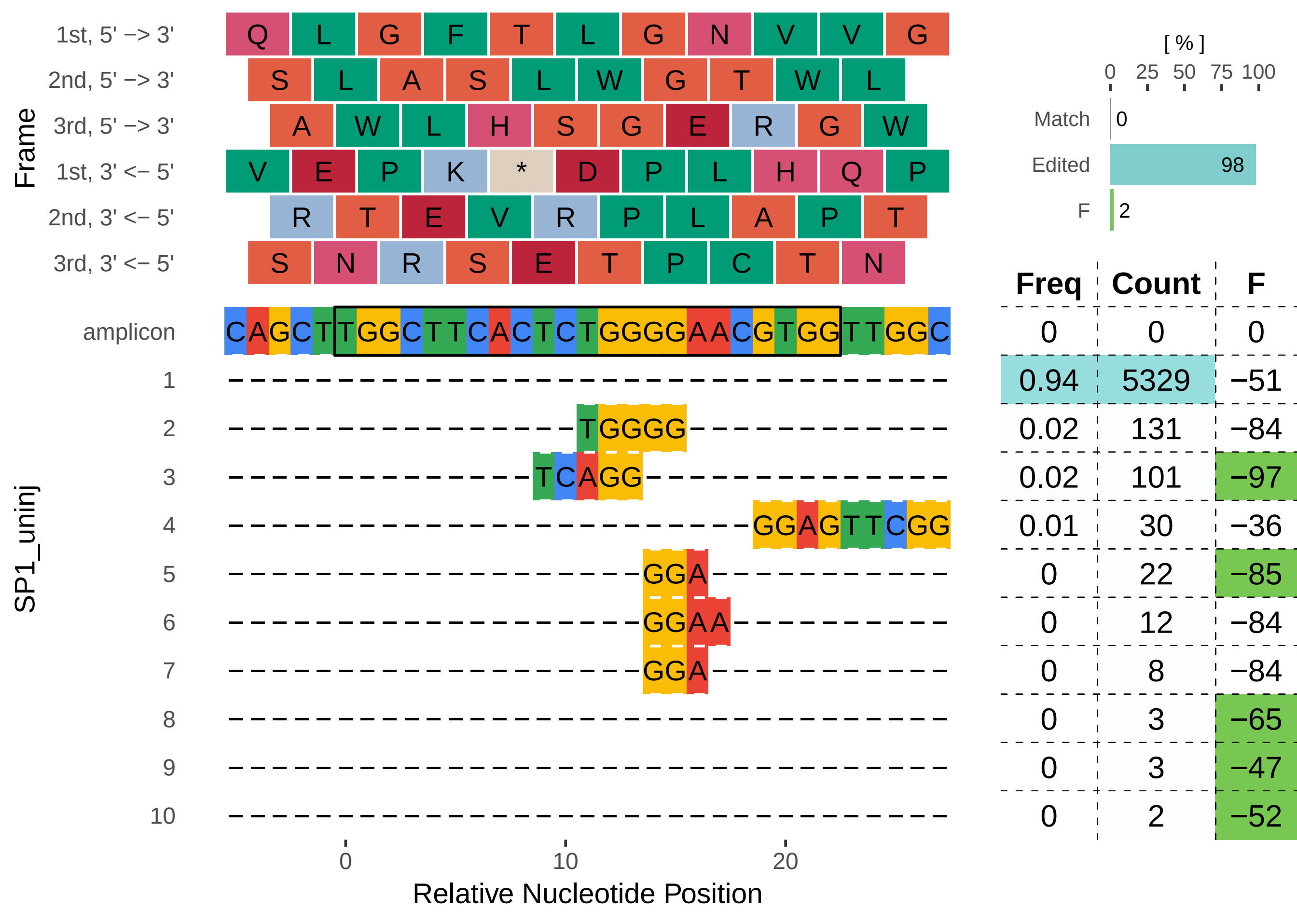

Frame
1st, 5' –> 3'
Q L G F T L G N V V G
2nd, 5' –> 3'
S L A S L W G T W L
3rd, 5' –> 3'
A W L H S G E R G W
1st, 3' <– 5'
V E P K * D P L H Q P
2nd, 3' <– 5'
R T E V R P L A P T
3rd, 3' <– 5'
S N R S E T P C T N
[%]
0 25 50 75 100
Match 0
Edited 98
F 2
amplicon
CAGCTTGGCTTCACTCTGGGGAACGTGGTTGGC
SP1_uninj
1 2 3 4 5 6 7 8 9 10
TGGGG
TCAGG
GGAGTTCGG
GGA
GGAA
GGA
Freq Count F
0 0 0
0.94 5329 −51
0.02 131 −84
0.02 101 −97
0.01 30 −36
0 22 −85
0 12 −84
0 8 −84
0 3 −65
0 3 −47
0 2 −52
0 10 20
Relative Nucleotide Position
