# Supplementary material for: Accurate analysis of genuine CRISPR editing events with ampliCan
Source: Genome Res. 2019 May;29(5):843–7. doi: 10.1101/gr.244293.118 (PMC6499316; doi:10.1101/gr.244293.118)
Supplement: Supplemental Material [file supp_gr.244293.118_Supplemental_Code_S1.zip › amplican_manuscript/figures/normalization/MiSeq_run7_2014_01_02/SP1_inj_normalized.pdf]

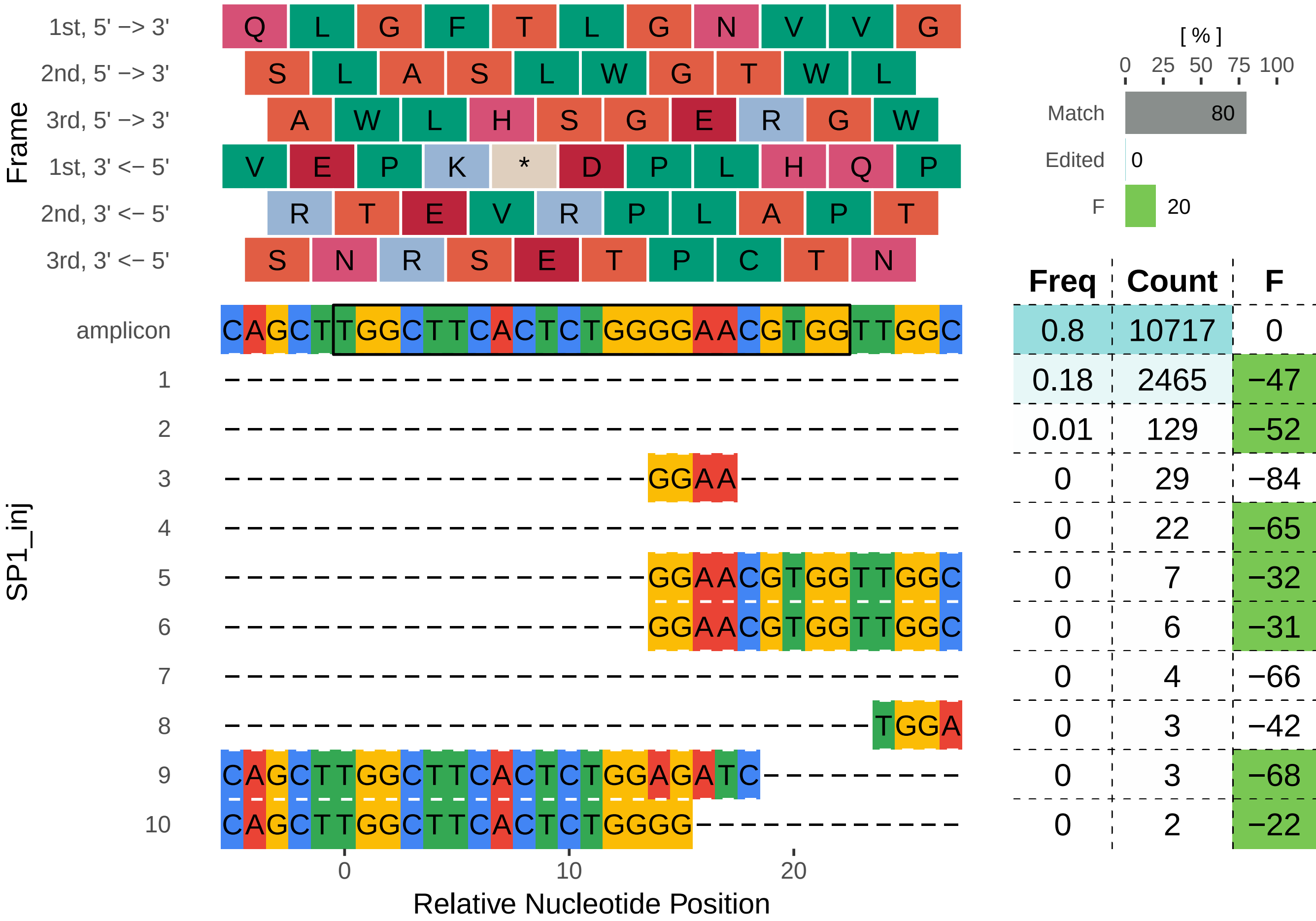
Frame
1st, 5' –> 3'
Q L G F T L G N V V G
2nd, 5' –> 3'
S L A S L W G T W L
3rd, 5' –> 3'
A W L H S G E R G W
1st, 3' <– 5'
V E P K * D P L H Q P
2nd, 3' <– 5'
R T E V R P L A P T
3rd, 3' <– 5'
S N R S E T P C T N
[%]
0 25 50 75 100
Match 80
Edited 0
F 20
amplicon CAGCTTGGCTTCACTCTGGGGAACGTGGTTGGC
SP1_inj
Freq Count F
0.8 10717 0
1 0.18 2465 −47
2 0.01 129 −52
3 GGAA 0 29 −84
4 0 22 −65
5 GGAACGTGGTTGGC 0 7 −32
6 GGAACGTGGTTGGC 0 6 −31
7 0 4 −66
8 TGGA 0 3 −42
9 CAGCTTGGCTTCACTCTGGAGATC 0 3 −68
10 CAGCTTGGCTTCACTCTGGGG 0 2 −22
0 10 20
Relative Nucleotide Position
