# Supplementary material for: Accurate analysis of genuine CRISPR editing events with ampliCan
Source: Genome Res. 2019 May;29(5):843–7. doi: 10.1101/gr.244293.118 (PMC6499316; doi:10.1101/gr.244293.118)
Supplement: Supplemental Material [file supp_gr.244293.118_Supplemental_Code_S1.zip › amplican_manuscript/figures/normalization/MiSeq_run7_2014_01_02/SP1_inj_raw.pdf]

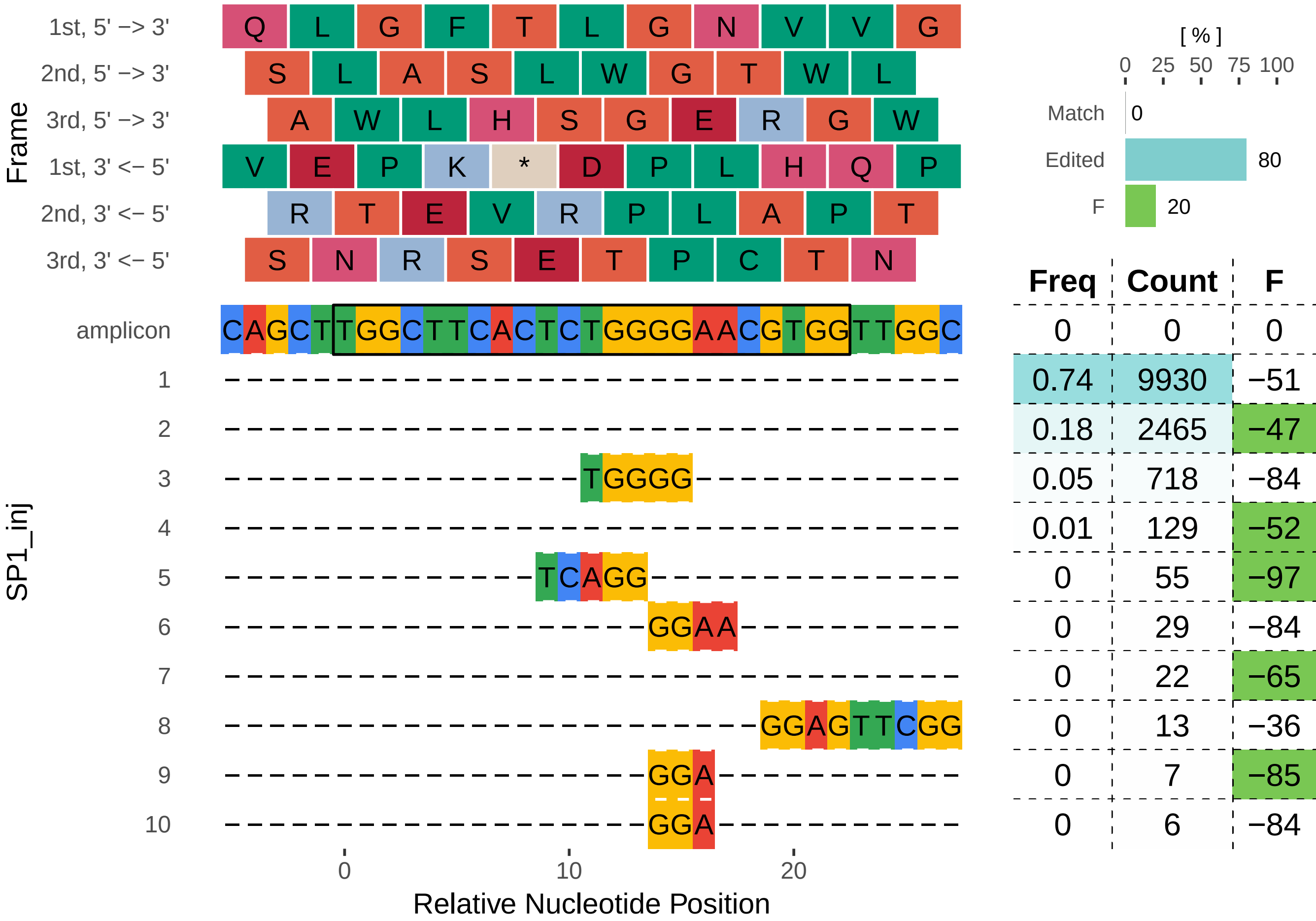
Frame
1st, 5' –> 3'
Q L G F T L G N V V G
2nd, 5' –> 3'
S L A S L W G T W L
3rd, 5' –> 3'
A W L H S G E R G W
1st, 3' <– 5'
V E P K * D P L H Q P
2nd, 3' <– 5'
R T E V R P L A P T
3rd, 3' <– 5'
S N R S E T P C T N
amplicon
CAGCTTGGCTTCACTCTGGGGAACGTGGTTGGC
SP1_inj
1
2
3
TGGGG
4
5
TCAGG
6
GGAA
7
8
GGAGTTCGG
9
GGA
10
GGA
0
10
20
Relative Nucleotide Position
[%]
0 25 50 75 100
Match 0
Edited 80
F 20
Freq Count F
0 0 0
0.74 9930 −51
0.18 2465 −47
0.05 718 −84
0.01 129 −52
0 55 −97
0 29 −84
0 22 −65
0 13 −36
0 7 −85
0 6 −84
