# Supplementary material for: Accurate analysis of genuine CRISPR editing events with ampliCan
Source: Genome Res. 2019 May;29(5):843–7. doi: 10.1101/gr.244293.118 (PMC6499316; doi:10.1101/gr.244293.118)
Supplement: Supplemental Material [file supp_gr.244293.118_Supplemental_Code_S1.zip › amplican_manuscript/figures/normalization/MiSeq_run7_2014_01_02/spaw-lnc_e1_1_inj_control.pdf]

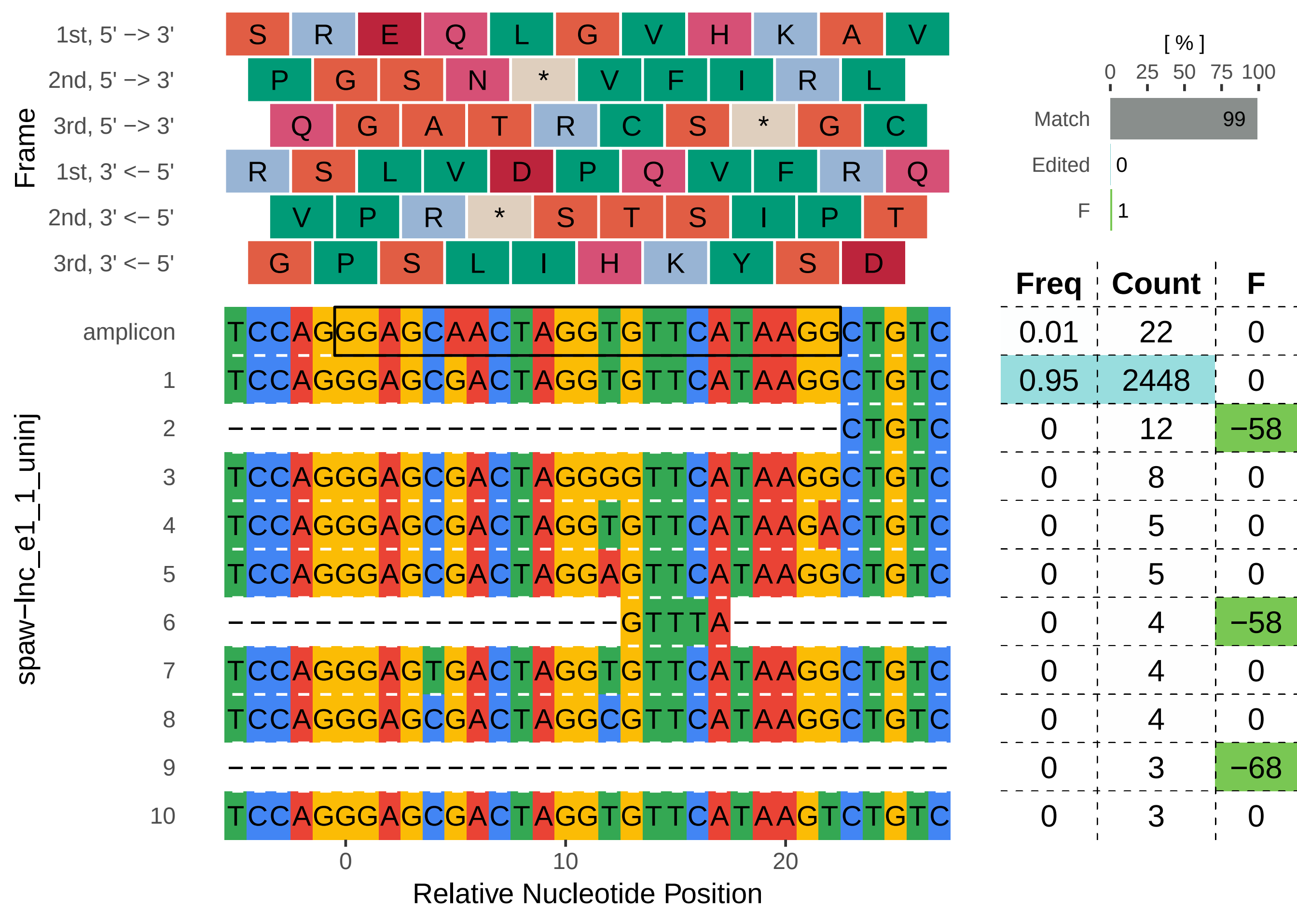
Frame
1st, 5' –> 3'
S R E Q L G V H K A V
2nd, 5' –> 3'
P G S N * V F I R L
3rd, 5' –> 3'
Q G A T R C S * G C
1st, 3' <– 5'
R S L V D P Q V F R Q
2nd, 3' <– 5'
V P R * S T S I P T
3rd, 3' <– 5'
G P S L I H K Y S D
[%]
0 25 50 75 100
Match 99
Edited 0
F 1
spaw–lnc_e1_1_uninj
amplicon TCCAGGGAGCAACTAGGTGTTCATAAGGCTGTC 0.01 22 0
1 TCCAGGGAGCGACTAGGTGTTCATAAGGCTGTC 0.95 2448 0
2 CTGTC 0 12 −58
3 TCCAGGGAGCGACTAGGGGTTCATAAGGCTGTC 0 8 0
4 TCCAGGGAGCGACTAGGTGTTCATAAGACTGTC 0 5 0
5 TCCAGGGAGCGACTAGGAGTTCATAAGGCTGTC 0 5 0
6 GTTTA 0 4 −58
7 TCCAGGGAGTGACTAGGTGTTCATAAGGCTGTC 0 4 0
8 TCCAGGGAGCGACTAGGCGTTCATAAGGCTGTC 0 4 0
9 0 3 −68
10 TCCAGGGAGCGACTAGGTGTTCATAAGTCTGTC 0 3 0
Freq Count F
0 10 20
Relative Nucleotide Position
