# Supplementary material for: Accurate analysis of genuine CRISPR editing events with ampliCan
Source: Genome Res. 2019 May;29(5):843–7. doi: 10.1101/gr.244293.118 (PMC6499316; doi:10.1101/gr.244293.118)
Supplement: Supplemental Material [file supp_gr.244293.118_Supplemental_Code_S1.zip › amplican_manuscript/figures/normalization/MiSeq_run8_2014_01_30/old_g118_1_control.pdf]

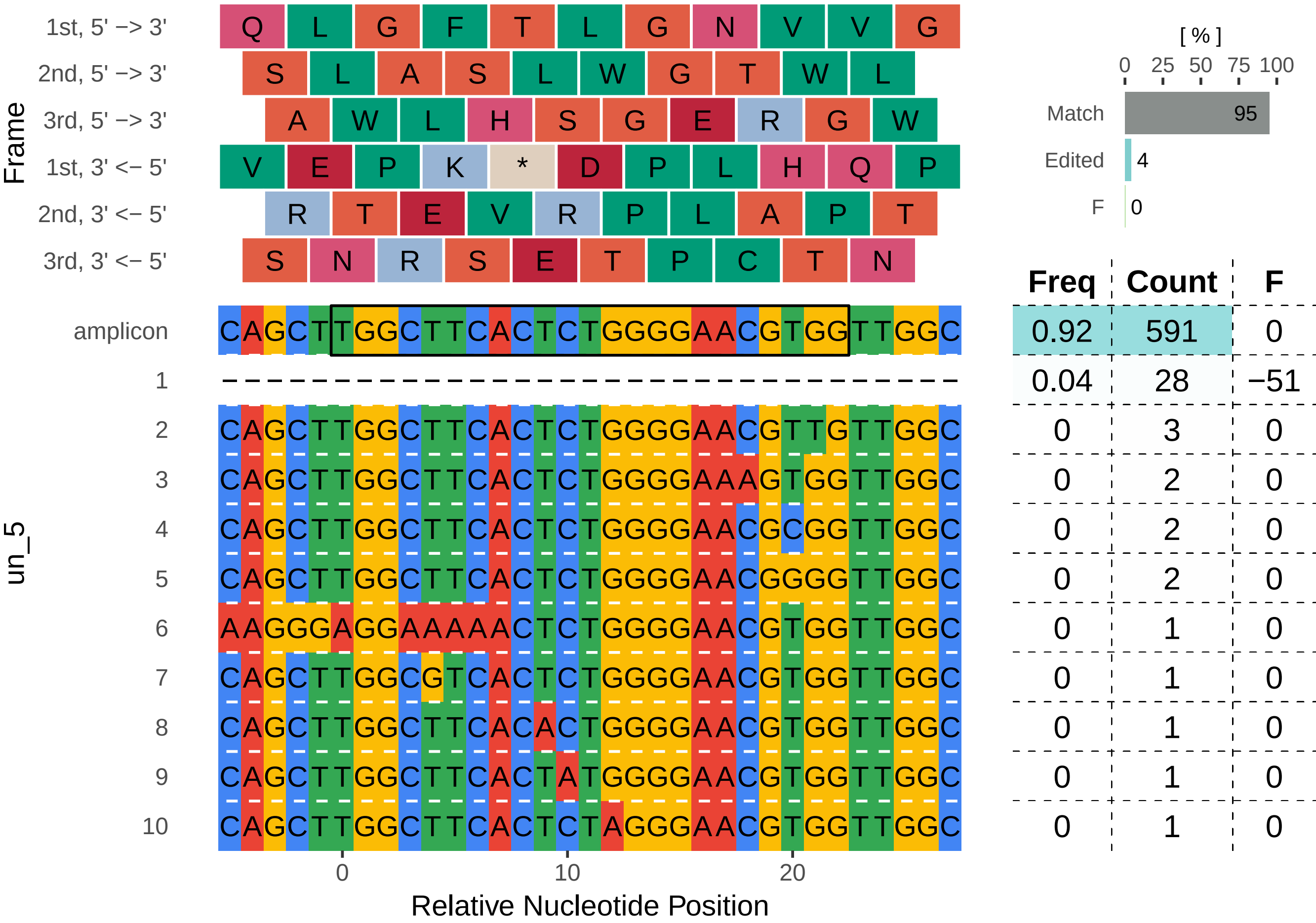
Frame
1st, 5' -> 3'
Q L G F T L G N V V G
2nd, 5' -> 3'
S L A S L W G T W L
3rd, 5' -> 3'
A W L H S G E R G W
1st, 3' <- 5'
V E P K * D P L H Q P
2nd, 3' <- 5'
R T E V R P L A P T
3rd, 3' <- 5'
S N R S E T P C T N
[%]
0 25 50 75 100
Match 95
Edited 4
F 0
Freq Count F
amplicon CAGCTTGGCTTCACTCTGGGGAACGTGGTTGGC 0.92 591 0
1 0.04 28 −51
2 CAGCTTGGCTTCACTCTGGGGAACGTTGTTGGC 0 3 0
3 CAGCTTGGCTTCACTCTGGGGAAAGTGGTTGGC 0 2 0
4 CAGCTTGGCTTCACTCTGGGGAACGCGGTTGGC 0 2 0
5 CAGCTTGGCTTCACTCTGGGGAACGGGGTTGGC 0 2 0
6 AAGGGAGGAAAAACTCTGGGGAACGTGGTTGGC 0 1 0
7 CAGCTTGGCGTCACTCTGGGGAACGTGGTTGGC 0 1 0
8 CAGCTTGGCTTCACACTGGGGAACGTGGTTGGC 0 1 0
9 CAGCTTGGCTTCACTATGGGGAACGTGGTTGGC 0 1 0
10 CAGCTTGGCTTCACTCTAGGGAACGTGGTTGGC 0 1 0
un_5
0 10 20
Relative Nucleotide Position
