# Supplementary material for: Accurate analysis of genuine CRISPR editing events with ampliCan
Source: Genome Res. 2019 May;29(5):843–7. doi: 10.1101/gr.244293.118 (PMC6499316; doi:10.1101/gr.244293.118)
Supplement: Supplemental Material [file supp_gr.244293.118_Supplemental_Code_S1.zip › amplican_manuscript/figures/normalization/MiSeq_run9_2014_03_26/213ss_normalized.pdf]

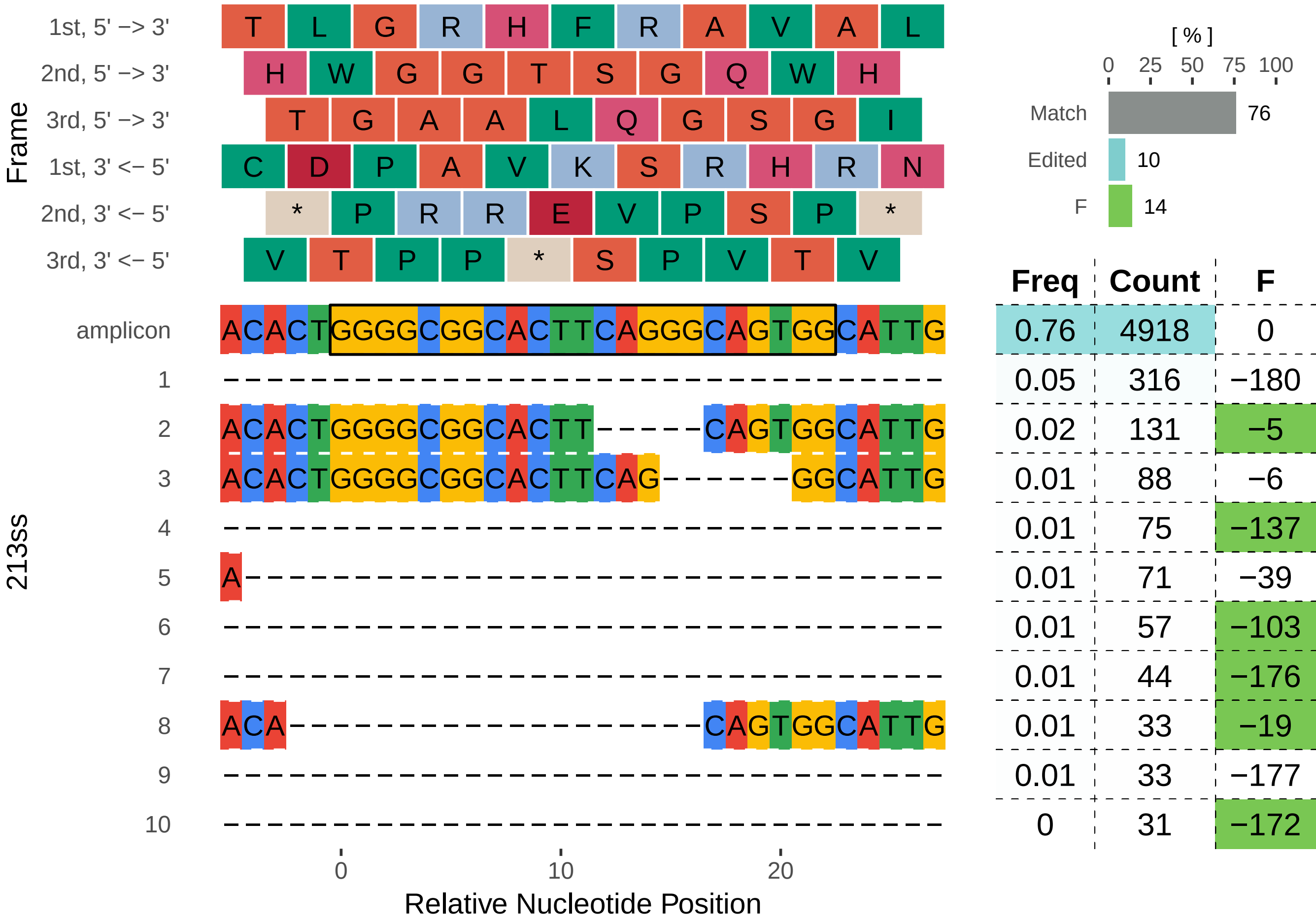

Frame
1st, 5' –> 3'
T L G R H F R A V A L
2nd, 5' –> 3'
H W G G T S G Q W H
3rd, 5' –> 3'
T G A A L Q G S G I
1st, 3' <– 5'
C D P A V K S R H R N
2nd, 3' <– 5'
* P R R E V P S P *
3rd, 3' <– 5'
V T P P * S P V T V
[%]
0 25 50 75 100
Match 76
Edited 10
F 14
Freq Count F
amplicon ACACTGGGGCGGCACTTCAGGGCAGTGGCATTG 0.76 4918 0
213ss
1 0.05 316 −180
2 ACACTGGGGCGGCACTT------CAGTGGCATTG 0.02 131 −5
3 ACACTGGGGCGGCACTTCAG------GGCATTG 0.01 88 −6
4 0.01 75 −137
5 A 0.01 71 −39
6 0.01 57 −103
7 0.01 44 −176
8 ACA------CAGTGGCATTG 0.01 33 −19
9 0.01 33 −177
10 0 31 −172
0 10 20
Relative Nucleotide Position
