# Supplementary material for: Accurate analysis of genuine CRISPR editing events with ampliCan
Source: Genome Res. 2019 May;29(5):843–7. doi: 10.1101/gr.244293.118 (PMC6499316; doi:10.1101/gr.244293.118)
Supplement: Supplemental Material [file supp_gr.244293.118_Supplemental_Code_S1.zip › amplican_manuscript/figures/normalization/MiSeq_run9_2014_03_26/213ss_raw.pdf]

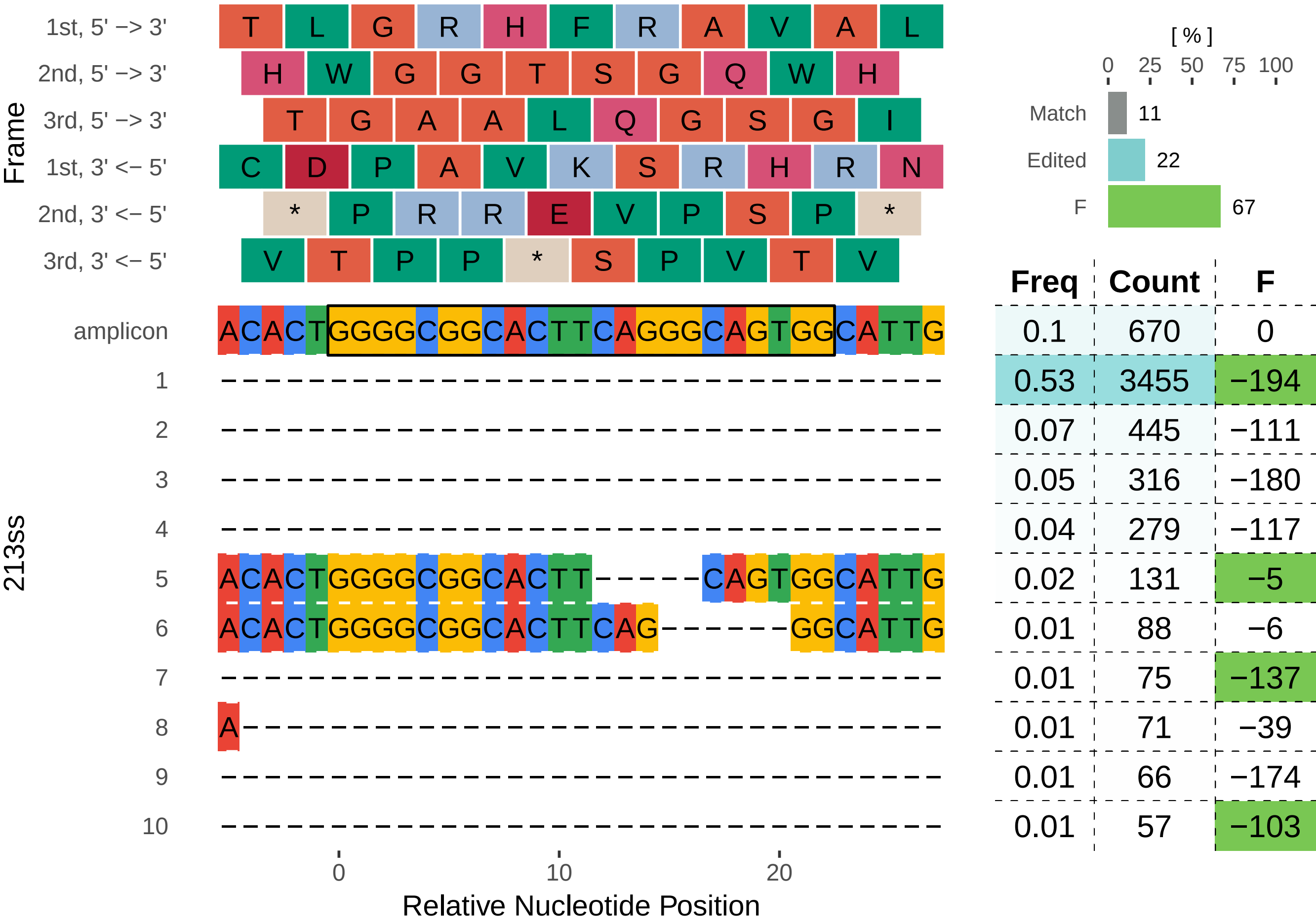
Frame
1st, 5' –> 3'
T L G R H F R A V A L
2nd, 5' –> 3'
H W G G T S G Q W H
3rd, 5' –> 3'
T G A A L Q G S G I
1st, 3' <– 5'
C D P A V K S R H R N
2nd, 3' <– 5'
* P R R E V P S P *
3rd, 3' <– 5'
V T P P * S P V T V
amplicon
ACACTGGGGCGGCACTTCAGGGCAGTGGCATTG
213ss
1
2
3
4
5 ACACTGGGGCGGCACTT------CAGTGGCATTG
6 ACACTGGGGCGGCACTTCAG------GGCATTG
7
8 A
9
10
0
10
20
Relative Nucleotide Position
[%]
0 25 50 75 100
Match 11
Edited 22
F 67
Freq Count F
0.1 670 0
0.53 3455 −194
0.07 445 −111
0.05 316 −180
0.04 279 −117
0.02 131 −5
0.01 88 −6
0.01 75 −137
0.01 71 −39
0.01 66 −174
0.01 57 −103
