# Supplementary material for: Accurate analysis of genuine CRISPR editing events with ampliCan
Source: Genome Res. 2019 May;29(5):843–7. doi: 10.1101/gr.244293.118 (PMC6499316; doi:10.1101/gr.244293.118)
Supplement: Supplemental Material [file supp_gr.244293.118_Supplemental_Code_S1.zip › amplican_manuscript/figures/normalization/MiSeq_run9_2014_03_26/GFPnoSTOP_gRNA-_normalized.pdf]

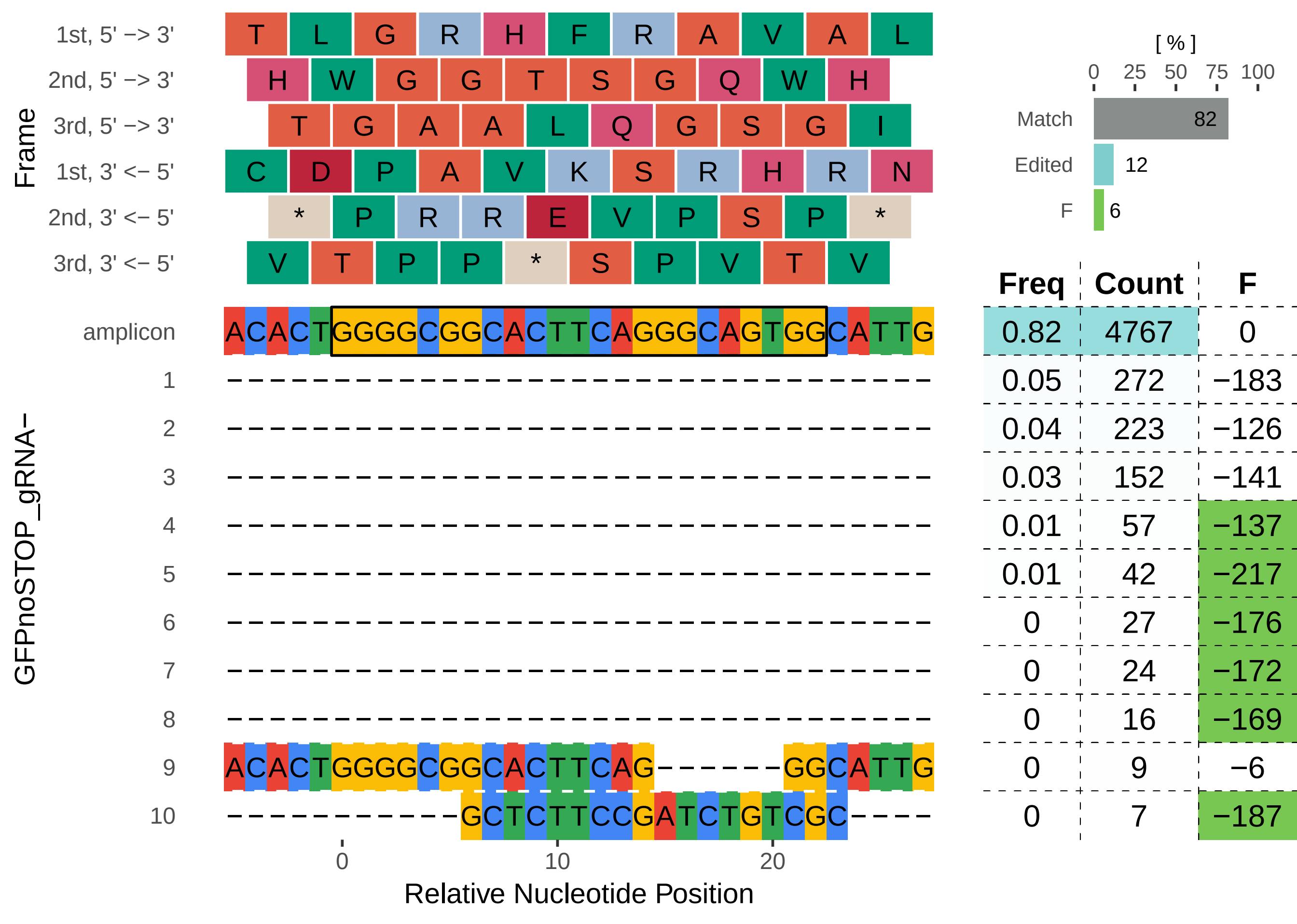
Frame
1st, 5' -> 3'
T L G R H F R A V A L
2nd, 5' -> 3'
H W G G T S G Q W H
3rd, 5' -> 3'
T G A A L Q G S G I
1st, 3' <- 5'
C D P A V K S R H R N
2nd, 3' <- 5'
* P R R E V P S P *
3rd, 3' <- 5'
V T P P * S P V T V
[%]
0 25 50 75 100
Match 82
Edited 12
F 6
amplicon
ACACTGGGGCGGCACTTCAGGGCAGTGGCATTG
GFPnoSTOP_gRNA-
Freq Count F
0.82 4767 0
1 0.05 272 −183
2 0.04 223 −126
3 0.03 152 −141
4 0.01 57 −137
5 0.01 42 −217
6 0 27 −176
7 0 24 −172
8 0 16 −169
9 ACACTGGGGCGGCACTTCAG------GGCATTG 0 9 −6
10 GCTCTTCCGATCTGTCGC 0 7 −187
0 10 20
Relative Nucleotide Position
