# Supplementary material for: Accurate analysis of genuine CRISPR editing events with ampliCan
Source: Genome Res. 2019 May;29(5):843–7. doi: 10.1101/gr.244293.118 (PMC6499316; doi:10.1101/gr.244293.118)
Supplement: Supplemental Material [file supp_gr.244293.118_Supplemental_Code_S1.zip › amplican_manuscript/figures/normalization/MiSeq_run9_2014_03_26/GFPnoSTOP_raw.pdf]

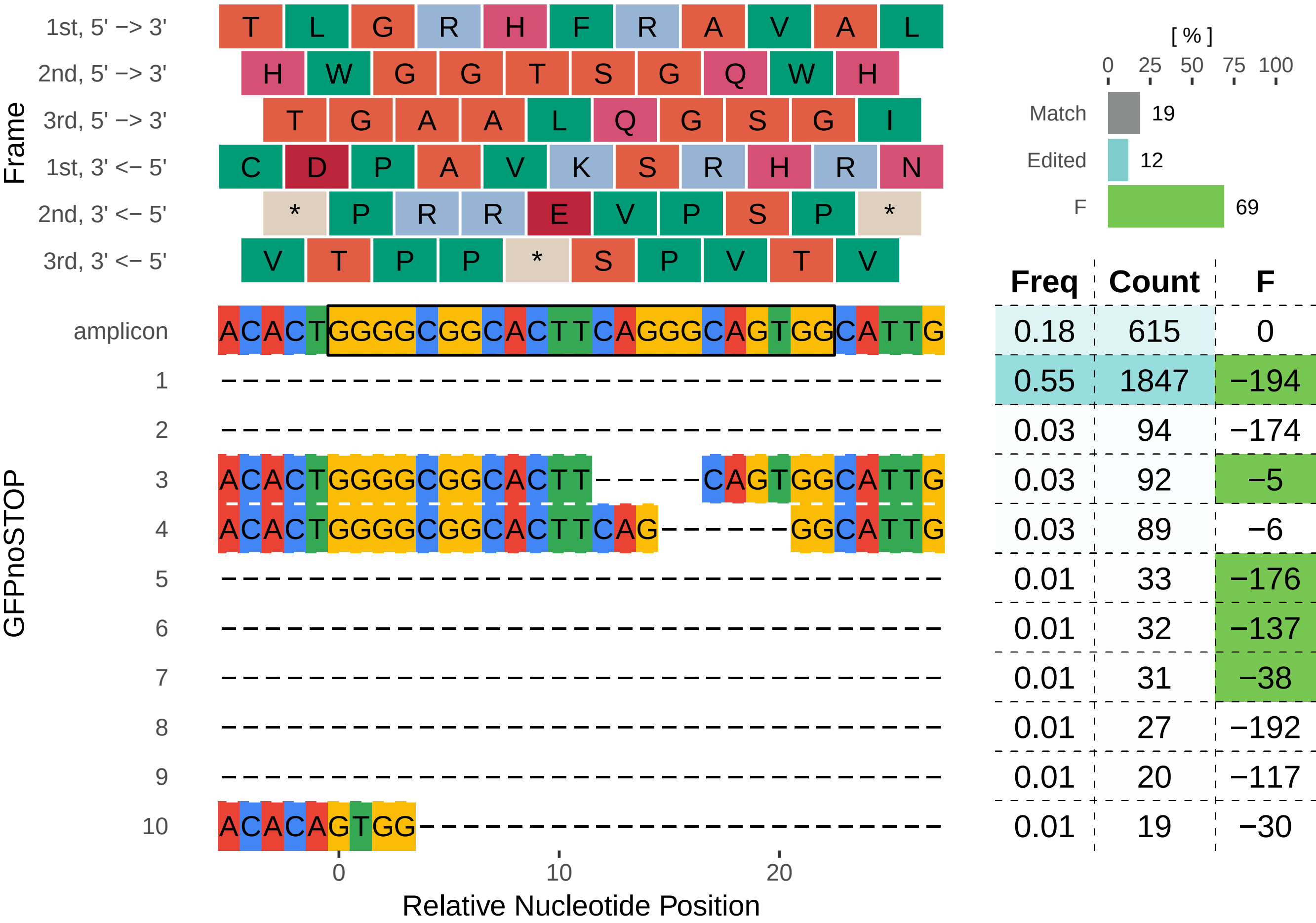
Frame
1st, 5' –> 3'
T L G R H F R A V A L
2nd, 5' –> 3'
H W G G T S G Q W H
3rd, 5' –> 3'
T G A A L Q G S G I
1st, 3' <– 5'
C D P A V K S R H R N
2nd, 3' <– 5'
* P R R E V P S P *
3rd, 3' <– 5'
V T P P * S P V T V
[%]
0 25 50 75 100
Match 19
Edited 12
F 69
Freq Count F
amplicon ACACTGGGGCGGCACTTCAGGGCAGTGGCATTG 0.18 615 0
1 0.55 1847 −194
2 0.03 94 −174
3 ACACTGGGGCGGCACTT------CAGTGGCATTG 0.03 92 −5
4 ACACTGGGGCGGCACTTCAG------GGCATTG 0.03 89 −6
5 0.01 33 −176
6 0.01 32 −137
7 0.01 31 −38
8 0.01 27 −192
9 0.01 20 −117
10 ACACAGTGG 0.01 19 −30
GFPnoSTOP
0 10 20
Relative Nucleotide Position
