# Supplementary figures and images for: Accurate analysis of genuine CRISPR editing events with ampliCan
Source: Genome Res. 2019 May;29(5):843–7. doi: 10.1101/gr.244293.118 (PMC6499316; doi:10.1101/gr.244293.118)

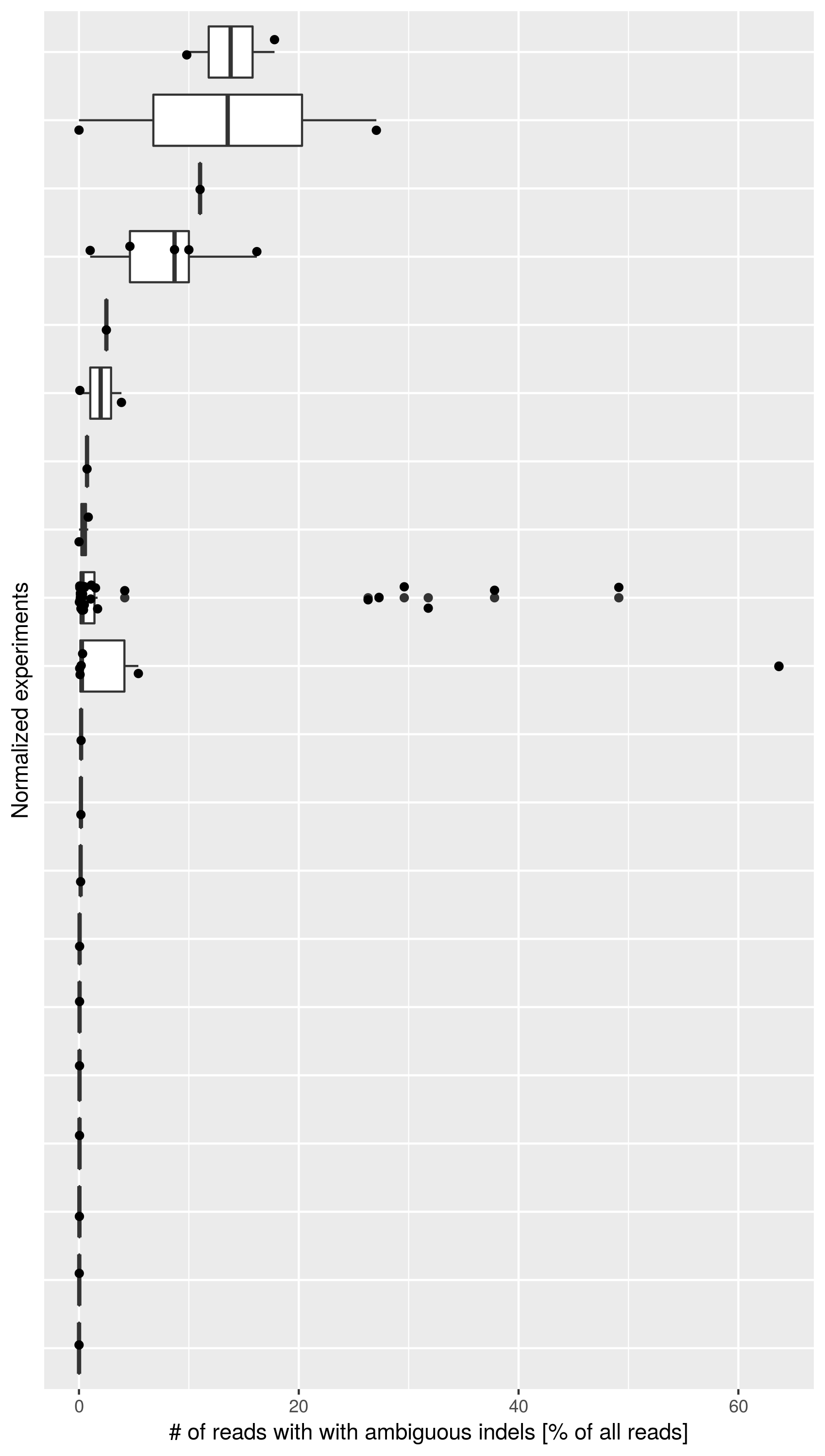

Supplement: Supplemental Material [file supp_gr.244293.118_Supplemental_Code_S1.zip › amplican_manuscript/figures/ambiguous_rate.png]

Difference to ampliCan, # of experiments [%]

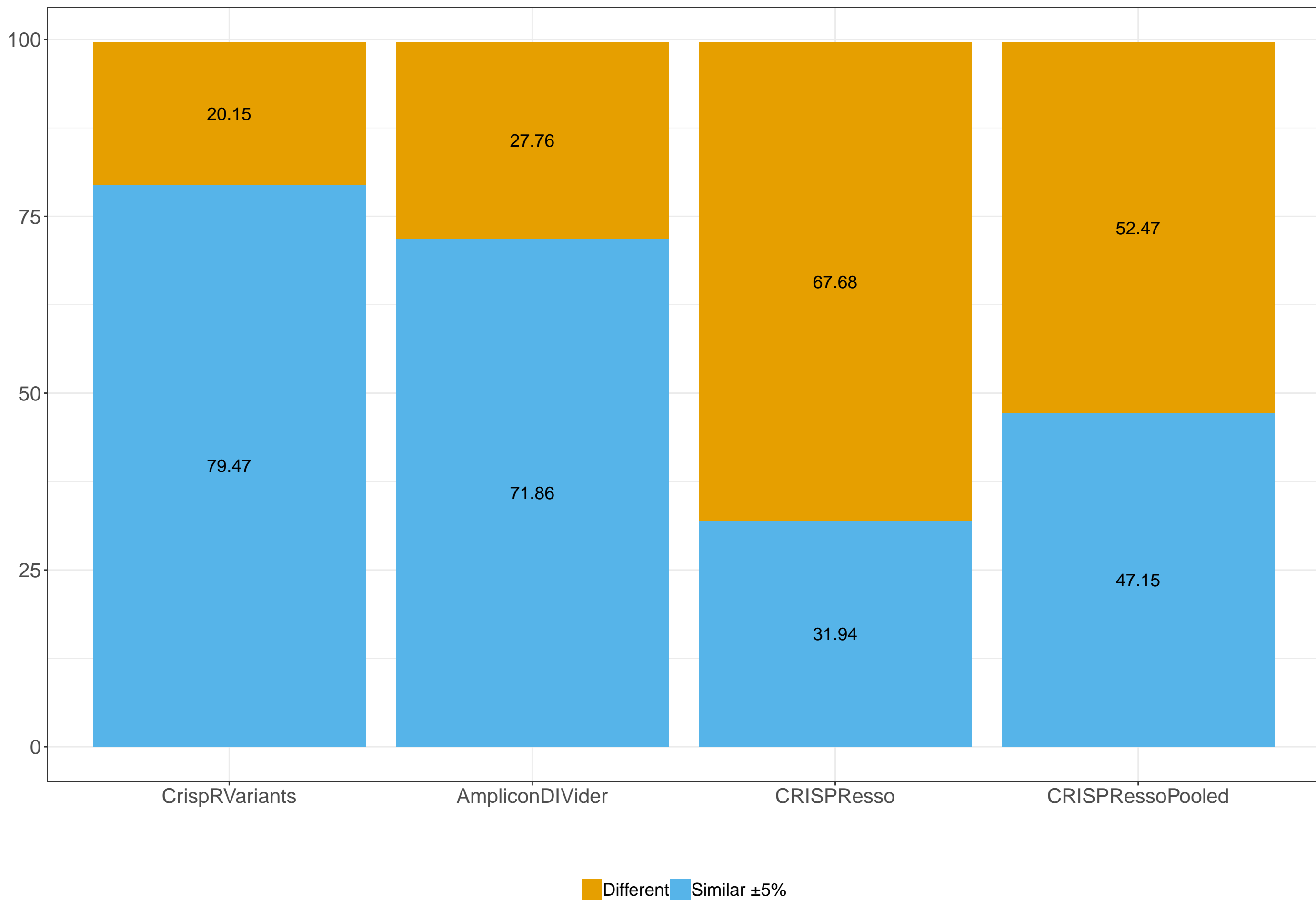

Supplement: Supplemental Material [file supp_gr.244293.118_Supplemental_Code_S1.zip › amplican_manuscript/figures/counts_rate_real_datasets.pdf]

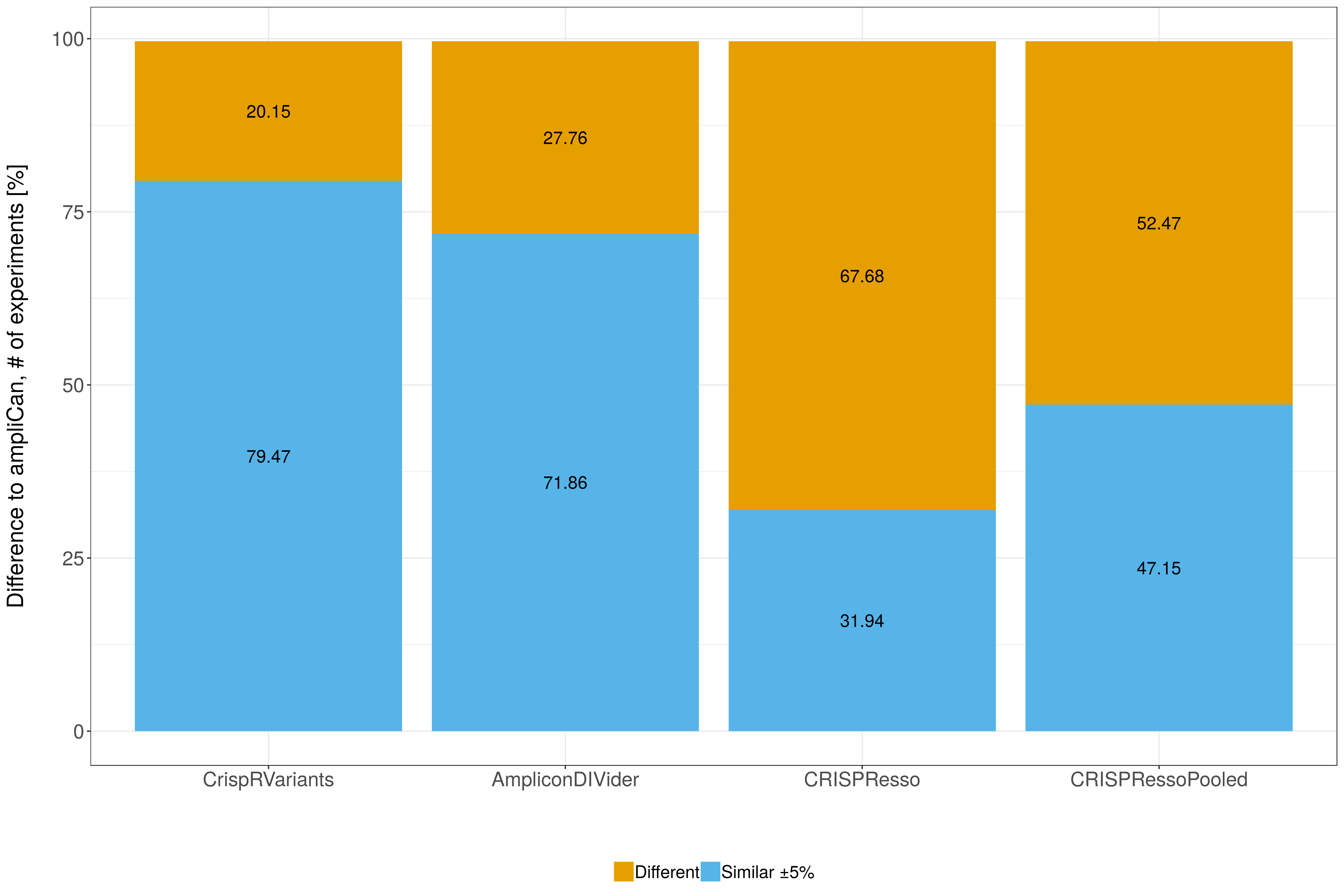

Supplement: Supplemental Material [file supp_gr.244293.118_Supplemental_Code_S1.zip › amplican_manuscript/figures/counts_real_datasets.png]

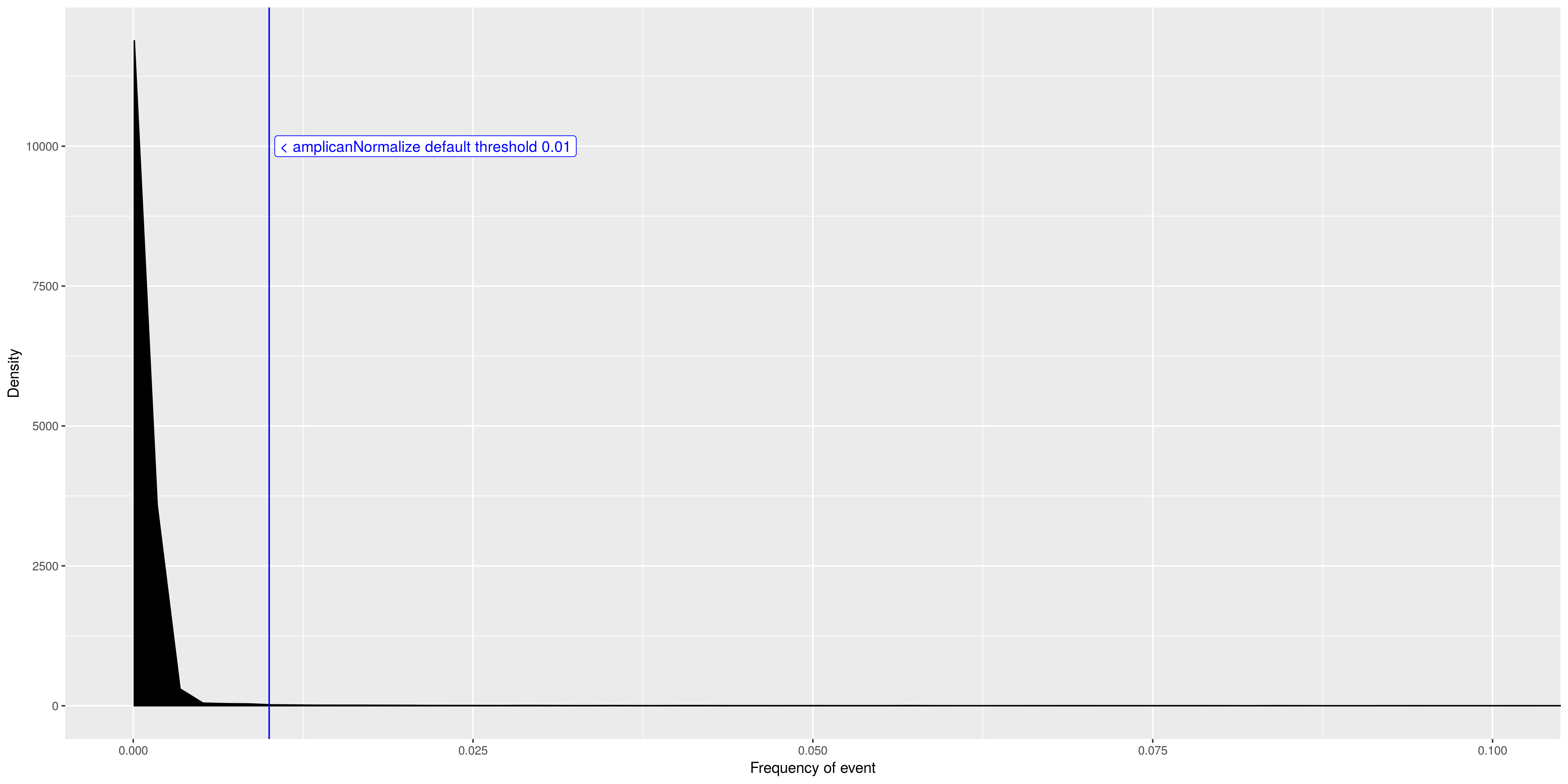

Supplement: Supplemental Material [file supp_gr.244293.118_Supplemental_Code_S1.zip › amplican_manuscript/figures/desitribution_of_control_event_freq.png]

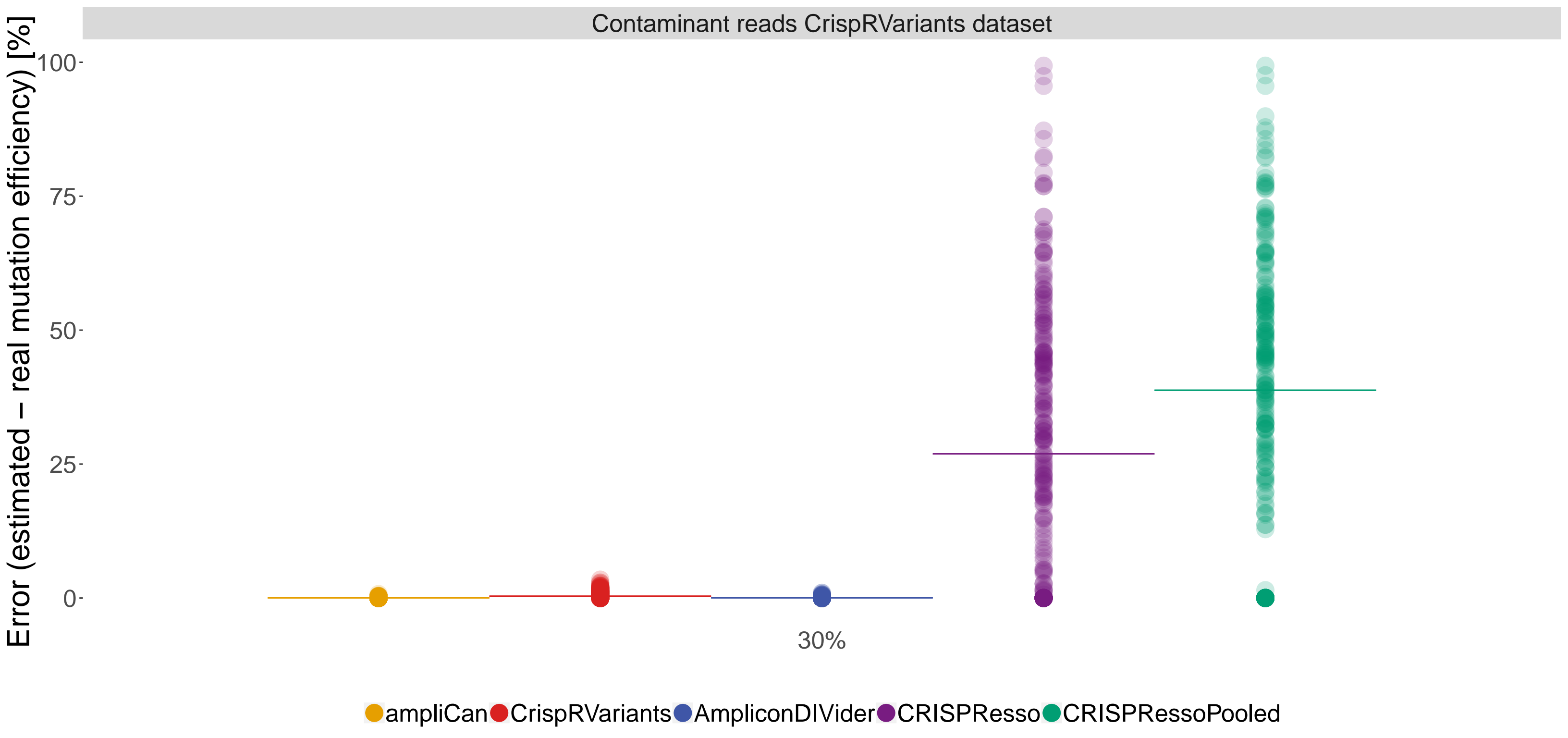

Supplement: Supplemental Material [file supp_gr.244293.118_Supplemental_Code_S1.zip › amplican_manuscript/figures/error_crisprvaraints_dataset.pdf]

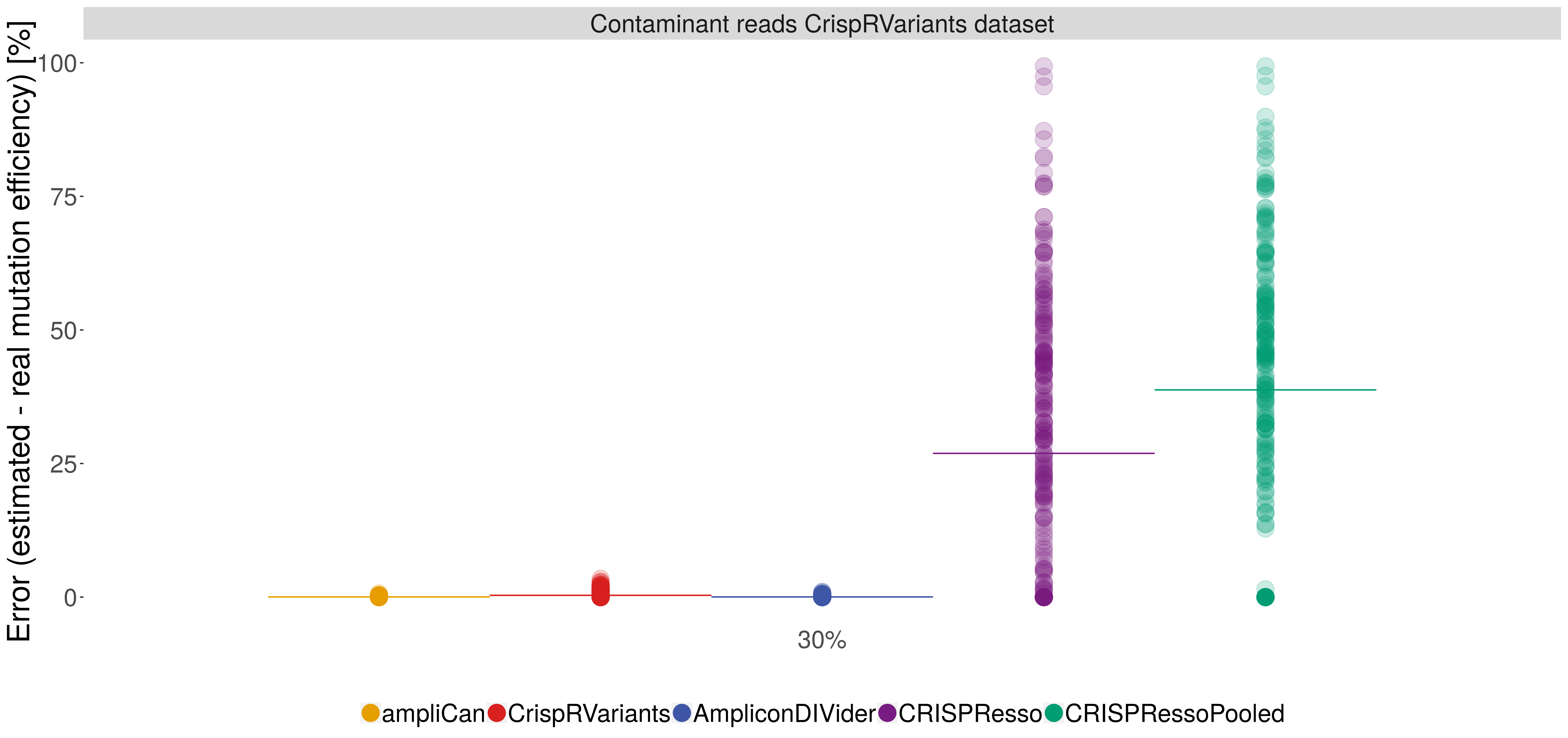

Supplement: Supplemental Material [file supp_gr.244293.118_Supplemental_Code_S1.zip › amplican_manuscript/figures/error_crisprvaraints_dataset.png]

Contaminant reads CrispRVariants dataset

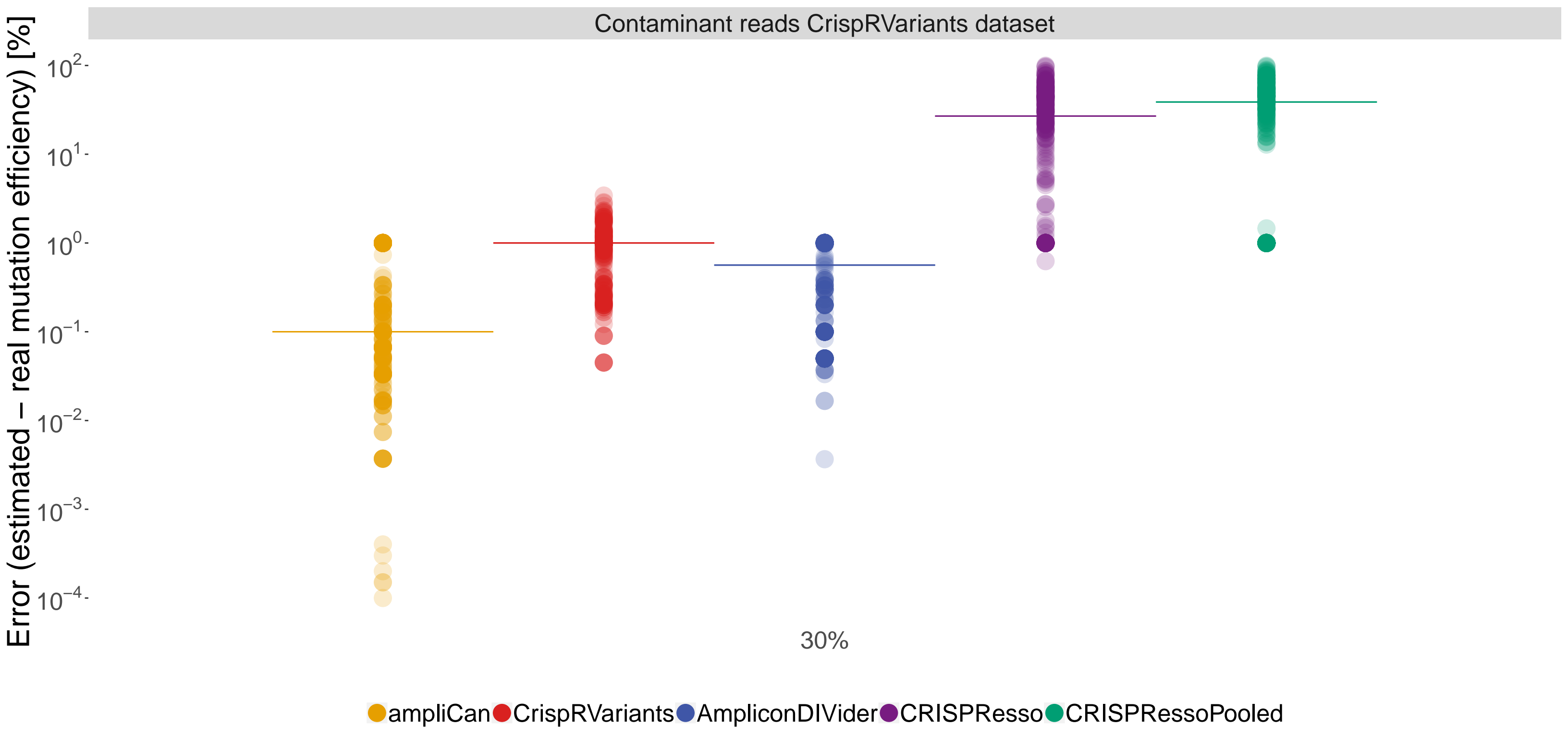

Supplement: Supplemental Material [file supp_gr.244293.118_Supplemental_Code_S1.zip › amplican_manuscript/figures/error_crisprvaraints_dataset_log.pdf]

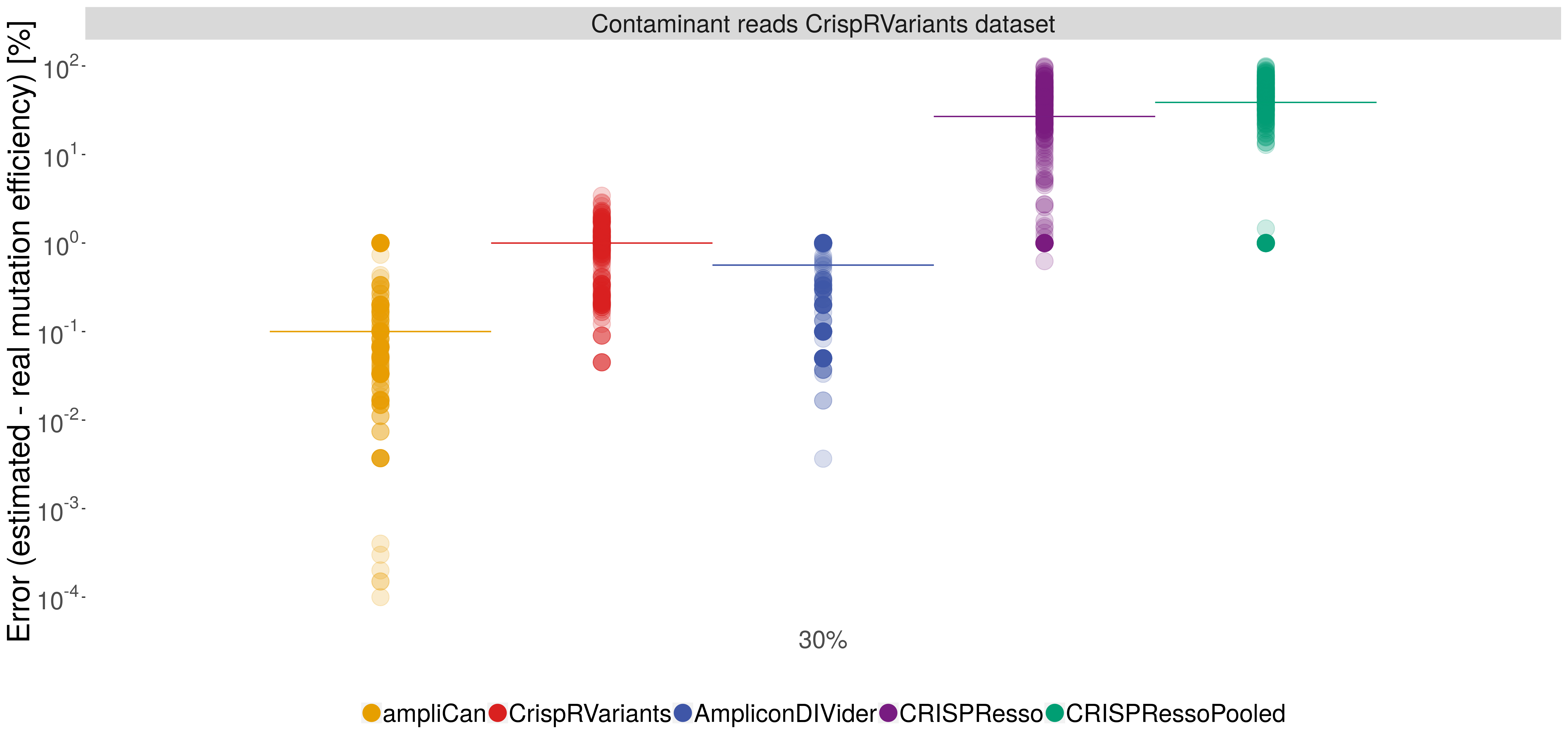

Supplement: Supplemental Material [file supp_gr.244293.118_Supplemental_Code_S1.zip › amplican_manuscript/figures/error_crisprvaraints_dataset_log.png]

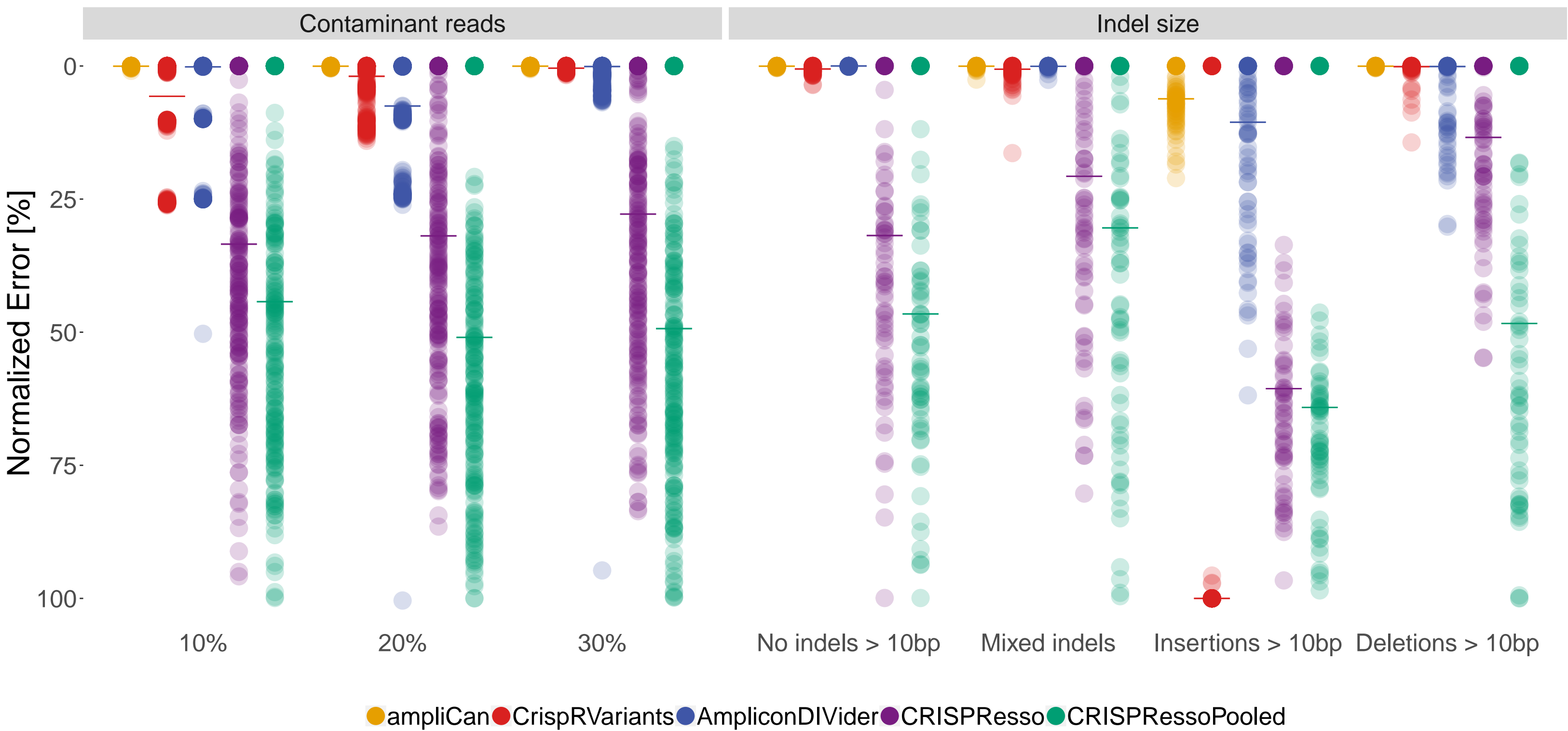

Supplement: Supplemental Material [file supp_gr.244293.118_Supplemental_Code_S1.zip › amplican_manuscript/figures/fig_2.pdf]

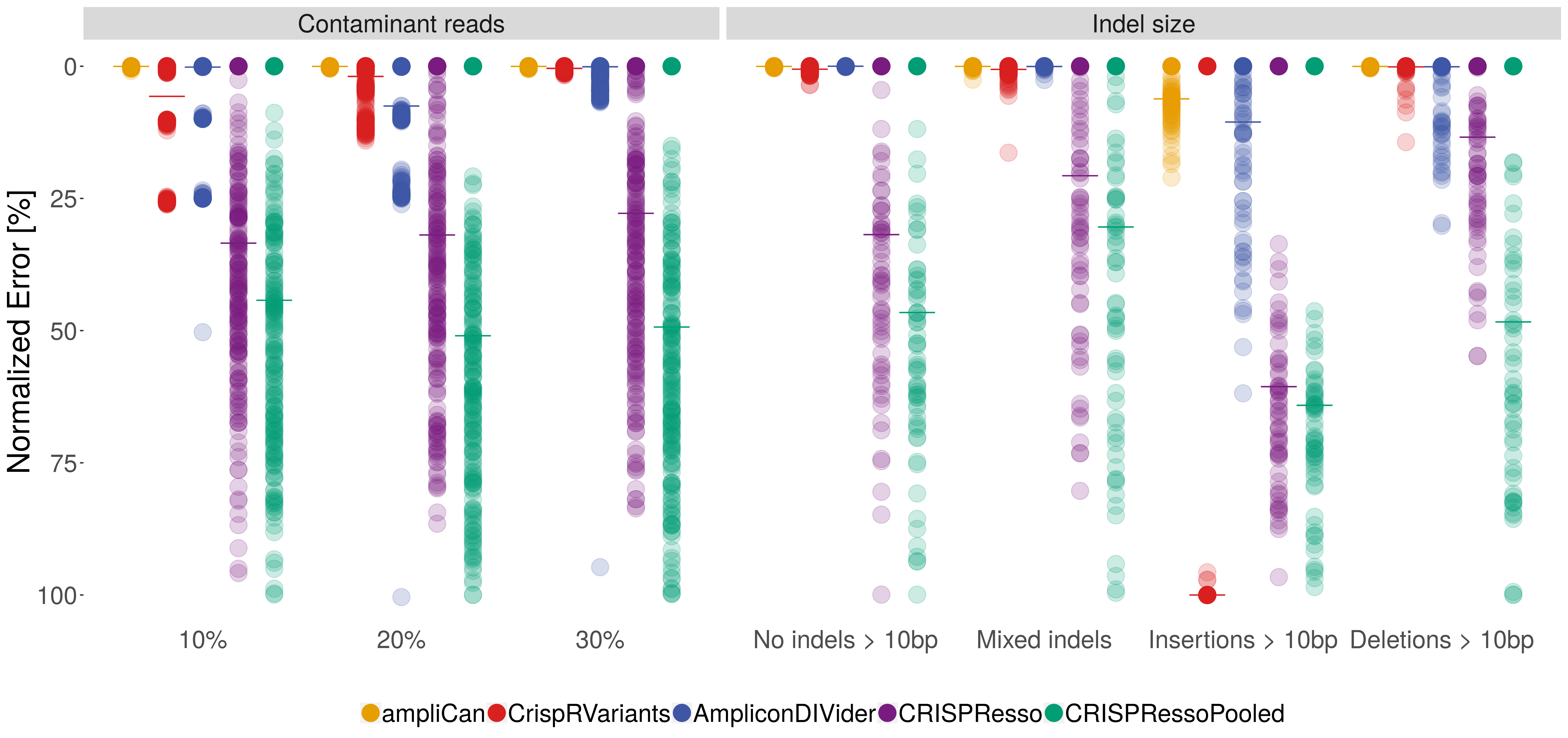

Supplement: Supplemental Material [file supp_gr.244293.118_Supplemental_Code_S1.zip › amplican_manuscript/figures/fig_2.png]

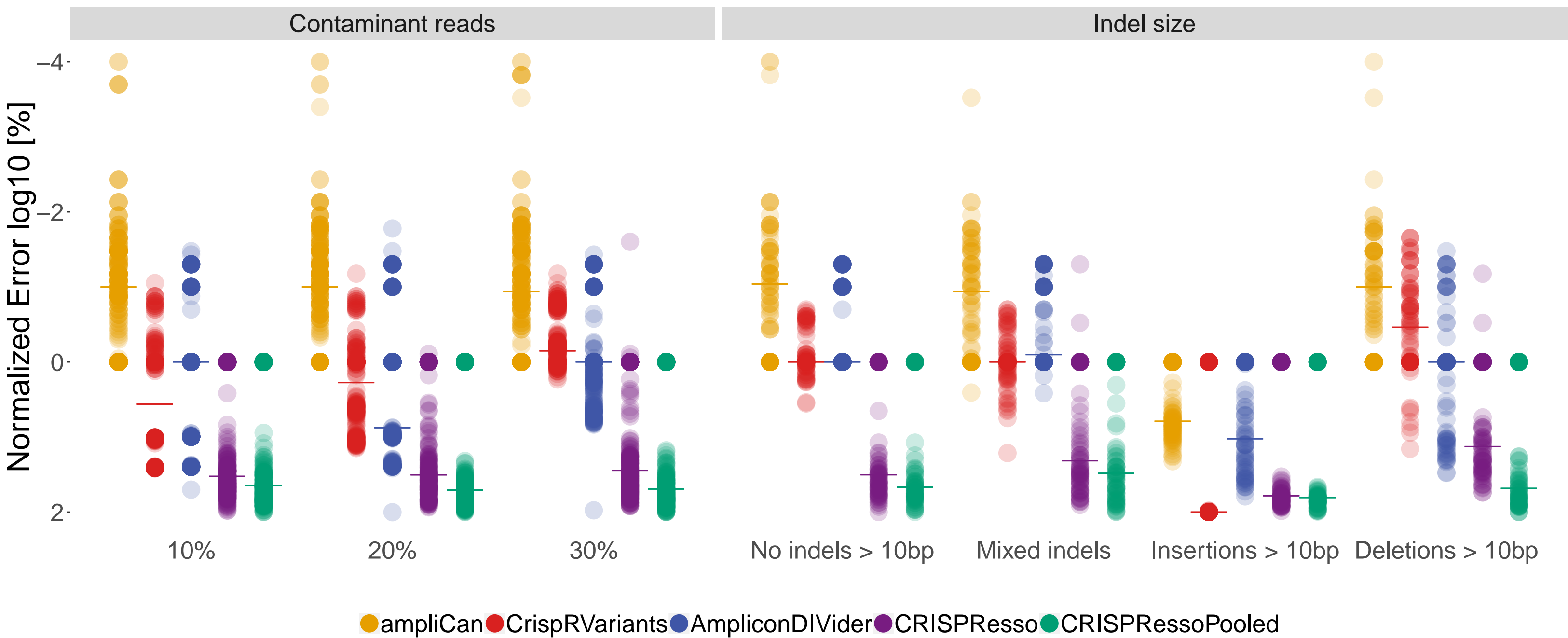

Supplement: Supplemental Material [file supp_gr.244293.118_Supplemental_Code_S1.zip › amplican_manuscript/figures/fig_2_log.pdf]

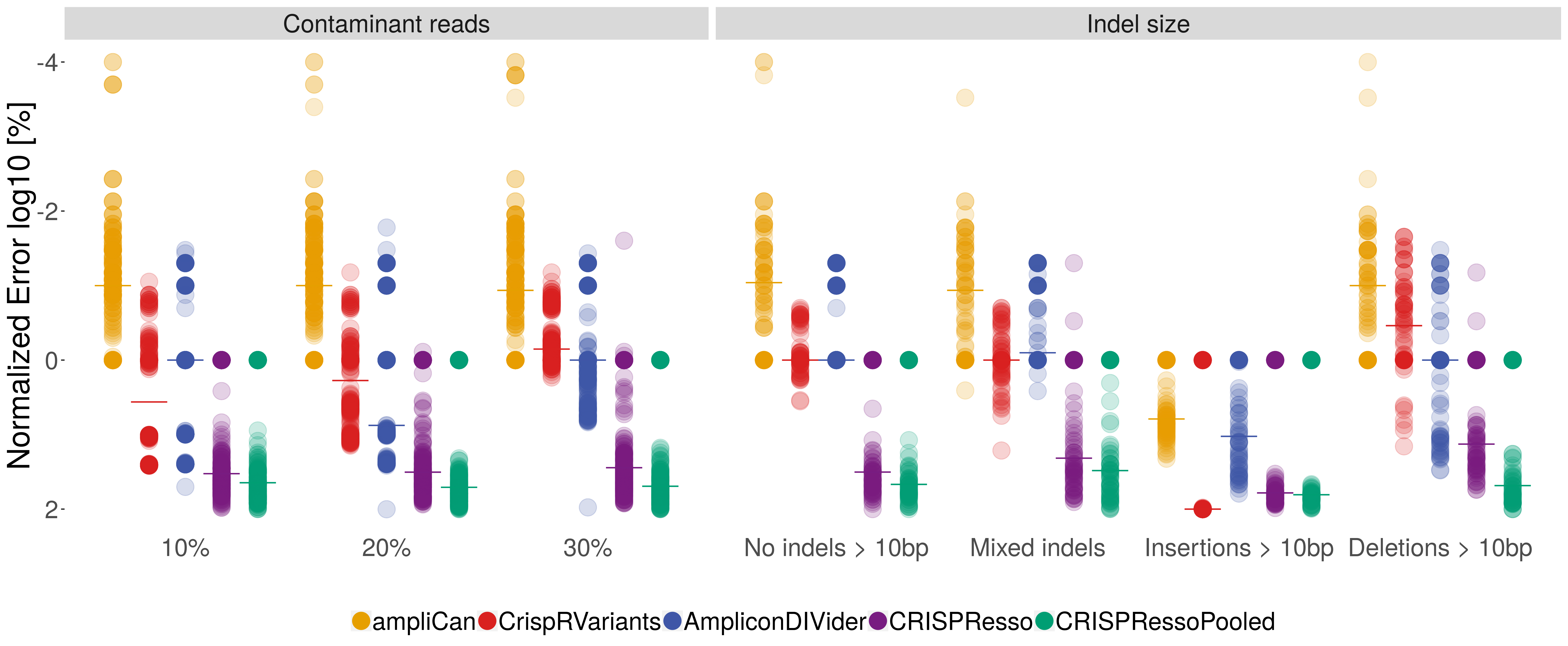

Supplement: Supplemental Material [file supp_gr.244293.118_Supplemental_Code_S1.zip › amplican_manuscript/figures/fig_2_log.png]

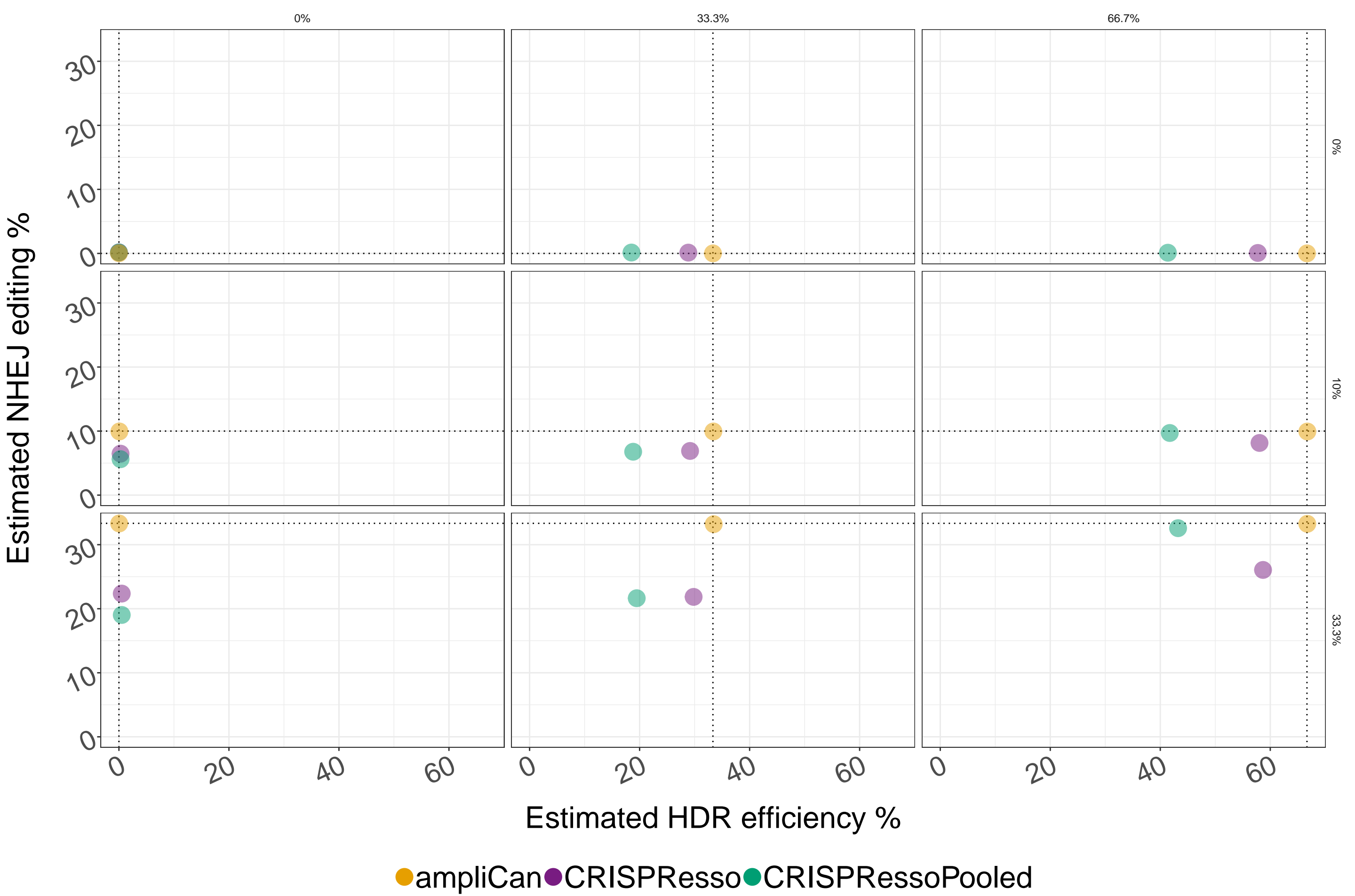

Supplement: Supplemental Material [file supp_gr.244293.118_Supplemental_Code_S1.zip › amplican_manuscript/figures/indel_rate_vs_hdr.pdf]

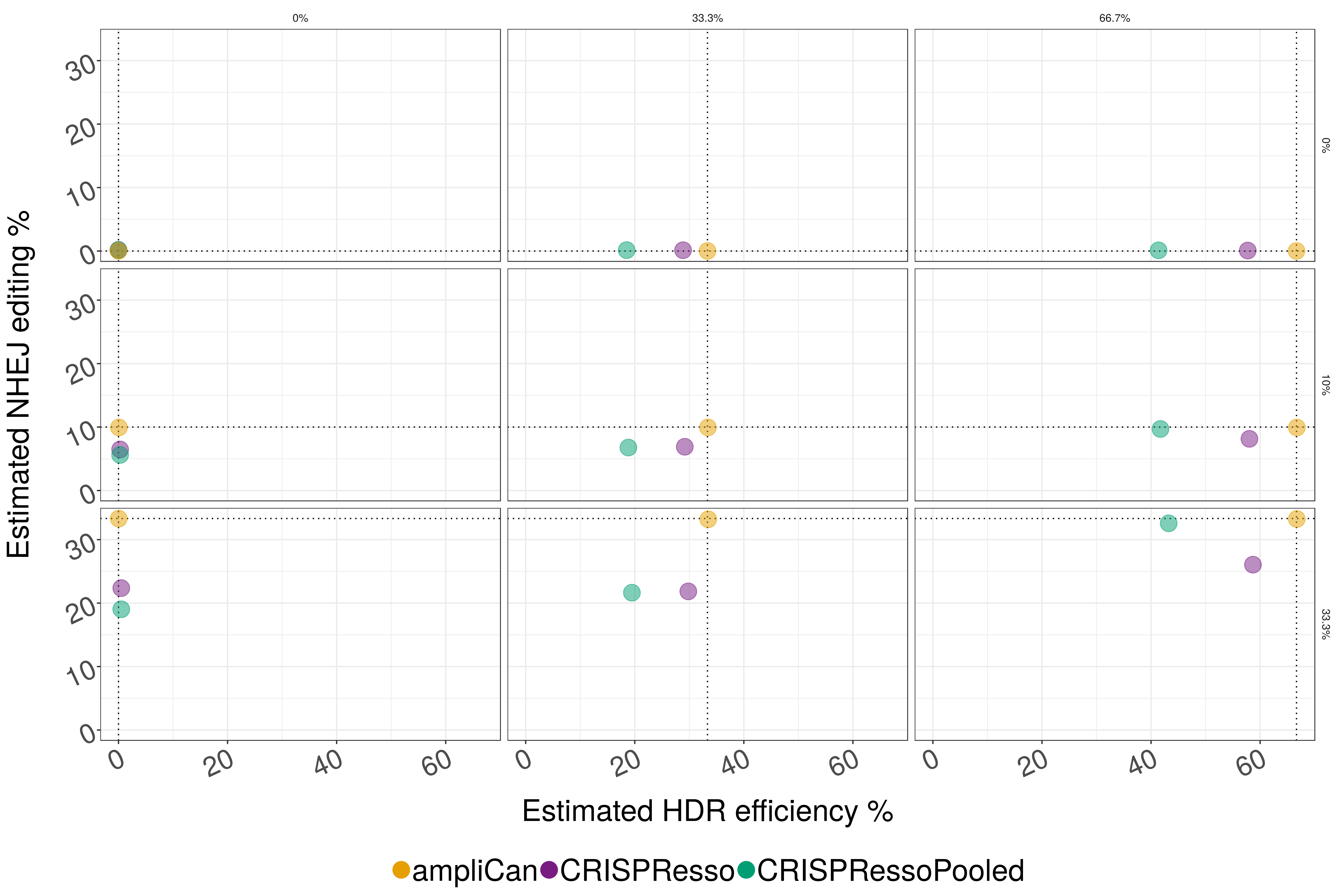

Supplement: Supplemental Material [file supp_gr.244293.118_Supplemental_Code_S1.zip › amplican_manuscript/figures/indel_rate_vs_hdr.png]

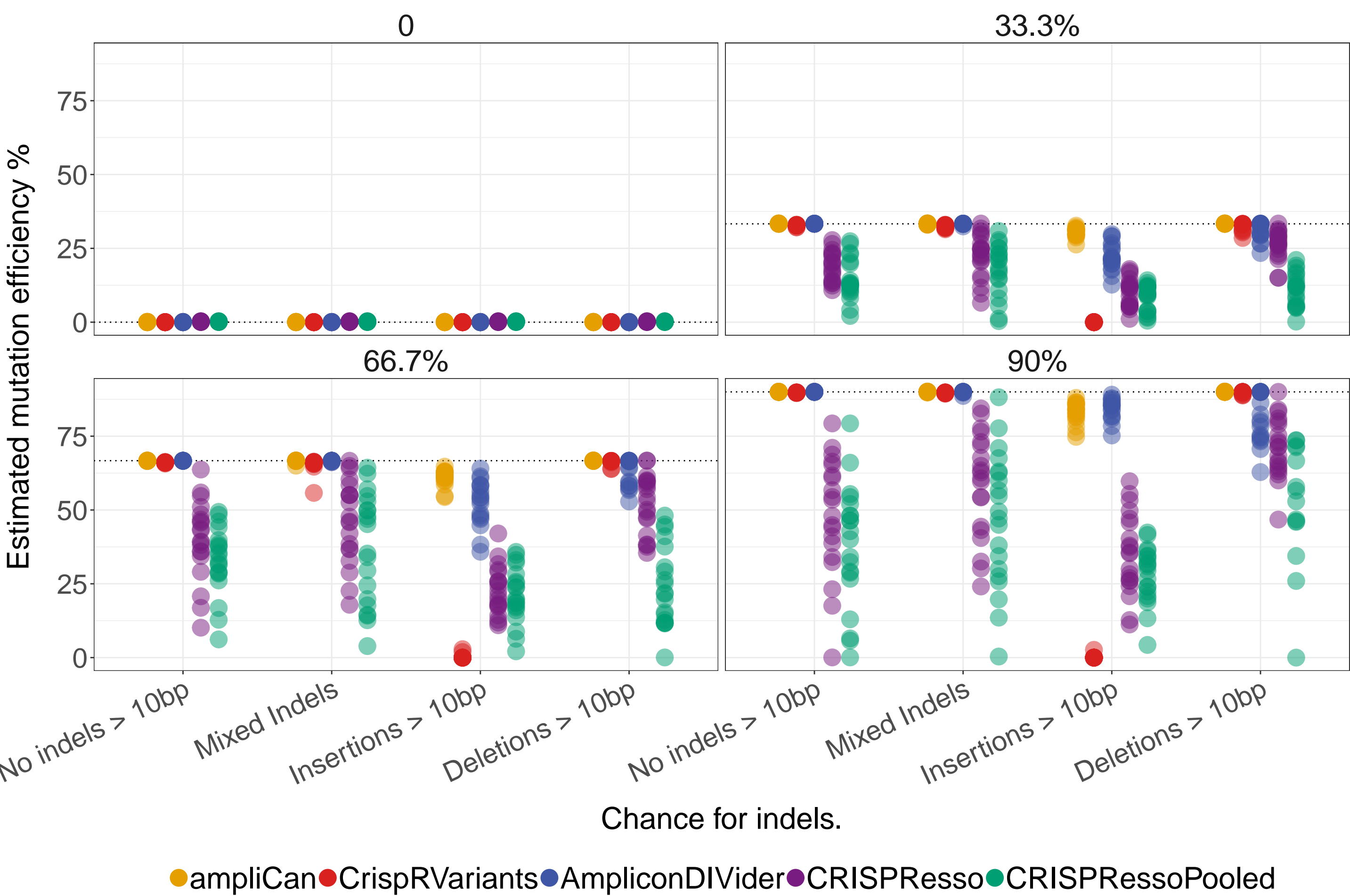

Supplement: Supplemental Material [file supp_gr.244293.118_Supplemental_Code_S1.zip › amplican_manuscript/figures/indel_rate_vs_indel_size.pdf]

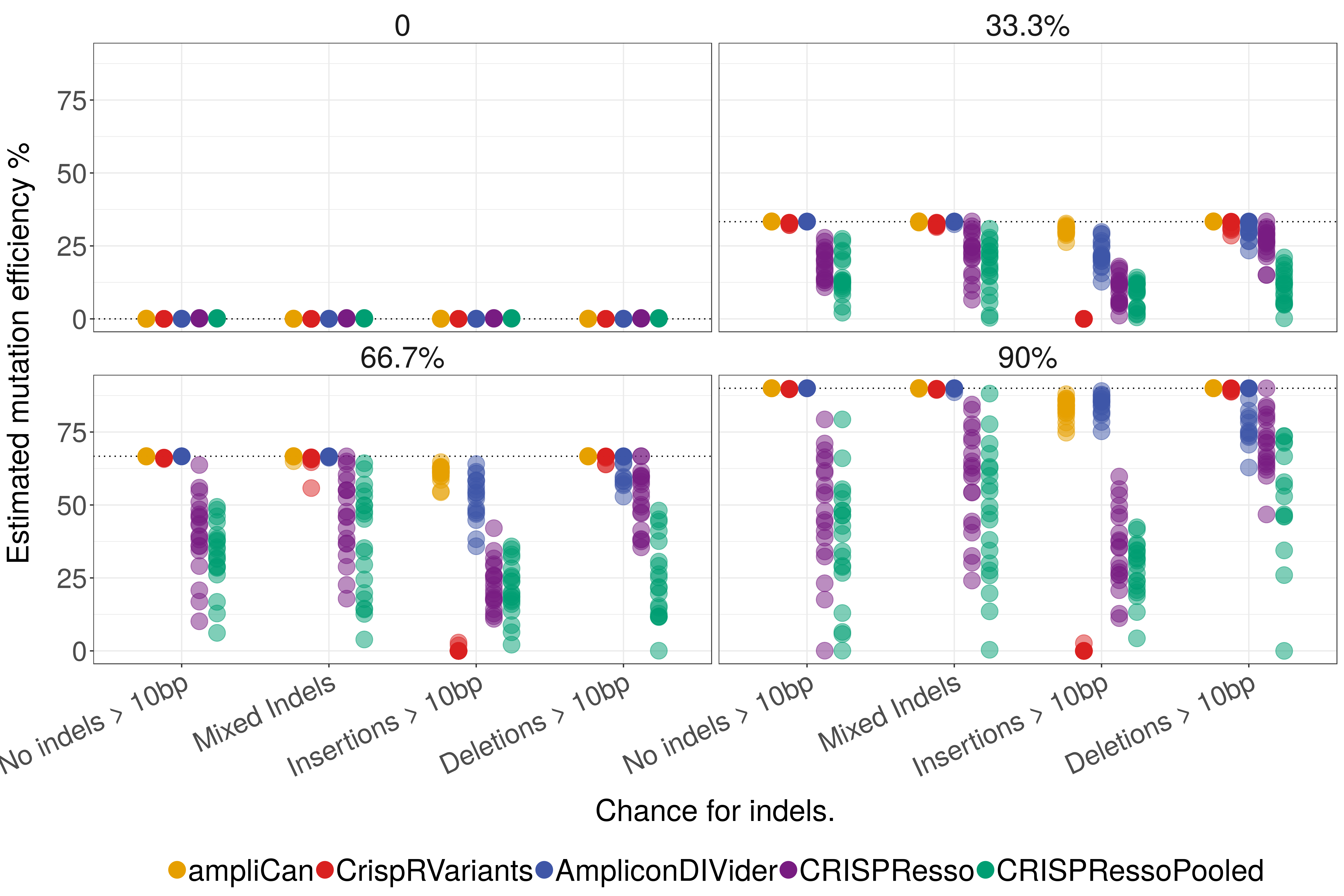

Supplement: Supplemental Material [file supp_gr.244293.118_Supplemental_Code_S1.zip › amplican_manuscript/figures/indel_rate_vs_indel_size.png]

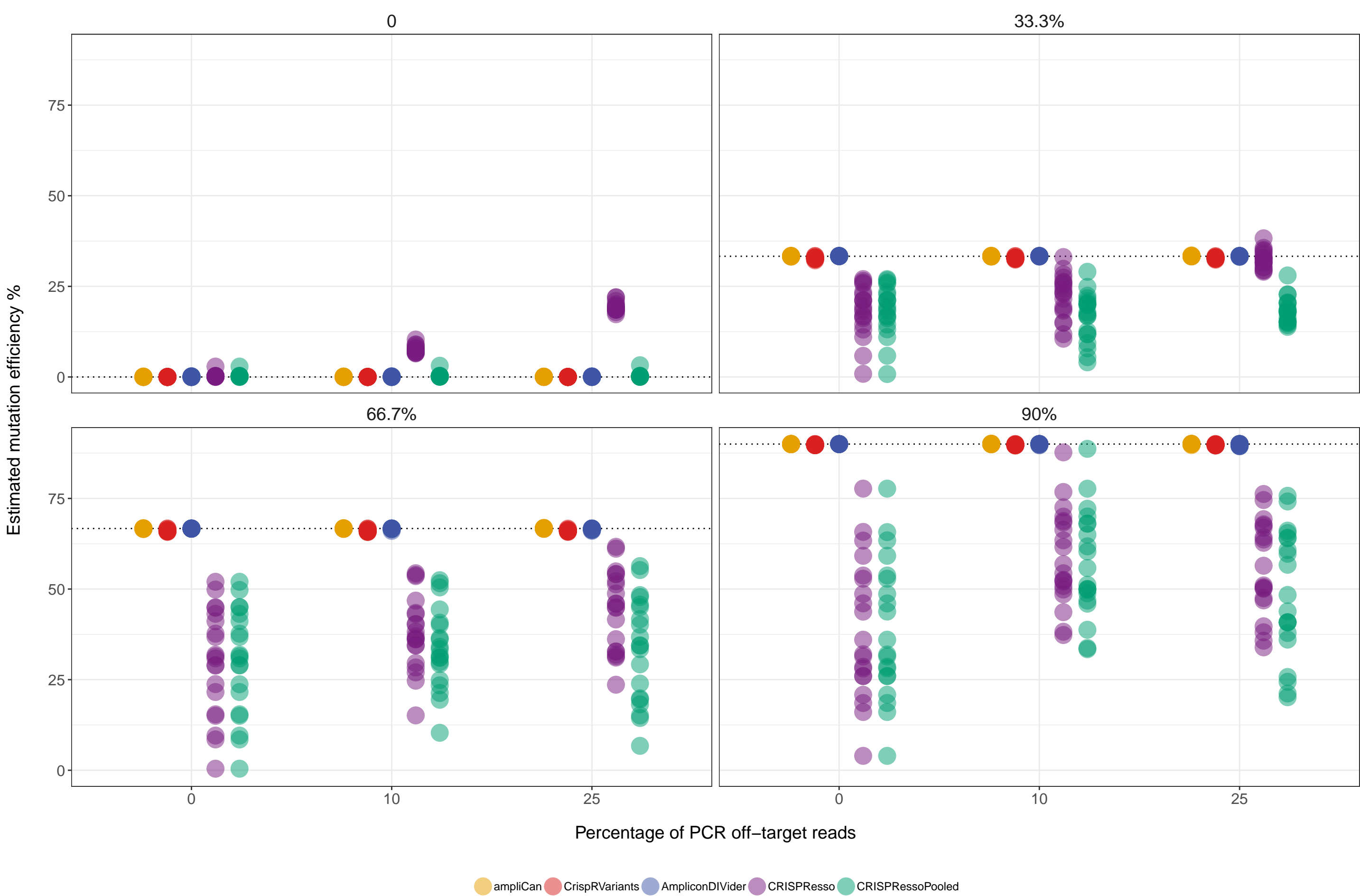

Supplement: Supplemental Material [file supp_gr.244293.118_Supplemental_Code_S1.zip › amplican_manuscript/figures/indel_rate_vs_offtarget.pdf]

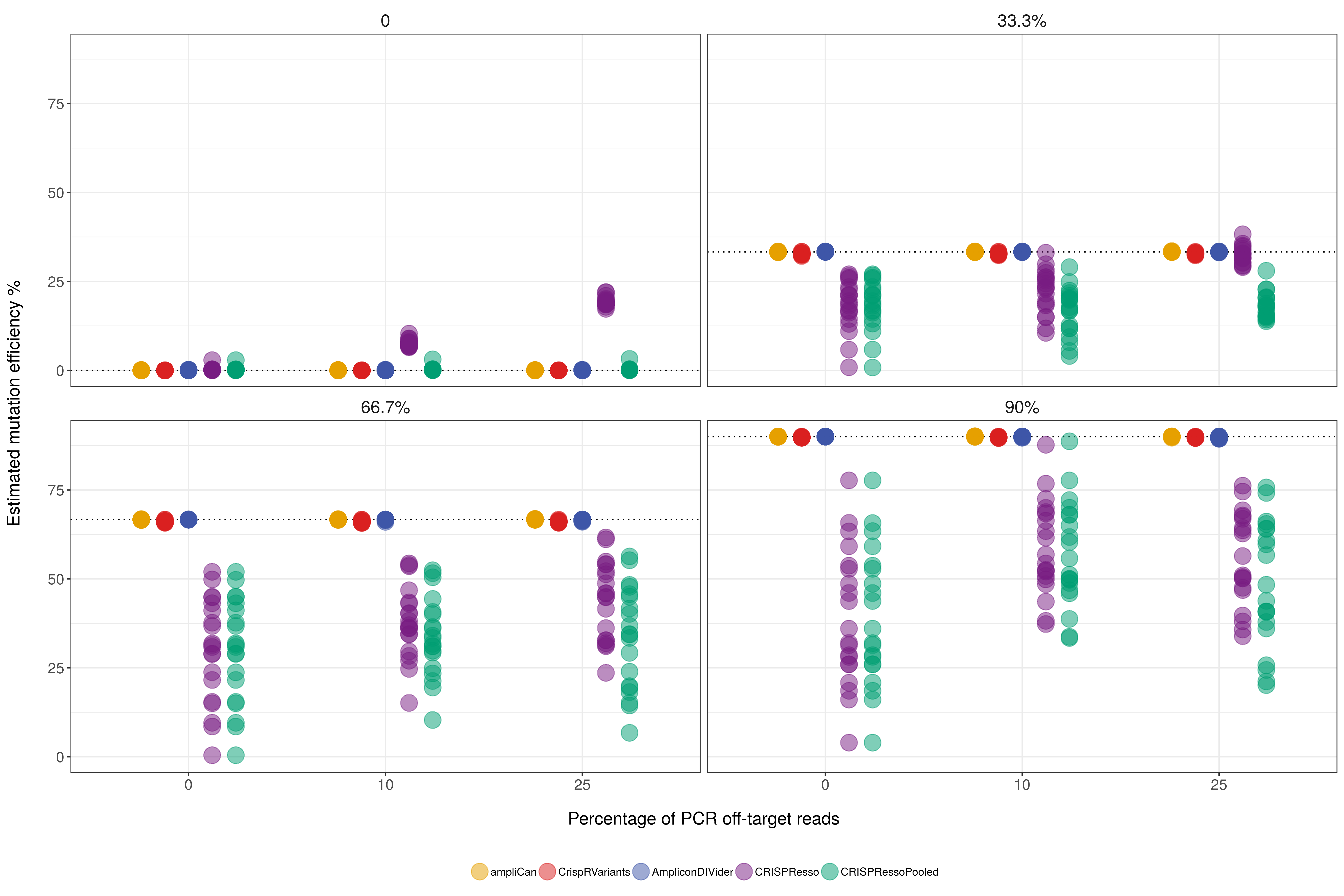

Supplement: Supplemental Material [file supp_gr.244293.118_Supplemental_Code_S1.zip › amplican_manuscript/figures/indel_rate_vs_offtarget.png]

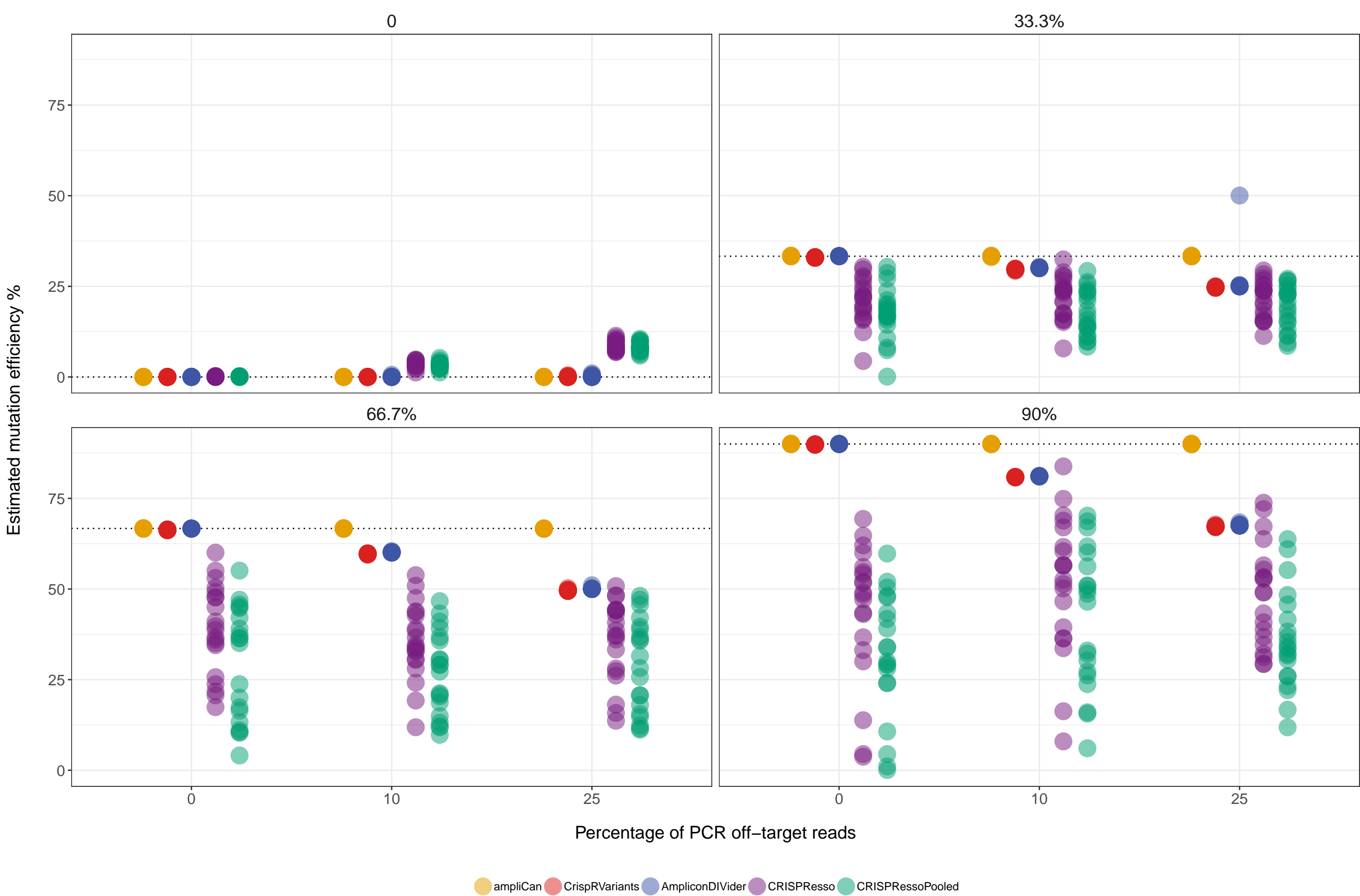

Supplement: Supplemental Material [file supp_gr.244293.118_Supplemental_Code_S1.zip › amplican_manuscript/figures/indel_rate_vs_real_offtargets_0.1.pdf]

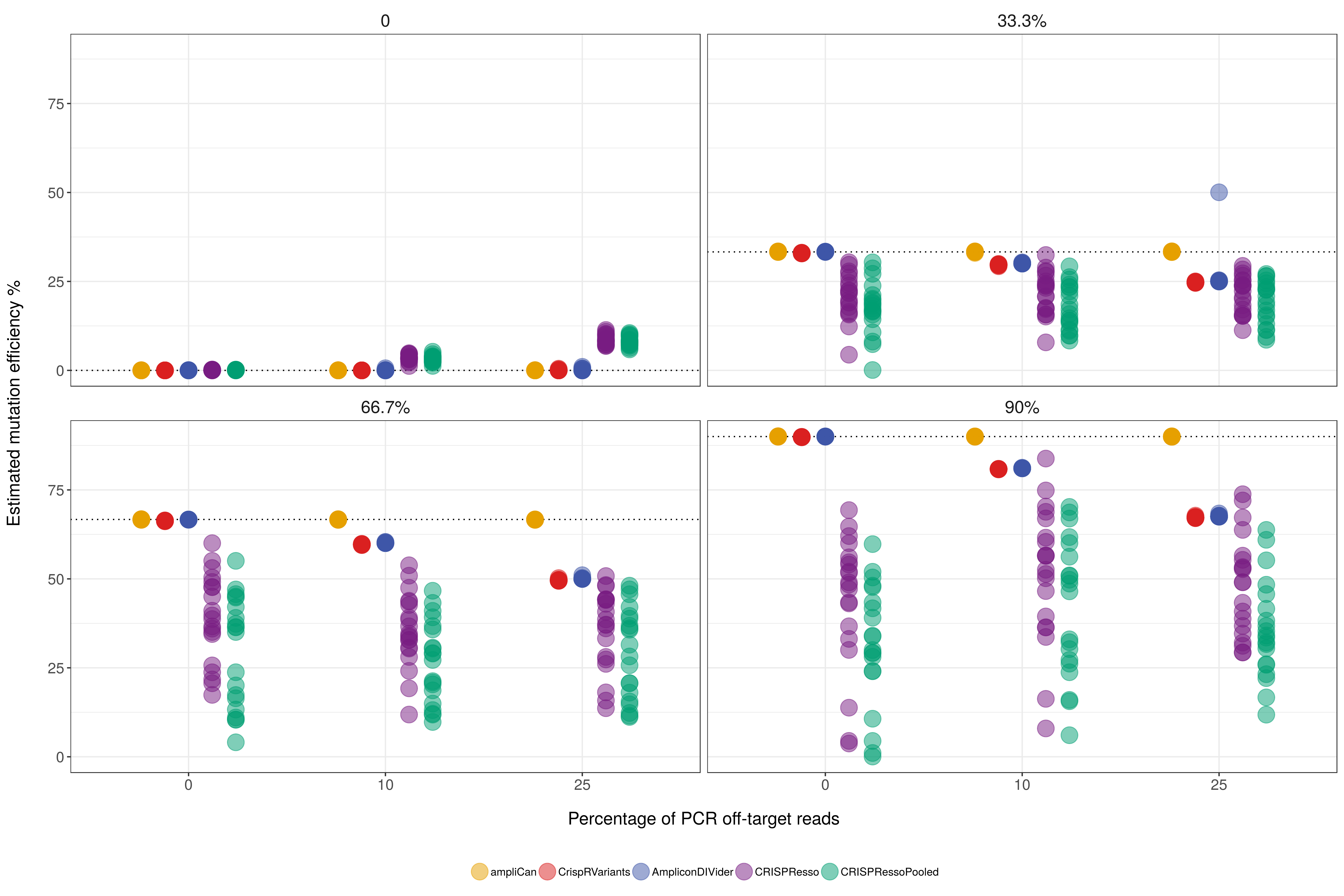

Supplement: Supplemental Material [file supp_gr.244293.118_Supplemental_Code_S1.zip › amplican_manuscript/figures/indel_rate_vs_real_offtargets_0.1.png]

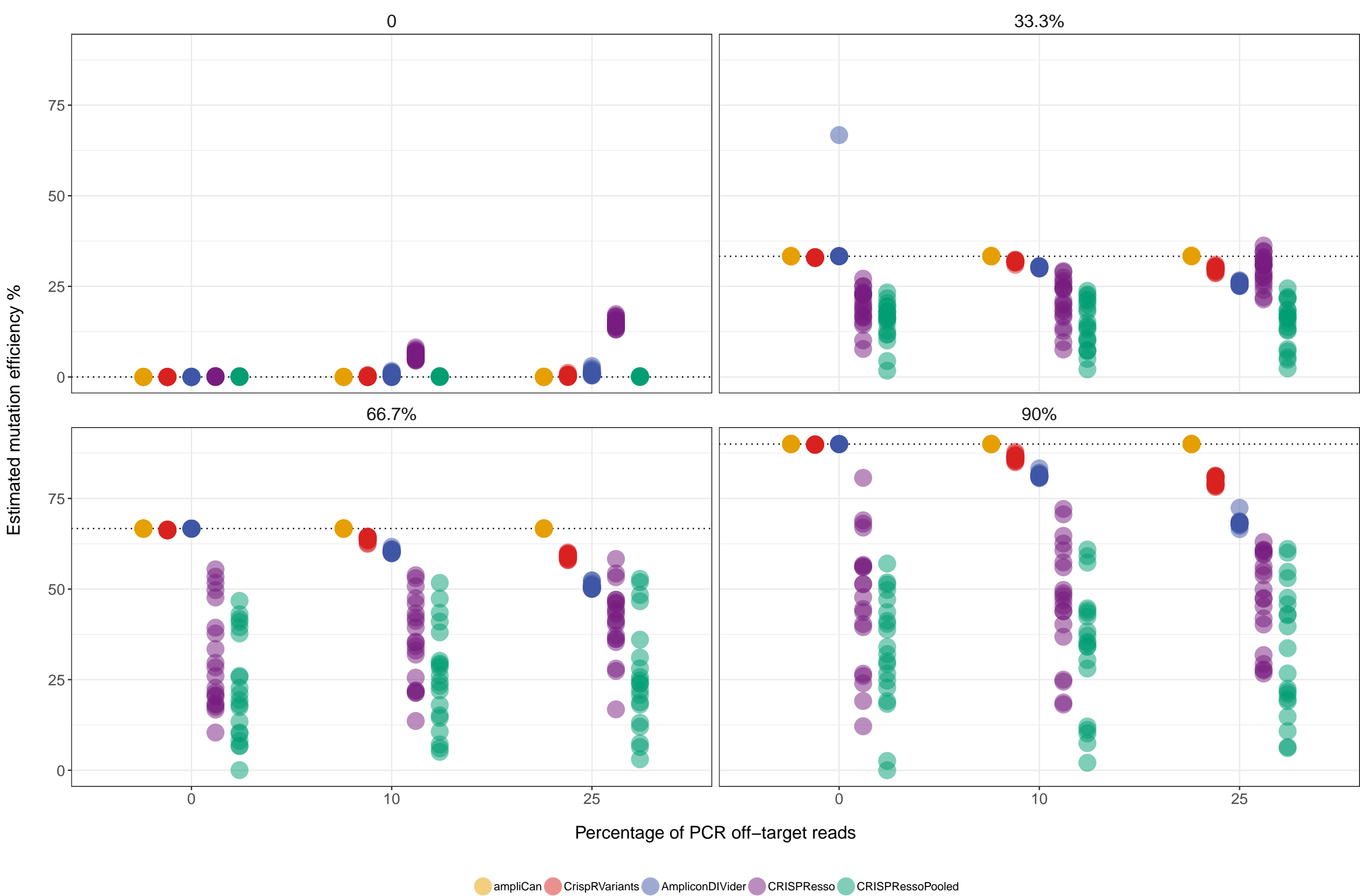

Supplement: Supplemental Material [file supp_gr.244293.118_Supplemental_Code_S1.zip › amplican_manuscript/figures/indel_rate_vs_real_offtargets_0.2.pdf]

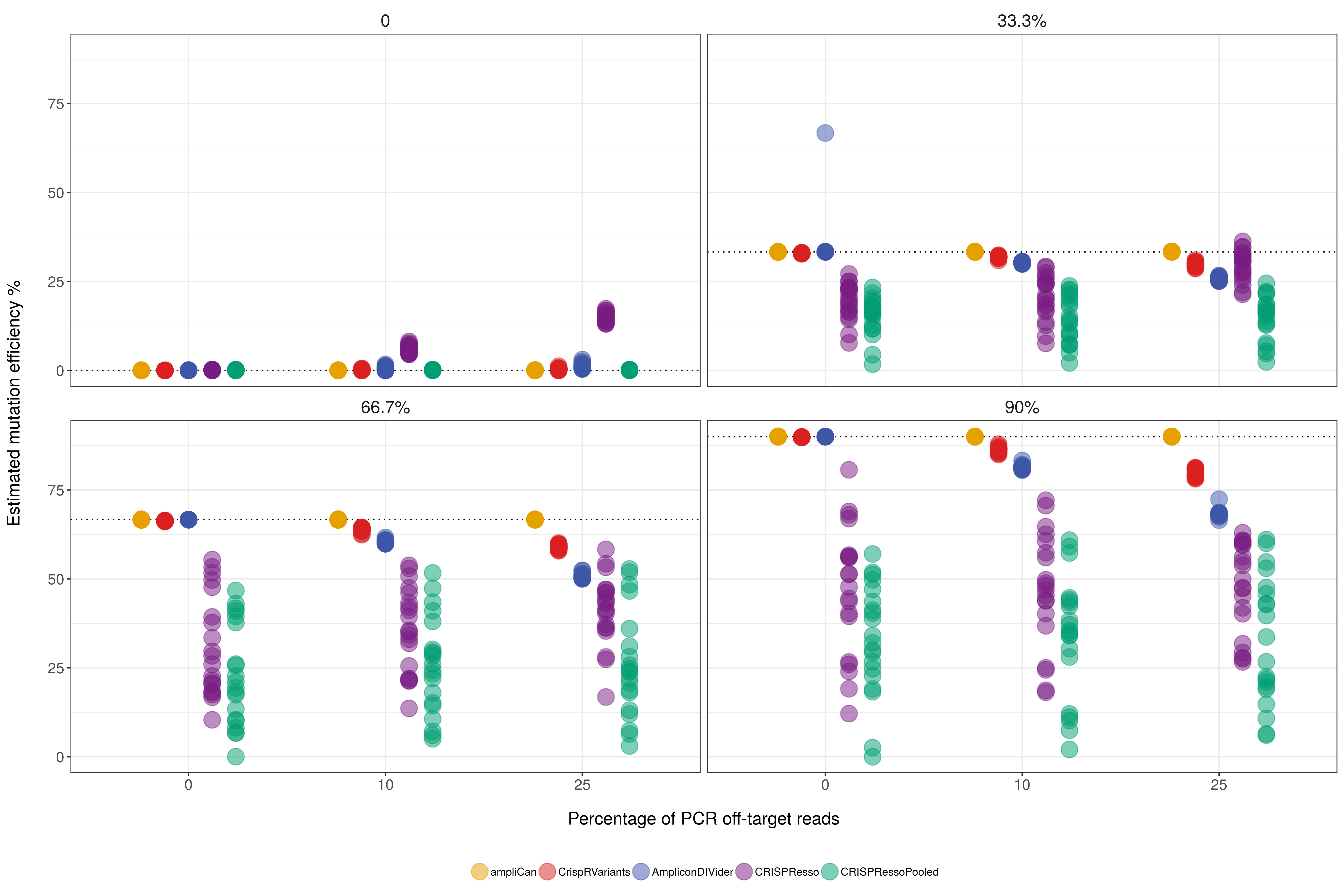

Supplement: Supplemental Material [file supp_gr.244293.118_Supplemental_Code_S1.zip › amplican_manuscript/figures/indel_rate_vs_real_offtargets_0.2.png]

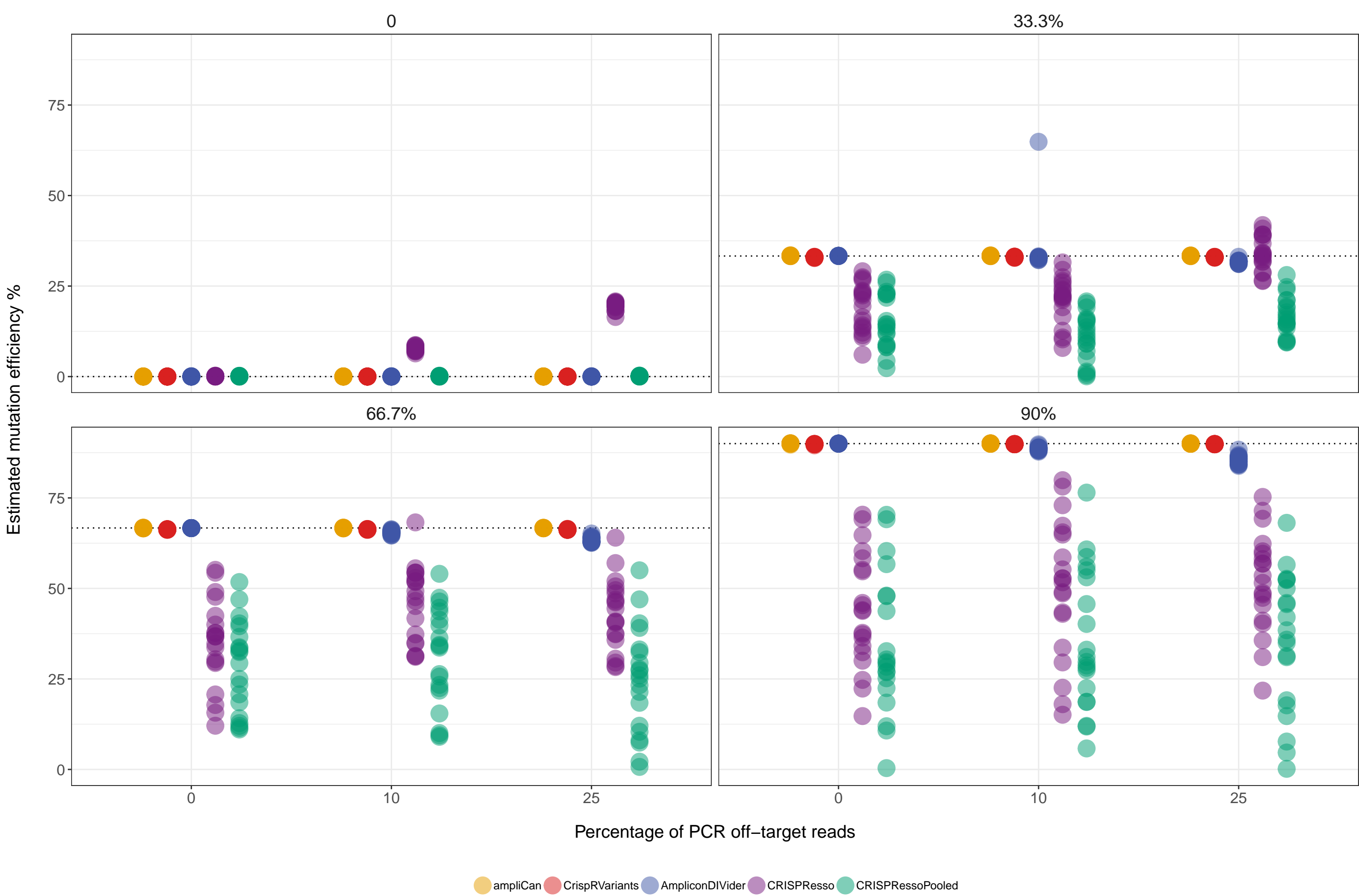

Supplement: Supplemental Material [file supp_gr.244293.118_Supplemental_Code_S1.zip › amplican_manuscript/figures/indel_rate_vs_real_offtargets_0.3.pdf]

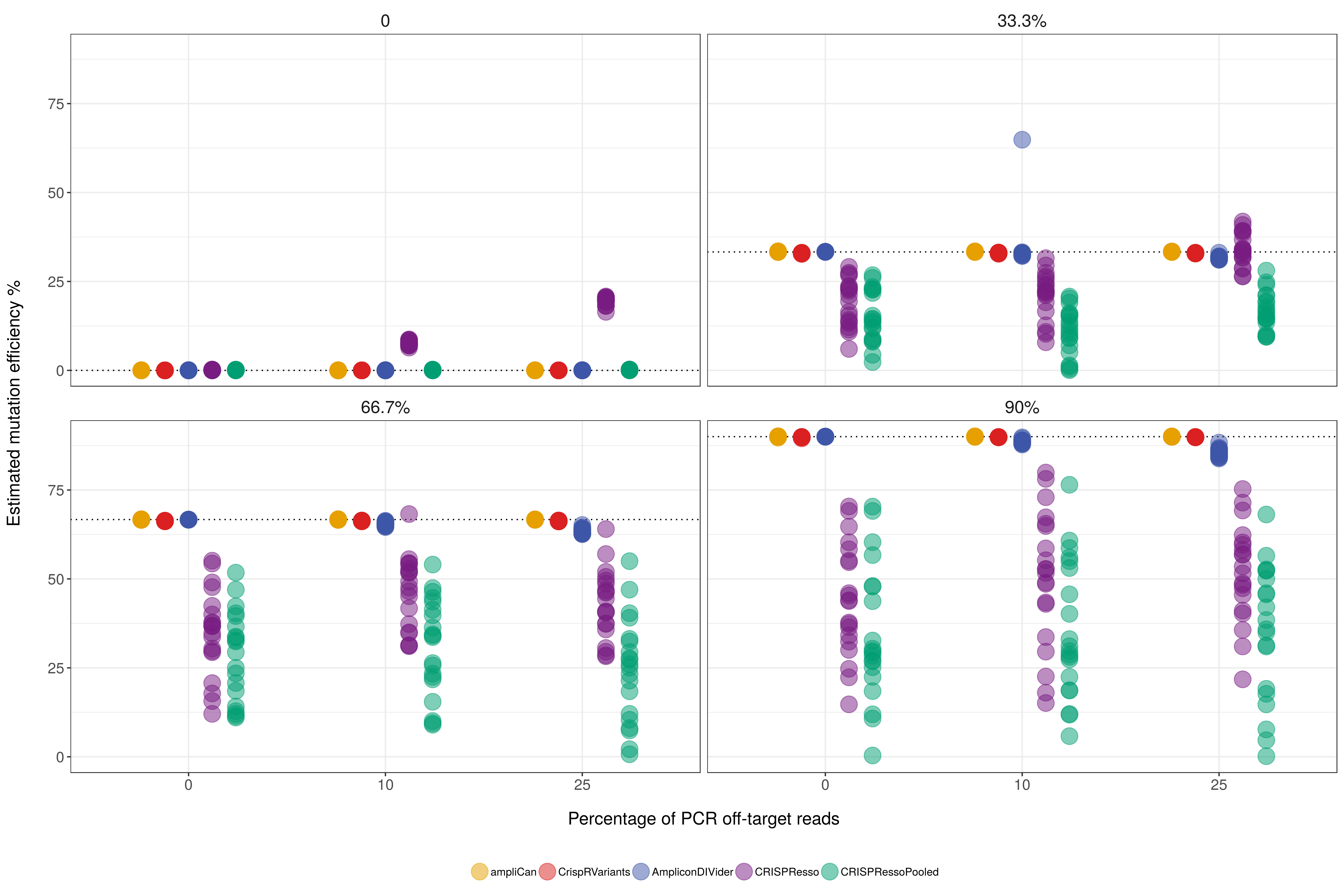

Supplement: Supplemental Material [file supp_gr.244293.118_Supplemental_Code_S1.zip › amplican_manuscript/figures/indel_rate_vs_real_offtargets_0.3.png]

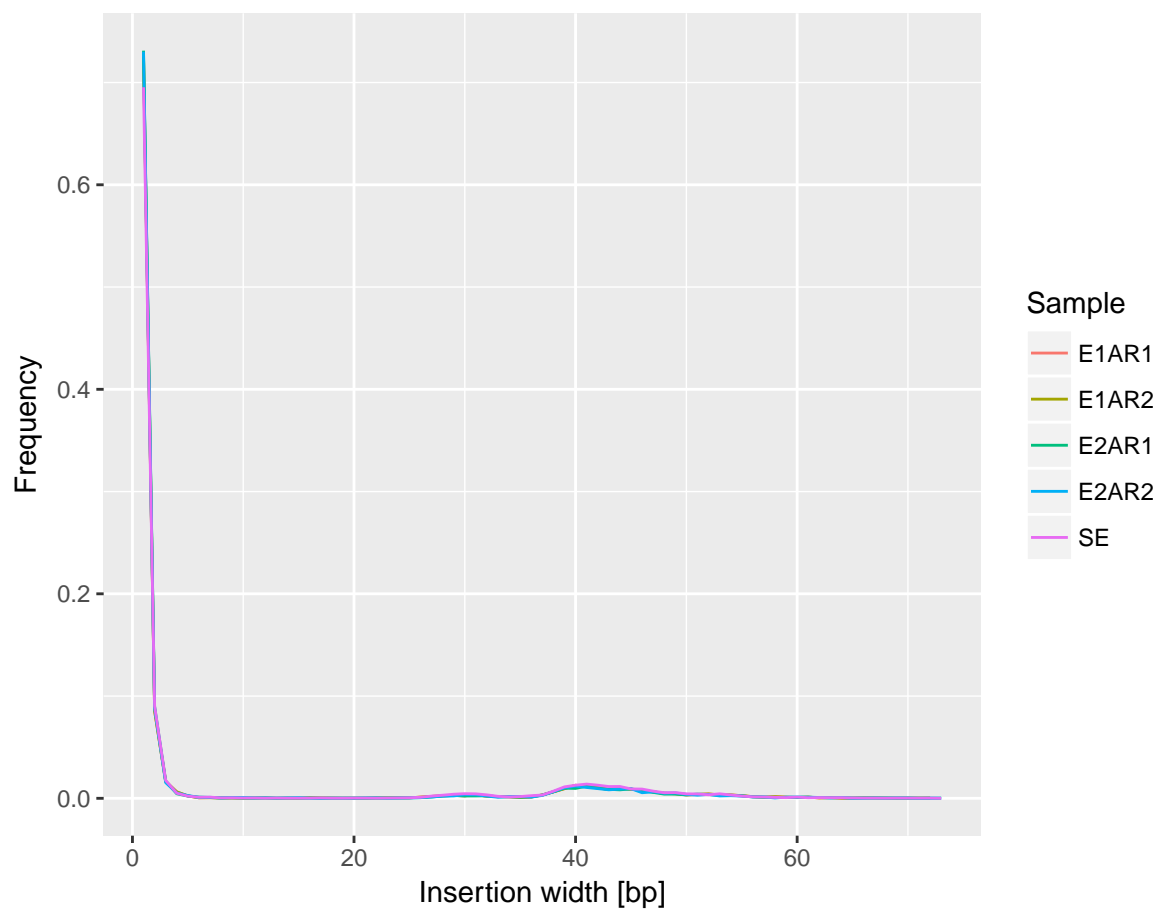

Supplement: Supplemental Material [file supp_gr.244293.118_Supplemental_Code_S1.zip › amplican_manuscript/figures/insertions_chari.pdf]

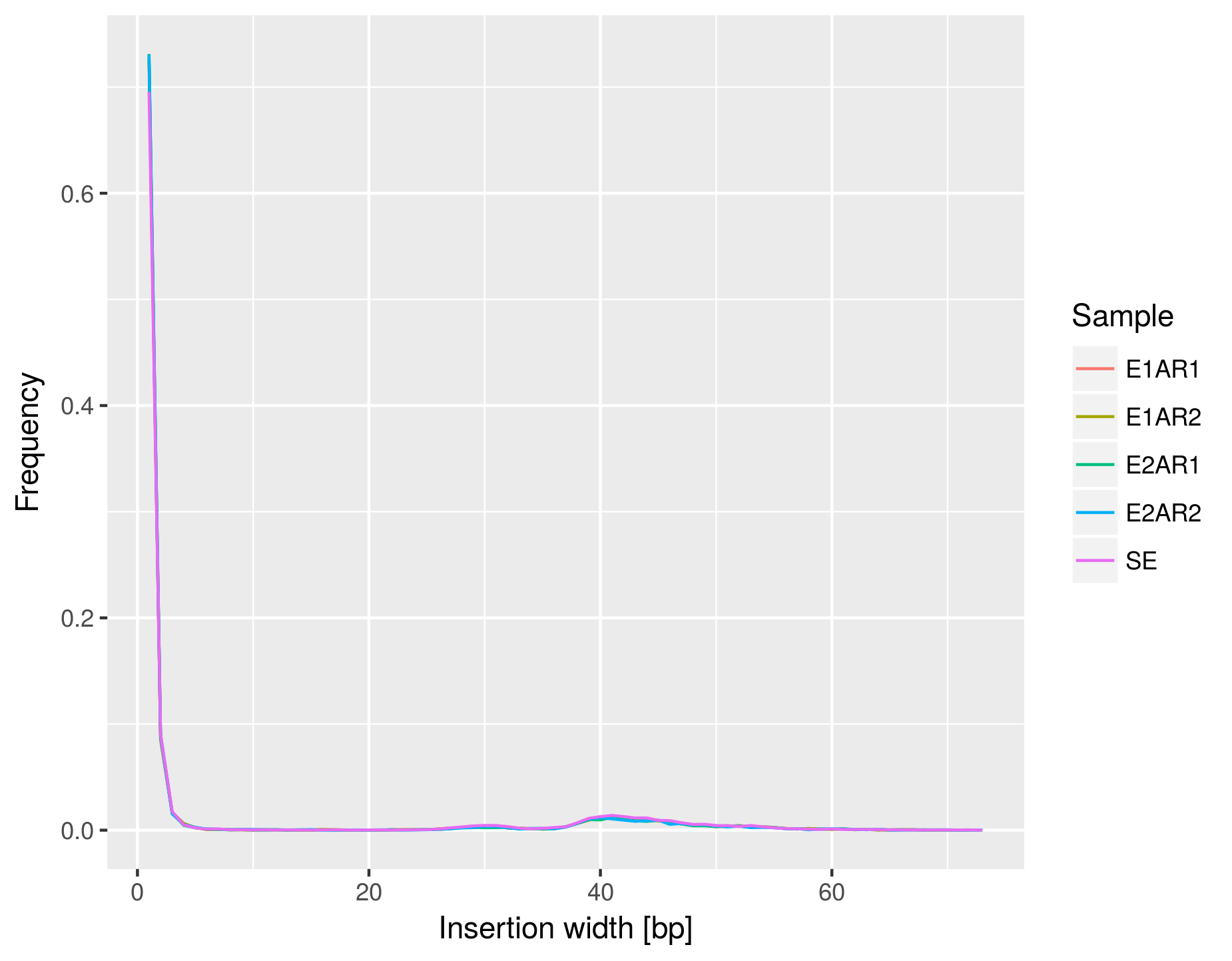

Supplement: Supplemental Material [file supp_gr.244293.118_Supplemental_Code_S1.zip › amplican_manuscript/figures/insertions_chari.png]

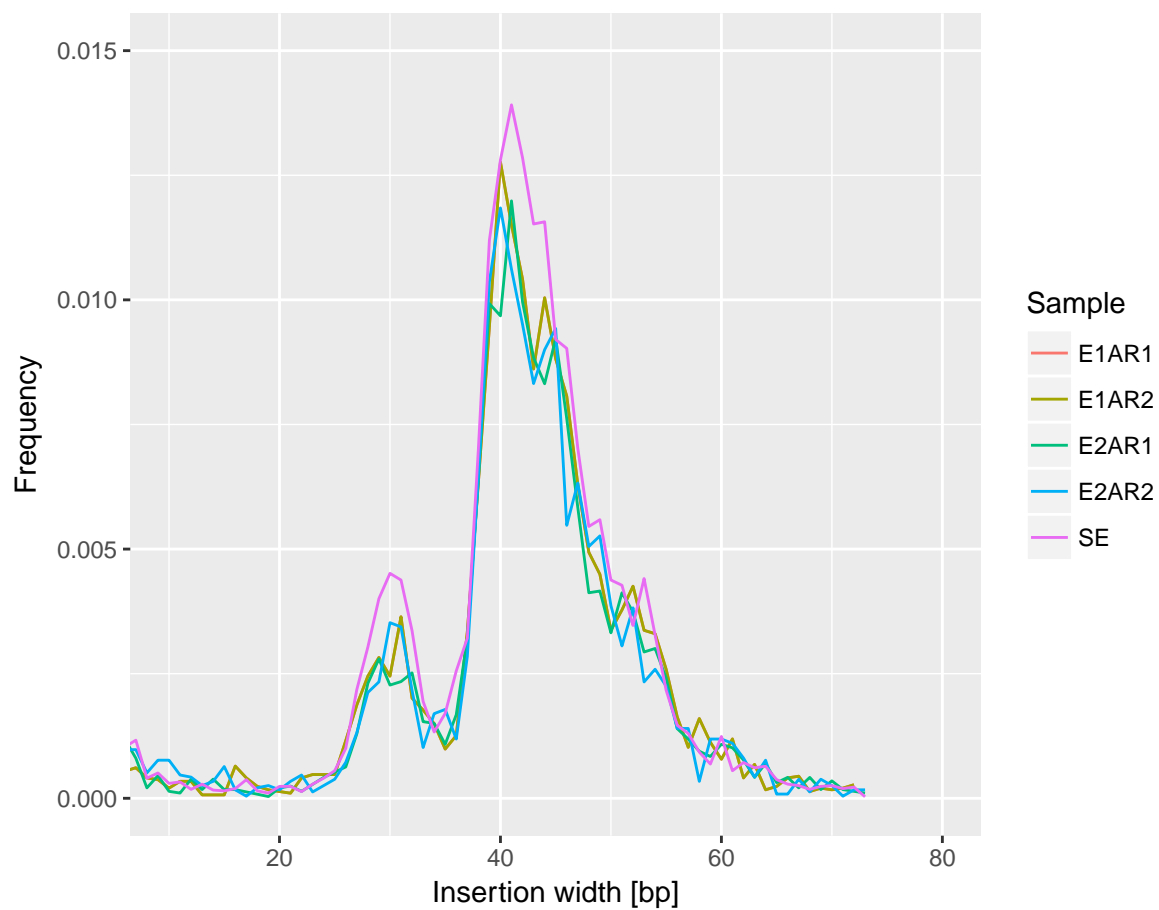

Supplement: Supplemental Material [file supp_gr.244293.118_Supplemental_Code_S1.zip › amplican_manuscript/figures/insertions_chari_zoomed.pdf]

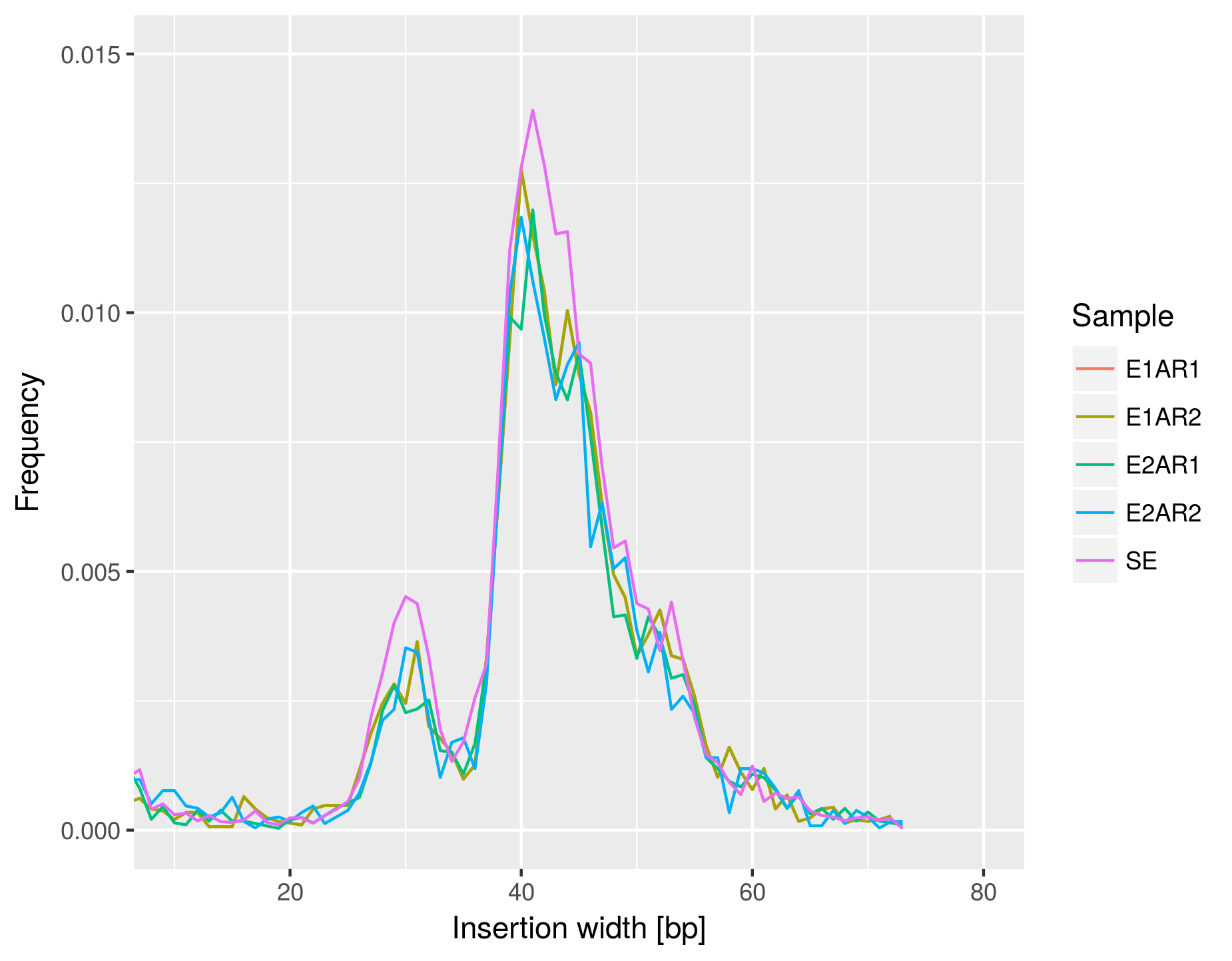

Supplement: Supplemental Material [file supp_gr.244293.118_Supplemental_Code_S1.zip › amplican_manuscript/figures/insertions_chari_zoomed.png]

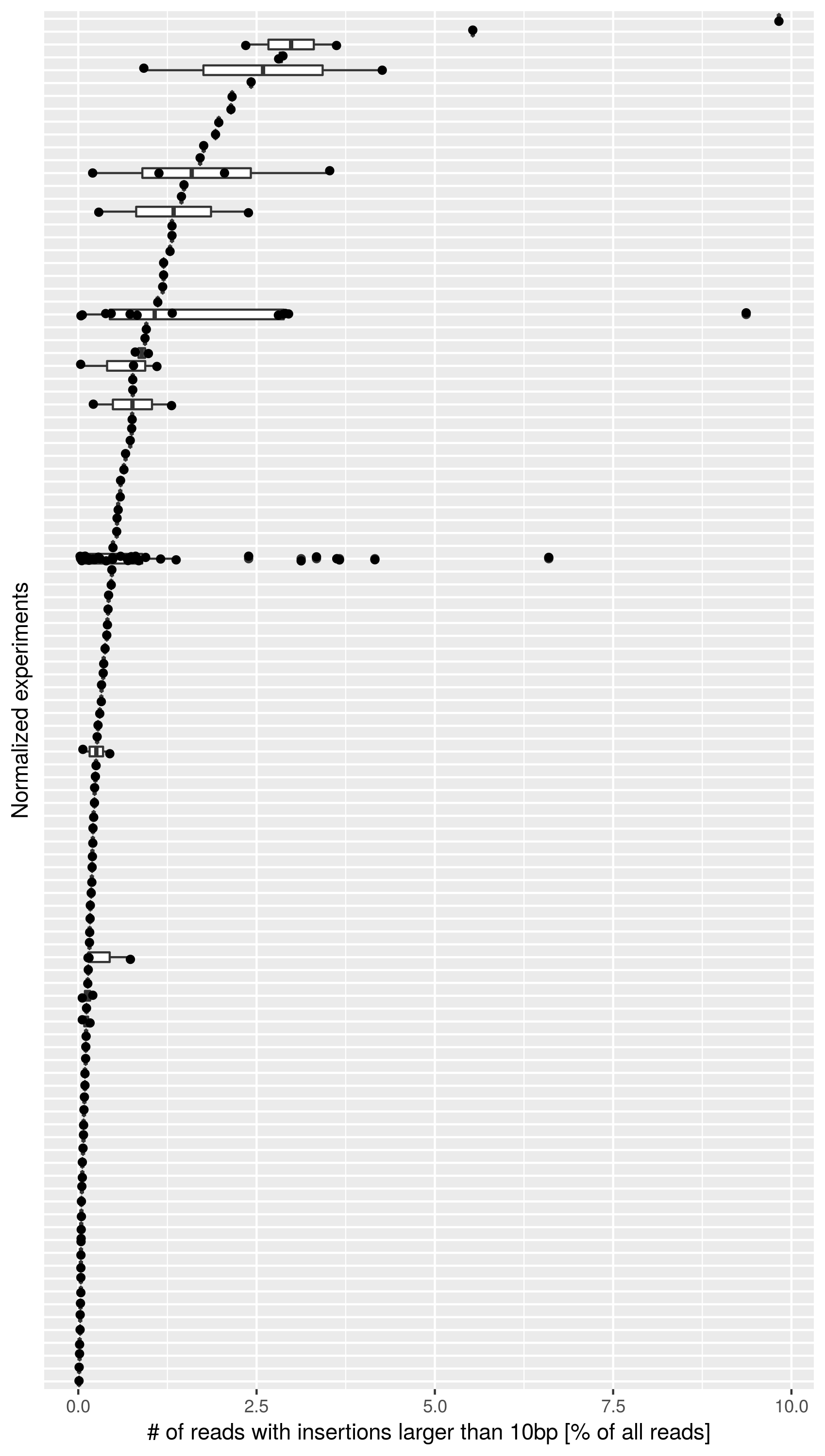

Supplement: Supplemental Material [file supp_gr.244293.118_Supplemental_Code_S1.zip › amplican_manuscript/figures/large_insertion_rate.png]

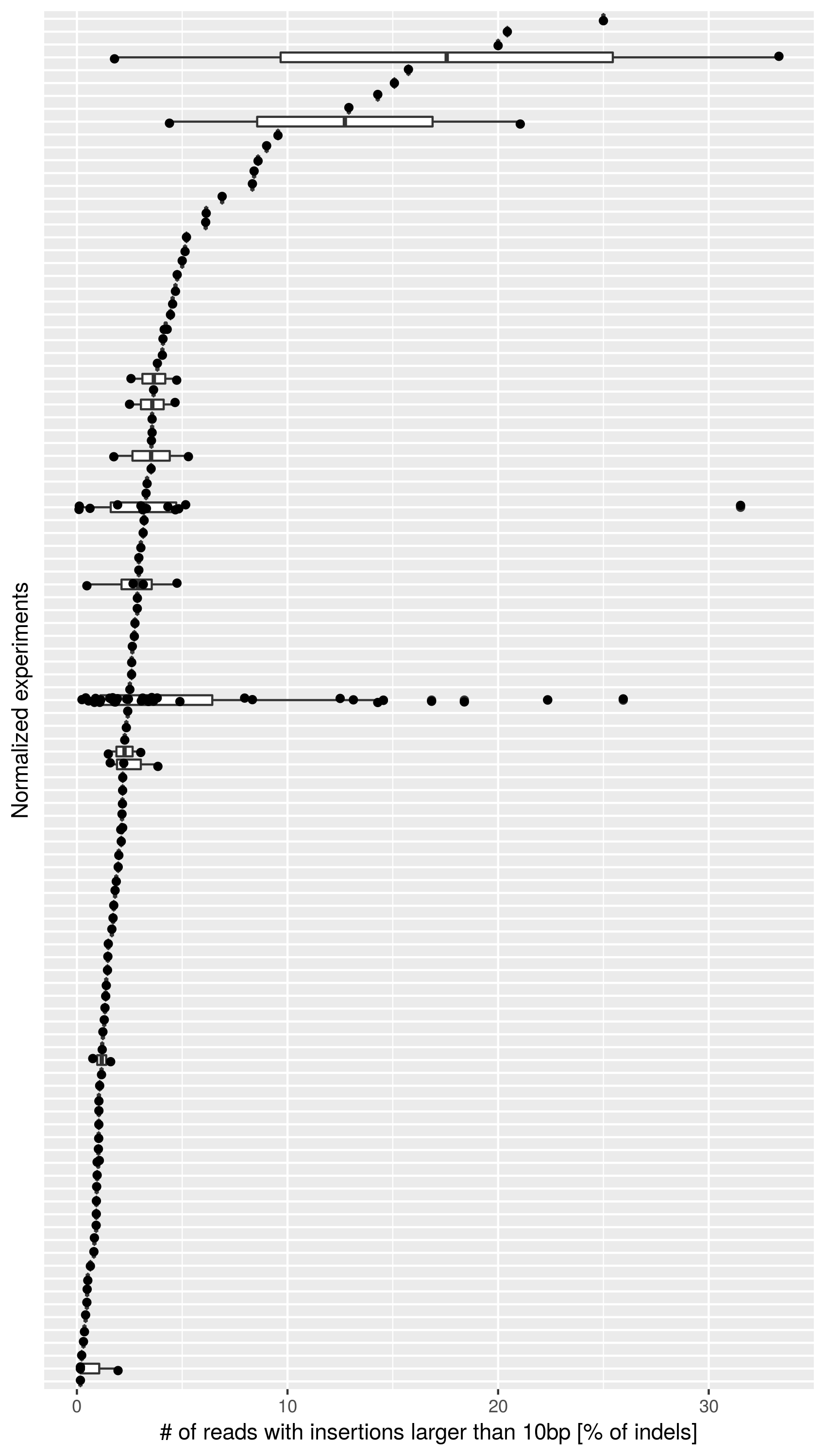

Supplement: Supplemental Material [file supp_gr.244293.118_Supplemental_Code_S1.zip › amplican_manuscript/figures/large_insertion_rate_normalized.png]

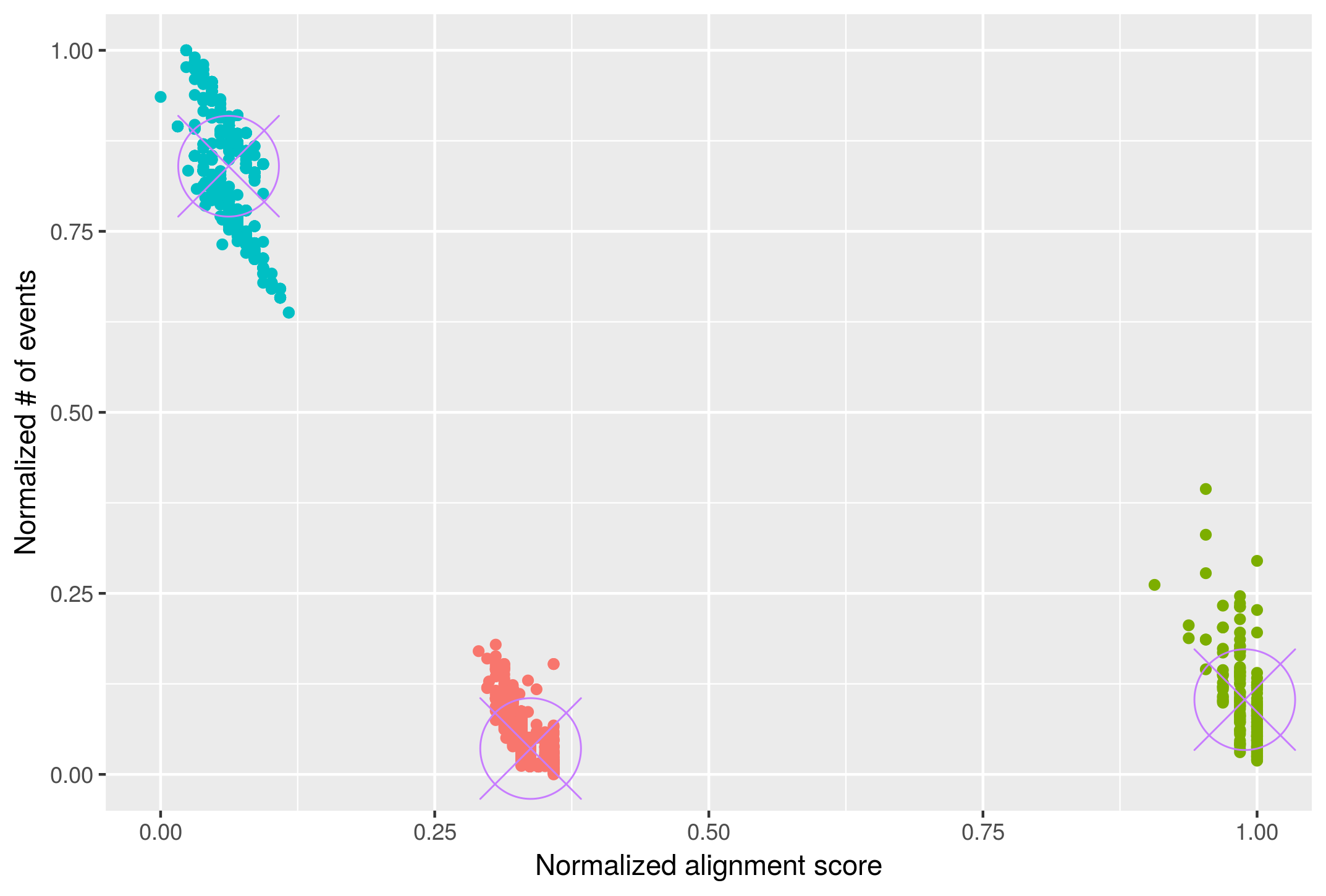

Supplement: Supplemental Material [file supp_gr.244293.118_Supplemental_Code_S1.zip › amplican_manuscript/figures/low_quality_reads_example.png]

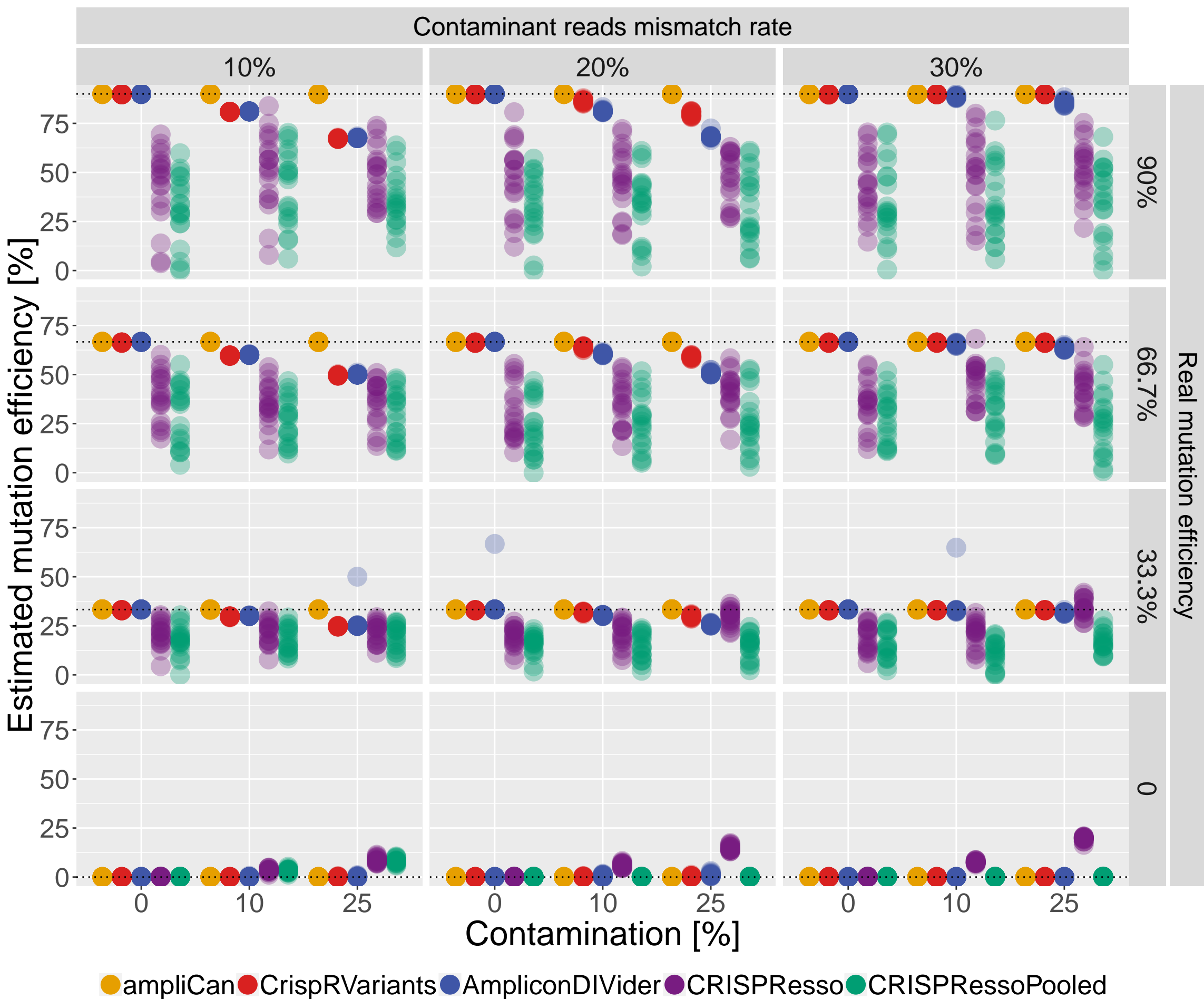

Supplement: Supplemental Material [file supp_gr.244293.118_Supplemental_Code_S1.zip › amplican_manuscript/figures/mutation_efficiency_mutation rate.pdf]

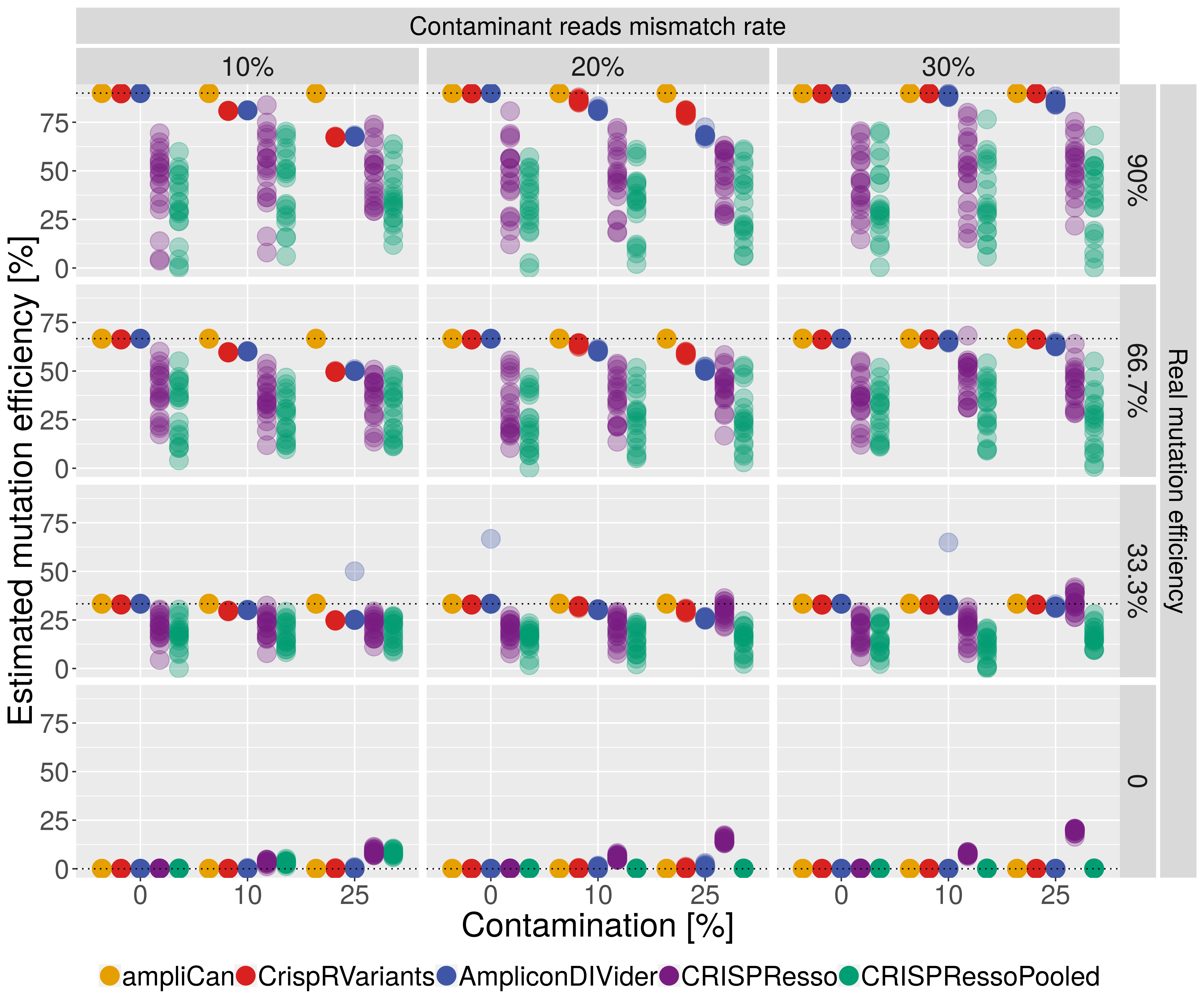

Supplement: Supplemental Material [file supp_gr.244293.118_Supplemental_Code_S1.zip › amplican_manuscript/figures/mutation_efficiency_mutation rate.png]

Data ● Raw ● Normalized

Experiment

0 25 50 75 100  
Estimated indel rate [%]

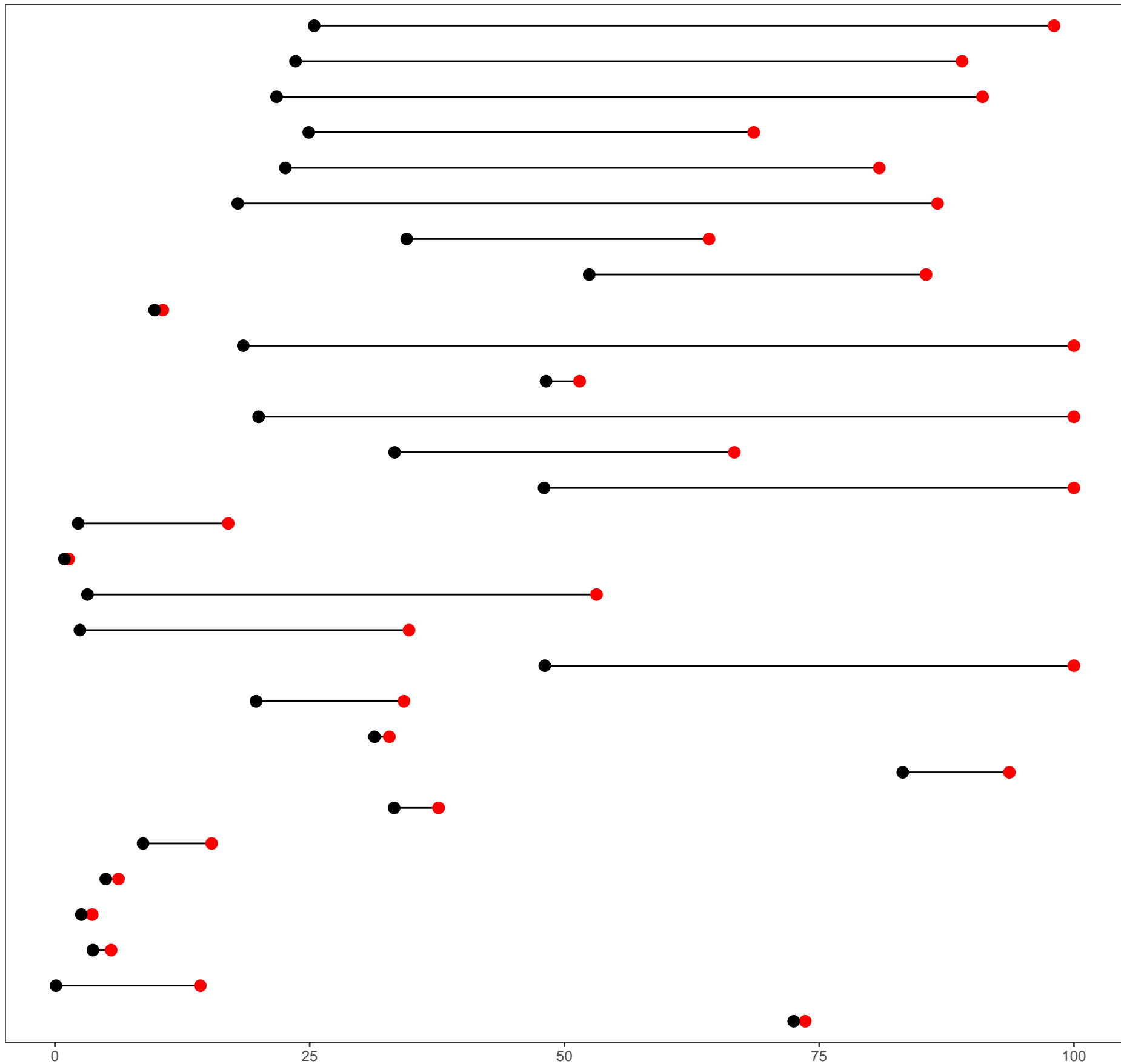

Supplement: Supplemental Material [file supp_gr.244293.118_Supplemental_Code_S1.zip › amplican_manuscript/figures/normalized_change.pdf]

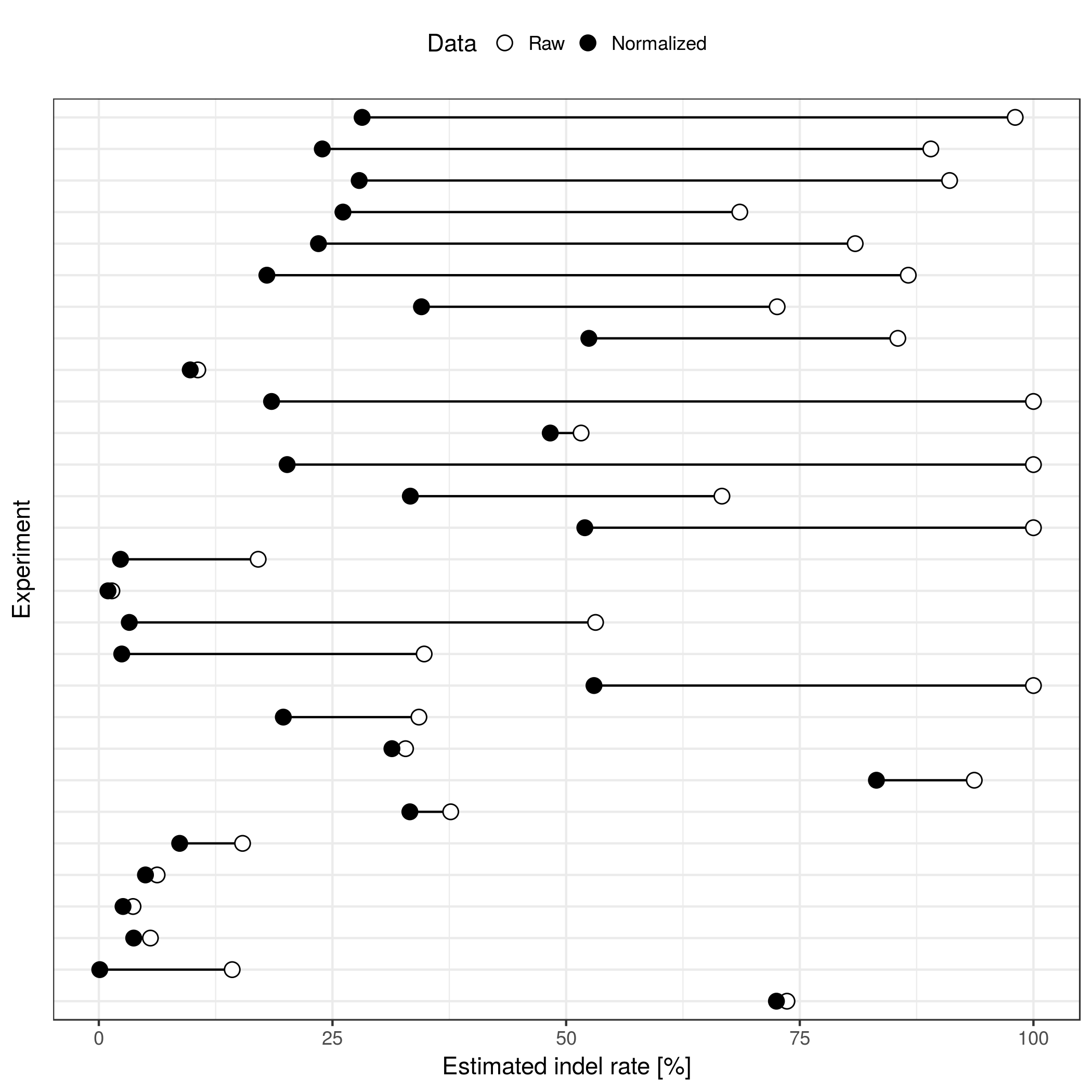

Supplement: Supplemental Material [file supp_gr.244293.118_Supplemental_Code_S1.zip › amplican_manuscript/figures/normalized_change.png]

Estimated mutation efficiency – ampliCan not normalized estimates [%]

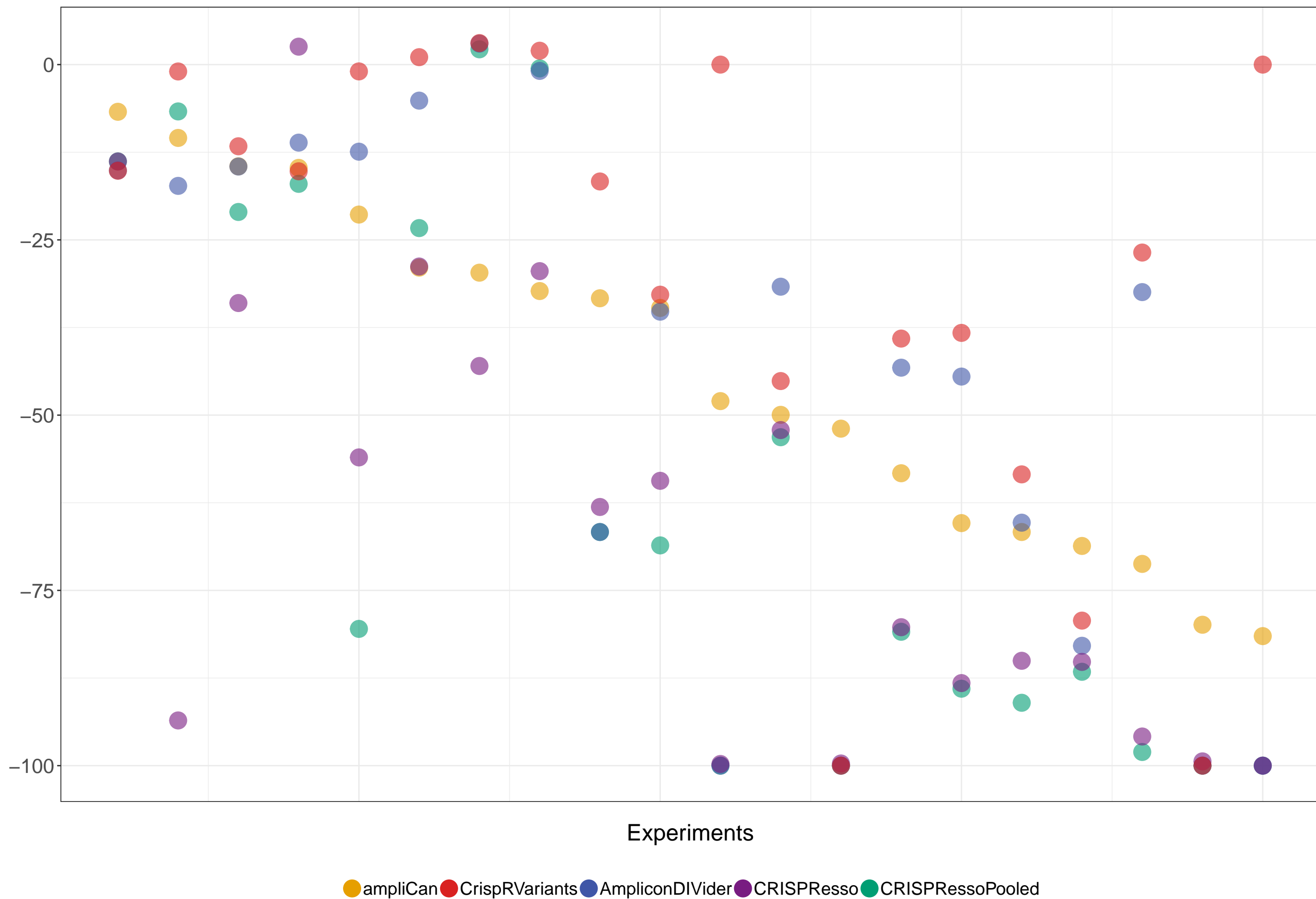

Supplement: Supplemental Material [file supp_gr.244293.118_Supplemental_Code_S1.zip › amplican_manuscript/figures/not_norm_dist_real_datasets.pdf]

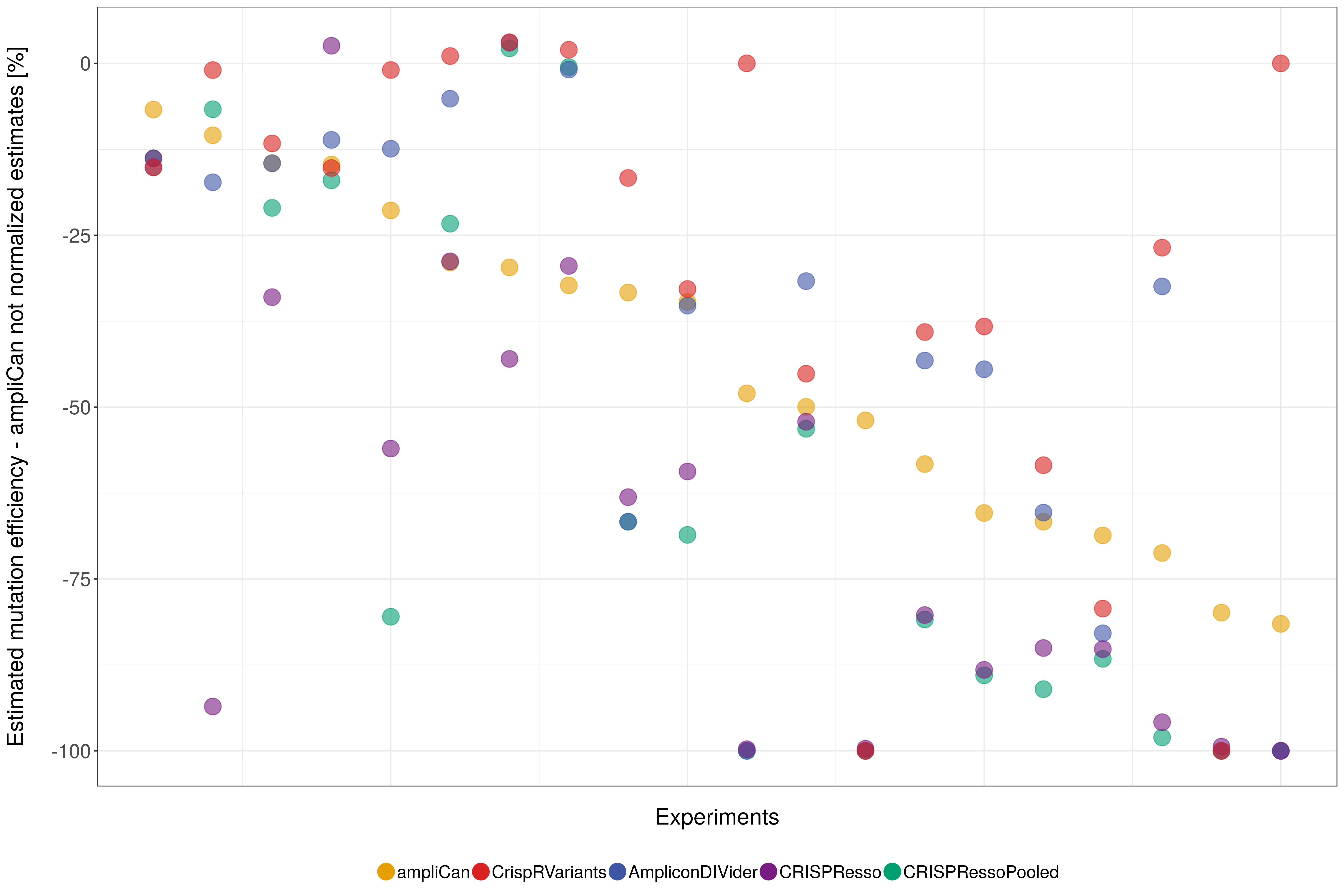

Supplement: Supplemental Material [file supp_gr.244293.118_Supplemental_Code_S1.zip › amplican_manuscript/figures/not_norm_dist_real_datasets.png]

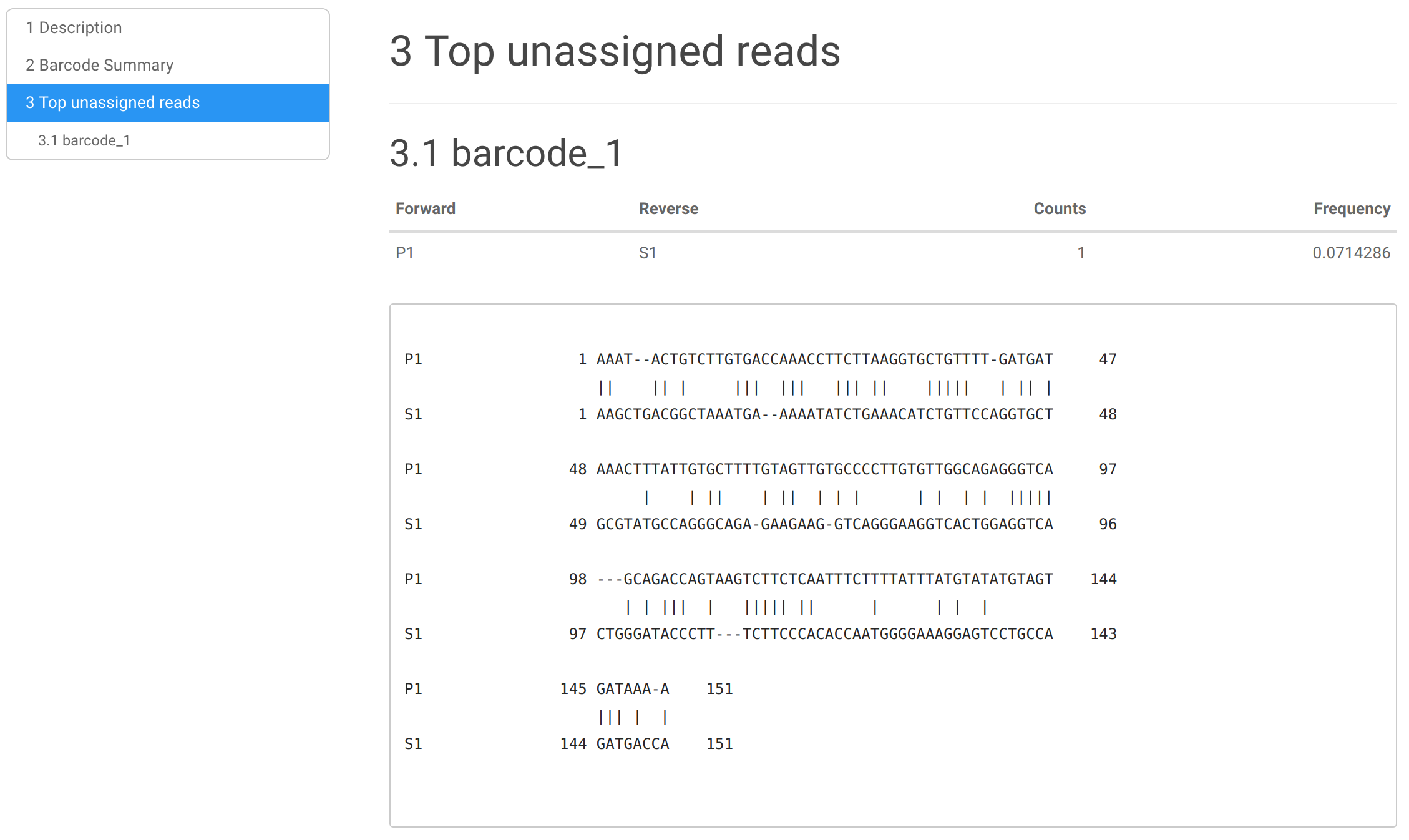

Supplement: Supplemental Material [file supp_gr.244293.118_Supplemental_Code_S1.zip › amplican_manuscript/figures/screenshot_of_example_unassigned_reads.png]

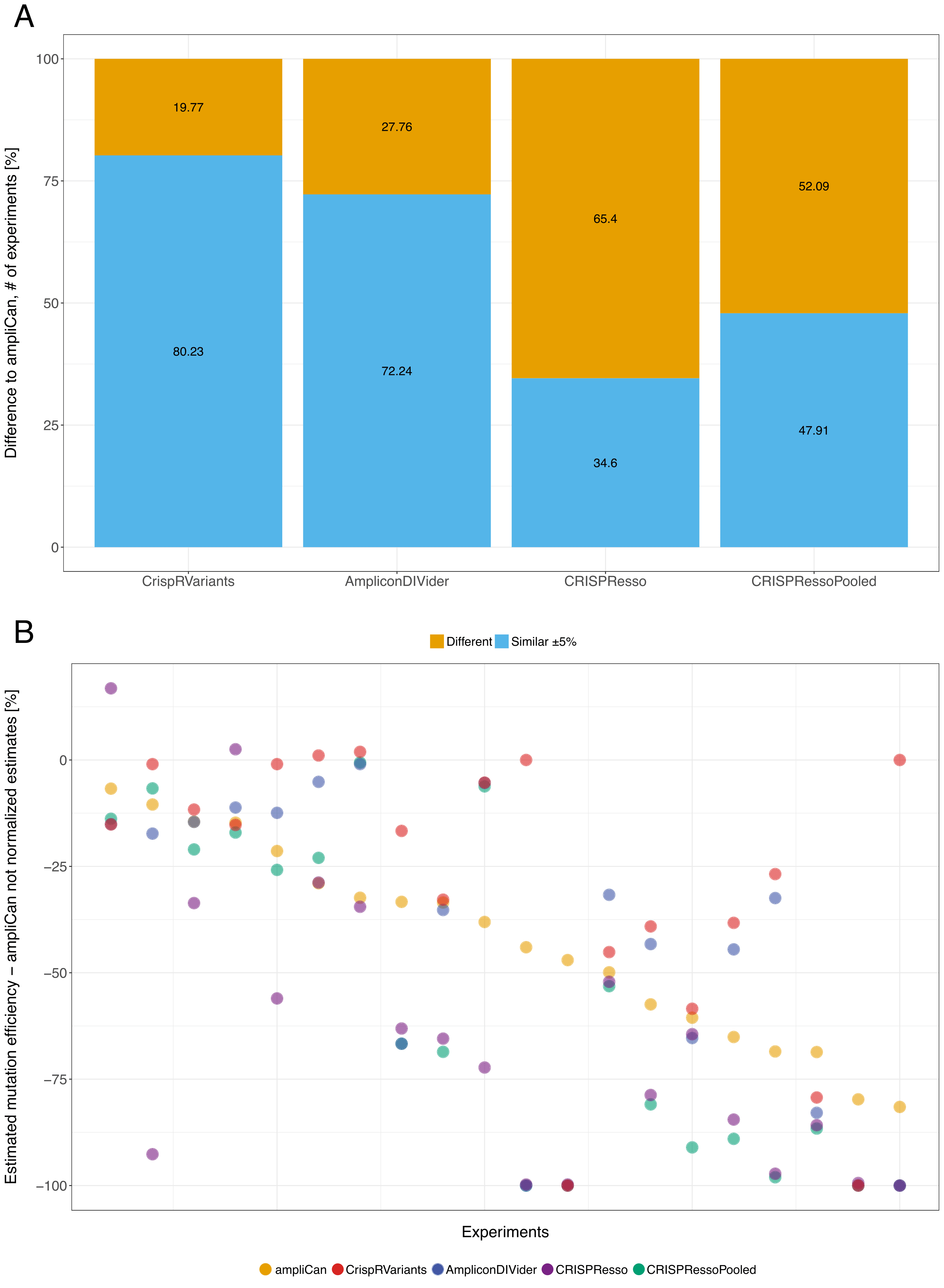

Supplement: Supplemental Material [file supp_gr.244293.118_Supplemental_Code_S1.zip › amplican_manuscript/figures/supp_fig_3-1.png]

Frame

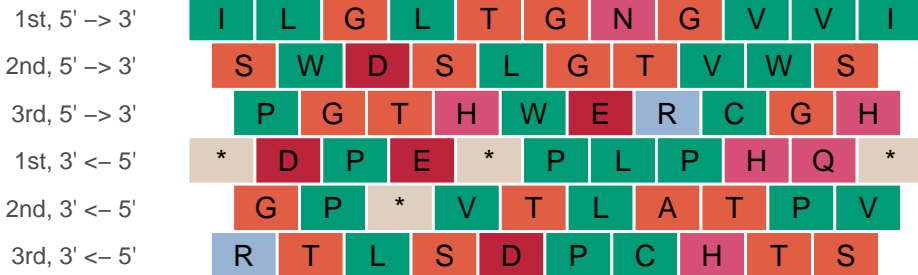

[%]

0 25 50 75 100

Match 97

Edited 0

F 3

amplicon

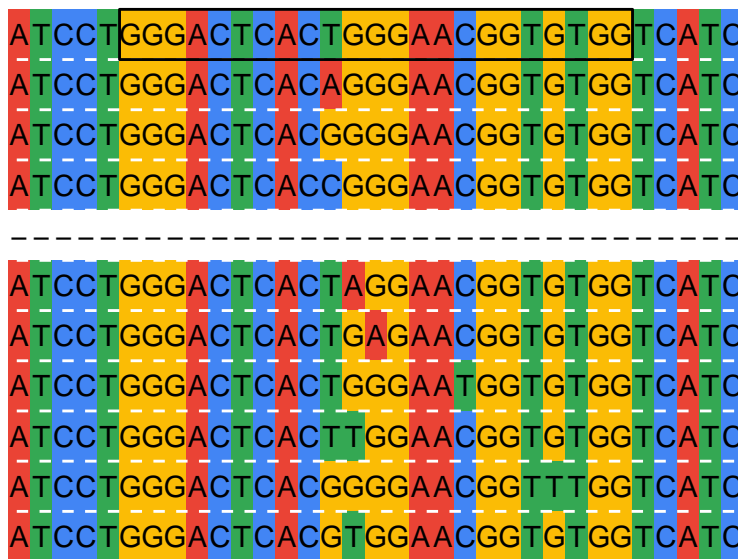

0

10

20

Relative Nucleotide Position

| Freq | Count | F    |
|------|-------|------|
| 0.74 | 2412  | 0    |
| 0.11 | 358   | 0    |
| 0.07 | 213   | 0    |
| 0.04 | 133   | 0    |
| 0.03 | 88    | -106 |
| 0    | 7     | 0    |
| 0    | 5     | 0    |
| 0    | 4     | 0    |
| 0    | 2     | 0    |
| 0    | 2     | 0    |
| 0    | 2     | 0    |

Uninjected\_aplnrB

Supplement: Supplemental Material [file supp_gr.244293.118_Supplemental_Code_S1.zip › amplican_manuscript/figures/normalization/MiSeq_run1/Injected_aplnrB_control.pdf]

Frame

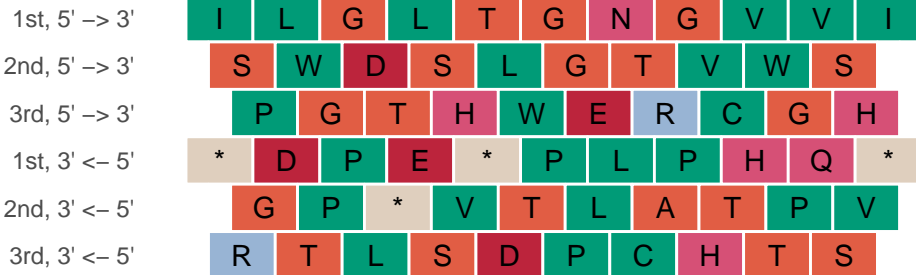

[%]

0 25 50 75 100

Match

67

Edited

8

F

26

amplicon

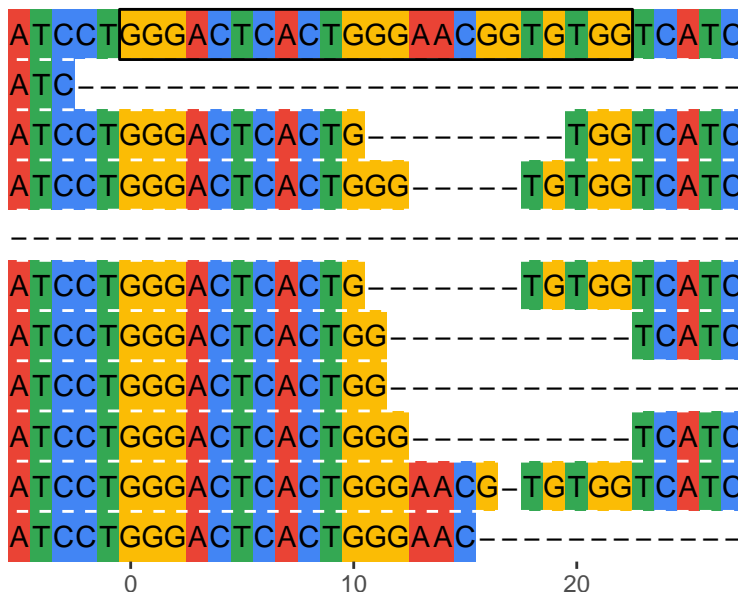

Freq

Count

F

|      |      |      |
|------|------|------|
| 0.65 | 1150 | 0    |
| 0.08 | 138  | -76  |
| 0.03 | 47   | -9   |
| 0.02 | 36   | -5   |
| 0.01 | 19   | -109 |
| 0.01 | 17   | -7   |
| 0.01 | 15   | -11  |
| 0.01 | 12   | -69  |
| 0.01 | 12   | -10  |
| 0.01 | 10   | -1   |
| 0    | 7    | -52  |

Injected\_aplhrB

Supplement: Supplemental Material [file supp_gr.244293.118_Supplemental_Code_S1.zip › amplican_manuscript/figures/normalization/MiSeq_run1/Injected_aplnrB_normalized.pdf]

Frame

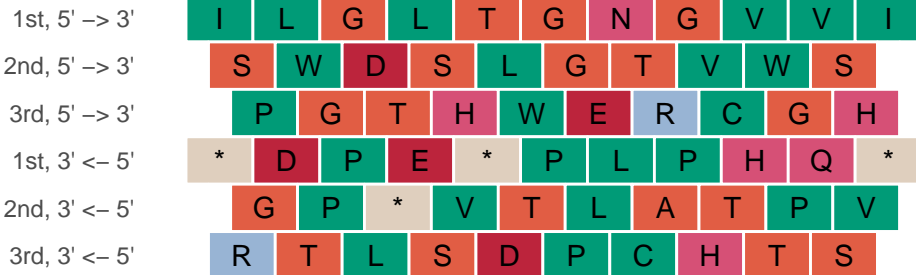

[%]

0 25 50 75 100

Match

62

Edited

8

F

30

amplicon

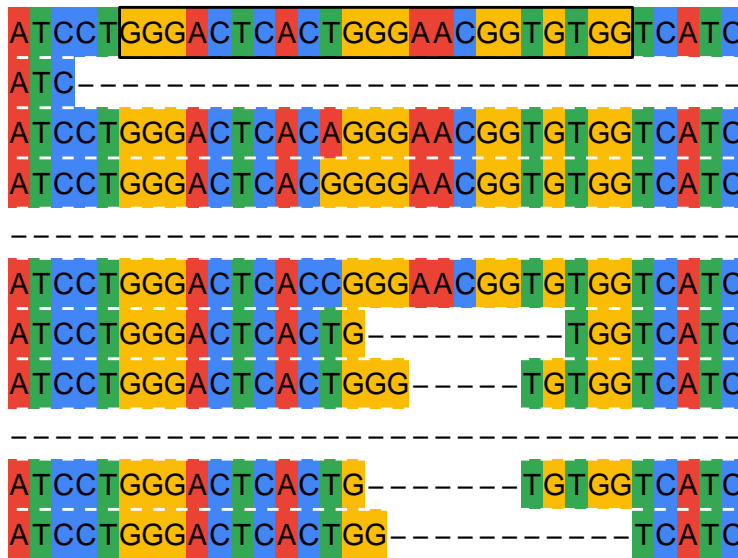

0

10

20

Relative Nucleotide Position

Freq

Count

F

0.45

808

0

0.08

138

-76

0.06

114

0

0.06

110

0

0.04

78

-106

0.02

40

0

0.02

35

-9

0.02

27

-5

0.01

19

-109

0.01

14

-7

0.01

13

-11

Supplement: Supplemental Material [file supp_gr.244293.118_Supplemental_Code_S1.zip › amplican_manuscript/figures/normalization/MiSeq_run1/Injected_aplnrB_raw.pdf]

1st, 5' → 3'

2nd, 5' → 3'

3rd, 5' → 3'

1st, 3' ← 5'

2nd, 3' ← 5'

3rd, 3' ← 5'

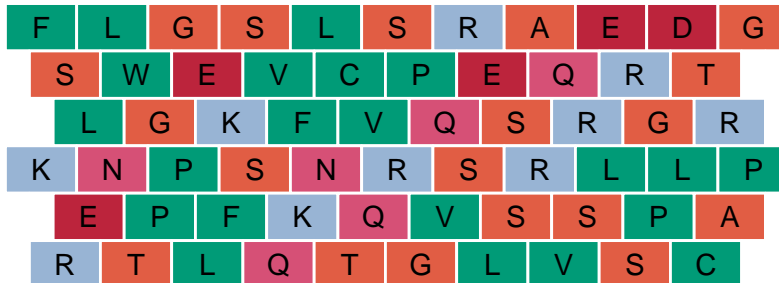

[ % ]

0 25 50 75 100

Match

100

Edited

0

F

0

amplicon

1

2

3

4

5

6

7

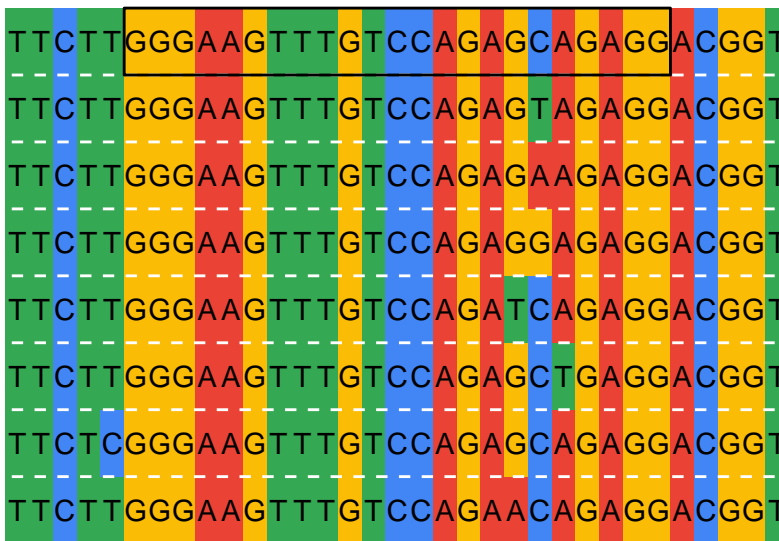

0

10

20

Relative Nucleotide Position

Freq

Count

F

0.86

2888

0

0.07

248

0

0.04

138

0

0.03

86

0

0

2

0

0

2

0

0

1

0

0

1

0

Supplement: Supplemental Material [file supp_gr.244293.118_Supplemental_Code_S1.zip › amplican_manuscript/figures/normalization/MiSeq_run1/Injected_cartb_control.pdf]

Frame

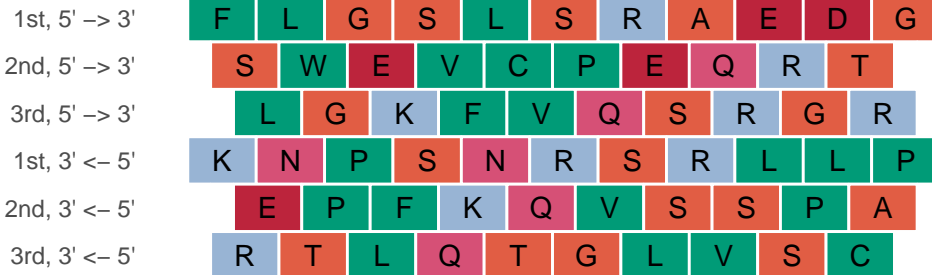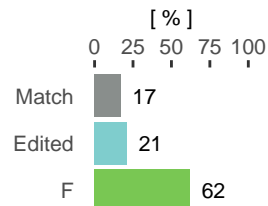

Injected\_carb

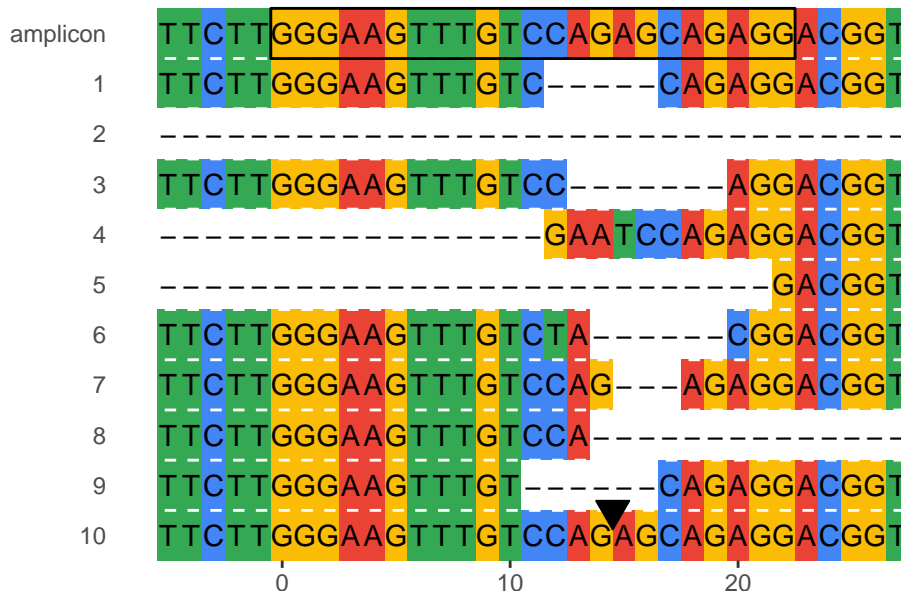

| Freq | Count | F   |
|------|-------|-----|
| 0.16 | 566   | 0   |
| 0.43 | 1551  | -5  |
| 0.09 | 339   | -87 |
| 0.03 | 108   | -7  |
| 0.02 | 67    | -54 |
| 0.01 | 52    | -51 |
| 0.01 | 40    | -6  |
| 0.01 | 35    | -3  |
| 0.01 | 34    | -29 |
| 0.01 | 33    | -6  |
| 0.01 | 31    | 1   |

Supplement: Supplemental Material [file supp_gr.244293.118_Supplemental_Code_S1.zip › amplican_manuscript/figures/normalization/MiSeq_run1/Injected_cartb_normalized.pdf]

Frame

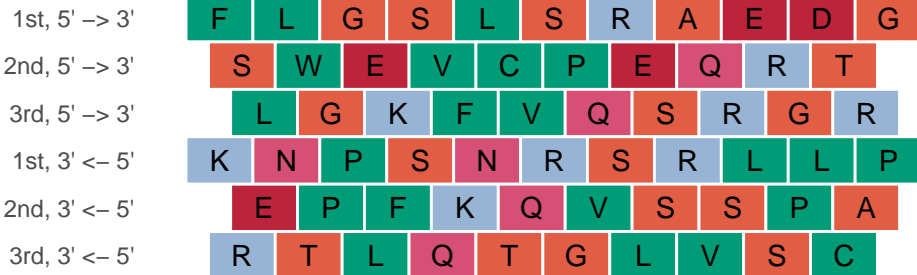

[%]

0 25 50 75 100

Match

6

Edited

23

F

71

amplicon

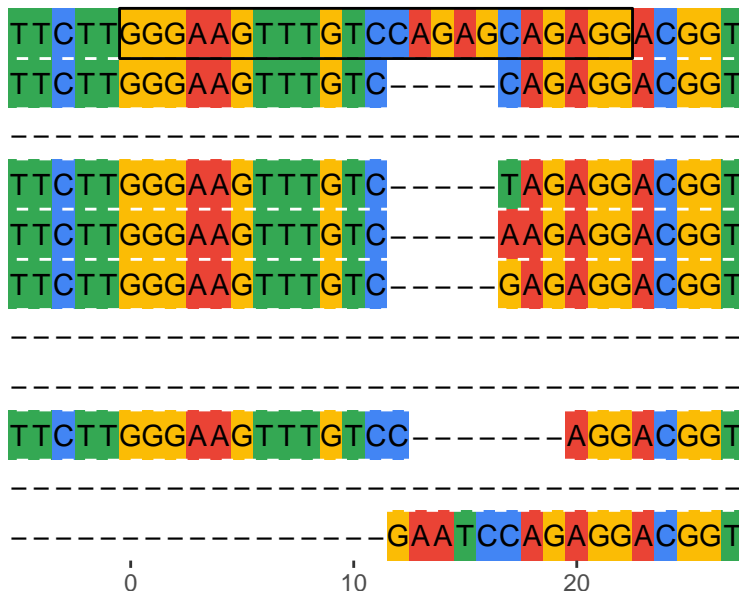

Relative Nucleotide Position

| Freq | Count | F    |
|------|-------|------|
| 0.04 | 156   | 0    |
| 0.28 | 1011  | -5   |
| 0.09 | 339   | -87  |
| 0.06 | 213   | -5   |
| 0.05 | 177   | -5   |
| 0.04 | 150   | -5   |
| 0.04 | 141   | -110 |
| 0.03 | 110   | -103 |
| 0.03 | 108   | -7   |
| 0.02 | 77    | -102 |
| 0.02 | 62    | -54  |

Supplement: Supplemental Material [file supp_gr.244293.118_Supplemental_Code_S1.zip › amplican_manuscript/figures/normalization/MiSeq_run1/Injected_cartb_raw.pdf]

Frame

Uninjected\_cartc

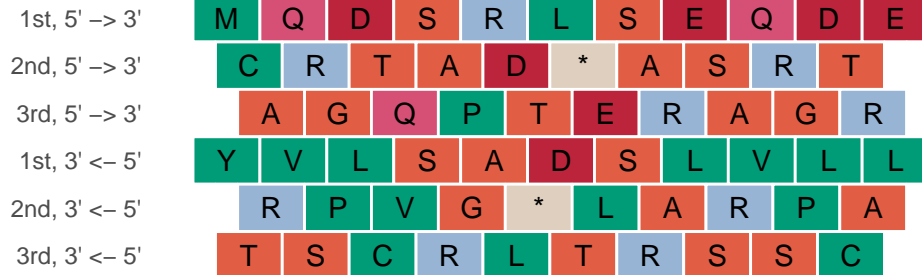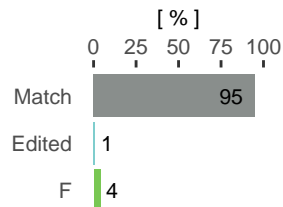

amplicon

1

2

3

4

5

6

7

8

9

10

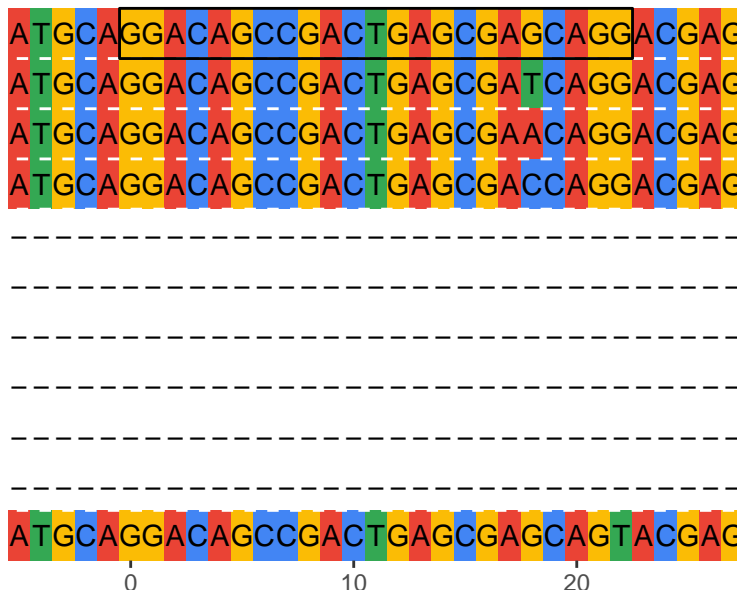

| Freq | Count | F    |
|------|-------|------|
| 0.73 | 8586  | 0    |
| 0.13 | 1524  | 0    |
| 0.05 | 601   | 0    |
| 0.03 | 396   | 0    |
| 0.01 | 176   | -95  |
| 0.01 | 173   | -95  |
| 0    | 52    | -92  |
| 0    | 49    | -93  |
| 0    | 18    | -96  |
| 0    | 11    | -122 |
| 0    | 10    | 0    |

Supplement: Supplemental Material [file supp_gr.244293.118_Supplemental_Code_S1.zip › amplican_manuscript/figures/normalization/MiSeq_run1/Injected_cartc_control.pdf]

Frame

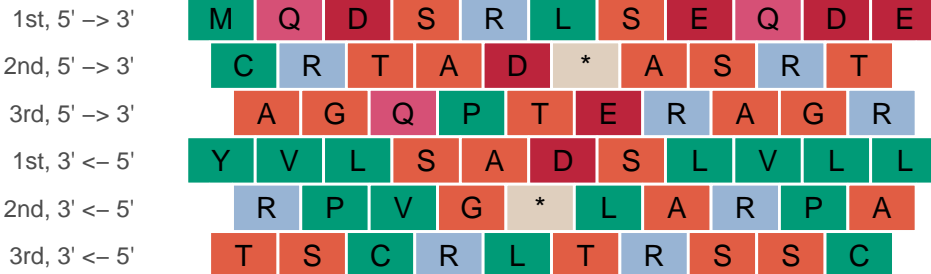

[ % ]

0 25 50 75 100

Match 69

Edited 8

F 24

amplicon

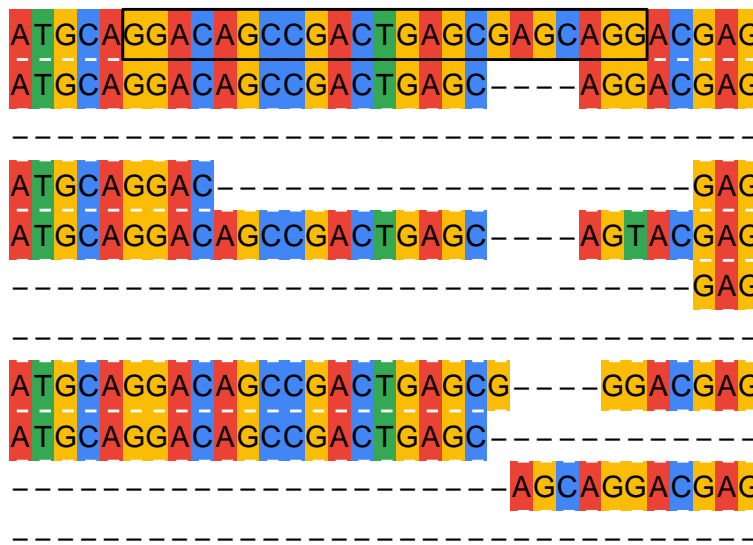

0

10

20

Relative Nucleotide Position

| Freq | Count | F   |
|------|-------|-----|
| 0.67 | 4897  | 0   |
| 0.04 | 295   | -4  |
| 0.02 | 153   | -75 |
| 0.02 | 119   | -21 |
| 0.01 | 93    | -4  |
| 0.01 | 71    | -56 |
| 0.01 | 67    | -70 |
| 0.01 | 66    | -4  |
| 0.01 | 61    | -13 |
| 0.01 | 59    | -40 |
| 0.01 | 46    | -77 |

Injected\_cartc

Supplement: Supplemental Material [file supp_gr.244293.118_Supplemental_Code_S1.zip › amplican_manuscript/figures/normalization/MiSeq_run1/Injected_cartc_normalized.pdf]

Frame

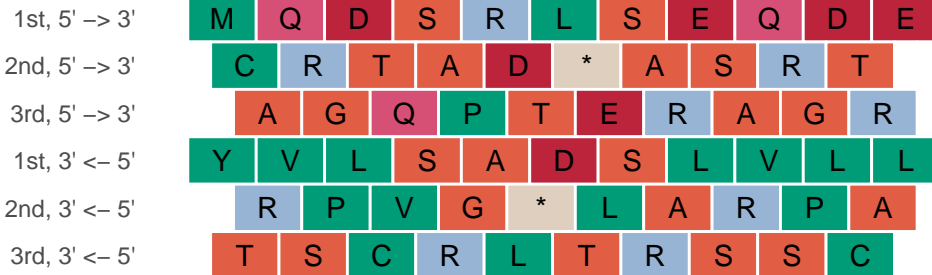[ % ]  
0 25 50 75 100

Match 67

Edited 8

F 25

amplicon

1

2

3

4

5

6

7

8

9

10

Injected\_carc

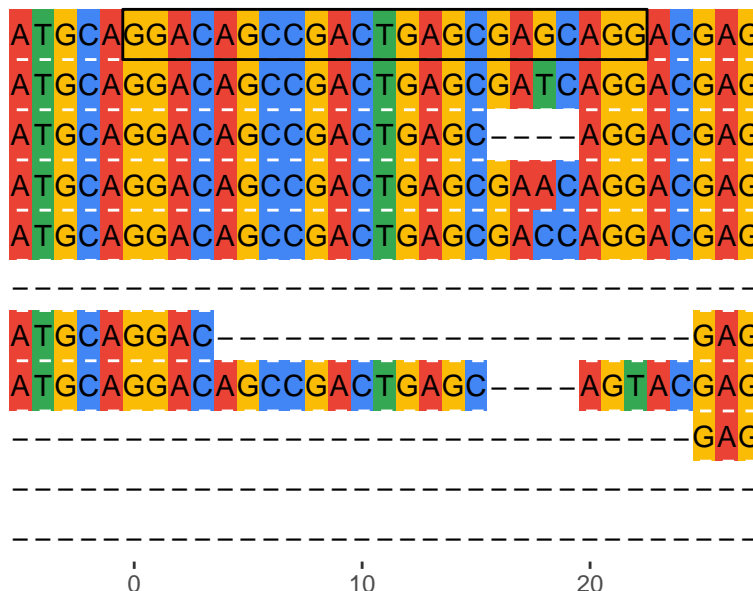

| Freq | Count | F   |
|------|-------|-----|
| 0.51 | 3689  | 0   |
| 0.09 | 681   | 0   |
| 0.04 | 295   | -4  |
| 0.03 | 241   | 0   |
| 0.02 | 179   | 0   |
| 0.02 | 153   | -75 |
| 0.02 | 119   | -21 |
| 0.01 | 93    | -4  |
| 0.01 | 71    | -56 |
| 0.01 | 71    | -95 |
| 0.01 | 67    | -70 |

Supplement: Supplemental Material [file supp_gr.244293.118_Supplemental_Code_S1.zip › amplican_manuscript/figures/normalization/MiSeq_run1/Injected_cartc_raw.pdf]

Frame

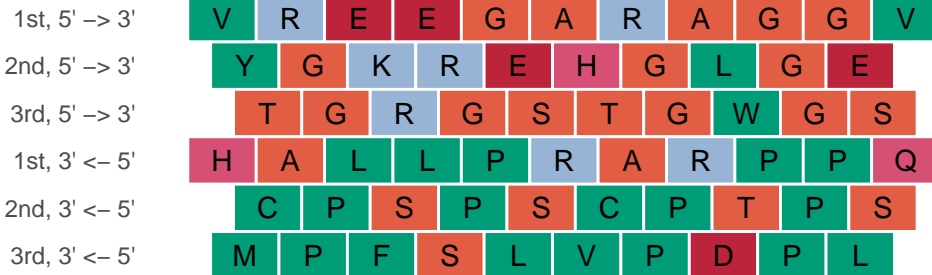

[ % ]

0 25 50 75 100

Match

80

Edited

8

F

12

amplicon

1

2

3

4

5

6

7

8

9

10

Injected\_eomesb

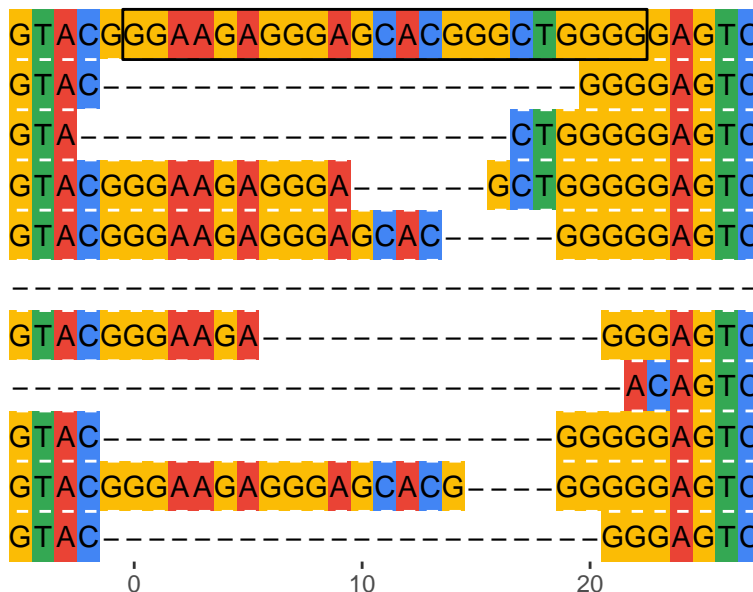

0

10

20

Relative Nucleotide Position

| Freq | Count | F   |
|------|-------|-----|
| 0.78 | 2515  | 0   |
| 0.03 | 98    | -21 |
| 0.02 | 69    | -19 |
| 0.01 | 33    | -6  |
| 0.01 | 30    | -5  |
| 0.01 | 28    | -80 |
| 0.01 | 26    | -15 |
| 0.01 | 25    | -27 |
| 0.01 | 21    | -20 |
| 0.01 | 17    | -4  |
| 0    | 16    | -22 |

Supplement: Supplemental Material [file supp_gr.244293.118_Supplemental_Code_S1.zip › amplican_manuscript/figures/normalization/MiSeq_run1/Injected_eomesb_normalized.pdf]

Frame

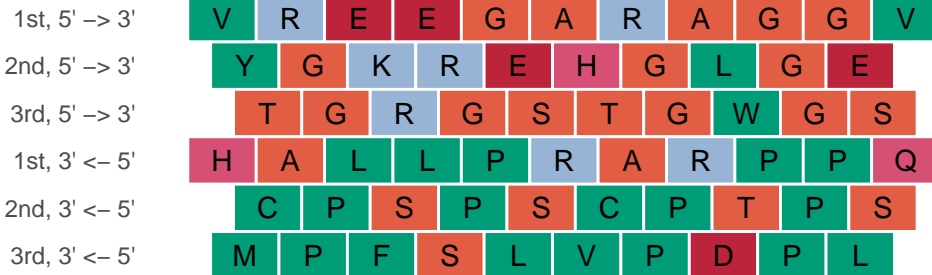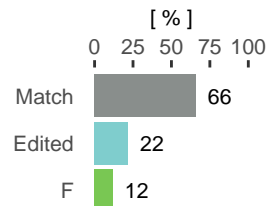

Injected\_eomesb

amplicon

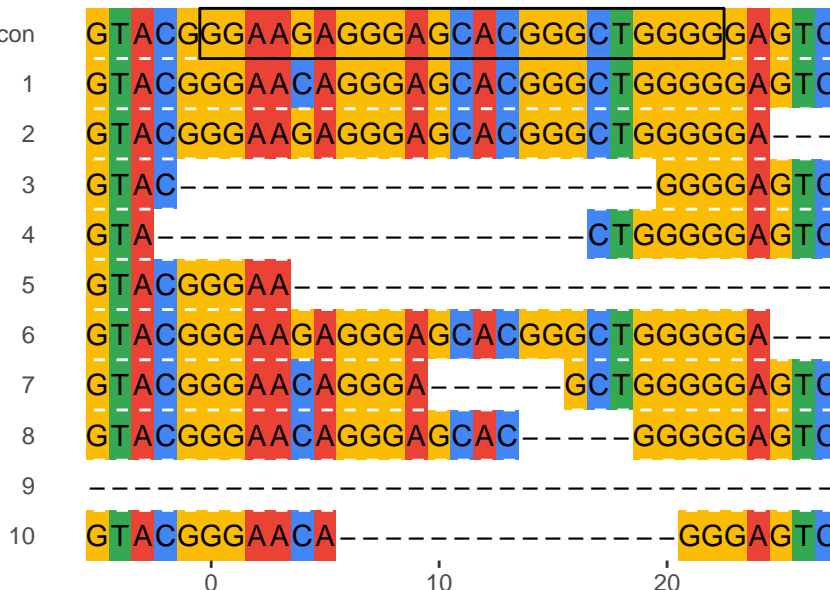

Relative Nucleotide Position

| Freq | Count | F   |
|------|-------|-----|
| 0.03 | 103   | 0   |
| 0.61 | 1946  | 0   |
| 0.11 | 356   | -66 |
| 0.03 | 98    | -21 |
| 0.02 | 69    | -19 |
| 0.02 | 50    | -87 |
| 0.01 | 36    | -69 |
| 0.01 | 33    | -6  |
| 0.01 | 30    | -5  |
| 0.01 | 28    | -80 |
| 0.01 | 26    | -15 |

Supplement: Supplemental Material [file supp_gr.244293.118_Supplemental_Code_S1.zip › amplican_manuscript/figures/normalization/MiSeq_run1/Injected_eomesb_raw.pdf]

Frame

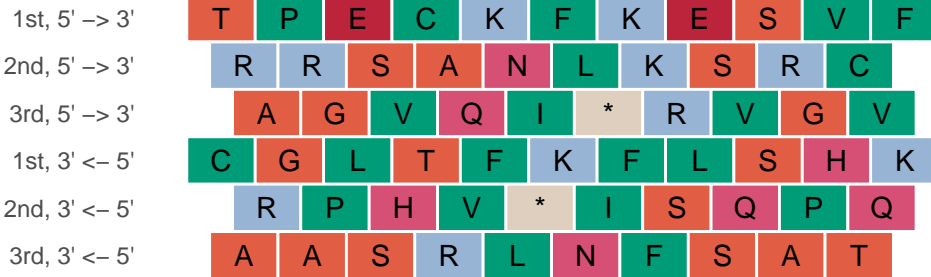

[%]

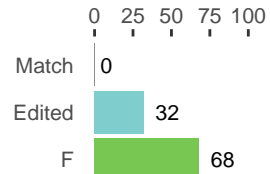

Uninjected\_fgf13b

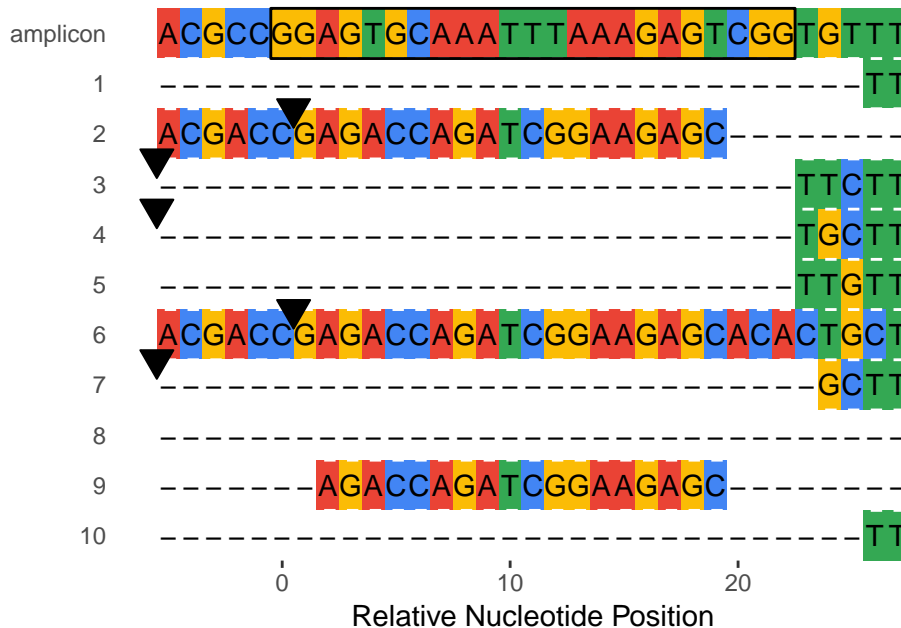

| Freq | Count | F   |
|------|-------|-----|
| 0    | 0     | 0   |
| 0.18 | 82    | -40 |
| 0.11 | 49    | -18 |
| 0.09 | 40    | 26  |
| 0.08 | 36    | 26  |
| 0.05 | 20    | -42 |
| 0.03 | 15    | 9   |
| 0.03 | 14    | 16  |
| 0.02 | 10    | -71 |
| 0.02 | 9     | -70 |
| 0.02 | 8     | -49 |

Supplement: Supplemental Material [file supp_gr.244293.118_Supplemental_Code_S1.zip › amplican_manuscript/figures/normalization/MiSeq_run1/Injected_fgf13b_control.pdf]

Frame

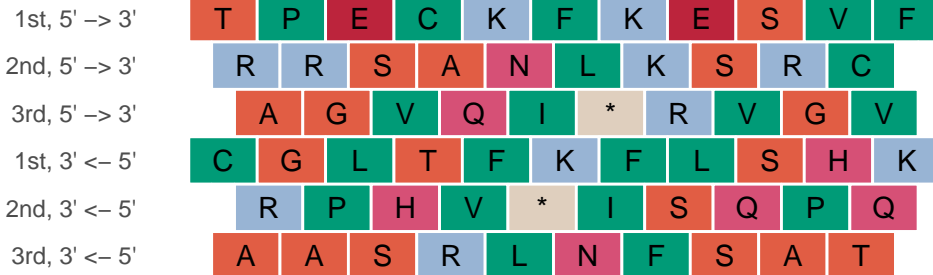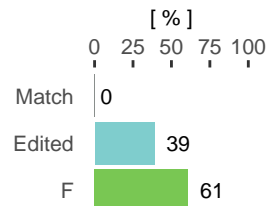

amplicon

ACGCCGGAGTGCAAATTTAAAGAGTCGGTGTTT

Injected\_fgf13b

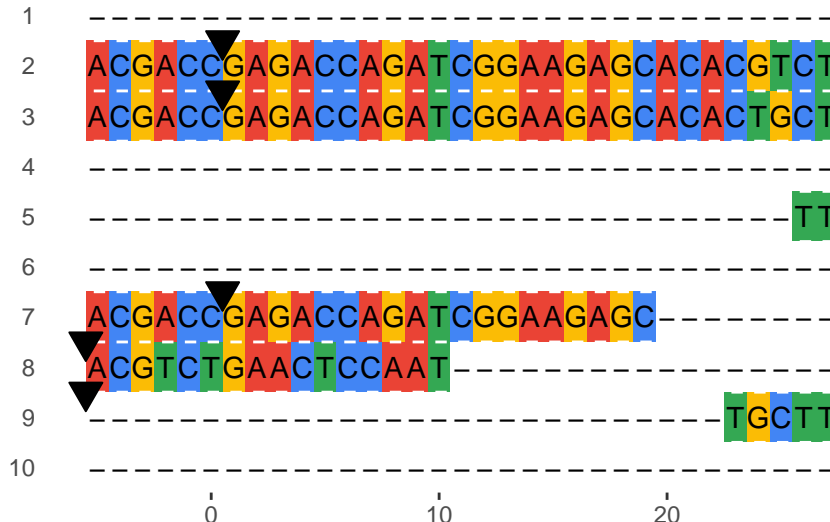

| Freq | Count | F   |
|------|-------|-----|
| 0    | 0     | 0   |
| 0.31 | 179   | -67 |
| 0.15 | 86    | 9   |
| 0.1  | 59    | 9   |
| 0.09 | 51    | -71 |
| 0.06 | 35    | -40 |
| 0.04 | 20    | -66 |
| 0.02 | 10    | -18 |
| 0.02 | 9     | -12 |
| 0.02 | 9     | 0   |
| 0.01 | 7     | -95 |

Supplement: Supplemental Material [file supp_gr.244293.118_Supplemental_Code_S1.zip › amplican_manuscript/figures/normalization/MiSeq_run1/Injected_fgf13b_raw.pdf]

1st, 5' → 3'

2nd, 5' → 3'

3rd, 5' → 3'

1st, 3' ← 5'

2nd, 3' ← 5'

3rd, 3' ← 5'

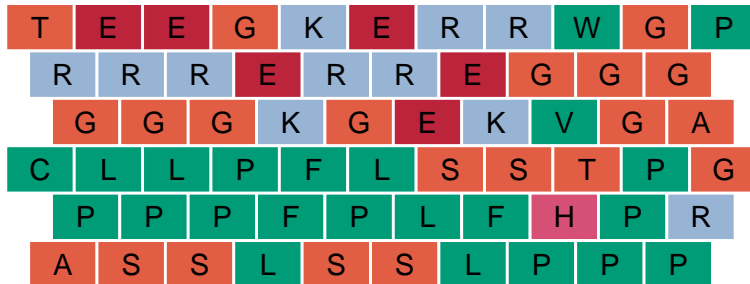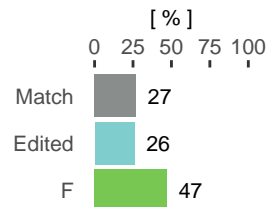

amplicon

1

2

3

4

5

6

7

8

9

10

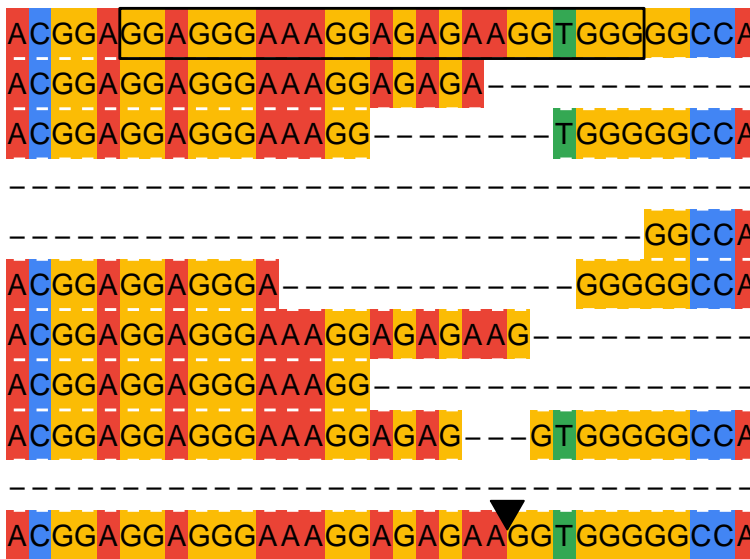

0

10

20

Relative Nucleotide Position

| Freq | Count | F   |
|------|-------|-----|
| 0.25 | 1486  | 0   |
| 0.08 | 471   | -12 |
| 0.05 | 298   | -8  |
| 0.03 | 175   | -71 |
| 0.03 | 174   | -76 |
| 0.03 | 153   | -13 |
| 0.02 | 145   | -11 |
| 0.02 | 126   | -64 |
| 0.02 | 109   | -3  |
| 0.02 | 94    | -63 |
| 0.02 | 89    | 2   |

Supplement: Supplemental Material [file supp_gr.244293.118_Supplemental_Code_S1.zip › amplican_manuscript/figures/normalization/MiSeq_run1/Injected_NC1b_megamind_normalized.pdf]

1st, 5' → 3'

2nd, 5' → 3'

3rd, 5' → 3'

1st, 3' ← 5'

2nd, 3' ← 5'

3rd, 3' ← 5'

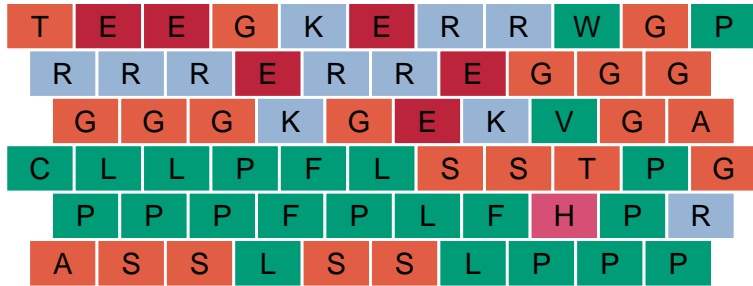

[ % ]

Match 26

Edited 26

F 48

amplicon

1

2

3

4

5

6

7

8

9

10

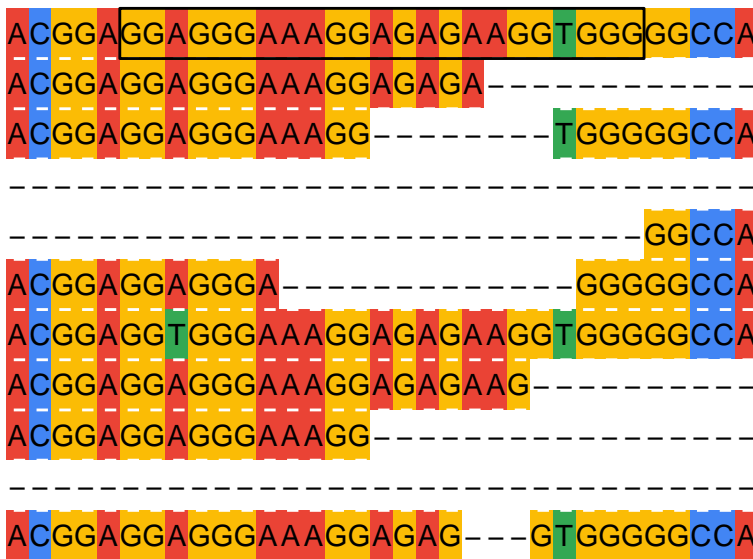

0

10

20

Relative Nucleotide Position

| Freq | Count | F   |
|------|-------|-----|
| 0.19 | 1142  | 0   |
| 0.07 | 391   | -12 |
| 0.04 | 252   | -8  |
| 0.03 | 175   | -71 |
| 0.03 | 174   | -76 |
| 0.02 | 126   | -13 |
| 0.02 | 116   | 0   |
| 0.02 | 112   | -11 |
| 0.02 | 96    | -64 |
| 0.02 | 94    | -63 |
| 0.01 | 85    | -3  |

Supplement: Supplemental Material [file supp_gr.244293.118_Supplemental_Code_S1.zip › amplican_manuscript/figures/normalization/MiSeq_run1/Injected_NC1b_megamind_raw.pdf]

1st, 5' → 3'

2nd, 5' → 3'

3rd, 5' → 3'

1st, 3' ← 5'

2nd, 3' ← 5'

3rd, 3' ← 5'

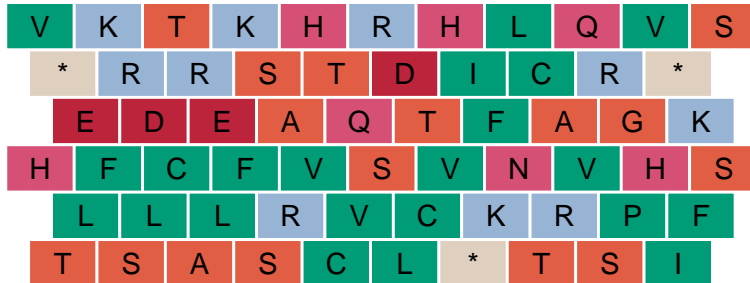

[ % ]

0 25 50 75 100

Match

84

Edited

4

F

12

amplicon

1

2

3

4

5

6

7

8

9

10

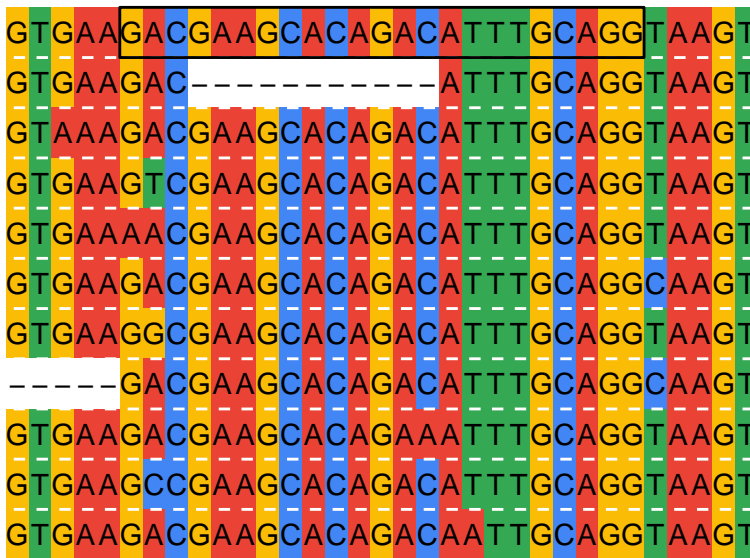

0

10

20

Relative Nucleotide Position

| Freq | Count | F   |
|------|-------|-----|
| 0.51 | 1474  | 0   |
| 0.1  | 278   | -11 |
| 0.05 | 157   | 0   |
| 0.04 | 119   | 0   |
| 0.04 | 104   | 0   |
| 0.03 | 77    | 0   |
| 0.02 | 60    | 0   |
| 0.02 | 58    | -12 |
| 0.02 | 52    | 0   |
| 0.02 | 44    | 0   |
| 0.01 | 42    | 0   |

Supplement: Supplemental Material [file supp_gr.244293.118_Supplemental_Code_S1.zip › amplican_manuscript/figures/normalization/MiSeq_run1/Injected_NC4_Cingulin_intronic_control.pdf]

1st, 5' → 3'

2nd, 5' → 3'

3rd, 5' → 3'

1st, 3' ← 5'

2nd, 3' ← 5'

3rd, 3' ← 5'

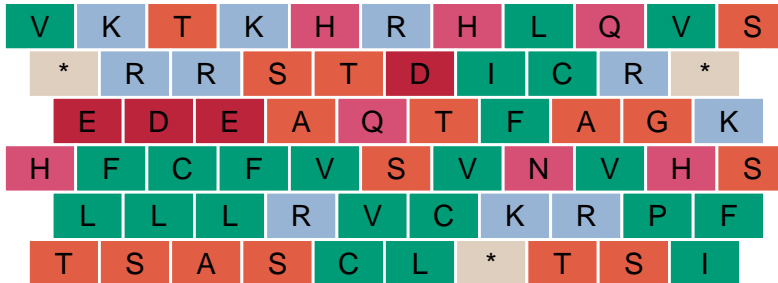

[ % ]  
0 25 50 75 100

Match

Edited

F

100

0

0

amplicon

1

2

3

4

5

6

7

8

9

10

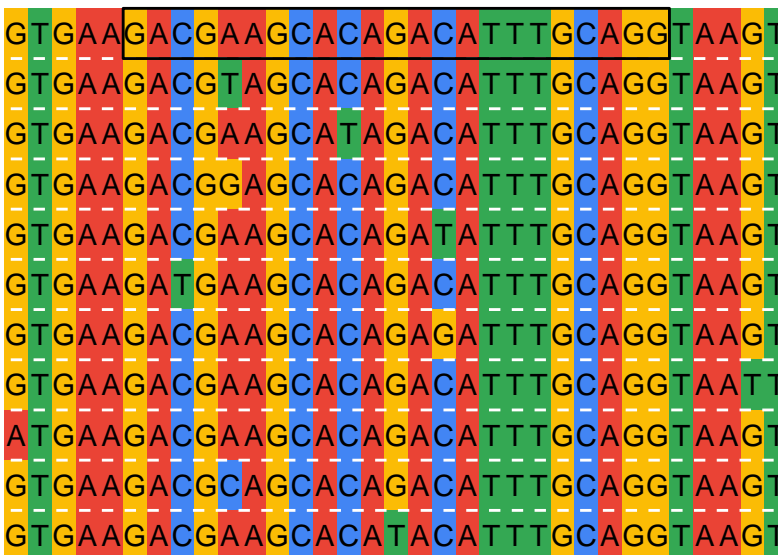

0

10

20

Relative Nucleotide Position

| Freq | Count | F |
|------|-------|---|
| 0.96 | 5987  | 0 |
| 0    | 28    | 0 |
| 0    | 22    | 0 |
| 0    | 19    | 0 |
| 0    | 14    | 0 |
| 0    | 14    | 0 |
| 0    | 12    | 0 |
| 0    | 11    | 0 |
| 0    | 10    | 0 |
| 0    | 9     | 0 |
| 0    | 9     | 0 |

Supplement: Supplemental Material [file supp_gr.244293.118_Supplemental_Code_S1.zip › amplican_manuscript/figures/normalization/MiSeq_run1/Injected_NC4_Cingulin_intronic_normalized.pdf]

Frame

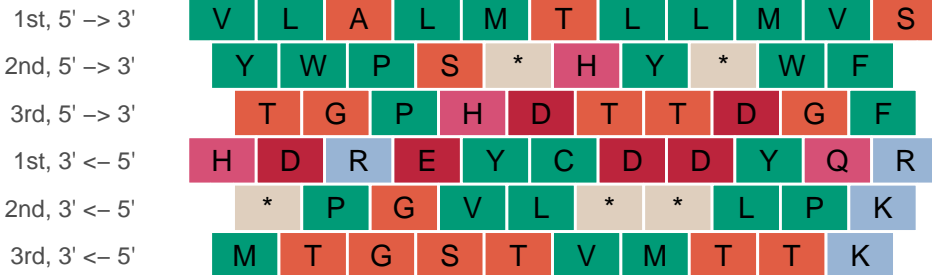

[ % ]

0 25 50 75 100

Match

96

Edited

2

F

2

amplicon

Injected\_NP005

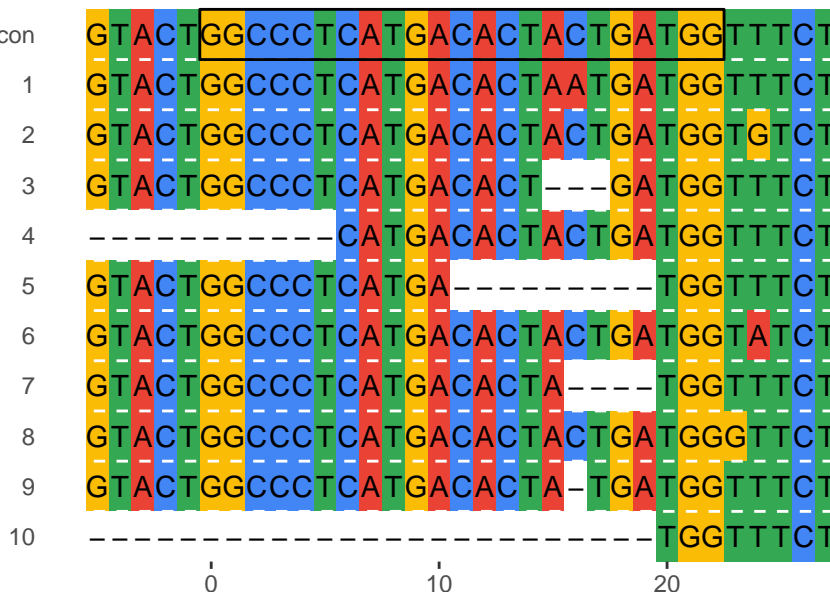

| Freq | Count | F   |
|------|-------|-----|
| 0.92 | 4911  | 0   |
| 0    | 18    | 0   |
| 0    | 17    | 0   |
| 0    | 17    | -3  |
| 0    | 17    | -37 |
| 0    | 15    | -9  |
| 0    | 13    | 0   |
| 0    | 13    | -4  |
| 0    | 11    | 0   |
| 0    | 10    | -1  |
| 0    | 10    | -27 |

Supplement: Supplemental Material [file supp_gr.244293.118_Supplemental_Code_S1.zip › amplican_manuscript/figures/normalization/MiSeq_run1/Injected_NP005_normalized.pdf]

Frame

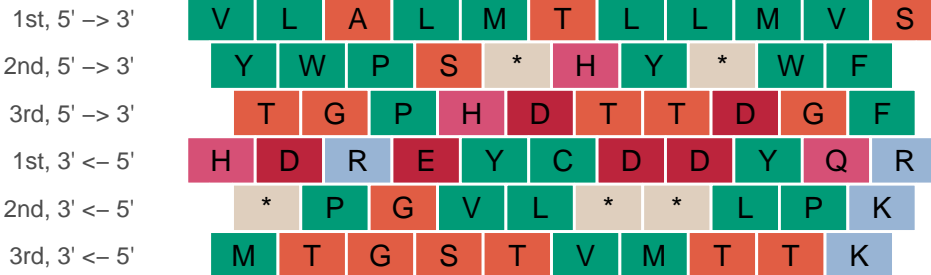

[ % ]

0 25 50 75 100

Match

94

Edited

2

F

4

amplicon

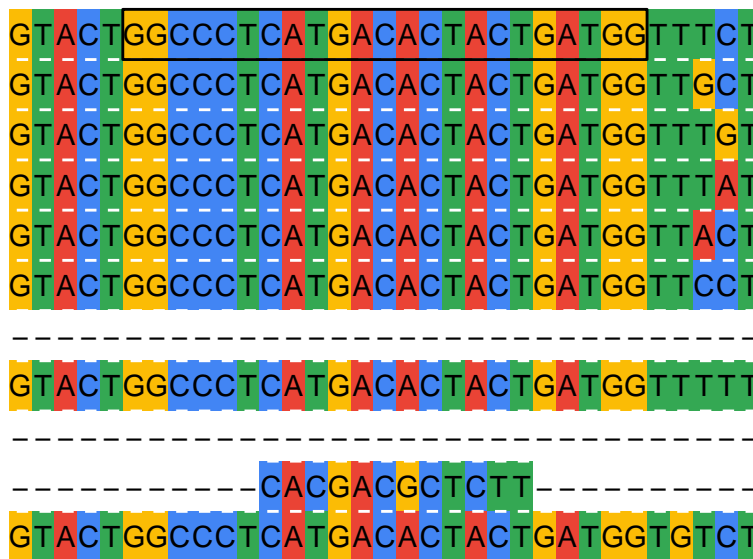

0

10

20

Relative Nucleotide Position

| Freq | Count | F   |
|------|-------|-----|
| 0.63 | 3349  | 0   |
| 0.1  | 550   | 0   |
| 0.05 | 269   | 0   |
| 0.05 | 259   | 0   |
| 0.04 | 214   | 0   |
| 0.02 | 116   | 0   |
| 0.01 | 54    | -83 |
| 0.01 | 46    | 0   |
| 0.01 | 41    | -83 |
| 0    | 17    | -54 |
| 0    | 16    | 0   |

Injected\_NP005

Supplement: Supplemental Material [file supp_gr.244293.118_Supplemental_Code_S1.zip › amplican_manuscript/figures/normalization/MiSeq_run1/Injected_NP005_raw.pdf]

Frame

Injected\_SP18

1st, 5' → 3'

2nd, 5' → 3'

3rd, 5' → 3'

1st, 3' ← 5'

2nd, 3' ← 5'

3rd, 3' ← 5'

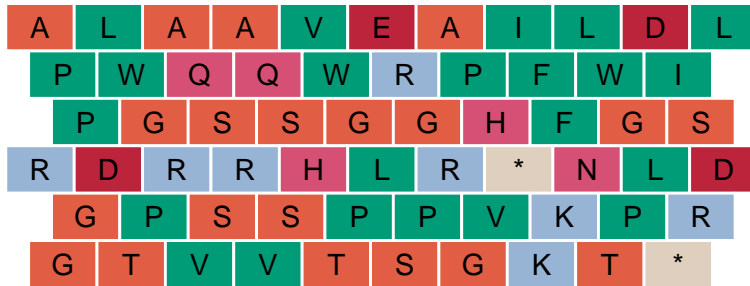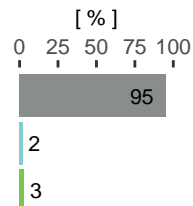

amplicon

1

2

3

4

5

6

7

8

9

10

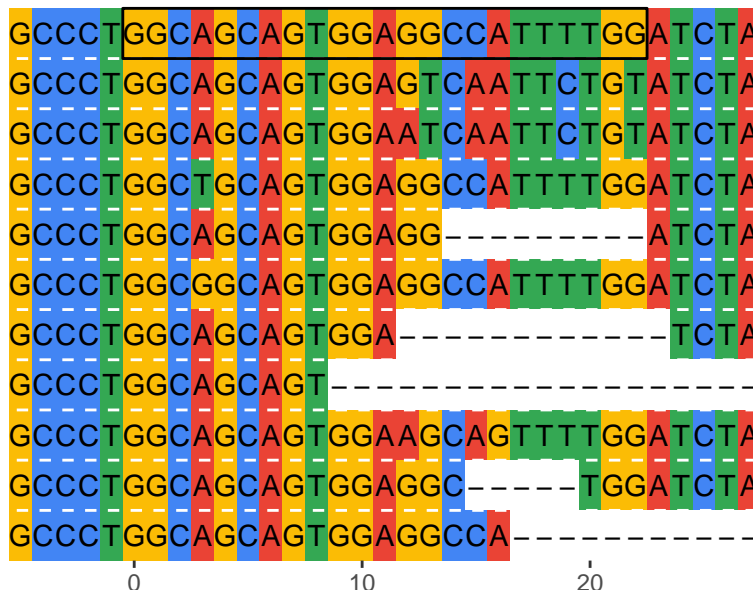

Relative Nucleotide Position

| Freq | Count | F   |
|------|-------|-----|
| 0.63 | 3594  | 0   |
| 0.19 | 1110  | 0   |
| 0.1  | 553   | 0   |
| 0.01 | 38    | 0   |
| 0.01 | 34    | -9  |
| 0    | 26    | 0   |
| 0    | 17    | -12 |
| 0    | 9     | -24 |
| 0    | 8     | 0   |
| 0    | 8     | -5  |
| 0    | 8     | -33 |

Supplement: Supplemental Material [file supp_gr.244293.118_Supplemental_Code_S1.zip › amplican_manuscript/figures/normalization/MiSeq_run1/Injected_SP18_normalized.pdf]

Frame

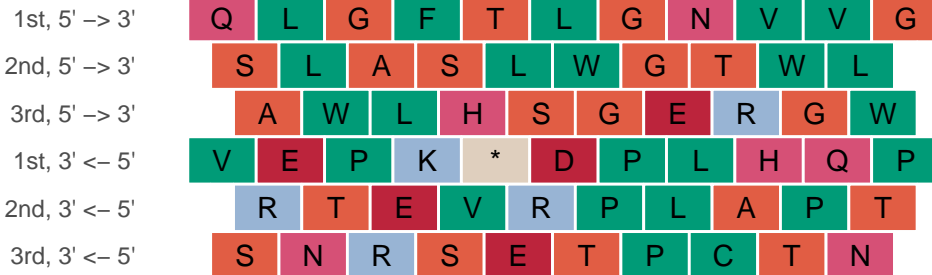

[ % ]

Match

97

Edited

0

F

2

Injected\_SP1

amplicon

1

2

3

4

5

6

7

8

9

10

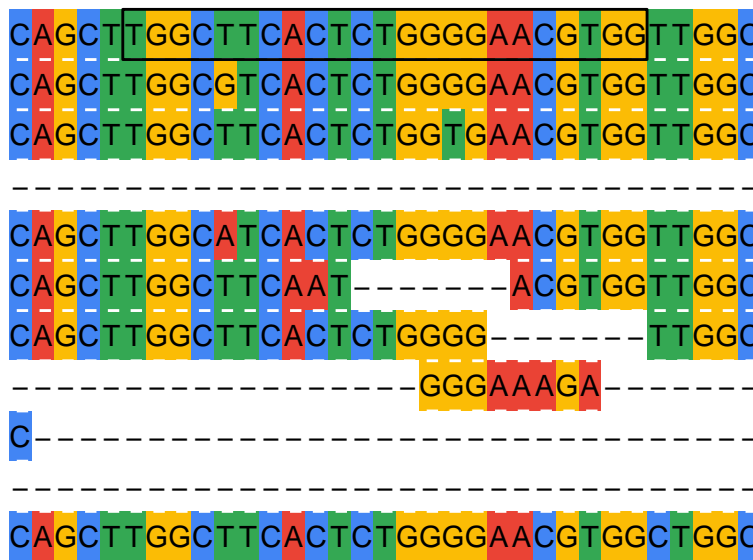

0

10

20

Relative Nucleotide Position

| Freq | Count | F   |
|------|-------|-----|
| 0.93 | 1654  | 0   |
| 0.01 | 13    | 0   |
| 0.01 | 9     | 0   |
| 0    | 6     | -49 |
| 0    | 5     | 0   |
| 0    | 5     | -7  |
| 0    | 5     | -7  |
| 0    | 5     | -95 |
| 0    | 4     | -49 |
| 0    | 4     | -53 |
| 0    | 3     | 0   |

Supplement: Supplemental Material [file supp_gr.244293.118_Supplemental_Code_S1.zip › amplican_manuscript/figures/normalization/MiSeq_run1/Injected_SP1_normalized.pdf]

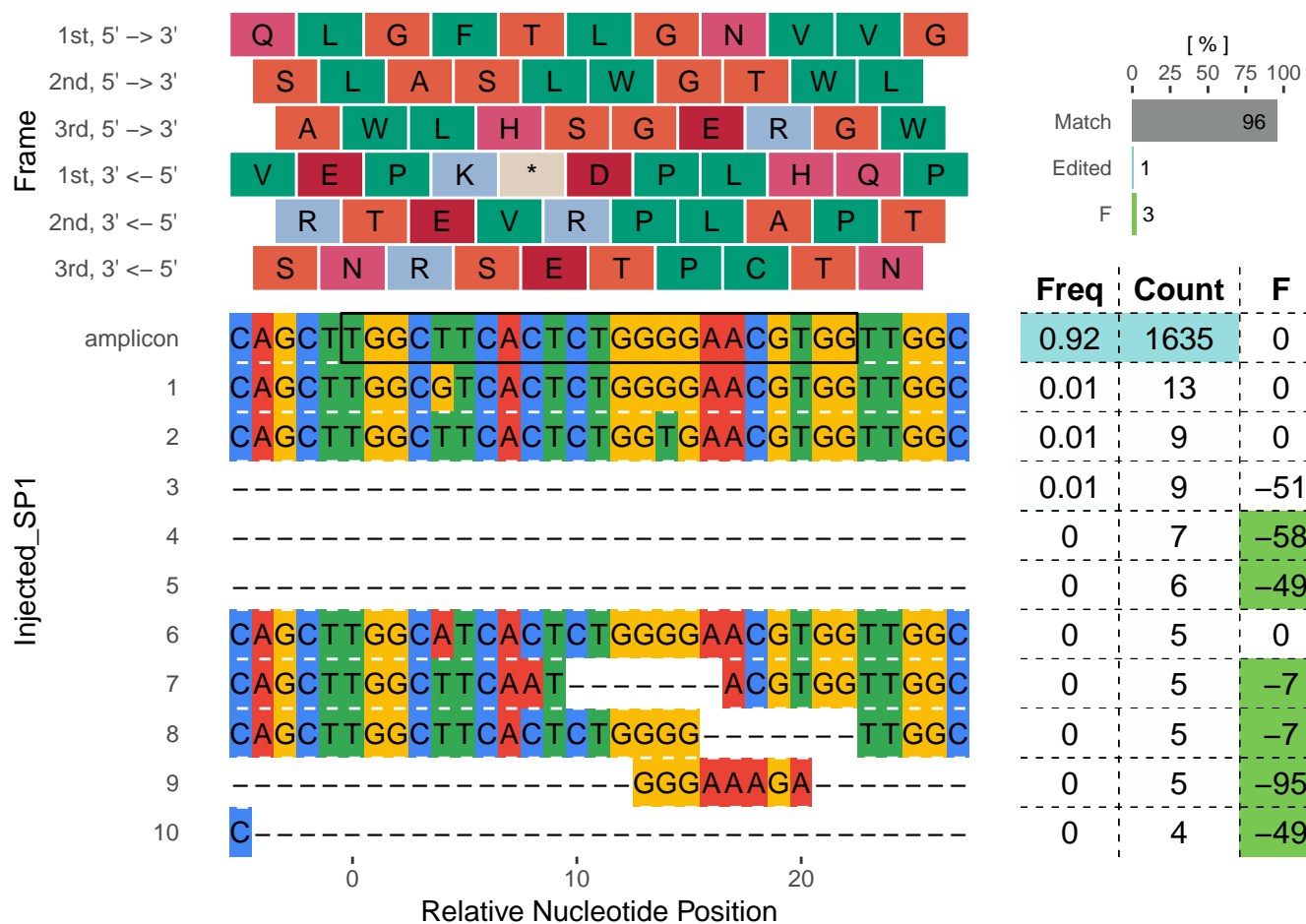

Supplement: Supplemental Material [file supp_gr.244293.118_Supplemental_Code_S1.zip › amplican_manuscript/figures/normalization/MiSeq_run1/Injected_SP1_raw.pdf]

1st, 5' → 3'

2nd, 5' → 3'

3rd, 5' → 3'

1st, 3' ← 5'

2nd, 3' ← 5'

3rd, 3' ← 5'

amplicon

1

2

3

4

5

6

7

8

9

10

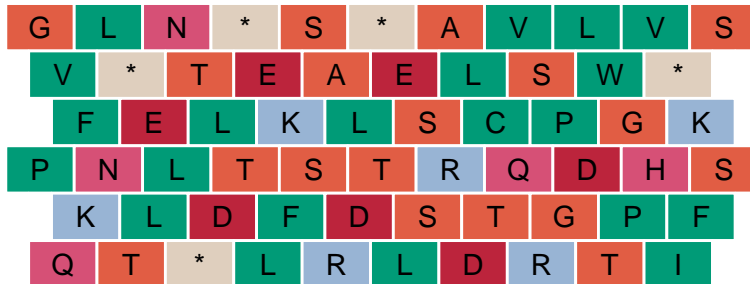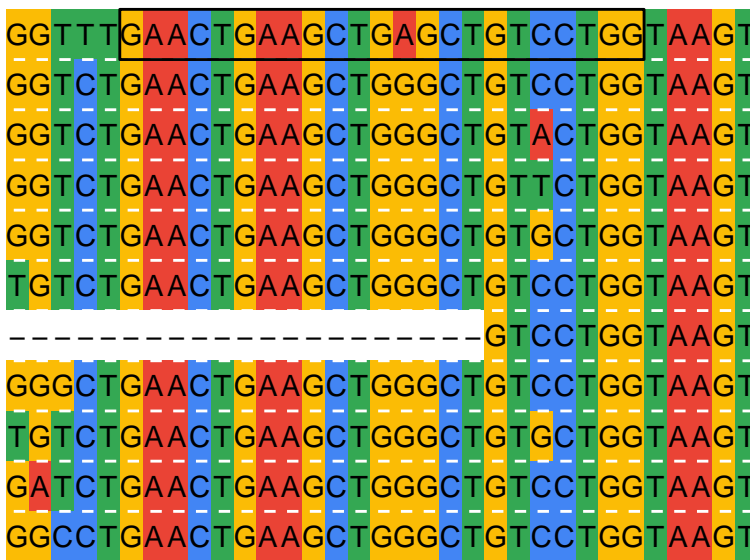

0

10

20

Relative Nucleotide Position

[%]

0 25 50 75 100

Match

97

Edited

3

F

0

Freq

Count

F

0.17

21

0

0.46

58

0

0.07

9

0

0.06

8

0

0.06

7

0

0.05

6

0

0.03

4

-54

0.02

2

0

0.02

2

0

0.01

1

0

0.01

1

0

Supplement: Supplemental Material [file supp_gr.244293.118_Supplemental_Code_S1.zip › amplican_manuscript/figures/normalization/MiSeq_run1/Injected_SP8_updated2_control.pdf]

Frame

Injected\_SP8\_updated2

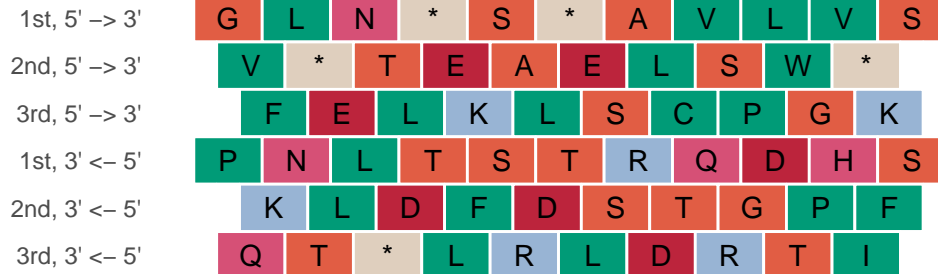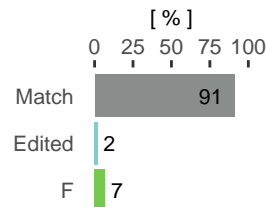

amplicon

1

2

3

4

5

6

7

8

9

10

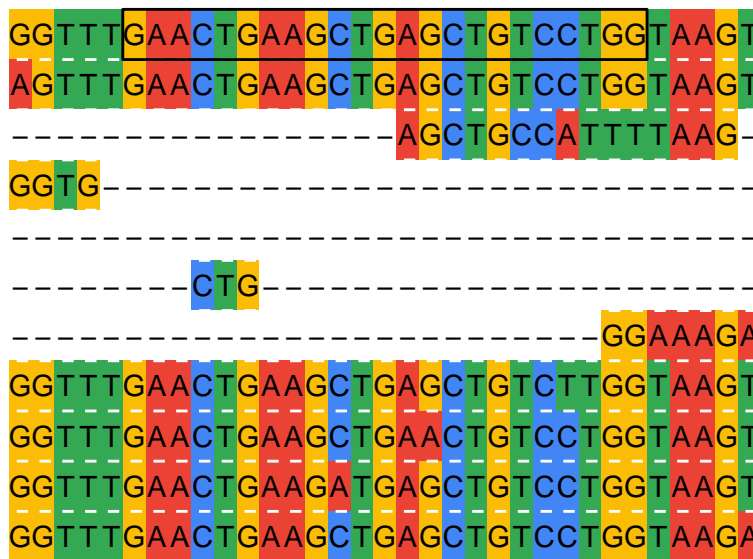

0

10

20

Relative Nucleotide Position

| Freq | Count | F   |
|------|-------|-----|
| 0.78 | 81    | 0   |
| 0.08 | 8     | 0   |
| 0.03 | 3     | -91 |
| 0.02 | 2     | -70 |
| 0.02 | 2     | -60 |
| 0.01 | 1     | -85 |
| 0.01 | 1     | -38 |
| 0.01 | 1     | 0   |
| 0.01 | 1     | 0   |
| 0.01 | 1     | 0   |
| 0.01 | 1     | 0   |

Supplement: Supplemental Material [file supp_gr.244293.118_Supplemental_Code_S1.zip › amplican_manuscript/figures/normalization/MiSeq_run1/Injected_SP8_updated2_normalized.pdf]

Frame

1st, 5' → 3'

2nd, 5' → 3'

3rd, 5' → 3'

1st, 3' ← 5'

2nd, 3' ← 5'

3rd, 3' ← 5'

amplicon

1

2

3

4

5

6

7

8

9

10

Injected\_SP8\_updated2

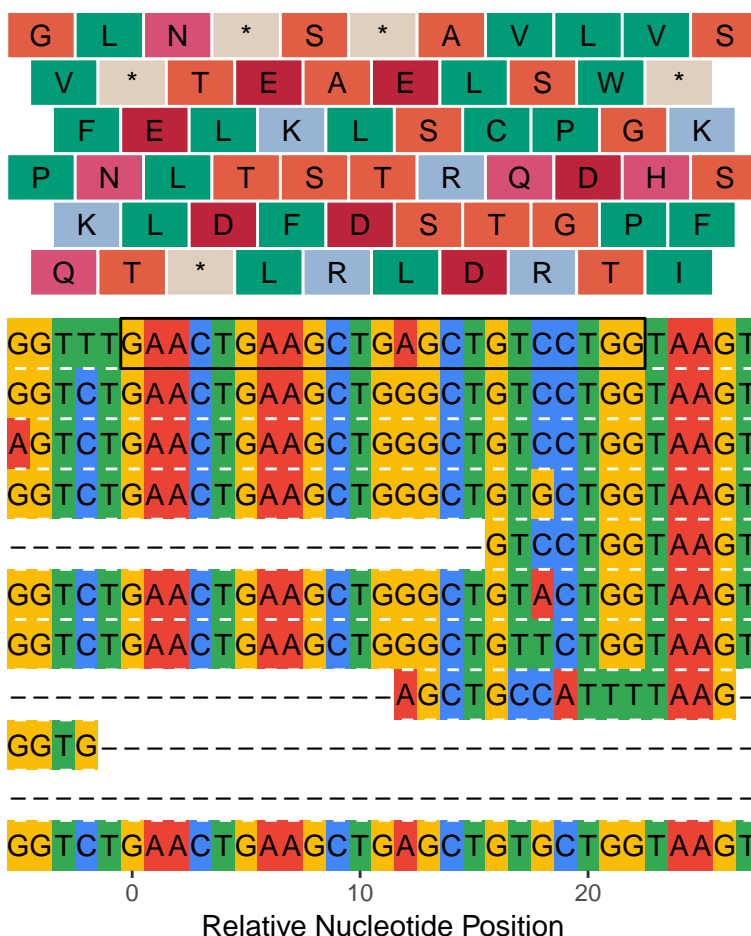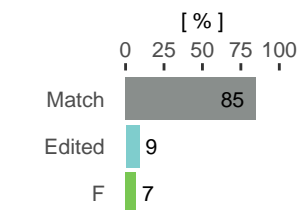

| Freq | Count | F   |
|------|-------|-----|
| 0.25 | 26    | 0   |
| 0.27 | 28    | 0   |
| 0.08 | 8     | 0   |
| 0.08 | 8     | 0   |
| 0.07 | 7     | -54 |
| 0.06 | 6     | 0   |
| 0.04 | 4     | 0   |
| 0.03 | 3     | -91 |
| 0.02 | 2     | -70 |
| 0.02 | 2     | -60 |
| 0.01 | 1     | 0   |

Supplement: Supplemental Material [file supp_gr.244293.118_Supplemental_Code_S1.zip › amplican_manuscript/figures/normalization/MiSeq_run1/Injected_SP8_updated2_raw.pdf]

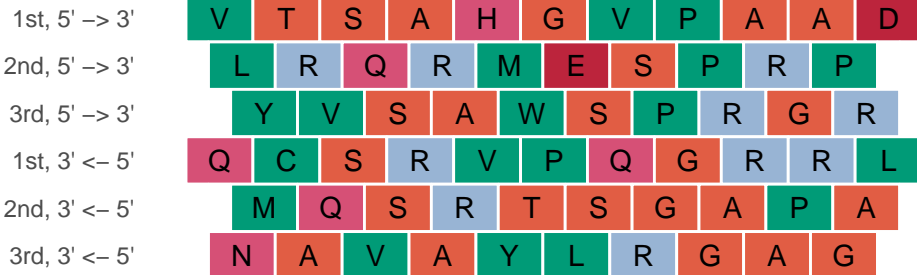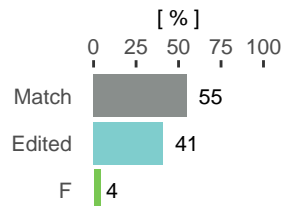

amplicon

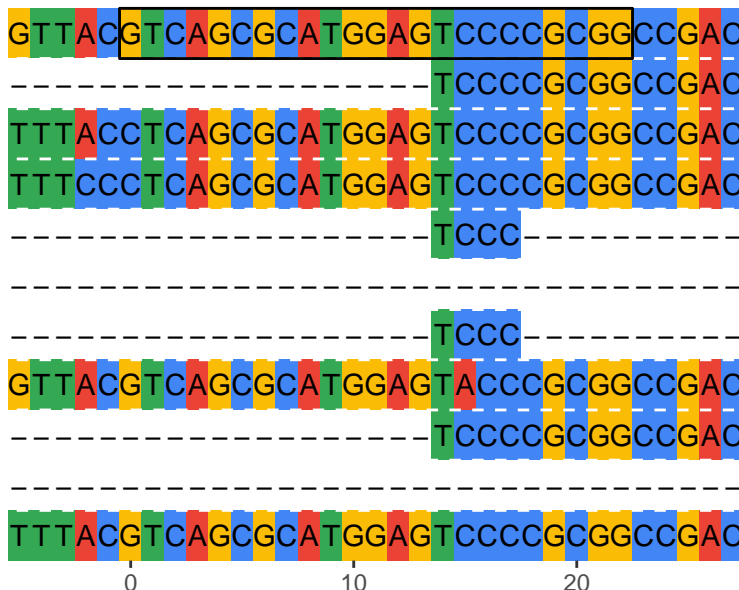

| Freq | Count | F    |
|------|-------|------|
| 0.45 | 282   | 0    |
| 0.4  | 249   | -120 |
| 0.04 | 26    | 0    |
| 0.02 | 14    | 0    |
| 0.01 | 7     | -152 |
| 0.01 | 5     | -136 |
| 0.01 | 4     | -164 |
| 0    | 3     | 0    |
| 0    | 3     | -106 |
| 0    | 3     | -99  |
| 0    | 3     | 0    |

Supplement: Supplemental Material [file supp_gr.244293.118_Supplemental_Code_S1.zip › amplican_manuscript/figures/normalization/MiSeq_run10_2014_05_16/7879_2_control.pdf]

Frame

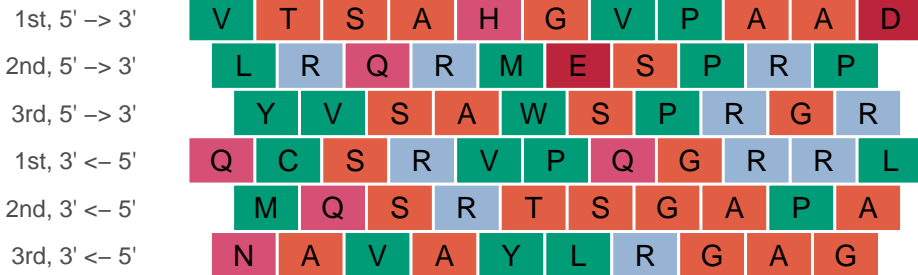

[%]

0 25 50 75 100

Match 65

Edited 29

F 5

amplicon

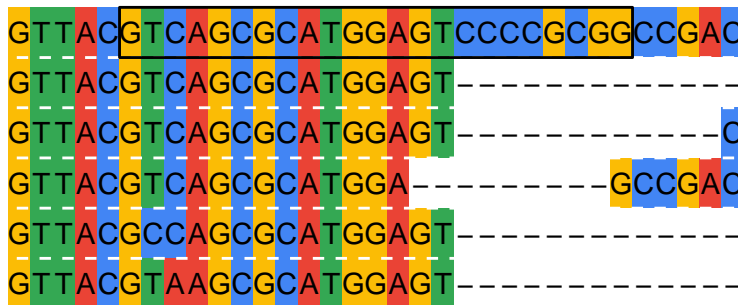

| Freq | Count | F    |
|------|-------|------|
| 0.65 | 200   | 0    |
| 0.13 | 40    | -15  |
| 0.04 | 12    | -12  |
| 0.04 | 11    | -9   |
| 0.02 | 5     | -15  |
| 0.02 | 5     | -15  |
| 0.01 | 4     | -136 |
| 0.01 | 4     | 1    |
| 0.01 | 4     | -9   |
| 0.01 | 3     | -2   |
| 0.01 | 2     | -99  |

0

10

20

Relative Nucleotide Position

7879\_2

Supplement: Supplemental Material [file supp_gr.244293.118_Supplemental_Code_S1.zip › amplican_manuscript/figures/normalization/MiSeq_run10_2014_05_16/7879_2_normalized.pdf]

Frame

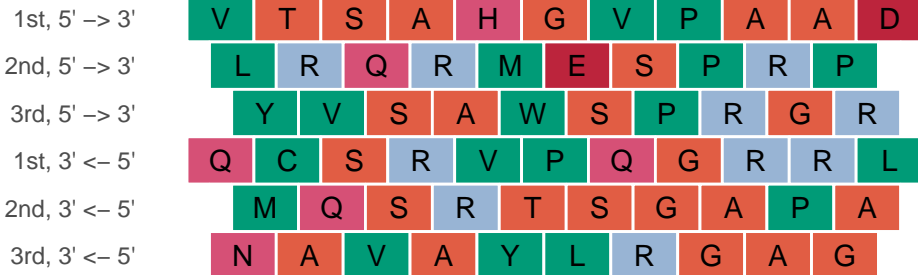

[%]

0 25 50 75 100

Match 36

Edited 59

F 5

amplicon

1

2

3

4

5

6

7

8

9

10

7879\_2

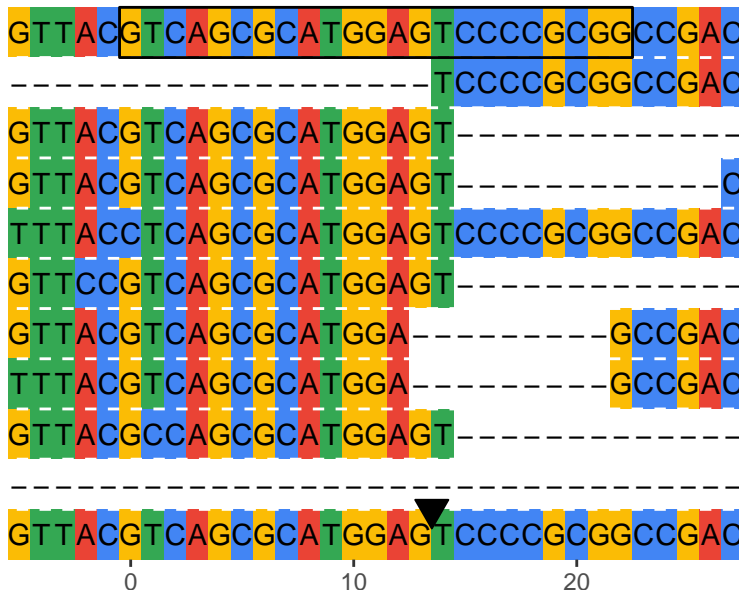

0

10

20

Relative Nucleotide Position

| Freq | Count | F    |
|------|-------|------|
| 0.29 | 91    | 0    |
| 0.3  | 92    | -120 |
| 0.1  | 32    | -15  |
| 0.04 | 11    | -12  |
| 0.03 | 8     | 0    |
| 0.02 | 6     | -15  |
| 0.02 | 6     | -9   |
| 0.02 | 5     | -9   |
| 0.01 | 4     | -15  |
| 0.01 | 4     | -136 |
| 0.01 | 4     | 1    |

Supplement: Supplemental Material [file supp_gr.244293.118_Supplemental_Code_S1.zip › amplican_manuscript/figures/normalization/MiSeq_run10_2014_05_16/7879_2_raw.pdf]

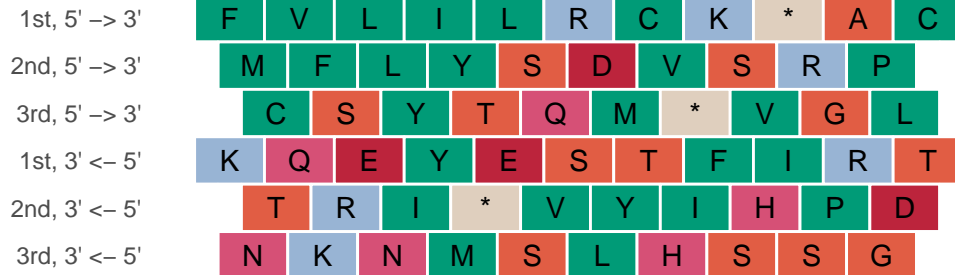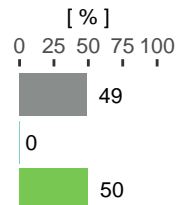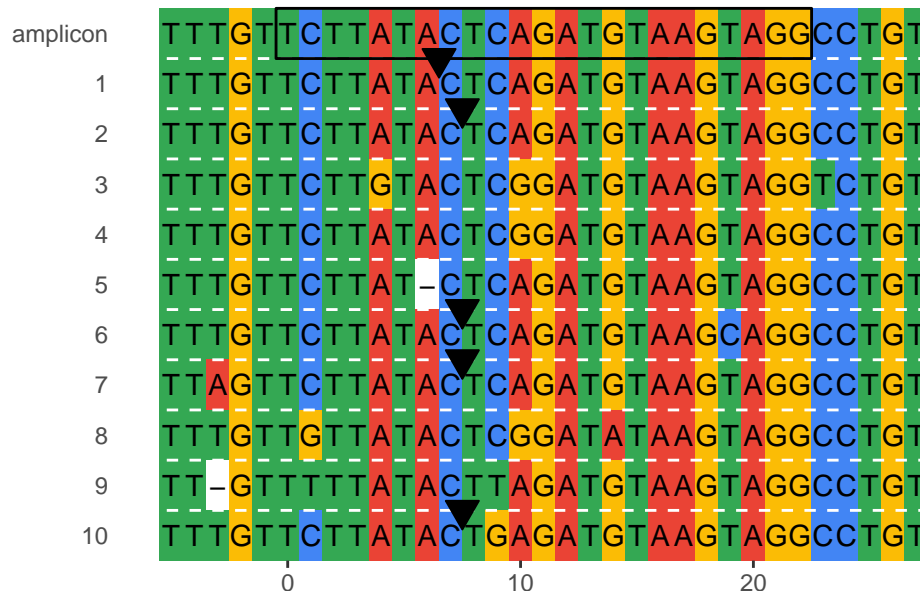

| Freq | Count | F  |
|------|-------|----|
| 0.27 | 449   | 0  |
| 0.23 | 373   | 1  |
| 0.14 | 236   | 1  |
| 0.11 | 173   | 0  |
| 0.06 | 101   | 0  |
| 0.04 | 61    | -1 |
| 0.02 | 33    | 1  |
| 0.01 | 23    | 1  |
| 0.01 | 22    | 0  |
| 0.01 | 22    | -1 |
| 0.01 | 16    | 1  |

Supplement: Supplemental Material [file supp_gr.244293.118_Supplemental_Code_S1.zip › amplican_manuscript/figures/normalization/MiSeq_run5_2013_09_25/Injected_NC24_mboatSall3_control.pdf]

Frame

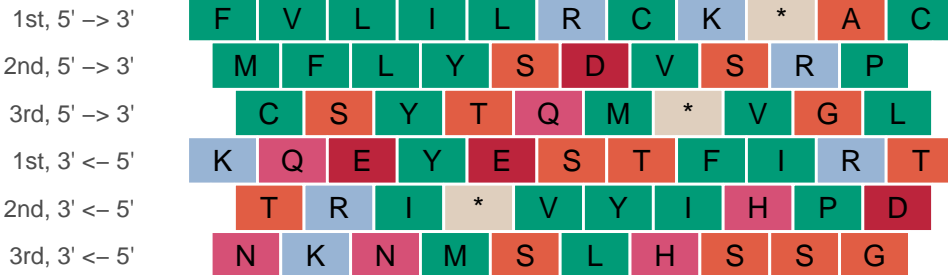

[ % ]

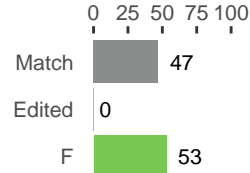

Injected\_NC24\_mboatSall3

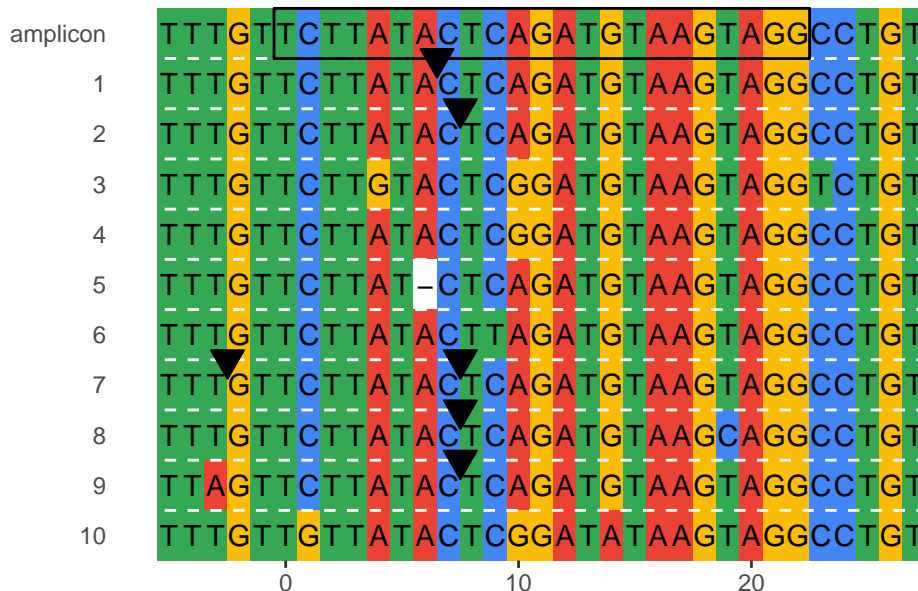

Relative Nucleotide Position

| Freq | Count | F  |
|------|-------|----|
| 0.23 | 317   | 0  |
| 0.24 | 328   | 1  |
| 0.14 | 192   | 1  |
| 0.1  | 131   | 0  |
| 0.06 | 81    | 0  |
| 0.04 | 57    | -1 |
| 0.04 | 50    | 0  |
| 0.02 | 26    | 2  |
| 0.02 | 24    | 1  |
| 0.02 | 23    | 1  |
| 0.01 | 14    | 0  |

Supplement: Supplemental Material [file supp_gr.244293.118_Supplemental_Code_S1.zip › amplican_manuscript/figures/normalization/MiSeq_run5_2013_09_25/Injected_NC24_mboatSall3_raw.pdf]

Frame

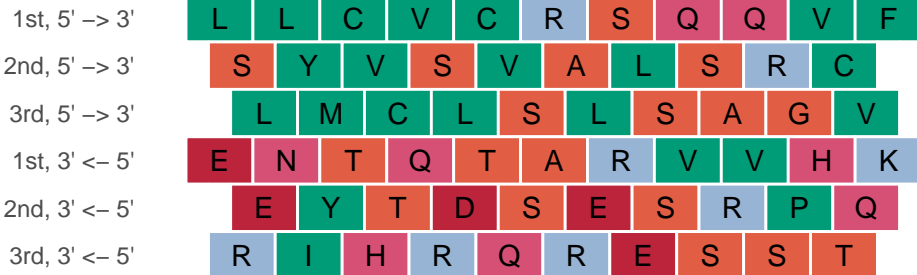

[%]

0 25 50 75 100

Match

99

Edited

1

F

1

amplicon

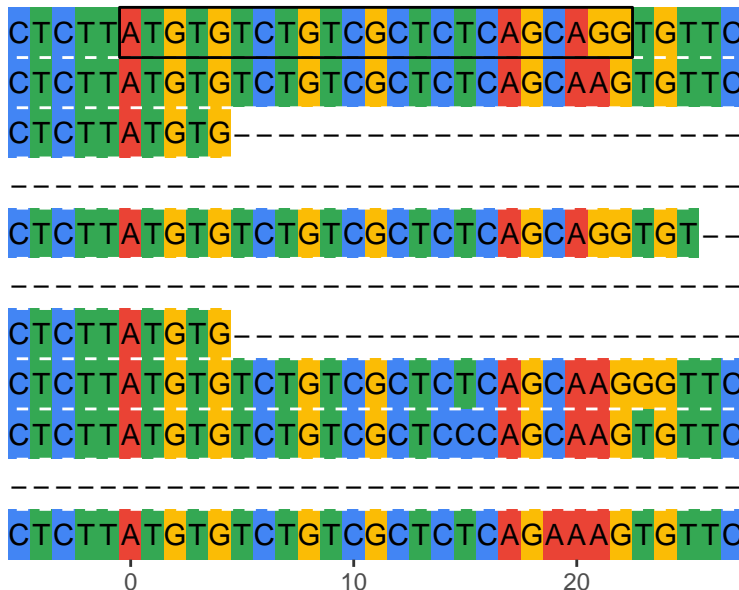

Freq

Count

F

|      |      |      |
|------|------|------|
| 0.23 | 439  | 0    |
| 0.75 | 1439 | 0    |
| 0    | 8    | -108 |
| 0    | 6    | -137 |
| 0    | 4    | -88  |
| 0    | 3    | -123 |
| 0    | 3    | -107 |
| 0    | 2    | 0    |
| 0    | 2    | 0    |
| 0    | 2    | -37  |
| 0    | 1    | 0    |

Supplement: Supplemental Material [file supp_gr.244293.118_Supplemental_Code_S1.zip › amplican_manuscript/figures/normalization/MiSeq_run5_2013_09_25/Injected_Toddler_u1_1_raw.pdf]

elov16\_e1-1-2\_7071\_uninj

1st, 5' → 3'  
2nd, 5' → 3'  
3rd, 5' → 3'  
1st, 3' ← 5'  
2nd, 3' ← 5'  
3rd, 3' ← 5'

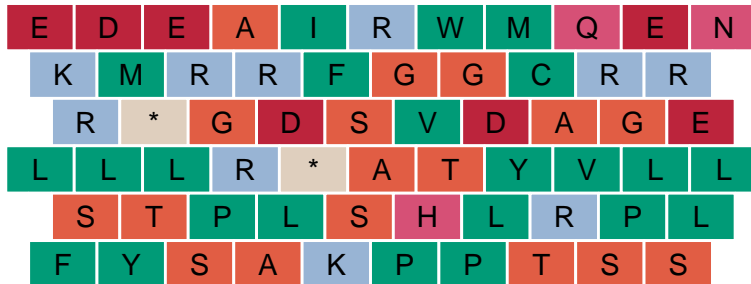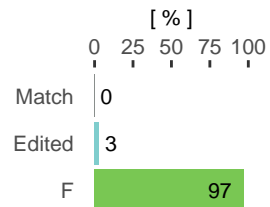

amplicon

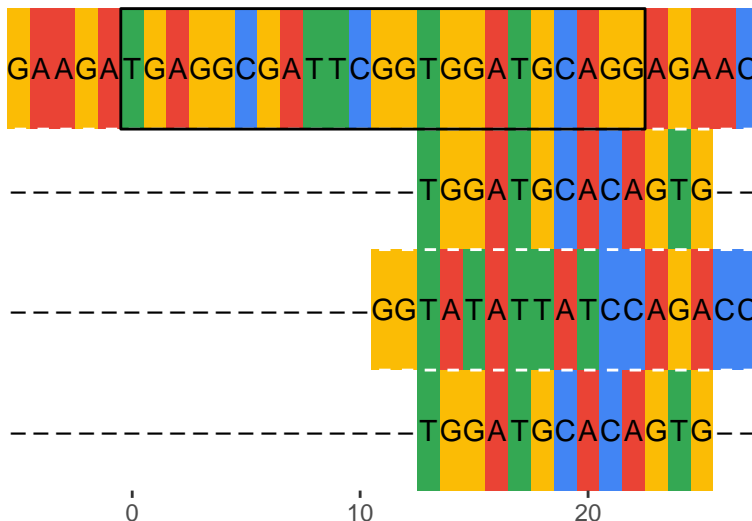

| Freq | Count | F   |
|------|-------|-----|
| 0    | 0     | 0   |
| 0.93 | 28    | -64 |
| 0.03 | 1     | -49 |
| 0.03 | 1     | -39 |

Relative Nucleotide Position

Supplement: Supplemental Material [file supp_gr.244293.118_Supplemental_Code_S1.zip › amplican_manuscript/figures/normalization/MiSeq_run6_2013_11_19/elovl6_e1-1-2_7071_inj_control.pdf]

elov6\_e1-1-2\_7071\_inj

1st, 5' → 3'

2nd, 5' → 3'

3rd, 5' → 3'

1st, 3' ← 5'

2nd, 3' ← 5'

3rd, 3' ← 5'

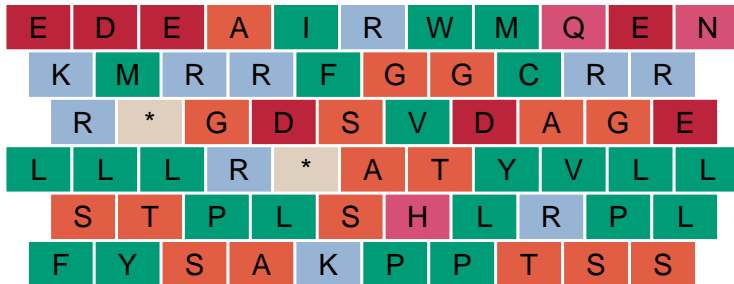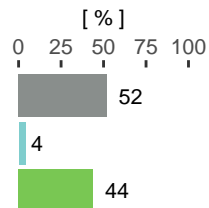

amplicon

1

2

3

4

5

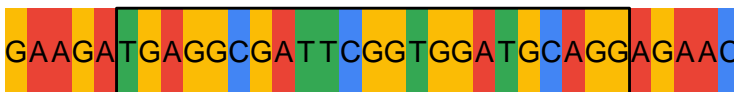

0

10

20

Relative Nucleotide Position

| Freq | Count | F    |
|------|-------|------|
| 0.52 | 13    | 0    |
| 0.28 | 7     | -218 |
| 0.08 | 2     | -151 |
| 0.04 | 1     | -109 |
| 0.04 | 1     | -51  |
| 0.04 | 1     | -100 |

Supplement: Supplemental Material [file supp_gr.244293.118_Supplemental_Code_S1.zip › amplican_manuscript/figures/normalization/MiSeq_run6_2013_11_19/elovl6_e1-1-2_7071_inj_normalized.pdf]

Frame

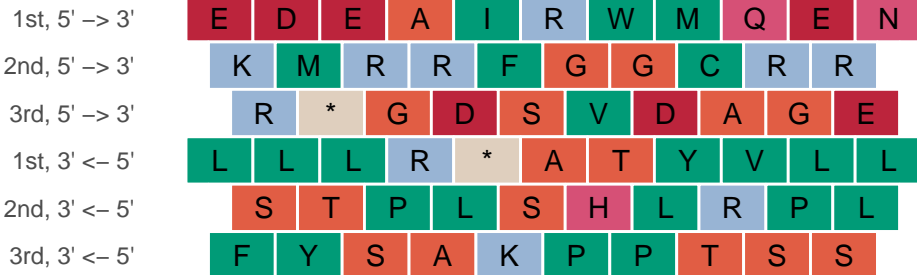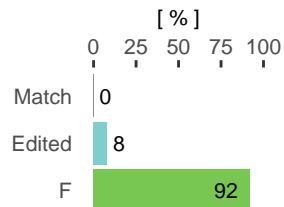

amplicon

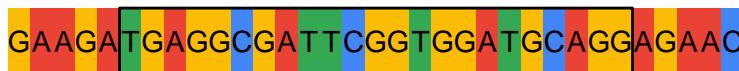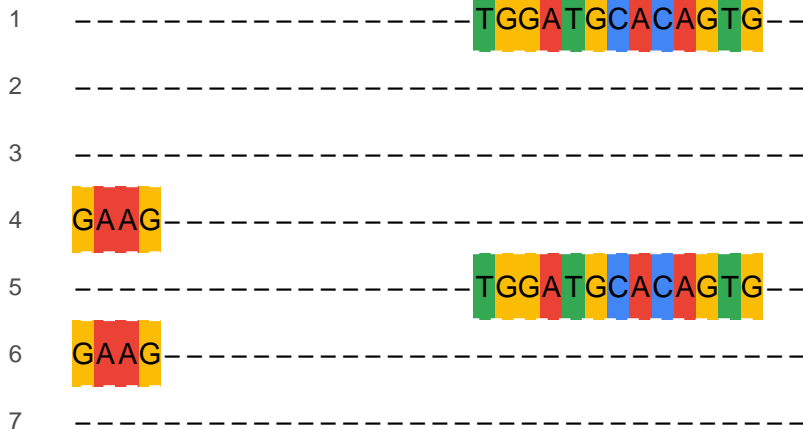

0

10

20

Relative Nucleotide Position

| Freq | Count | F    |
|------|-------|------|
| 0    | 0     | 0    |
| 0.48 | 12    | -64  |
| 0.28 | 7     | -218 |
| 0.08 | 2     | -151 |
| 0.04 | 1     | -109 |
| 0.04 | 1     | -39  |
| 0.04 | 1     | -51  |
| 0.04 | 1     | -100 |

elovl6\_e1-1-2\_7071\_inj

Supplement: Supplemental Material [file supp_gr.244293.118_Supplemental_Code_S1.zip › amplican_manuscript/figures/normalization/MiSeq_run6_2013_11_19/elovl6_e1-1-2_7071_inj_raw.pdf]

insert\_ctgfa\_my96ds\_3

1st, 5' → 3'  
2nd, 5' → 3'  
3rd, 5' → 3'  
1st, 3' ← 5'  
2nd, 3' ← 5'  
3rd, 3' ← 5'

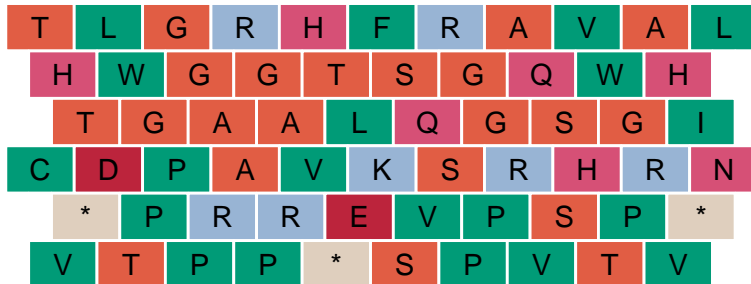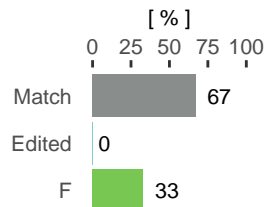

amplicon

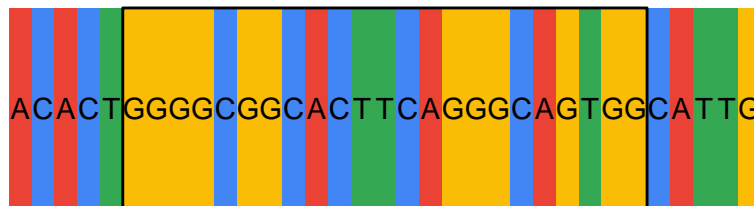

1

0

10

20

Relative Nucleotide Position

Freq Count F

0.67 2 0

0.33 1 -70

Supplement: Supplemental Material [file supp_gr.244293.118_Supplemental_Code_S1.zip › amplican_manuscript/figures/normalization/MiSeq_run6_2013_11_19/insert_ctgfa_my96ds_3_normalized.pdf]

Frame

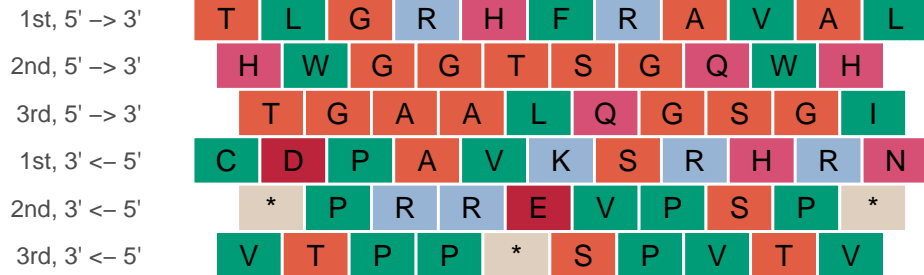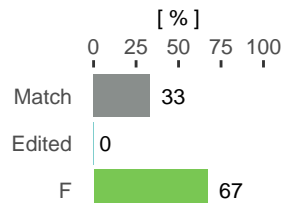

insert\_ctgfa\_my96ds\_3

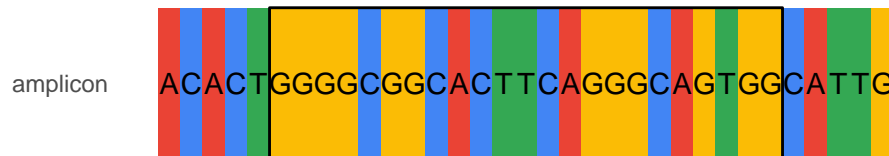

1

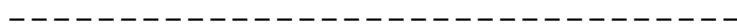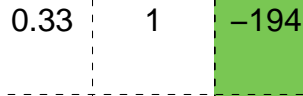

2

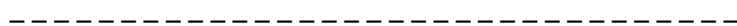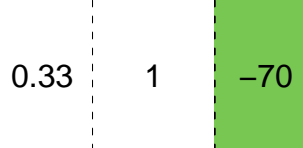

0

10

20

Relative Nucleotide Position

Supplement: Supplemental Material [file supp_gr.244293.118_Supplemental_Code_S1.zip › amplican_manuscript/figures/normalization/MiSeq_run6_2013_11_19/insert_ctgfa_my96ds_3_raw.pdf]

Frame

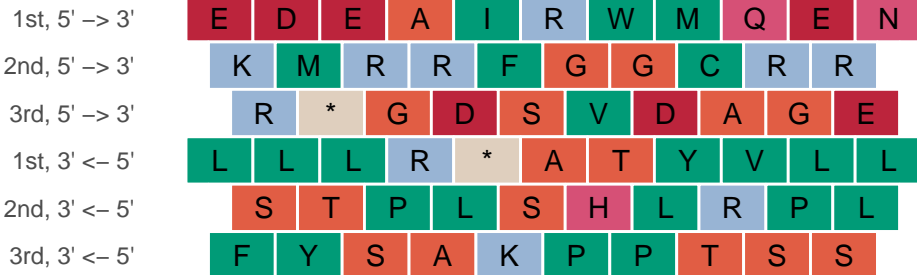

amplicon

1

2

3

4

5

6

7

8

9

10

elov6\_e1\_1-2\_inj

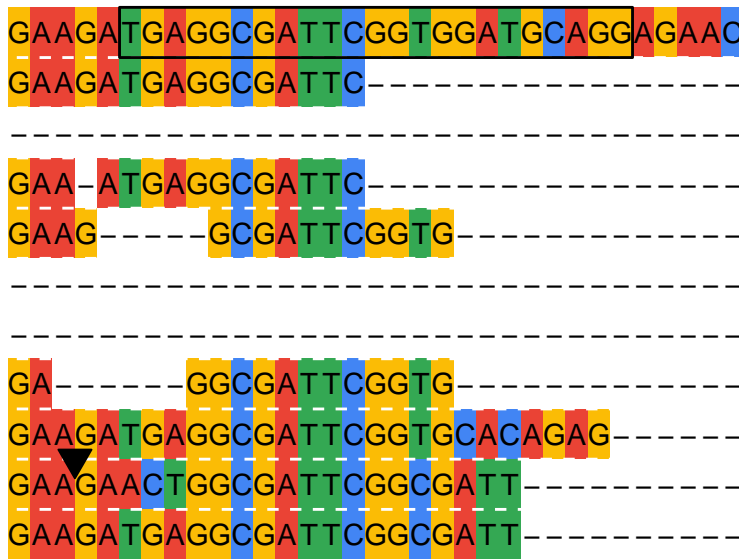

0

10

20

Relative Nucleotide Position

[%]

0 25 50 75 100

Match

82

Edited

4

F

14

| Freq | Count | F    |
|------|-------|------|
| 0.82 | 150   | 0    |
| 0.03 | 5     | -77  |
| 0.02 | 4     | -100 |
| 0.02 | 3     | -78  |
| 0.01 | 2     | -97  |
| 0.01 | 2     | -86  |
| 0.01 | 2     | -107 |
| 0.01 | 2     | -67  |
| 0.01 | 2     | -6   |
| 0.01 | 1     | -75  |
| 0.01 | 1     | -77  |

Supplement: Supplemental Material [file supp_gr.244293.118_Supplemental_Code_S1.zip › amplican_manuscript/figures/normalization/MiSeq_run7_2014_01_02/elovl6_e1_1-2_inj_normalized.pdf]

Frame

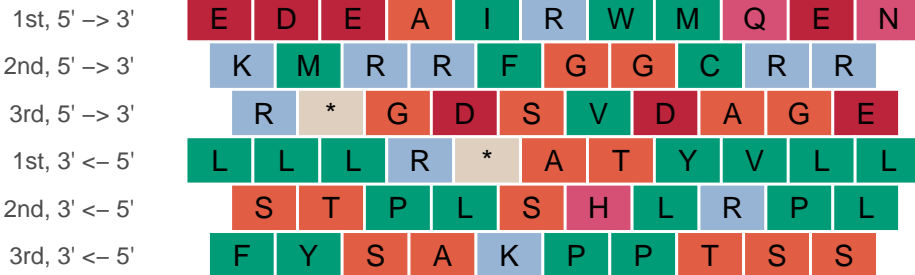

[%]

0 25 50 75 100

Match

0

Edited

9

F

91

amplicon

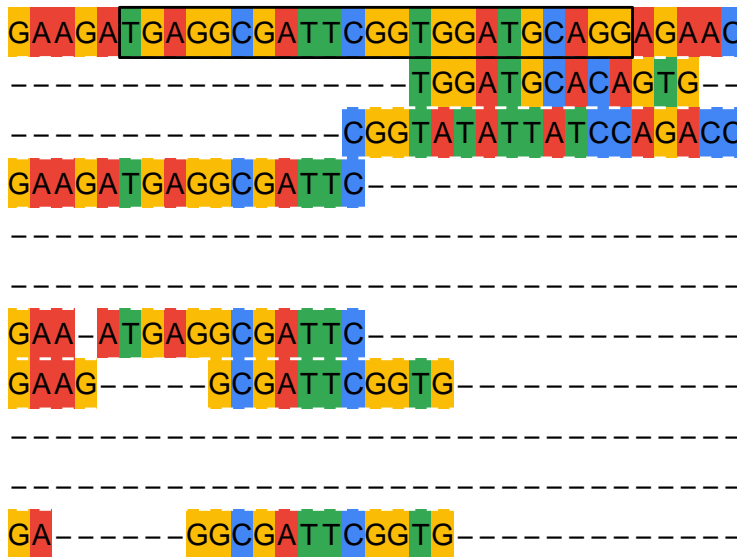

Freq

Count

F

0

0

0

0.58

106

-64

0.2

37

-113

0.03

5

-77

0.03

5

-204

0.02

4

-100

0.02

3

-78

0.01

2

-97

0.01

2

-86

0.01

2

-107

0.01

2

-67

0

10

20

Relative Nucleotide Position

elov16\_e1\_1-2\_inj

Supplement: Supplemental Material [file supp_gr.244293.118_Supplemental_Code_S1.zip › amplican_manuscript/figures/normalization/MiSeq_run7_2014_01_02/elovl6_e1_1-2_inj_raw.pdf]

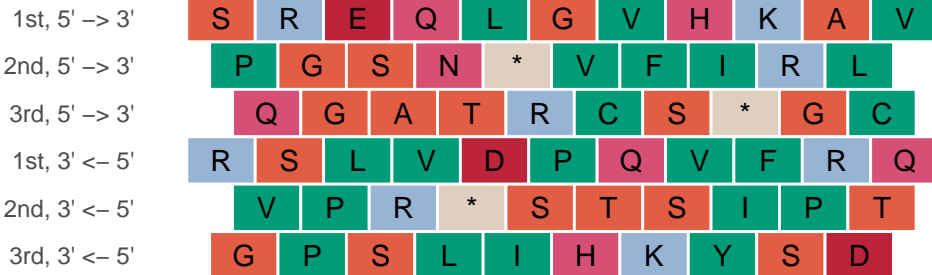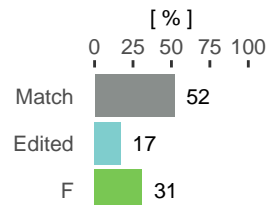

amplicon

1

2

3

4

5

6

7

8

9

10

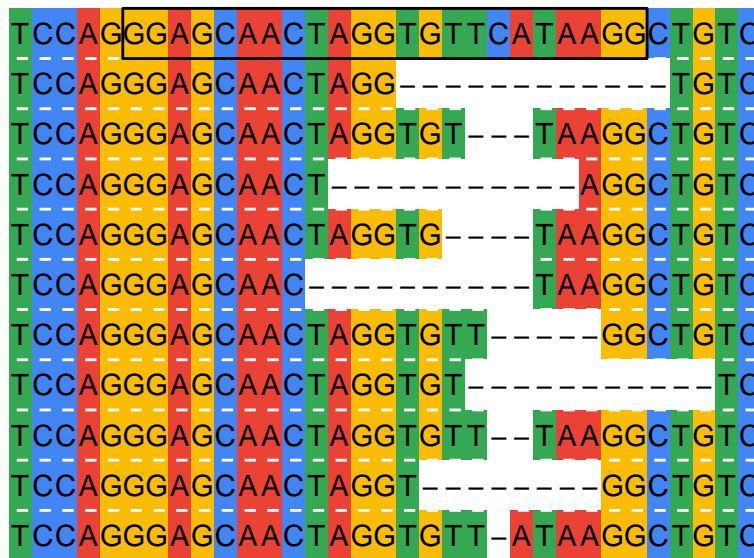

0

10

20

Relative Nucleotide Position

| Freq | Count | F   |
|------|-------|-----|
| 0.49 | 916   | 0   |
| 0.04 | 76    | -12 |
| 0.04 | 73    | -3  |
| 0.04 | 69    | -11 |
| 0.02 | 35    | -4  |
| 0.01 | 28    | -10 |
| 0.01 | 26    | -5  |
| 0.01 | 23    | -11 |
| 0.01 | 20    | -2  |
| 0.01 | 19    | -8  |
| 0.01 | 17    | -1  |

Supplement: Supplemental Material [file supp_gr.244293.118_Supplemental_Code_S1.zip › amplican_manuscript/figures/normalization/MiSeq_run7_2014_01_02/spaw-lnc_e1_1_inj_normalized.pdf]

spaw-inc\_e1\_1\_inj

1st, 5' → 3'

2nd, 5' → 3'

3rd, 5' → 3'

1st, 3' ← 5'

2nd, 3' ← 5'

3rd, 3' ← 5'

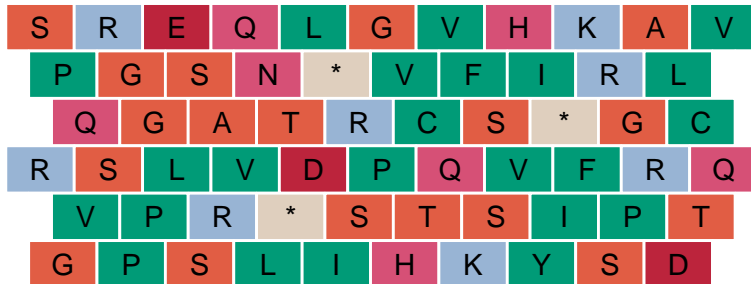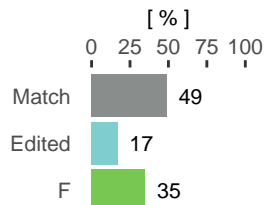

amplicon

1

2

3

4

5

6

7

8

9

10

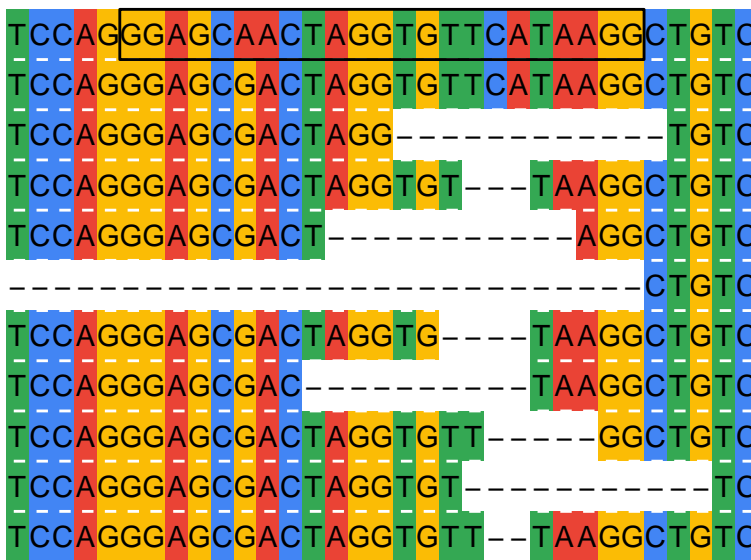

0

10

20

Relative Nucleotide Position

| Freq | Count | F   |
|------|-------|-----|
| 0.07 | 127   | 0   |
| 0.39 | 728   | 0   |
| 0.04 | 76    | -12 |
| 0.04 | 73    | -3  |
| 0.04 | 69    | -11 |
| 0.03 | 61    | -58 |
| 0.02 | 35    | -4  |
| 0.01 | 28    | -10 |
| 0.01 | 26    | -5  |
| 0.01 | 23    | -11 |
| 0.01 | 20    | -2  |

Supplement: Supplemental Material [file supp_gr.244293.118_Supplemental_Code_S1.zip › amplican_manuscript/figures/normalization/MiSeq_run7_2014_01_02/spaw-lnc_e1_1_inj_raw.pdf]

Frame

1st, 5' → 3'

2nd, 5' → 3'

3rd, 5' → 3'

1st, 3' ← 5'

2nd, 3' ← 5'

3rd, 3' ← 5'

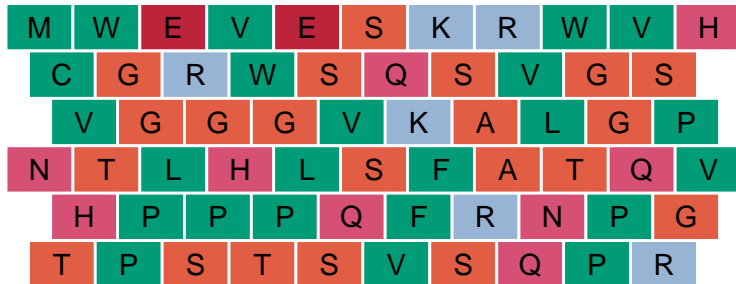[%]  
0 25 50 75 100

Match

62

Edited

9

F

28

amplicon

1

2

3

4

5

6

7

8

9

10

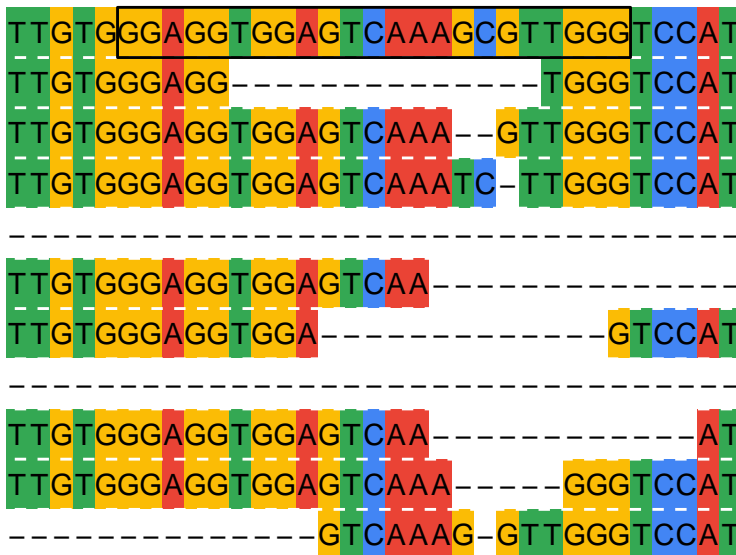

0

10

20

Relative Nucleotide Position

Freq

Count

F

0.6

3693

0

0.03

186

-14

0.03

177

-2

0.03

167

-1

0.03

154

-149

0.02

141

-14

0.02

95

-13

0.01

91

-87

0.01

86

-12

0.01

69

-5

0.01

69

-16

Supplement: Supplemental Material [file supp_gr.244293.118_Supplemental_Code_S1.zip › amplican_manuscript/figures/normalization/MiSeq_run8_2014_01_30/atp1a1.1_e4_1_inj_control.pdf]

atp1a1.1\_e4\_1\_inj

1st, 5' → 3'  
2nd, 5' → 3'  
3rd, 5' → 3'  
1st, 3' ← 5'  
2nd, 3' ← 5'  
3rd, 3' ← 5'

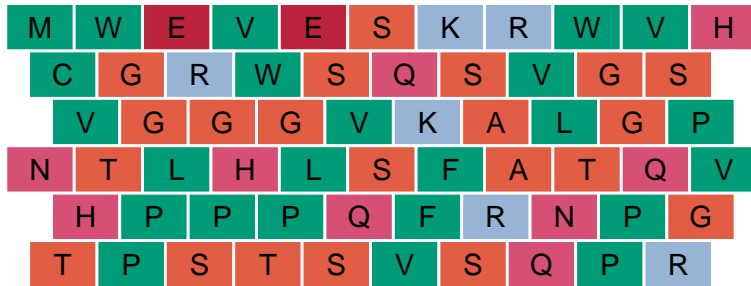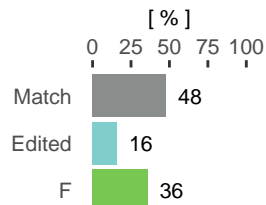

amplicon

1  
2  
3  
4  
5  
6  
7  
8  
9  
10

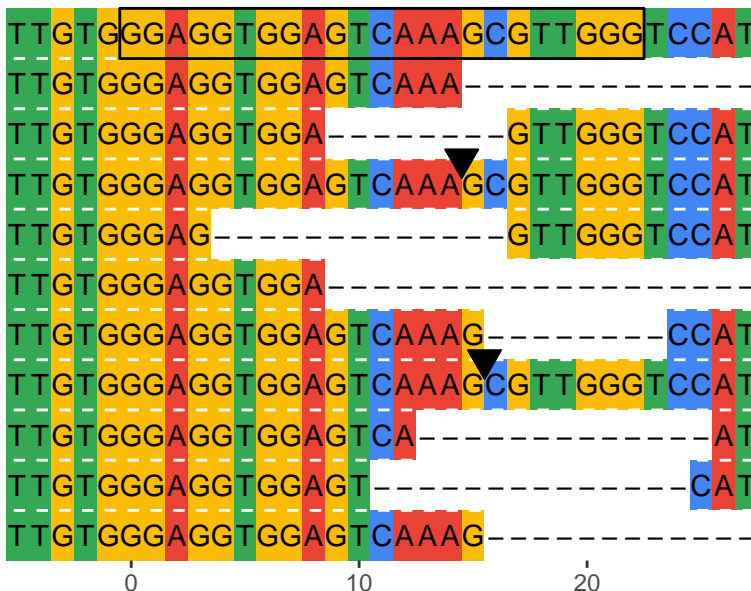

| Freq | Count | F   |
|------|-------|-----|
| 0.45 | 1813  | 0   |
| 0.07 | 271   | -16 |
| 0.03 | 110   | -8  |
| 0.02 | 85    | 1   |
| 0.02 | 70    | -13 |
| 0.02 | 63    | -30 |
| 0.02 | 62    | -8  |
| 0.01 | 59    | 3   |
| 0.01 | 58    | -13 |
| 0.01 | 52    | -14 |
| 0.01 | 45    | -12 |

Relative Nucleotide Position

Supplement: Supplemental Material [file supp_gr.244293.118_Supplemental_Code_S1.zip › amplican_manuscript/figures/normalization/MiSeq_run8_2014_01_30/atp1a1.1_e4_1_inj_normalized.pdf]

atp1a1.1\_e4\_1\_inj

1st, 5' → 3'  
2nd, 5' → 3'  
3rd, 5' → 3'  
1st, 3' ← 5'  
2nd, 3' ← 5'  
3rd, 3' ← 5'

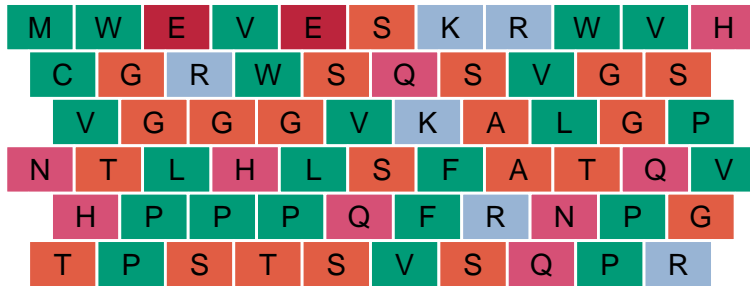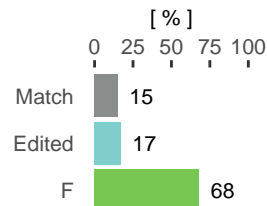

amplicon

1  
2  
3  
4  
5  
6  
7  
8  
9  
10

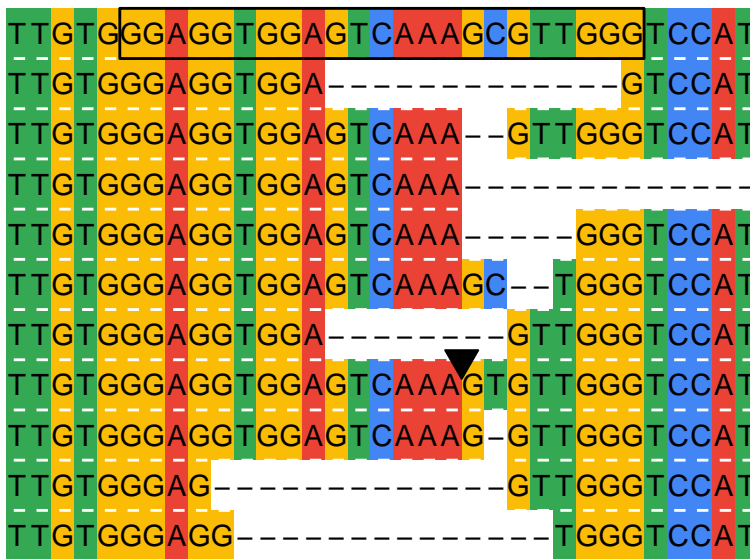

0

10

20

Relative Nucleotide Position

| Freq | Count | F   |
|------|-------|-----|
| 0.13 | 525   | 0   |
| 0.12 | 474   | -13 |
| 0.07 | 301   | -2  |
| 0.07 | 271   | -16 |
| 0.04 | 162   | -5  |
| 0.03 | 114   | -2  |
| 0.03 | 110   | -8  |
| 0.02 | 85    | 1   |
| 0.02 | 79    | -1  |
| 0.02 | 70    | -13 |
| 0.02 | 64    | -14 |

Supplement: Supplemental Material [file supp_gr.244293.118_Supplemental_Code_S1.zip › amplican_manuscript/figures/normalization/MiSeq_run8_2014_01_30/atp1a1.1_e4_1_inj_raw.pdf]

Frame

old\_g118\_1

1st, 5' → 3'

2nd, 5' → 3'

3rd, 5' → 3'

1st, 3' ← 5'

2nd, 3' ← 5'

3rd, 3' ← 5'

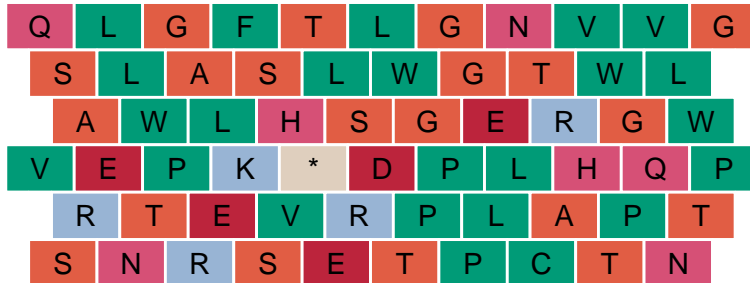

[ % ]

0 25 50 75 100

Match

90

Edited

6

F

4

amplicon

1

2

3

4

5

6

7

8

9

10

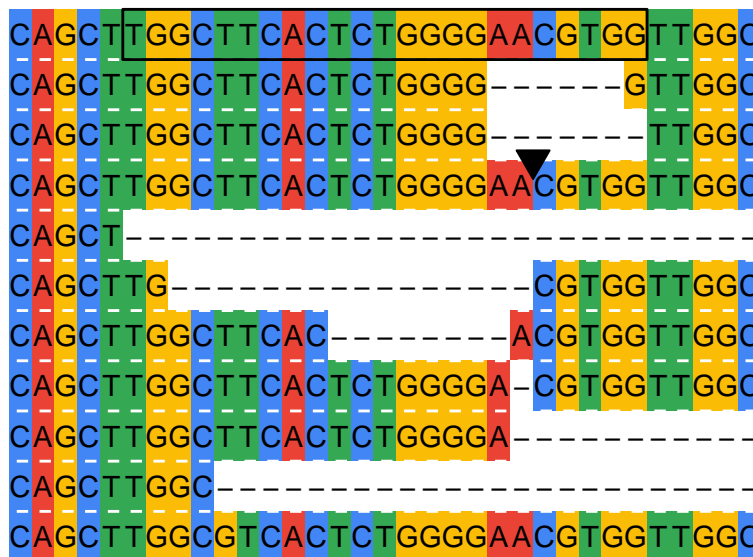

0

10

20

Relative Nucleotide Position

| Freq | Count | F   |
|------|-------|-----|
| 0.88 | 434   | 0   |
| 0.04 | 22    | -6  |
| 0.01 | 3     | -7  |
| 0.01 | 3     | 1   |
| 0    | 2     | -40 |
| 0    | 2     | -16 |
| 0    | 2     | -8  |
| 0    | 2     | -1  |
| 0    | 2     | -12 |
| 0    | 1     | -24 |
| 0    | 1     | 0   |

Supplement: Supplemental Material [file supp_gr.244293.118_Supplemental_Code_S1.zip › amplican_manuscript/figures/normalization/MiSeq_run8_2014_01_30/old_g118_1_normalized.pdf]

Frame

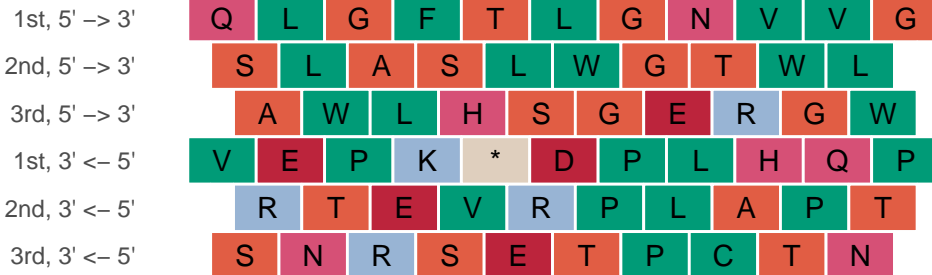[ % ]  
0 25 50 75 100

Match 89

Edited 7

F 4

amplicon

1

2

3

4

5

6

7

8

9

10

old\_g118\_1

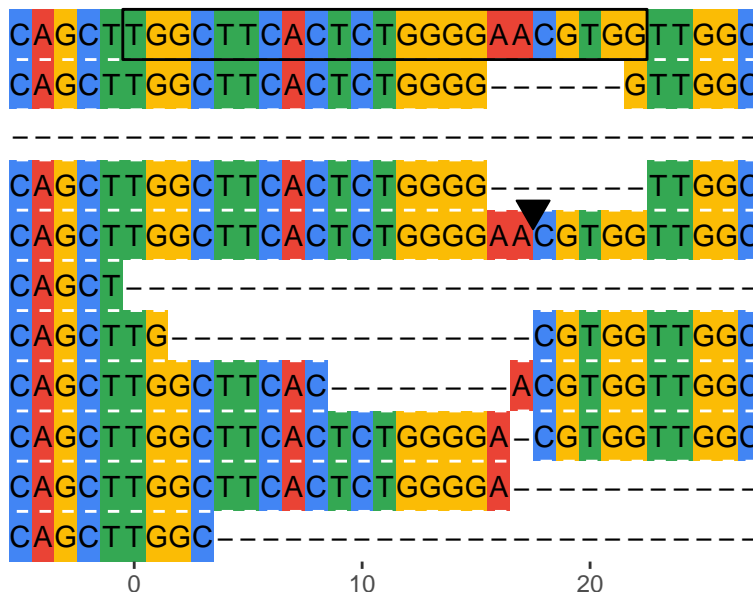

| Freq | Count | F   |
|------|-------|-----|
| 0.88 | 430   | 0   |
| 0.04 | 22    | -6  |
| 0.01 | 4     | -51 |
| 0.01 | 3     | -7  |
| 0.01 | 3     | 1   |
| 0    | 2     | -40 |
| 0    | 2     | -16 |
| 0    | 2     | -8  |
| 0    | 2     | -1  |
| 0    | 2     | -12 |
| 0    | 1     | -24 |

Supplement: Supplemental Material [file supp_gr.244293.118_Supplemental_Code_S1.zip › amplican_manuscript/figures/normalization/MiSeq_run8_2014_01_30/old_g118_1_raw.pdf]

Frame

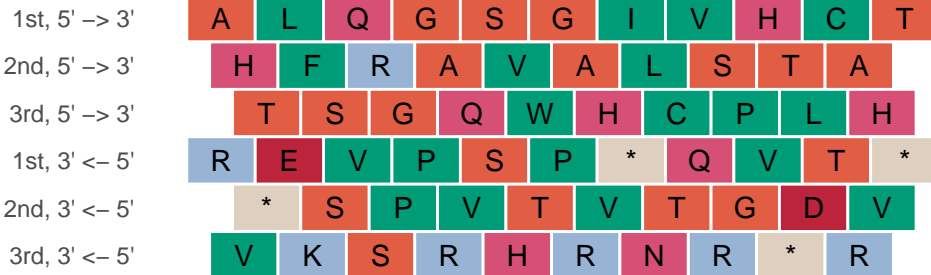[ % ]  
0 25 50 75 100

Match 93

Edited 2

F 5

amplicon

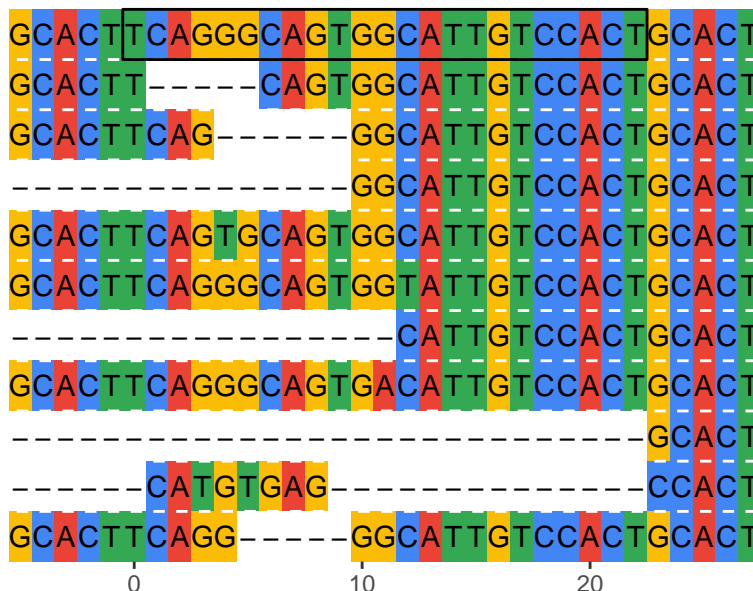

Relative Nucleotide Position

| Freq | Count | F   |
|------|-------|-----|
| 0.9  | 971   | 0   |
| 0.01 | 14    | -5  |
| 0.01 | 7     | -6  |
| 0.01 | 7     | -31 |
| 0    | 4     | 0   |
| 0    | 4     | 0   |
| 0    | 4     | -27 |
| 0    | 3     | 0   |
| 0    | 3     | -28 |
| 0    | 3     | -21 |
| 0    | 2     | -5  |

Supplement: Supplemental Material [file supp_gr.244293.118_Supplemental_Code_S1.zip › amplican_manuscript/figures/normalization/MiSeq_run9_2014_03_26/213ds_control.pdf]

Frame

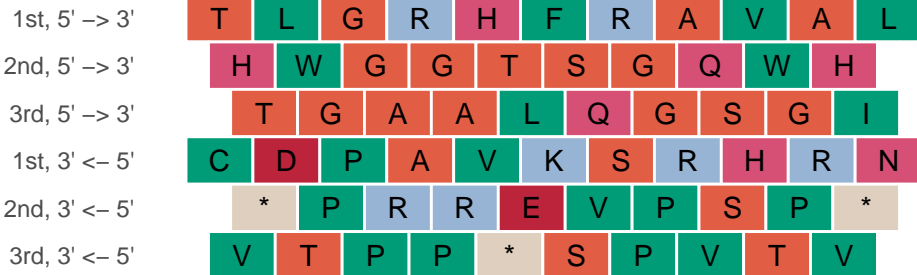

[%]

0 25 50 75 100

Match

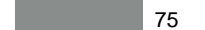

Edited

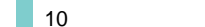

F 16

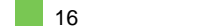

amplicon

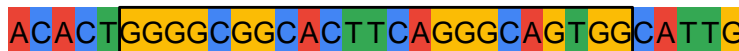

1

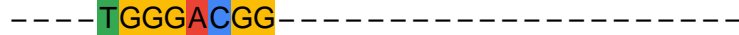

2

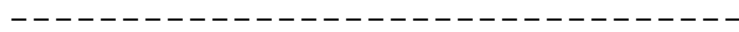

3

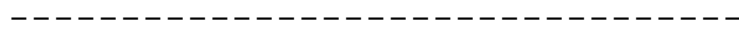

4

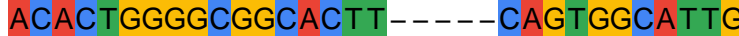

5

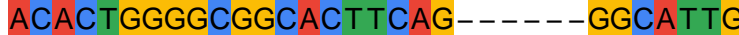

6

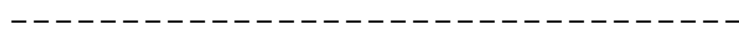

7

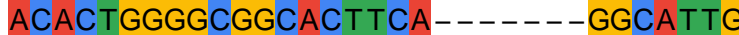

8

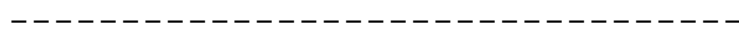

9

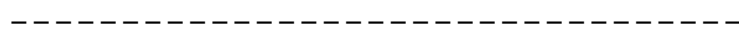

10

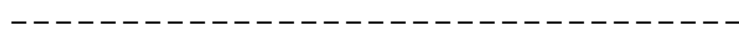

0

10

20

Relative Nucleotide Position

Freq

Count

F

0.74

6099

0

0.06

509

-79

0.03

215

-183

0.02

169

-141

0.01

100

-5

0.01

55

-6

0

25

-192

0

18

-7

0

17

-137

0

16

-176

0

15

-169

Supplement: Supplemental Material [file supp_gr.244293.118_Supplemental_Code_S1.zip › amplican_manuscript/figures/normalization/MiSeq_run9_2014_03_26/213ds_normalized.pdf]

Frame

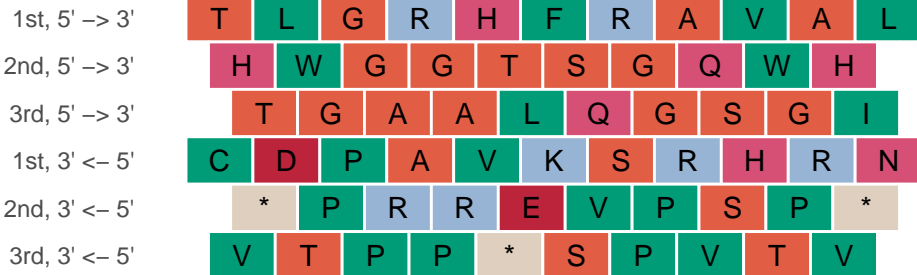

[%]

0 25 50 75 100

Match

2

Edited

28

F

70

213ds

1

2

3

4

5

6

7

8

9

10

Freq

Count

F

0.02

144

0

0.46

3802

-194

0.18

1459

-111

0.06

509

-79

0.03

215

-183

0.02

201

-182

0.02

176

-188

0.02

169

-141

0.02

142

-155

0.01

114

-154

0.01

100

-5

0

10

20

Relative Nucleotide Position

Supplement: Supplemental Material [file supp_gr.244293.118_Supplemental_Code_S1.zip › amplican_manuscript/figures/normalization/MiSeq_run9_2014_03_26/213ds_raw.pdf]

Frame

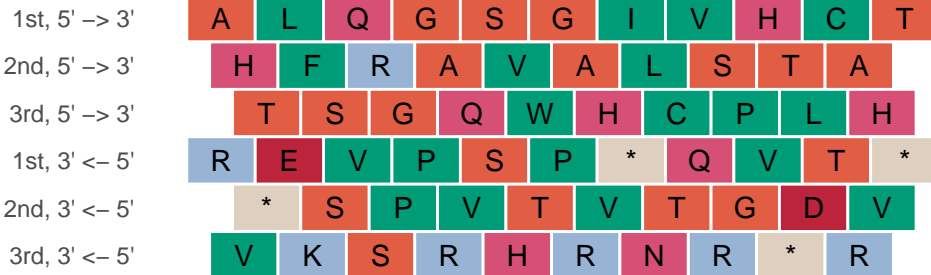

[ % ]

Match 93

Edited 2

F 5

amplicon

1

2

3

4

5

6

7

8

9

10

control1

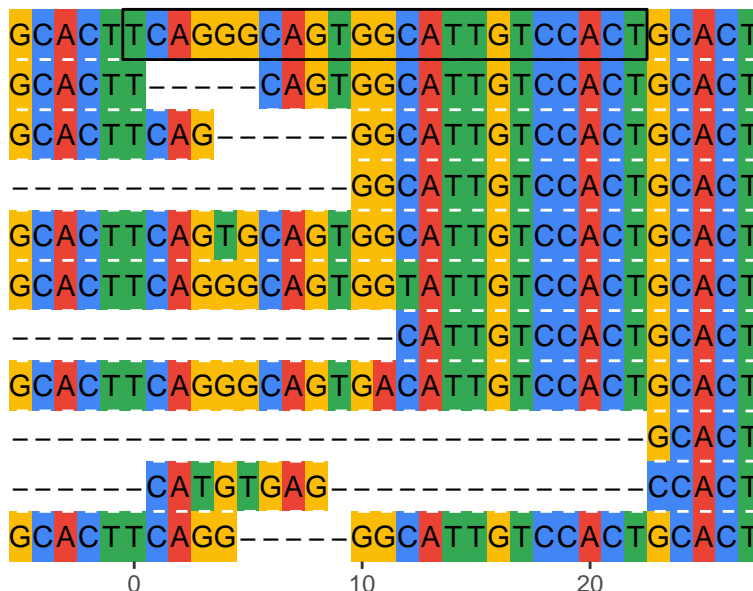

0

10

20

Relative Nucleotide Position

| Freq | Count | F   |
|------|-------|-----|
| 0.9  | 971   | 0   |
| 0.01 | 14    | -5  |
| 0.01 | 7     | -6  |
| 0.01 | 7     | -31 |
| 0    | 4     | 0   |
| 0    | 4     | 0   |
| 0    | 4     | -27 |
| 0    | 3     | 0   |
| 0    | 3     | -28 |
| 0    | 3     | -21 |
| 0    | 2     | -5  |

Supplement: Supplemental Material [file supp_gr.244293.118_Supplemental_Code_S1.zip › amplican_manuscript/figures/normalization/MiSeq_run9_2014_03_26/213ss_control.pdf]

Frame

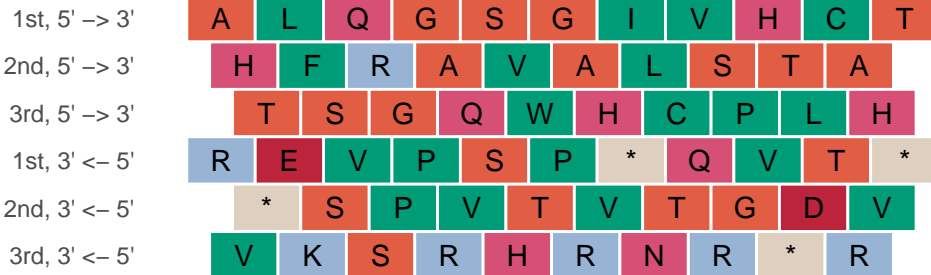

[ % ]

0 25 50 75 100

Match 93

Edited 2

F 5

control1

amplicon

1

2

3

4

5

6

7

8

9

10

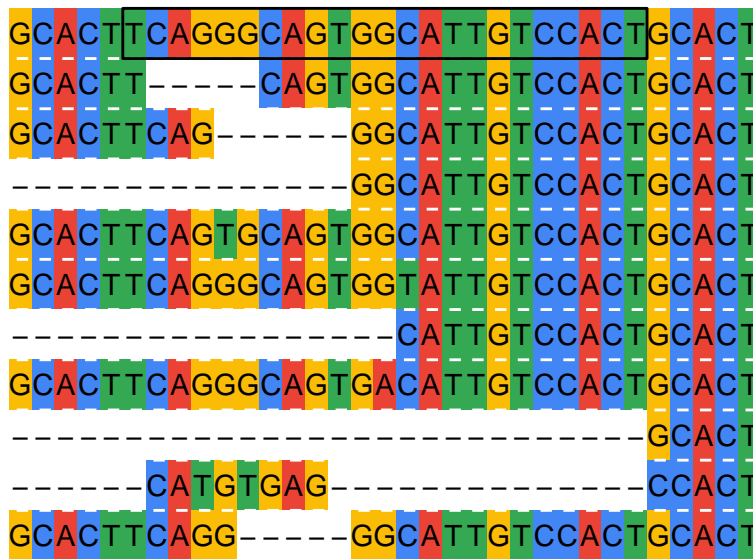

0

10

20

Relative Nucleotide Position

| Freq | Count | F   |
|------|-------|-----|
| 0.9  | 971   | 0   |
| 0.01 | 14    | -5  |
| 0.01 | 7     | -6  |
| 0.01 | 7     | -31 |
| 0    | 4     | 0   |
| 0    | 4     | 0   |
| 0    | 4     | -27 |
| 0    | 3     | 0   |
| 0    | 3     | -28 |
| 0    | 3     | -21 |
| 0    | 2     | -5  |

Supplement: Supplemental Material [file supp_gr.244293.118_Supplemental_Code_S1.zip › amplican_manuscript/figures/normalization/MiSeq_run9_2014_03_26/GFPnoSTOP_control.pdf]

Frame

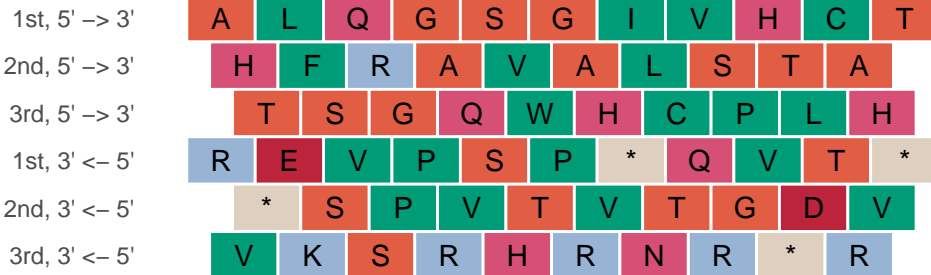[ % ]  
0 25 50 75 100

Match 93

Edited 2

F 5

amplicon

1

2

3

4

5

6

7

8

9

10

control1

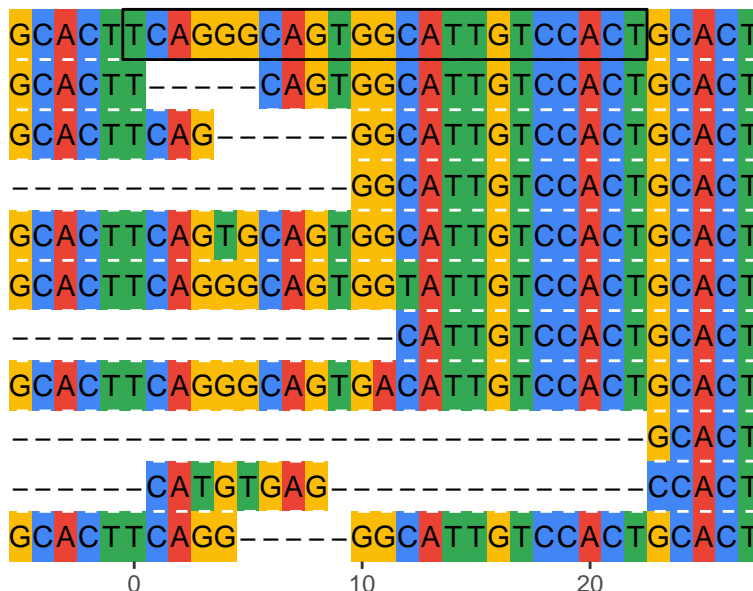

| Freq | Count | F   |
|------|-------|-----|
| 0.9  | 971   | 0   |
| 0.01 | 14    | -5  |
| 0.01 | 7     | -6  |
| 0.01 | 7     | -31 |
| 0    | 4     | 0   |
| 0    | 4     | 0   |
| 0    | 4     | -27 |
| 0    | 3     | 0   |
| 0    | 3     | -28 |
| 0    | 3     | -21 |
| 0    | 2     | -5  |

Supplement: Supplemental Material [file supp_gr.244293.118_Supplemental_Code_S1.zip › amplican_manuscript/figures/normalization/MiSeq_run9_2014_03_26/GFPnoSTOP_gRNA-_control.pdf]

Frame

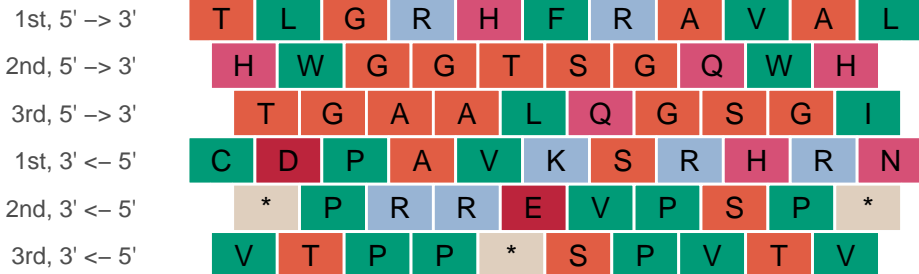

GFPnoSTOP\_gRNA--

1

2

3

4

5

6

7

8

9

10

Relative Nucleotide Position

[%]

0 25 50 75 100

Match

13

Edited

19

F

67

| Freq | Count | F    |
|------|-------|------|
| 0.13 | 752   | 0    |
| 0.62 | 3611  | -194 |
| 0.06 | 332   | -111 |
| 0.05 | 272   | -183 |
| 0.04 | 223   | -126 |
| 0.03 | 152   | -141 |
| 0.01 | 57    | -137 |
| 0.01 | 55    | -174 |
| 0.01 | 42    | -217 |
| 0    | 27    | -176 |
| 0    | 24    | -172 |

Supplement: Supplemental Material [file supp_gr.244293.118_Supplemental_Code_S1.zip › amplican_manuscript/figures/normalization/MiSeq_run9_2014_03_26/GFPnoSTOP_gRNA-_raw.pdf]

Frame

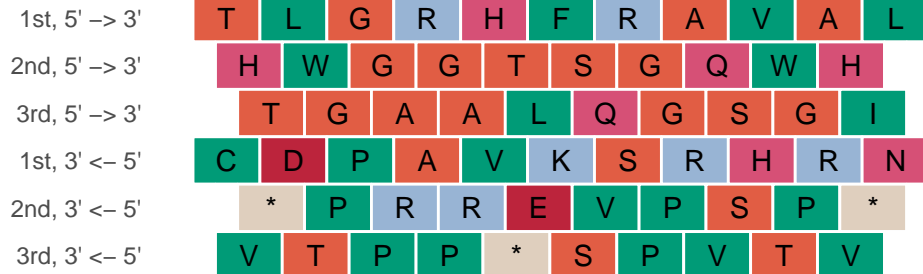

[%]

0 25 50 75 100

Match

77

Edited

9

F

14

amplicon

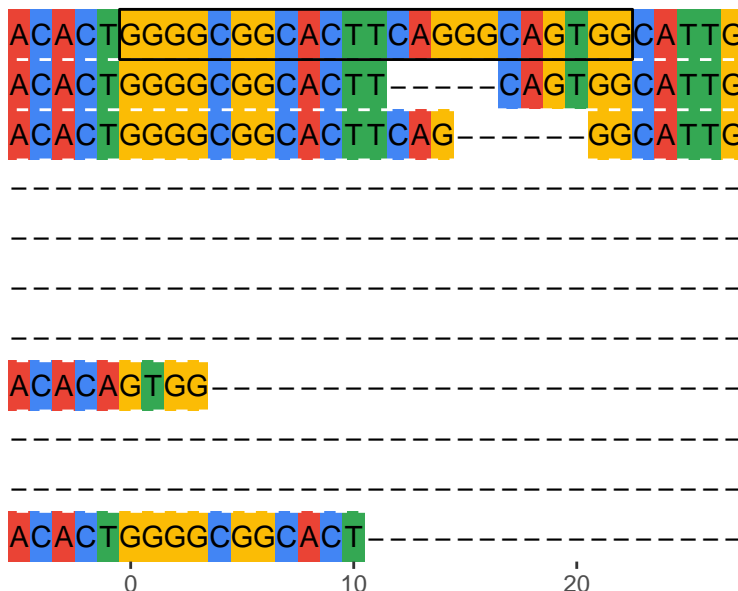

0

10

20

Relative Nucleotide Position

| Freq | Count | F    |
|------|-------|------|
| 0.77 | 2577  | 0    |
| 0.03 | 92    | -5   |
| 0.03 | 89    | -6   |
| 0.01 | 33    | -176 |
| 0.01 | 32    | -137 |
| 0.01 | 31    | -38  |
| 0.01 | 27    | -192 |
| 0.01 | 19    | -30  |
| 0.01 | 17    | -42  |
| 0    | 15    | -172 |
| 0    | 13    | -27  |

GFPnoSTOP

Supplement: Supplemental Material [file supp_gr.244293.118_Supplemental_Code_S1.zip › amplican_manuscript/figures/normalization/MiSeq_run9_2014_03_26/GFPnoSTOP_normalized.pdf]

Frame

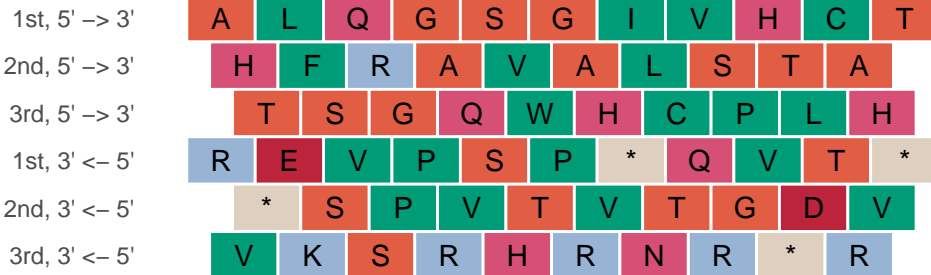

[ % ]

0 25 50 75 100

Match 93

Edited 2

F 5

amplicon

1

2

3

4

5

6

7

8

9

10

control1

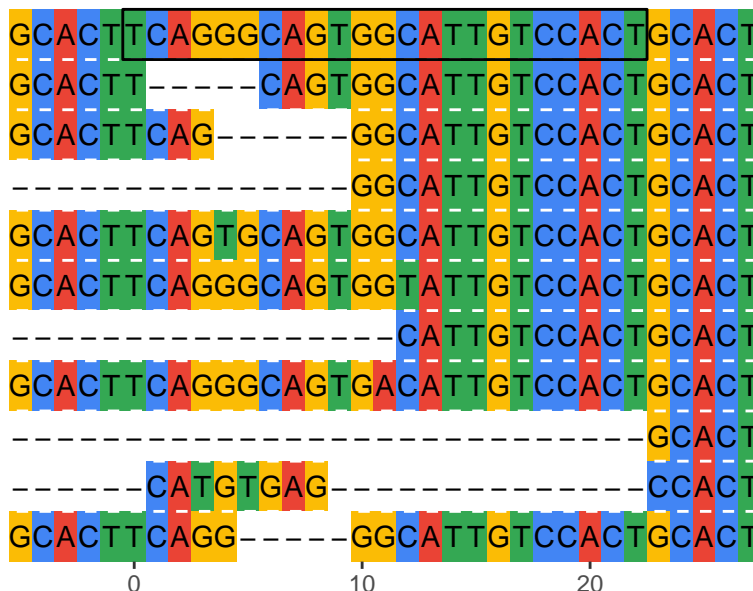

| Freq | Count | F   |
|------|-------|-----|
| 0.9  | 971   | 0   |
| 0.01 | 14    | -5  |
| 0.01 | 7     | -6  |
| 0.01 | 7     | -31 |
| 0    | 4     | 0   |
| 0    | 4     | 0   |
| 0    | 4     | -27 |
| 0    | 3     | 0   |
| 0    | 3     | -28 |
| 0    | 3     | -21 |
| 0    | 2     | -5  |

Supplement: Supplemental Material [file supp_gr.244293.118_Supplemental_Code_S1.zip › amplican_manuscript/figures/normalization/MiSeq_run9_2014_03_26/invit96ss_control.pdf]

Frame

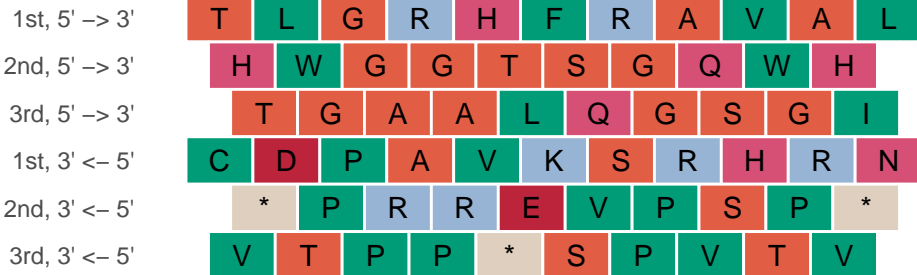

[%]

0 25 50 75 100

Match

78

Edited

9

F

13

Freq

Count

F

|      |      |      |
|------|------|------|
| 0.78 | 7508 | 0    |
| 0.03 | 305  | -183 |
| 0.02 | 201  | -112 |
| 0.01 | 106  | -192 |
| 0.01 | 81   | -5   |
| 0.01 | 54   | -137 |
| 0    | 40   | -6   |
| 0    | 32   | -50  |
| 0    | 27   | -133 |
| 0    | 27   | -153 |
| 0    | 25   | -67  |

Supplement: Supplemental Material [file supp_gr.244293.118_Supplemental_Code_S1.zip › amplican_manuscript/figures/normalization/MiSeq_run9_2014_03_26/invit96ss_normalized.pdf]

Frame

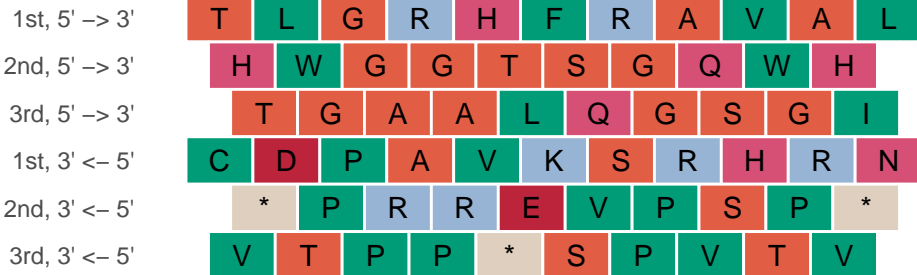

invit96ss

1

2

3

4

5

6

7

8

9

10

0

10

20

Relative Nucleotide Position

[%]

0 25 50 75 100

Match

9

Edited

26

F

65

Freq

Count

F

0.08

812

0

0.4

3852

-194

0.13

1275

-111

0.04

419

-155

0.03

305

-183

0.03

290

-79

0.03

252

-154

0.02

212

-188

0.02

201

-112

0.02

199

-174

0.02

196

-117

Supplement: Supplemental Material [file supp_gr.244293.118_Supplemental_Code_S1.zip › amplican_manuscript/figures/normalization/MiSeq_run9_2014_03_26/invit96ss_raw.pdf]

Frame

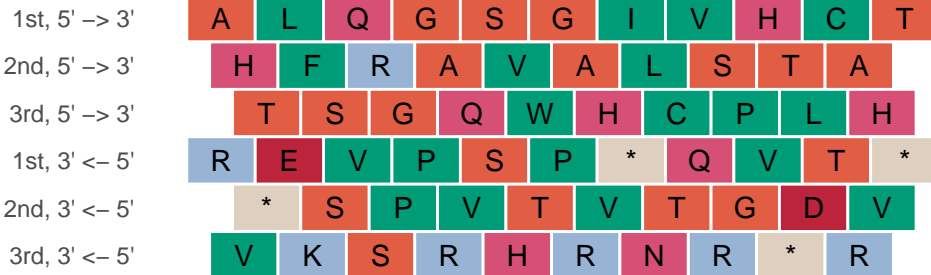

[ % ]

Match 93

Edited 2

F 5

amplicon

1

2

3

4

5

6

7

8

9

10

control1

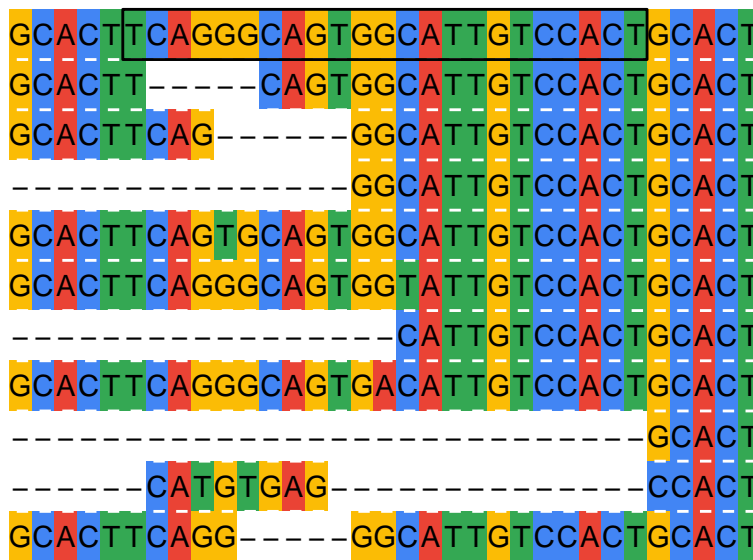

0

10

20

Relative Nucleotide Position

| Freq | Count | F   |
|------|-------|-----|
| 0.9  | 971   | 0   |
| 0.01 | 14    | -5  |
| 0.01 | 7     | -6  |
| 0.01 | 7     | -31 |
| 0    | 4     | 0   |
| 0    | 4     | 0   |
| 0    | 4     | -27 |
| 0    | 3     | 0   |
| 0    | 3     | -28 |
| 0    | 3     | -21 |
| 0    | 2     | -5  |

Supplement: Supplemental Material [file supp_gr.244293.118_Supplemental_Code_S1.zip › amplican_manuscript/figures/normalization/MiSeq_run9_2014_03_26/my96ss_control.pdf]

Frame

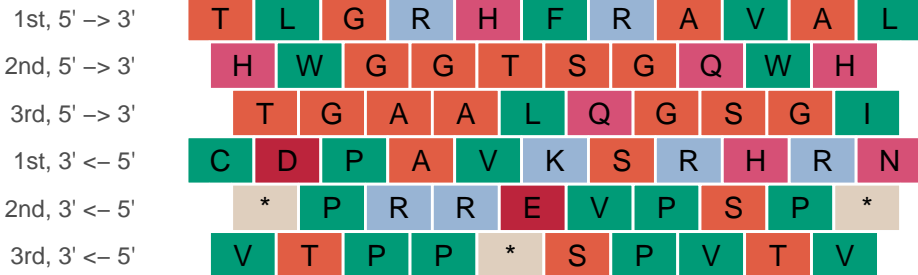

[%]

0 25 50 75 100

Match

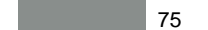

Edited

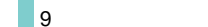

F 16

| Freq | Count | F    |
|------|-------|------|
| 0.73 | 2352  | 0    |
| 0.03 | 94    | -5   |
| 0.03 | 84    | -6   |
| 0.01 | 45    | -176 |
| 0.01 | 32    | -39  |
| 0.01 | 20    | -60  |
| 0.01 | 20    | -16  |
| 0    | 14    | -34  |
| 0    | 14    | 0    |
| 0    | 14    | -21  |
| 0    | 14    | -11  |

amplicon

1

2

3

4

5

6

7

8

9

10

my96ss

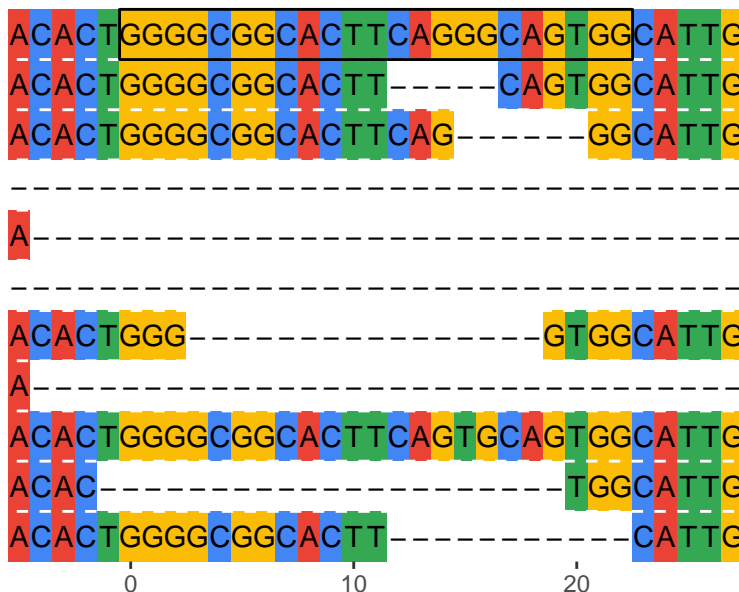

0

10

20

Relative Nucleotide Position

Supplement: Supplemental Material [file supp_gr.244293.118_Supplemental_Code_S1.zip › amplican_manuscript/figures/normalization/MiSeq_run9_2014_03_26/my96ss_normalized.pdf]

Frame

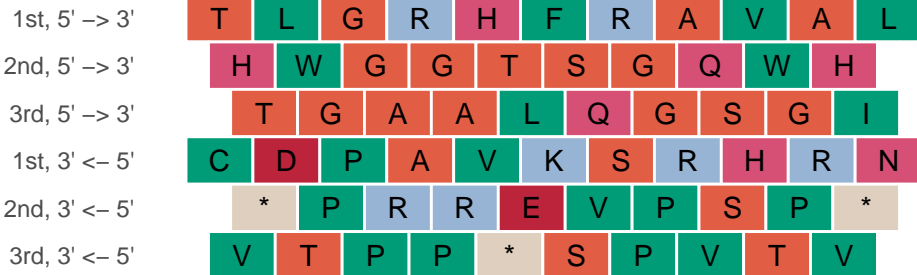

[%]

0 25 50 75 100

Match

31

Edited

19

F

49

amplicon

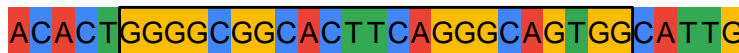

1

2

3

4

5

6

7

8

9

10

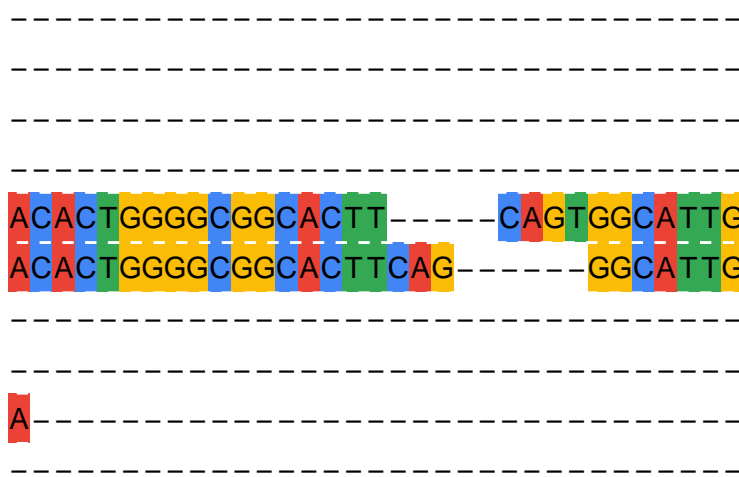

Freq

Count

F

0.29

943

0

0.21

666

-194

0.09

294

-111

0.09

289

-154

0.03

104

-155

0.03

94

-5

0.03

84

-6

0.01

45

-176

0.01

39

-174

0.01

32

-39

0.01

20

-60

0

10

20

Relative Nucleotide Position

my96ss

Supplement: Supplemental Material [file supp_gr.244293.118_Supplemental_Code_S1.zip › amplican_manuscript/figures/normalization/MiSeq_run9_2014_03_26/my96ss_raw.pdf]

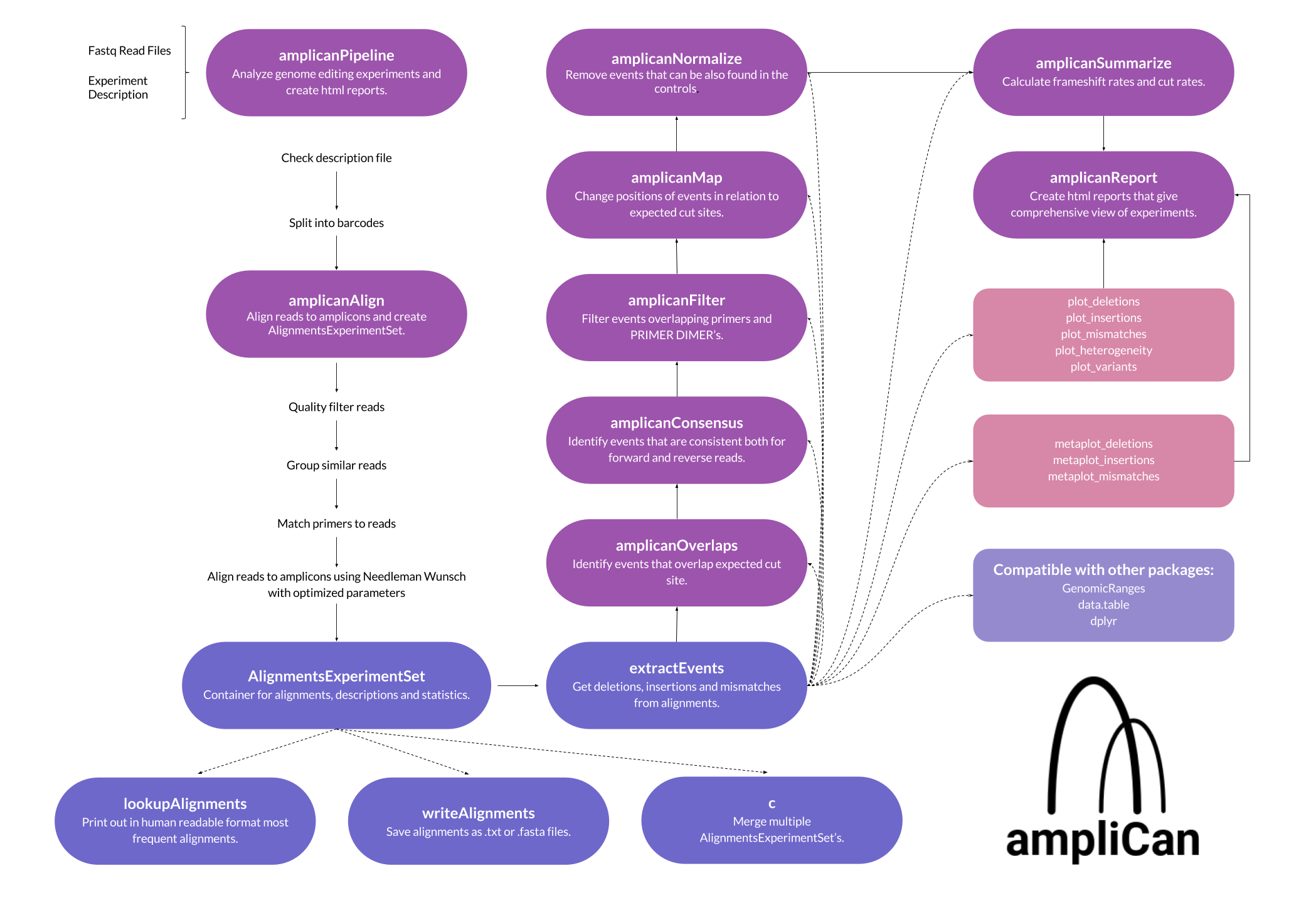

Supplement: Supplemental Material [file supp_gr.244293.118_Supplemental_Code_S2.tar.gz › amplican/vignettes/figures/amplican_conceptual_map.png]

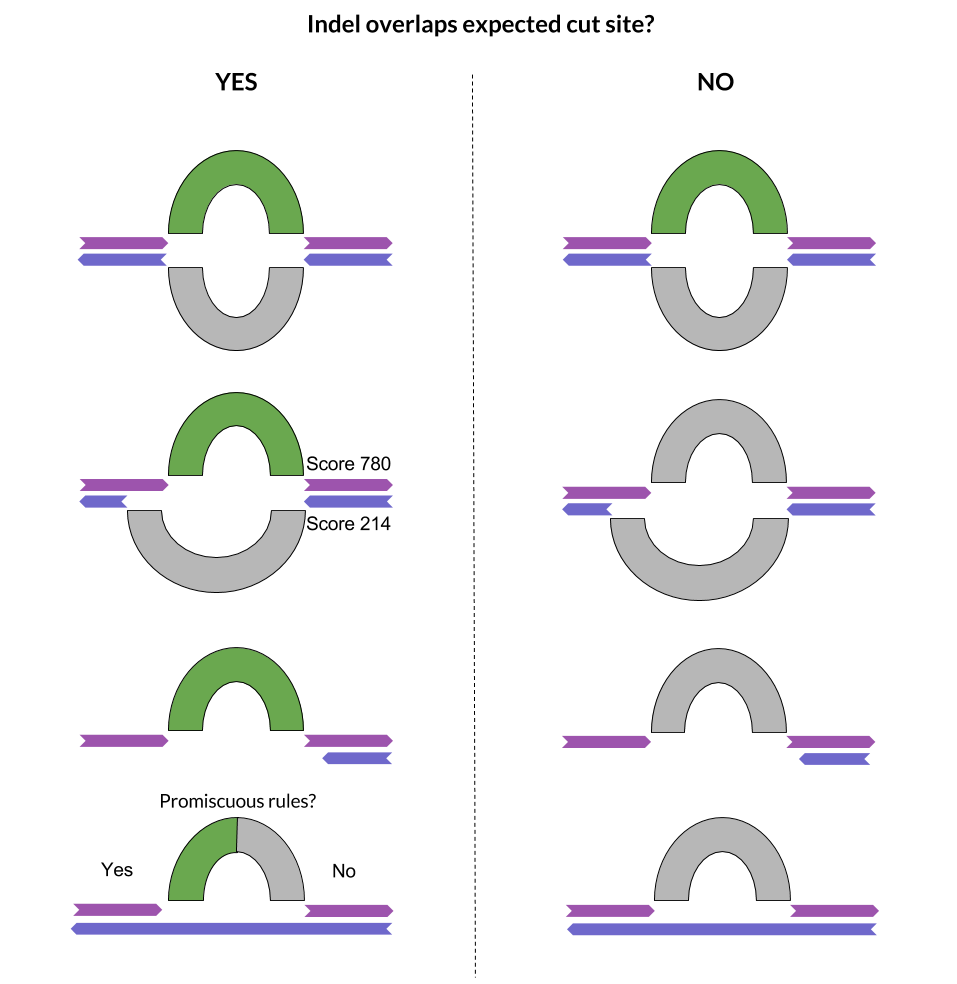

Supplement: Supplemental Material [file supp_gr.244293.118_Supplemental_Code_S2.tar.gz › amplican/vignettes/figures/amplican_consensus_rules.png]
