# Supplementary material for: Accurate analysis of genuine CRISPR editing events with ampliCan
Source: Genome Res. 2019 May;29(5):843–7. doi: 10.1101/gr.244293.118 (PMC6499316; doi:10.1101/gr.244293.118)
Supplement: Supplemental Material [file supp_gr.244293.118_Supplemental_Code_S2.tar.gz › amplican/inst/doc/amplicanFAQ.html]

ampliCan FAQ


# ampliCan FAQ

Kornel Labun & Eivind Valen

#### *22 January 2019*

#### Package

amplican 1.5.6

# Contents

- 1 Can ampliCan be used for TALENs, NICKASE or other types of genome editing?
- 2 I have one control that I want to use for many guides? How should I design the config file?
- 3 What is unique reads?
- 4 Why are Reads\_Edited different from the sum of Reads\_Del and Reads\_Ins?
- 5 Can amplican handle ABI files?
- 6 When should I adjust the cutoff for normalization?
- 7 What when I have not used unique dual indexing pooling combinations?

This vignette lists the most Frequently Asked Questions we receive about ampliCan.

# 1 Can ampliCan be used for TALENs, NICKASE or other types of genome editing?

Yes, `amplican` can be used more or less as normal. The expected edit site should still be placed as UPPER case letters, but should in the case of dimers span the region between the two binding sites. The guide sequence column is then typically set to the same as the uppercase region. If you have controls, you should make sure their Guide column and Group column are the same as the experiment for normalization.

# 2 I have one control that I want to use for many guides? How should I design the config file?

`amplican` is versatile in its normalization. In the default pipeline the guideRNA and Group columns determine which experiments are normalized by which. The Control column specifies what are to be considered controls as opposed to cases. The controls that match both the guideRNA and Group are averaged and are used to normalize every read from the case group with the same guideRNA and Group.

| ID | guideRNA | Group | Control |
| --- | --- | --- | --- |
| 1 | ACTG | g1 | 0 |
| 2 | ACTG | g1 | 0 |
| 3 | ACTG | g1 | 1 |
| 4 | ACTG | g2 | 1 |
| 5 | ACTG | g2 | 0 |
| 6 | ACTG | g2 | 0 |

In the above example, with default configuration, Experiment ID 1 and 2 will be normalized with ID 3, while ID 5 and 6 with ID 4.

However, as an alternative the user can only normalize by guideRNA match by specifying `normalize = c("guideRNA")` in the `amplicanPipeline`. If so, ID 3 and 4 will be averaged and will be used to normalize all cases since all experiments have matching guideRNA.

# 3 What is unique reads?

Unique reads is the number of reads when all duplicates are only counted once. For paired-end sequencing we reuiqre the combination of forward and reverse read to be unique. This is a simple metric of the heterogeneity of your reads.

If you have many reads, but few unique it means that many reads are identical. Possibly because CRISPR did not cut, or have cut in a highly specific manner. If you have very high number of unique reads, your reads are mostly different to each other. Sequencing errors, alignments and mosaic CRISPR activity can contribute to this. Both of those cases can happen in successful experiments, but usually a few reads tend to be more frequently sampled.

# 4 Why are Reads\_Edited different from the sum of Reads\_Del and Reads\_Ins?

Reads\_Del is the number of reads that had a deletion, Reads\_Ins is number of reads that had an insertion. Reads\_Edited is number of reads that had any edit, which can include reads with both insertion and deletion.

# 5 Can amplican handle ABI files?

`ampliCan` can at present not handle ABI directly, but ABI can be converted to fastq files using other software.

# 6 When should I adjust the cutoff for normalization?

There are mainly two reason to alter the normalization threshold:

1. When high precision is required (below 0.01%) it is beneficial to lower the normalization threshold eg. `min_freq = 0.001` if you have sufficient sequencing depth
2. When you have a homogenous genetic background or your sequencing depth is low it might be beneficial to set the threshold higher e.g. `min_freq = 0.1`.
3. You suspect/expect that there is Index Hopping occuring in your reads, in that scenario you should adjust threshold to e.g. `min_freq = 0.03` as expected Index Hopping levels can be as high as 0.02 frequency and can be confused as genetic background during normalization, if threshold is kept at default.

This should be apparent from the mismatch plot, where the frequency line of mismatches in the control should give you an idea of what the background noise level is.

# 7 What when I have not used unique dual indexing pooling combinations?

You can adjust threshold for normalization to `min_freq = 0.15` or use function `amplicanPipelineConservative`.
