# Supplementary material for: Accurate analysis of genuine CRISPR editing events with ampliCan
Source: Genome Res. 2019 May;29(5):843–7. doi: 10.1101/gr.244293.118 (PMC6499316; doi:10.1101/gr.244293.118)
Supplement: Supplemental Material [file supp_gr.244293.118_Supplemental_Code_S2.tar.gz › amplican/inst/doc/amplicanOverview.html]

ampliCan Overview


# ampliCan Overview

Kornel Labun & Eivind Valen

#### *22 January 2019*

#### Package

amplican 1.5.6

# Contents

- 1 Introduction
- 2 Configuration file
- 3 Default options
- 4 Files created during analysis
  - 4.1 barcode\_reads\_filters.csv
  - 4.2 config\_summary.csv
  - 4.3 RunParameters.txt
  - 4.4 “alignments” folder
  - 4.5 reports folder
- 5 Detailed analysis
  - 5.1 Aligning reads
  - 5.2 Normalization
  - 5.3 Making reports
  - 5.4 Plotting alignments events

Welcome to the `amplican` package. This vignette will walk you through our main package usage with example MiSeq dataset. You will learn how to interpret results, create summary reports and plot deletions, insertions and mutations with our functions. This package, `amplican`, is created for fast and precise analysis of CRISPR experiments.

# 1 Introduction

`amplican` creates reports of deletions, insertions, frameshifts, cut rates and other metrics in *knitable* to HTML style. `amplican` uses many `Bioconductor` and `CRAN` packages, and is high level package with purpose to align your fastq samples and automate analysis across different experiments. `amplican` maintains elasticity through configuration file, which, with your fastq samples are the only requirements.

For those inpatient of you, who want to see an example of our whole pipeline analysis on attached example data look here. Below you will find the conceptual map of amplican.

Conceptual map of amplican.

Below you will find the `amplicanConsensus` rules. That allow you to understand how ampliCan treats unambiguous forward and reverse reads. Green color indicates events that will be accepted. When forward and reverse reads agree, their events are in the same place and span the same length, we will take forward read event as representative. In case when events from forward and reverse read don’t agree we select event from strand with higher alignment score. In situation where one of the reads is not spanning event in question we consider this event as real (as we don’t have other information). If both strands cover event in question, but one strand has no indel, `amplicanConsensus` will change behavior according to the `strict` parameter.

Consensus rules of ampliCan.

# 2 Configuration file

To successfully run our analysis it is mandatory to have a configuration file. Take a look at our example:

| ID | Barcode | Forward\_Reads | Reverse\_Reads | Group | Control | guideRNA | Forward\_Primer | Reverse\_Primer | Direction | Amplicon | Donor |
| --- | --- | --- | --- | --- | --- | --- | --- | --- | --- | --- | --- |
| ID\_1 | barcode\_1 | R1\_001.fastq | R2\_001.fastq | Betty | 0 | AGGTGGTCAGGGAACTGG | AAGCTGACGGCTAAATGA | AATTACACAAGCGCAAACACAC | 0 | aagctgacggctaaatgaaaaatgtcaaacatctgttccaggtgctgcgtatgccagggcagaggAGGTGGTCAGGGAACTGGtggaggtcactgggataccctttcttcccacaccaatggggaaaggagtcctgccagatgaccatcccaactgtgttgctgcagccagatccaggtgtgtttgcgcttgtgtaatt |  |
| ID\_2 | barcode\_1 | R1\_001.fastq | R2\_001.fastq | Tom | 0 | TGACCCTCTGCCAACACAAGGGG | TGACCAAACCTTCTTAAGGTGC | CTCTGCTGCAAAATGCAAGG | 1 | aaatactgtcttgtgaccaaaccttcttaaggtgctattttgataataaactttattgtgcttttgtagttgtgCCCCTTGTGTTGGCAGAGGGTCAgcagaccagtaagtcttctcaatttcttttatttatgtgtagtgataaaaaaatgttaaattaaaattaaatgtttttttttgccttgcattttgcagcagaggatgat |  |
| ID\_3 | barcode\_2 | R1\_002.fastq | R2\_002.fastq | Tom | 0 | AGGTGGTCAGGGAACTGG | AAGCTGACGGCTAAATGA | AATTACACAAGCGCAAACACAC | 0 | aagctgacggctaaatgaaaaatgtcaaacatctgttccaggtgctgcgtatgccagggcagaggAGGTGGTCAGGGAACTGGtggaggtcactgggataccctttcttcccacaccaatggggaaaggagtcctgccagatgaccatcccaactgtgttgctgcagccagatccaggtgtgtttgcgcttgtgtaatt |  |
| ID\_4 | barcode\_2 | R1\_002.fastq | R2\_002.fastq | Betty | 0 | GTCCCTGCAACATTAAAGGCCGG | GCTGGCAACATTCCTACCAGT | GAGCGCTGAGGCAGGATTAT | 0 | gctggcaacattcctaccagtaatttacgtaaaaaaatgctataaaatgtgtagctctccagtctaatgtaacttgtgcttgcattgtgtttacaggaaaccaGTCCCTGCAACATTAAAGGCCGGaagtctaagaactcacatcagcaggtgtcaagtgtgcatgaagagggtataatcctgcctcagcgctc |  |
| ID\_5 | barcode\_2 | R1\_002.fastq | R2\_002.fastq | Betty | 0 | GTCCCTGCAACATTAAAGGCCGG | ACTGGCAACATTCCTACCAGT | ACTGGCTGAGGCAGGATTAT | 0 | actggcaacattcctaccagtaatttacgtaaaaaaatgctataaaatgtgtagctctccagtctaatgtaacttgtgcttgcattgtgtttacaggaaaccaGTCCCTGCAACATTAAAGGCCGGaagtctaagaactcacatcagcaggtgtcaagtgtgcatgaagagggtataatcctgcctcagccagt | actggcaacattcctaccagtaatttacgtaaaaaaatgctataaaatgtgtagctctccagtctaatgtaacttgtgcttgcattgtgtttacaggaaaccaGTCCCTGCAACATTAAAAGCCGGaagtctaagaactcacatcagcaggtgtcaagtgtgcatgaagagggtataatcctgcctcagccagt |

Configuration file should be a “,” delimited csv file with information about your experiments. You can find example config file path by running:

```
system.file("extdata", "config.csv", package = "amplican")
```

Columns of the config file:

- **ID** - should be a unique identifier of your experiments
- **Barcode** - use this to group experiments with the same barcode
- **Forward\_Reads**, **Reverse\_Reads** - put names of your files in these fields, you can put here files compressed to .gz, we will unpack them for you
- **Group** - use this field if you want to group ID by other criteria, here for instance we will group by person that performed experiment, on later stage it is possible to generate report with breakdown using this field
- **guideRNA** - put your guideRNA in 5’ to 3’ fashion here, if he has to be reverse complemented to be found in the amplicon put “1” into **Direction** field, we will use this field to make sure your guideRNA is inside the amplicon and during plotting of the results
- **Forward\_Primer**, **Reverse\_Primer** - make sure these primers are correct for each of your IDs, **Forward\_Primer** should be found in the amplicon as is, on the left side of the guide, **Reverse\_Primer** reverse complement should be found on the amplicon on the right side of the guide
- **Direction** - here “0” means guide does not requires to be reverse complemented to be matched to the amplicon, “1” means it should be reverse complemented, this field is also used when plotting deletions, mismatches and insertions
- **Amplicon** - insert amplicon sequence as lower case letters, by putting UPPER CASE letters you can specify expected cut site, you should specify at least one cut site, here for example we specify in uppercase letter PAM sequence of the corresponding guideRNA
- **Donor** - insert donor template if you have designed HDR experiments or used base editors, otherwise leave empty, but keep the column. `amplican` will estimate HDR efficiency based on the events from aligning donor and amplicon sequences, donor and reads and reads and amplicon.

If you have only forward primers leave column **Reverse\_Primer** empty, leave empty also the **Reverse\_Reads** column. You can still use amplican like normal.

# 3 Default options

To run `amplican` with default options, along with generation of all posible reports you can use `amplicanPipeline` function. We have already attached results of the default amplican analysis (look at other vignettes) of the example dataset, but take a look below at how you can do that yourself. Be prepared to grab a coffe when running `amplicanPipeline` with `knit_files = TRUE` as this will take some time. You will understand it is worth waiting when reports will be ready.

```
# path to example config file
config <- system.file("extdata", "config.csv", package = "amplican")
# path to example fastq files
fastq_folder <- system.file("extdata", package = "amplican") 
# output folder, a full path
results_folder <- tempdir()  

#  run amplican
amplicanPipeline(config, fastq_folder, results_folder)

# results of the analysis can be found at
message(results_folder)
```

Take a look into “results\_folder” folder. Here you can find `.Rmd` files that are our reports for example dataset. We already crafted `.html` versions and you can find them in the “reports” folder. Open one of the reports with your favourite browser now. To zoom on an image just open it in new window (right click -> open image in new tab).

`amplicanPipeline` just crafted very detailed reports for you, but this is not all, if you need something different e.g. different plot colours, just change the `.Rmd` file and `knit` it again. This way you have all the power over plotting.

# 4 Files created during analysis

## 4.1 barcode\_reads\_filters.csv

First step of our analysis is to filter out reads that are not complying with our default restrictions:

- bad base quality - default minimum base quality is 0
- bad average quality - default minimum average quality is 30
- bad alphabet - by default we accept only reads with A,C,T,G bases

| Barcode | experiment\_count | read\_count | bad\_base\_quality | bad\_average\_quality | bad\_alphabet | filtered\_read\_count | unique\_reads | unassigned\_reads | assigned\_reads |
| --- | --- | --- | --- | --- | --- | --- | --- | --- | --- |
| barcode\_1 | 2 | 20 | 0 | 3 | 3 | 14 | 8 | 1 | 7 |
| barcode\_2 | 3 | 21 | 0 | 0 | 0 | 21 | 9 | 0 | 9 |

This table is also summarized in one of the reports. As you can see for our example dataset we have two barcodes, to which correspond 21 and 20 reads. Six reads are rejected for barcode\_1 due to bad alphabet and bad average quality. Each of the barcodes has unique reads, which means forward and reverse reads are compacted when they are identical. There is 8 and 9 unique reads for each barcode. One read failed with assignment for barcode\_1, you can see this read in the top unassgned reads for barcode report in human readable form. Normally you will probably see only half of your reads being assigned to the barcodes. Reads are assigned when for forward read we can find forward primer and for reverse read we can find reverse primer. Primers have to be perfectly matched. Nevertheless, there is option `fastqreads = 0.5` which changes method of assigning reads to each IDs. With this option specified only one of the reads (forward or reverse) have to have primer perfectly matched. If you don’t have the reverse reads or you don’t want to use them you can use option `fastqreads = 1`, this option should be detectd autmatically, if you leave empty field **Reverse\_Primer** in the config file.

## 4.2 config\_summary.csv

`config_summary.csv` contains extended version of the config file. It should provide you additional look at raw numbers which we use for various plots in reports. Take a look at example extension:

| ID | Barcode | Reads | Reads\_Filtered | Reads\_In | Reads\_Del | Reads\_Edited | Reads\_Frameshifted |
| --- | --- | --- | --- | --- | --- | --- | --- |
| ID\_1 | barcode\_1 | 7 | 6 | 0 | 6 | 6 | 2 |
| ID\_2 | barcode\_1 | 6 | 6 | 0 | 6 | 6 | 4 |
| ID\_3 | barcode\_2 | 9 | 8 | 1 | 7 | 8 | 1 |
| ID\_4 | barcode\_2 | 7 | 7 | 7 | 4 | 7 | 2 |
| ID\_5 | barcode\_2 | 5 | 5 | 0 | 0 | 2 | 0 |

During `amplicanPipeline` these columns are added to the config file:

- **Reads\_Del, Reads\_In, Reads\_Edited** - number of deletions, insertions or any of those two (mutations) overlapping with user specified UPPER CASE group in the amplicon (extended by the buffer), events are confirmed with the reverse strand when using paired-end reads, for more information check `amplicanConsensus`
- **Frameshifted** - number of reads that have frameshift (insertions and deletions)
- **PRIMER\_DIMER** - number of reads that were classified as PRIMER DIMERs
- **Reads** - number of reads assigned to this unique ID
- **Reads\_Filtered** - number of reads assigned to this unique ID with excusion of PRIMER DIMERs and Low Alignment Score reads

## 4.3 RunParameters.txt

File RunParameters.txt lists all options used for the analysis, this file you might find useful when reviewing analysis from the past where you forgot what kind of options you used. Other than that this file has no purpose.

```
# path to example RunParameters.txt
run_params <- system.file("extdata", "results", "RunParameters.txt", 
                          package = "amplican")
# show contents of the file
readLines(run_params)
```

```
##  [1] "Config file:         full/path/to/config/file/that/has/been/used.csv"
##  [2] "Average Quality:     30"                                             
##  [3] "Minimum Quality:     0"                                              
##  [4] "Write Alignments:    txt"                                            
##  [5] "Fastq files Mode:    0"                                              
##  [6] "Gap Opening:         25"                                             
##  [7] "Gap Extension:       0"                                              
##  [8] "Consensus:           TRUE"                                           
##  [9] "Normalize:           guideRNA, Group"                                
## [10] "PRIMER DIMER buffer: 30"                                             
## [11] "Cut buffer: 5"                                                       
## [12] "Scoring Matrix:"                                                     
## [13] ",A,C,G,T"                                                            
## [14] "A,5,-4,-4,-4"                                                        
## [15] "C,-4,5,-4,-4"                                                        
## [16] "G,-4,-4,5,-4"                                                        
## [17] "T,-4,-4,-4,5"
```

## 4.4 “alignments” folder

As name indicates it contains all alignments.

```
# path to the example alignments folder
system.file("extdata", "results", "alignments", package = "amplican")
```

In `unassigned_reads.csv` you can find detailed information about unassigned reads. In example dataset there is one unassigned read.

Take a look at the alignment events file which contains all the insertions, deletions, cuts and mutations. This file can be used in various ways. Examples you can find in `.Rmd` files we prepare using `amplicanReport`. These can be easily converted into `GRanges` and used for further analysis! Events are saved at three points of `amplicanPipeline` analysis. First file “raw\_events.csv” contains all events directly extracted from aligned reads. After filtering PRIMER DIMER reads, removing events overlapping primers (alignment artifacts) and shifting events so that they are relative to the expected cut sites “events\_filtered\_shifted.csv” is saved. After normalization through `amplicanNormalize` “events\_filtered\_shifted\_normalized.csv” is saved, probably it is the file you should use for further analysis.

| seqnames | start | end | width | strand | originally | replacement | type | read\_id | score | counts | readType | overlaps | consensus |
| --- | --- | --- | --- | --- | --- | --- | --- | --- | --- | --- | --- | --- | --- |
| ID\_1 | 42 | 61 | 20 | + |  | AAAAAAAAAAAAAAAAAAAA | insertion | 1 | 597 | 3 | FALSE | FALSE | TRUE |
| ID\_1 | 46 | 65 | 20 | + |  | AAAAAAAAAAAAAAAAAAAA | insertion | 2 | 557 | 2 | FALSE | FALSE | TRUE |
| ID\_1 | -24 | 41 | 66 | + |  |  | deletion | 1 | 597 | 3 | FALSE | TRUE | TRUE |
| ID\_1 | -28 | 45 | 74 | + |  |  | deletion | 2 | 557 | 2 | FALSE | TRUE | TRUE |
| ID\_1 | -32 | 51 | 84 | + |  |  | deletion | 4 | 532 | 1 | FALSE | TRUE | TRUE |
| ID\_1 | -35 | -35 | 1 | + | A | G | mismatch | 1 | 597 | 3 | FALSE | FALSE | TRUE |

Human readable alignments can be accesed using `lookupAlignment` function of `AlignmentsExperimentSet` object which contains all information after alignments from multiple experiments. Human readable format looks like this:

```
aln <- system.file("extdata", "results", "alignments", 
                   "AlignmentsExperimentSet.rds", 
                   package = "amplican")
aln <- readRDS(aln)
amplican::lookupAlignment(aln, ID = "ID_1") # will print most frequent alignment for ID_1
```

```
## ########################################
## # Forward read for ID ID_1 read_id 1
## # Program: Biostrings (version 2.48.0), a Bioconductor package
## # Rundate: Tue Jan 22 14:46:56 2019
## ########################################
## #=======================================
## #
## # Aligned sequences:
## # Forward read: P1
## # Amplicon sequence: S1
## # Matrix: NA
## # Gap_penalty: 25.0
## # Extend_penalty: 0.0
## #
## # Length: 219
## # Identity:     131/219 (59.8%)
## # Similarity:    NA/219 (NA%)
## # Gaps:          86/219 (39.3%)
## # Score: 597
## #
## #
## #=======================================
## 
## P1                 1 AAGCTGACGGCTAAATGAAAAATGTCAAACGTCTGTTCCAG---------     41
##                      |||||||||||||||||||||||||||||| ||||||||||         
## S1                 1 AAGCTGACGGCTAAATGAAAAATGTCAAACATCTGTTCCAGGTGCTGCGT     50
## 
## P1                42 --------------------------------------------------     41
##                                                                        
## S1                51 ATGCCAGGGCAGAGGAGGTGGTCAGGGAACTGGTGGAGGTCACTGGGATA    100
## 
## P1                42 -------AAAAAAAAAAAAAAAAAAAATTCCCACACCAATGGGGAAAGGA     84
##                                                 |||||||||||||||||||||||
## S1               101 CCCTTTC--------------------TTCCCACACCAATGGGGAAAGGA    130
## 
## P1                85 GTCCTGCCAGATGACCATCCCAACTGTGTTGCAGCAGCCAGATCCAGGTG    134
##                      |||||||||||||||||||||||||||||||| |||||||||||||||||
## S1               131 GTCCTGCCAGATGACCATCCCAACTGTGTTGCTGCAGCCAGATCCAGGTG    180
## 
## P1               135 TGTTTGCGCTTGTGTAATT    153
##                      |||||||||||||||||||
## S1               181 TGTTTGCGCTTGTGTAATT    199
## 
## 
## #---------------------------------------
## #---------------------------------------
## ########################################
## # Reverse read for ID ID_1 read_id 1
## # Program: Biostrings (version 2.48.0), a Bioconductor package
## # Rundate: Tue Jan 22 14:46:56 2019
## ########################################
## #=======================================
## #
## # Aligned sequences:
## # Reverse read: P1
## # Amplicon sequence: S1
## # Matrix: NA
## # Gap_penalty: 25.0
## # Extend_penalty: 0.0
## #
## # Length: 219
## # Identity:     131/219 (59.8%)
## # Similarity:    NA/219 (NA%)
## # Gaps:          86/219 (39.3%)
## # Score: 597
## #
## #
## #=======================================
## 
## P1                 1 AAGCTGACGGCTAAATGAAAAATGTCAAACGTCTGTTCCAG---------     41
##                      |||||||||||||||||||||||||||||| ||||||||||         
## S1                 1 AAGCTGACGGCTAAATGAAAAATGTCAAACATCTGTTCCAGGTGCTGCGT     50
## 
## P1                42 --------------------------------------------------     41
##                                                                        
## S1                51 ATGCCAGGGCAGAGGAGGTGGTCAGGGAACTGGTGGAGGTCACTGGGATA    100
## 
## P1                42 -------AAAAAAAAAAAAAAAAAAAATTCCCACACCAATGGGGAAAGGA     84
##                                                 |||||||||||||||||||||||
## S1               101 CCCTTTC--------------------TTCCCACACCAATGGGGAAAGGA    130
## 
## P1                85 GTCCTGCCAGATGACCATCCCAACTGTGTTGCAGCAGCCAGATCCAGGTG    134
##                      |||||||||||||||||||||||||||||||| |||||||||||||||||
## S1               131 GTCCTGCCAGATGACCATCCCAACTGTGTTGCTGCAGCCAGATCCAGGTG    180
## 
## P1               135 TGTTTGCGCTTGTGTAATT    153
##                      |||||||||||||||||||
## S1               181 TGTTTGCGCTTGTGTAATT    199
## 
## 
## #---------------------------------------
## #---------------------------------------
```

## 4.5 reports folder

Reports are automated for the convenience of the users. We provide 6 different reports. Reports are .Rmd files which can be easily crafted through `rmarkdown::render(path_to_report)` or by clicking `Knit` in Rstudio to make HTML version of the report. If you have run our example analysis, then you can open one of the reports with Rstudio and try knitting it now! Otherwise you can open one of already knitted example report in the vignettes.

# 5 Detailed analysis

## 5.1 Aligning reads

When you want to have more control over alignments and you need more advanced options use `amplicanAlign`. This function has many parameters which can be used to bend our pipeline to your needs. Read more about `amplicanAlign` on the help page `?amplican::amplicanAlign`.

## 5.2 Normalization

Read more about normalization in the description of the `?amplican::amplicanNormalize` or in FAQ. Just note that default setting of normalization threshold is 1%, if you notice in your mismatch plots that general level of noise (mismatches outside cut region) might be higher than that of 1%, you have to adjust threshold value to higher levels (min\_freq input parameter). In case where you expect index hopping to occour, you can use either `?amplican::amplicanPipelineConservative` or rise the min\_freq above expected index hopping level.

## 5.3 Making reports

Reports are made for user convenience, they are powerful as they:

- contain reproducible code
- make plots ready for publication
- show how to use results of our pipeline
- are easy to share
- allow quick, but detailed assessment of the data

We decided to separate reports into 6 different types. Function `amplicanReport` is made for automated report creation.

Types of report:

- **ID** - Each of the IDs is treated separately, you get to see what kind of cuts, deletions, insertions and mismatches are being found for each of the IDs. example
- **Amplicon** - We aggregate information about each unique amplicon, you will find here also plots for events found during alignment. example
- **Barcode** - Grouped by Barcode, instead of the information about alignment events you can find here histograms explaining cut rates, frameshifts etc. example
- **Group** - This field is for your convenience and allows you to group IDs by anything you want. In our example dataset we use this field to group experiments by the users. That will allow us to asses performance of our technicians. example
- **Guide** - Unique guideRNAs are being summarized by boxplots showing cut rates across all experiments. example
- **Index** - Has links to the other reports and can be treated as main summary. Contains overall statistics gathered during reads assesment. Can contain only barcode level information, but shows which parts of reads is being filtered. example

## 5.4 Plotting alignments events

We provide specialized plots for each type of the alignment events.

`plot_mismatches` - plots mismatches as stacked bar-plot over the amplicon, top plot is for the forward and bottom is for reverse reads  
`plot_deletions` - plots deletions as arch-plot from the ggbio package  
`plot_insertions` - each insertion is represented by triangle over the amplicon  
`plot_cuts` - gathers cuts from each of the experiments and overlays multiple arch-plots on top of each other, useful when analyzing what kind of cuts were introduced in the amplicon  
`plot_variants` - presents most frequent mutations with frameshift and codon information  
`plot_heterogeneity` - shows a measure of read “uniqueness”

You can take a look at all these plots and how to make them in the example reports. There are also meta versions of above plots, that discard amplicon information and allow to overlay multiple different amplicons on top of each other eg. `metaplot_deletions`.
