# Supplementary material for: Accurate analysis of genuine CRISPR editing events with ampliCan
Source: Genome Res. 2019 May;29(5):843–7. doi: 10.1101/gr.244293.118 (PMC6499316; doi:10.1101/gr.244293.118)
Supplement: Supplemental Material [file supp_gr.244293.118_Supplemental_Code_S2.tar.gz › amplican/inst/doc/example_barcode_report.html]

Report breakdown by barcode


# Report breakdown by barcode

#### *ampliCan*

#### *22 January 2019*

---

# 1 Description

---

**Read distribution plot** - plot shows number of reads assigned during read grouping  
**Filtered Reads** - plot shows percentage of assigned reads that have been recognized as PRIMER DIMERS or filtered based on low alignment score  
**Edit rates** - plot gives overview of percentage of reads (not filtered as PRIMER DIMER) that have edits  
**Frameshift** - plot shows what percentage of reads that have frameshift  
**Frameshift overlapping** - shows what percentage of reads have frameshift counting only deletions and insertions that overlap expected cut site (should be more accurate when controls are not available)  
**Read heterogeneity plot** - shows what is the share of each of the unique reads in total count of all reads. The more yellow each row, the less heterogeneity in the reads, more black means reads don’t repeat often and are unique  
**Top unassigned reads** - take a look at the alignment of most abundant forward and reverse complemented reverse reads for each barcode, if you find that there is many unassigned reads you can ivestigate here.

---

# 2 Barcode Summary

---

## 2.1 Groups IDs

| group | IDs |
| --- | --- |
| barcode\_1 | ID\_1, ID\_2 |
| barcode\_2 | ID\_3, ID\_4, ID\_5 |

## 2.2 Read distribution

## 2.3 Filtered Reads

## 2.4 Edit rates

## 2.5 Frameshift

## 2.6 Heterogeneity of reads

---

# 3 Top unassigned reads

---

## 3.1 barcode\_1

| Forward | Reverse | Counts | Frequency |
| --- | --- | --- | --- |
| P1 | S1 | 1 | 0.0714286 |

```
P1                 1 AAAT--ACTGTCTTGTGACCAAACCTTCTTAAGGTGCTGTTTT-GATGAT     47
                     ||    || |     |||  |||   ||| ||    |||||   | || |
S1                 1 AAGCTGACGGCTAAATGA--AAAATATCTGAAACATCTGTTCCAGGTGCT     48

P1                48 AAACTTTATTGTGCTTTTGTAGTTGTGCCCCTTGTGTTGGCAGAGGGTCA     97
                          |    | ||    | ||  | | |      | |  | |  |||||
S1                49 GCGTATGCCAGGGCAGA-GAAGAAG-GTCAGGGAAGGTCACTGGAGGTCA     96

P1                98 ---GCAGACCAGTAAGTCTTCTCAATTTCTTTTATTTATGTATATGTAGT    144
                        | | |||  |   ||||| ||      |      | |  |       
S1                97 CTGGGATACCCTT---TCTTCCCACACCAATGGGGAAAGGAGTCCTGCCA    143

P1               145 GATAAA-A    151
                     ||| |  |
S1               144 GATGACCA    151
```
