# Supplementary material for: Accurate analysis of genuine CRISPR editing events with ampliCan
Source: Genome Res. 2019 May;29(5):843–7. doi: 10.1101/gr.244293.118 (PMC6499316; doi:10.1101/gr.244293.118)
Supplement: Supplemental Material [file supp_gr.244293.118_Supplemental_Code_S2.tar.gz › amplican/inst/doc/example_guide_report.html]

Report breakdown by guideRNA


# Report breakdown by guideRNA

#### *ampliCan*

#### *22 January 2019*

---

# 1 Description

---

**Read distribution plot** - plot shows number of reads assigned during read grouping  
**Filtered Reads** - plot shows percentage of assigned reads that have been recognized as PRIMER DIMERS or filtered based on low alignment score  
**Edit rates** - plot gives overview of percentage of reads (not filtered as PRIMER DIMER) that have edits  
**Frameshift** - plot shows what percentage of reads have frameshift  
**Frameshift overlapping** - shows what percentage of reads have frameshift counting only deletions and insertions that overlap expected cut site (should be more accurate when controls are not available)  
**Read heterogeneity plot** - shows what is the share of each of the unique reads in total count of all reads. The more yellow each row, the less heterogeneity in the reads, more black means reads don’t repeat often and are unique

---

# 2 guideRNA Summary

---

## 2.1 Read distribution

## 2.2 Filtered Reads

## 2.3 Edit rates

## 2.4 Frameshift

## 2.5 Heterogeneity of reads

---

# 3 Alignments plots

---

## 3.1 Guide AGGTGGTCAGGGAACTGG

### 3.1.1 Deletions

### 3.1.2 Insertions

### 3.1.3 Mismatches

[1] “No mismatches to plot.”

## 3.2 Guide TGACCCTCTGCCAACACAAGGGG

### 3.2.1 Deletions

### 3.2.2 Insertions

[1] “No insertions to plot.”

### 3.2.3 Mismatches

[1] “No mismatches to plot.”

## 3.3 Guide GTCCCTGCAACATTAAAGGCCGG

### 3.3.1 Deletions

### 3.3.2 Insertions

### 3.3.3 Mismatches

[1] “No mismatches to plot.”
