# Supplementary material for: Accurate analysis of genuine CRISPR editing events with ampliCan
Source: Genome Res. 2019 May;29(5):843–7. doi: 10.1101/gr.244293.118 (PMC6499316; doi:10.1101/gr.244293.118)
Supplement: Supplemental Material [file supp_gr.244293.118_Supplemental_Code_S2.tar.gz › amplican/inst/doc/example_index.html]

Summary Read Report


# Summary Read Report

#### *ampliCan*

#### *22 January 2019*

---

# 1 Other Reports

---

1. Report by id
2. Report by barcode
3. Report by group
4. Report by guide
5. Report by amplicon

---

# 2 Explanation of variables

---

**Experiment Count** - how many IDs belongs to this barcode  
**Read Count** - how many reads belongs to this barcode  
**Bad Base Quality** - how many reads had base quality worse than specified (default is 0)  
**Bad Average Quality** - how many reads had average base quality worse than specified (default is 0)  
**Bad Alphabet** - how many reads had alphabet with bases other than A, C, G, T  
**Filtered Read Count** - how many reads were left after filtering  
**Unique Reads** - how many reads (forward and reverse together) for this barcode is unique  
**Assigned Reads/Unassigned Reads** - how many reads have been assigned/not assigned to any of the experiments

---

# 3 Total reads

---

## 3.1 Read Quality

## 3.2 Read assignment

## 3.3 Edits

---

# 4 Reads by barcode

---

---

# 5 Summary Table

---

| Barcodes | Experiment Count | Read Count | Bad base quality | Bad average quality | Bad alphabet | Good Reads | Unique Reads | Unassigned Reads | Assigned Reads |
| --- | --- | --- | --- | --- | --- | --- | --- | --- | --- |
| barcode\_1 | 2 | 20 | 0 | 3 | 3 | 14 | 8 | 1 | 7 |
| barcode\_2 | 3 | 21 | 0 | 0 | 0 | 21 | 9 | 0 | 9 |

Table 1. Reads distributed for each barcode

---
