# Supplementary material for: Accurate analysis of genuine CRISPR editing events with ampliCan
Source: Genome Res. 2019 May;29(5):843–7. doi: 10.1101/gr.244293.118 (PMC6499316; doi:10.1101/gr.244293.118)
Supplement: Supplemental Material [file supp_gr.244293.118_Supplemental_Material.pdf]

Supplemental Text and Figures

Table of contents

Availability of code and data **2**

Data Overview **2**

Supplemental Note S1: Tools differ in estimating editing efficiency on real data. **3**

Supplemental Note S2: Automatic normalization using control reads **4**

Supplemental Note S3: ampliCan utilizes optimized alignments **6**

Supplemental Note S4: Synthetic data set evaluation **7**

Supplemental Note S5: ampliCan is able to correctly call longer indels **8**

Supplemental Note S6: ampliCan consistently recovers the true HDR efficiency when faced with diverse donor templates **10**

Supplemental Note S7: Visualization and aggregation of the complete activity of gRNAs **10**

Supplemental Note S8: Filtering of noise **11**

Low quality reads **11**

Primer-dimers **12**

Erroneously assigned reads and sequencing artifacts **12**

Supplemental Note S9: Read assignment **13**

Supplemental Figures **14**

Supplemental Tables **40**

References **45**

Availability of code and data

All code and configuration files used for the analyses in this manuscript are available at https://github.com/valenlab/amplican_manuscript where they can be used for independent verification. Copy of this repository is also available as **Supplementary Code S1**. Data analysed is publicly available and accessible at their respective accession numbers. ampliCan is available from Bioconductor as the R package at http://bioconductor.org/packages/amplican. To obtain the newest development version visit https://github.com/valenlab/amplican.

Data Overview

In this manuscript we have used multiple datasets, both real and simulated. Real datasets from Gagnon et al. 2014, runs 6-10 (accessible under E-MTAB-6310, E-MTAB-6355, E-MTAB-6356, E-MTAB-6357, E-MTAB-6358) were supplemented with our experiments for a total of 263 loci. All experiments had a control sample where no guideRNA was injected (accessible under BioProject PRJNA245510, run 1 and run 5). This dataset was used to highlight importance of controls as well as differences between the tools. The data is further discussed in **Supplemental Note S1**. Additional real data from ~ 1400 loci from Chari et al. 2015 was used for assessment whether large deletions can happen in real experiments.

Simulated datasets were created using a strategy similar to Lindsay et al. 2016 where CRISPR editing is emulated based on distributions of events (mismatches, deletions and insertions) from real experimental data (20 loci from (Shah et al. 2016)). FASTQ files were created using ART (Huang et al. 2012) where qualities were set to be uniformly high. Configuration files were

created using base loci sequences, same as used for CRISPR editing simulation. This data is further detailed in **Supplemental Note S4**. Complete overview of all datasets is presented in **Supplemental Tab S6**.

Supplemental Note S1: Tools differ in estimating editing efficiency on real data.

We assessed how tools estimate editing efficiency on 263 real CRISPR experiments, of which 151 were previously published by us (Gagnon et al. 2014), datasets from run 1 and run 5 available at BioProject under accession number PRJNA245510), and 112 novel experiments from 5 sets for this study (datasets from run 6-10 available at ArrayExpress: E-MTAB-6310, E-MTAB-6355, E-MTAB-6356, E-MTAB-6357, E-MTAB-6358). All experiments were conducted by injection into 1 cell zebrafish embryos and sequenced 2 days post-fertilization (Gagnon et al. 2014). Due to the rapid cell division and development these experiments are likely to result in highly heterogeneous mutational efficiencies from mosaicism. For these experiments the true mutation efficiency is not known and we can therefore not assess how precise the tools are in their estimates. Instead, we quantified how much the tools differ in their estimates (**Supplemental Fig S1A)** and, to qualitatively assess the underlying reason for their discrepancy, we plotted the estimated mutation efficiency values of the tools relative to the non-normalized ampliCan result (**Supplemental Fig S1B**). This showed that discrepancies are likely to originate from different causes. Some, those above the normalized ampliCan estimate, likely stem from a failure to consider control experiments. In our data the experiments impacted by controls is about 5%, but this will depend heavily on the reference genome, heterogeneity of the region and organism under study. Specific examples of the importance of normalization are shown in **Supplemental Fig S5**. The discrepancies of the other experiments

are due to the steps in the processing pipeline, e.g. off-target detection, primer dimer filtering, alignment strategy and read merging. To investigate the specific sources of these discrepancies and quantitatively assess the performance of the tools we created several synthetic benchmark datasets.

Supplemental Note S2: Automatic normalization using control reads

By default ampliCan normalizes through the strict removal of all editing events (insertions, deletions, mutations) that are also found above a threshold in the control sample. The default threshold value is a frequency of 0.01 and was chosen based on the typical frequency of low-abundance editing events (‘background noise’) present in control experiments (**Supplemental Fig S4**). These events are assumed to be technical or experimental artifacts present in most experiments. The threshold is also selected to be well above the expected Illumina error rate (Ross et al. 2013).

The threshold can be adjusted to increase the precision of indel detection when sequencing depth is high or account for a higher error rate in low-depth/precision experiments. The threshold can be set for instance based on the background error in the user’s own control experiments or in the absence of controls by inspecting the error rate outside of the target site (see for instance in **Supplemental Fig S14A**). Alternatively, if the user has information about the level of variance or noise expected in the case and/or the controls (e.g. genetic variance restricted to 100% or 50%) the threshold can be raised for increased stringency. This may in particular be useful if the user expects high frequency background events in the control experiments. One such use case is if index hopping is likely to be an issue. Index hopping may cause reads from the edited sample to erroneously be assigned to the control sample. We

therefore offer a second pipeline ‘amplicanPipelineConservative’ for experiments where this is expected to be a problem featuring a more stringent threshold of 0.15. For more information about index hopping and how to mitigate the problem see Illumina’s webpage (https://www.illumina.com/science/education/minimizing-index-hopping.html, Accessed January 22, 2019).

Given that the sequencing depth is sufficient the default settings should allow detection of indels as low as 0.01% (**Supplemental Tab S5**). If higher accuracy is necessary this threshold can be lowered and the sequencing depth increased. In the extreme case of setting the normalization threshold to 0% any event found in control would be removed from the case sample. This may result in removal of real edits and consequently the underestimation of real editing events.

Due to the stochasticity in sequencing data this approach is better suited to handle more heterogeneous cases than the subtraction method where indel frequency is simply normalized by subtraction of the control indel frequency. In the latter case variation in the levels of indel frequencies in the control versus CRISPR treated samples can lead to partially normalized data. Both normalization methods are outlined in **Supplemental Fig S2** while **Supplemental Fig S3** shows examples of highly heterogeneous data where without normalization estimated mutation efficiency would be biased. Normalization becomes even more important when the exact nature of the indel event matters, for instance whether it induces a frameshift or not (**Supplemental Fig S5**). Examples in **Supplemental Fig S5** can be recreated with the use of “make_comparison_normalized.Rmd” in the amplican_manuscript repository.

As a test for the normalization method we calculated the (non-existent) mutation frequency of the control samples normalized by the CRISPR-edited samples in order to see if false positives were generated. This was accomplished by 1) only considering the region between the sequence complementary to the guide RNA and the primer-matching sequence, and 2) excluding all large deletion events (>10bp) which could extend beyond the boundaries of this region. This resulted in a mean estimated mutation frequency of 0.0038 (median of 0.0012), well below the detection limit for the standard settings of 0.01.

Supplemental Note S3: ampliCan utilizes optimized alignments

CRISPR genome editing events typically result in a single break at a single site and by extension produce a single deletion and/or insertion. Sequence read aligners are generally not optimized for this type of genome editing event which can lead to the aligner fragmenting the indels and creating multiple events (example in **Supplemental Fig S6**). In the worst case fragmented alignments could shift the indel events resulting in a distortion of the mutation efficiency for those tools that only allow events within a certain distance from the expected site. A more likely outcome however, is the misinterpretation of the nature of the mutation.

Under certain assumptions the theoretically optimal alignment can be obtained by the Needleman-Wunsch algorithm. ampliCan uses this algorithm with optimized parameters to reflect the expectation that a CRISPR experiment should result in one deletion and/or insertion event, of unknown length (match = 5, mismatch = -4, gap opening = 25, gap extension = 0, no end gap penalties). With these parameters the Needleman-Wunsch algorithm performs well over a broad range of test cases (data not shown). ampliCan uses these optimized parameters

by default, but also allows for supervision of the alignments through human readable output of individual alignment results (**Supplemental Table S4**).

Supplemental Note S4: Synthetic data set evaluation

The latest available versions was used for all tools and packages. The assessment set from Lindsay et al. (Lindsay et al. 2016) paper (Synthetic Dataset 2, Supplemental 4) was replicated with the same settings and seed values as described (**Supplemental Fig S7**). The script from Lindsay et al. 2016 was used for parsing, but a small bug in the code was fixed for the CRISPResso output. In Lindsay et al. only NHEJ estimation of mutation efficiency was considered for CRISPResso, skipping HDR and "mixed" mutation frequencies. However, fixing this error did not influence CRISPResso's overall performance in any significant way. Versions of tools, scripts and details needed for replication are available in the https://github.com/valenlab/amplican_manuscript repository. ampliCan used the same amplicon sequences as CRISPResso.

It should be noted that Synthetic Dataset 2 from Lindsay et al. 2016 (used for **Supplemental Fig S7**) is not a good approximation of a real life situation. First, the sequence matching the primers can not be very divergent as they would then fail to amplify. Second, several experiments are badly designed in that the target sites are very close to the sequencing end of the reads. This makes it difficult to correctly call indels with support from both paired reads. Third, paired-end sequencing of 200bp or longer is somewhat expensive and error-prone and most labs would seek to restrict this to shorter reads. To account for this we created an additional set, Synthetic Dataset 3, in a similar fashion to Synthetic Dataset 2, but with with the following minor modifications. First, the length of amplicons and reads (150 bp) were adjusted.

Second, gRNA target sites were designed to be covered by both reads. Third, PCR off-target reads were created without mutating the primer sequences. Finally, mutation efficiency was tested across a range of mismatch rates, 10%, 20% and 30% (**Fig 2**, **Supplemental Fig S8**), to reflect different levels of similarity to the contaminant reads.

For Synthetic Dataset 2 ampliCan matches the perfect score of CrispRVariants and AmpliconDivider. However, on Synthetic Dataset Dataset 3 ampliCan is more consistent at estimating the known mutation efficiencies within the dataset (**Fig 2**). AmpliconDIVider has no filtering step and is confused by the contaminating reads. CrispRVariants has a filtering step, but is unable to discern divergent off-target sequences (e.g. homologous regions) that are still able to align to the correct target site. As in the benchmark from Lindsay et al. 2016 CRISPResso performs poorly on all benchmarks. When increasing mismatch rate (from 10% of all bases to 20% and 30%), AmpliconDIVider and CrispRVariants get closer to the correct estimated indel rate, but in all cases ampliCan obtains the highest precision and shows the most robust performance (**Fig 2**, **Supplemental Fig S8**).

Supplemental Note S5: ampliCan is able to correctly call longer indels

We have found that even without targeted insertion CRISPR mutagenesis can frequently result in some proportion of longer indels (**Supplemental Fig S9**). In particular, we have observed unintended insertions from lentiviral vectors used to introduce the guides and Cas9 (**Supplemental Fig S10**, (Chari et al. 2015).

Current tools primarily rely on either global mapping (CrispRVariants, AmpliconDIVider) (**Supplemental Tab S1**) that can have problems identifying the correct loci in the presence of a

larger insertion or have certain processing steps that are incompatible with longer events (see below for CrispRVariants). This mitigates primer dimer contamination problems (which can be identified by too large deletion gaps after alignments), but ignores bona fide large indels. These tools are therefore often unable to handle longer indels whether unintended or targeted. Long deletions are also a problem for some tools. For instance, CrispRVariants filters out any deletion that does not start or end within the gRNA complementary sequence plus a buffer of 5 bp. Any deletion spanning this region is ignored. This can be used as a strategy to filter primer-dimers, but also has the side-effect of ignoring any bona fide longer deletions. ampliCan uses a local alignment strategy that can detect these longer indels and a more realistic model of primer-dimer artifacts (**Supplemental Note S8**).

We noticed that in the Synthetic Dataset 2 from Lindsay et al. 2016 (CrispRVariants benchmark dataset) large indels (>10 bp) were disabled. To assess the capabilities of leading tools in handling longer indels we created Synthetic Dataset 4. We made three subsets: 1) with no indels > 10bp, 2) with a mix of indels by simply removing the line disabling longer reads in the Lindsay et al. script. 3) To check explicitly how tools handle experiments with planned shorter insertion of donor sequence we created a third scenario described as “insertions > 10bp” on the figure. 4) For completeness we also created a set of large deletions. ampliCan match the best competitors on the the set with no long indels and consistently outperform the other tools on the mixed set and the set only containing long indels (**Fig 2**, **Supplemental Fig S11**).

Supplemental Note S6: ampliCan consistently recovers the true HDR efficiency when faced with diverse donor templates

ampliCan takes into account the donor template and the original genomic sequence to define the set of events that corresponds to a correct HDR editing experiment, but allowing for some background sequencing noise (currently 3 mismatches by default). This is unlike CRISPResso, the other CRISPR tool that can handle HDR events, which do not model events but simply align reads against donor and original sequence picking the best-scoring instance. The advantage of ampliCan's approach is that it accounts for alignment imperfections in a more robust fashion, allowing for complex donor-amplicon relations and sequencing errors.

We designed a dataset for benchmarking the HDR calling capabilities of the most popular tools. Using the same loci as in **Supplemental Note S1** we tested 20 different donor templates for each of three kinds of donor types: with point mutations, insertions or deletions of variable length from 5bp to 70bp introduced into the amplicon sequences. We simulated 2000 reads with different levels of HDR efficiency rate (0, 33, 66, 90). In this benchmark set only ampliCan makes no errors (**Supplemental Fig S12**).

Supplemental Note S7: Visualization and aggregation of the complete activity of gRNAs

While the default alignment plot shows the most abundant reads across the expected cut site it doesn't provide an overview over all editing events. ampliCan therefore also produces multiple plots that aggregate and visualize editing events (**Supplemental Fig S14**). Unlike the alignment plots these show the complete activity of the gRNA allowing for comparison of gRNAs by

manual inspection. The pipeline in ampliCan treats forward and reverse reads separately which, after visualization, makes it possible to spot read-related problems immediately (**Supplemental Figs S21, S22**, **S23**). In addition, ampliCan provides meta plots that aggregate information across groups of experiments allowing for visualization of deletions, mismatches or insertions across groups of gRNAs, amplicons or any other set. This can for instance show the combined activity of a single guide across multiple experiments.

ampliCan builds on top of ggplot2 (Wickham 2016) package and provides higher level functions that automatically group event data (eg. collapse on start and end of deletion) and plot results. Users can extend those plot objects and treat them like any other ggplot2 object. ampliCan supports multiple types of meta plots to facilitate comparison of not only the gRNAs, but also any group that a user wants e.g. barcode, amplicon, type of treatment (**Supplemental Figs S3**, **S17**, **S18**).

Supplemental Note S8: Filtering of noise

Multiple sources of noise can confound the estimation of cut rates, low quality reads, primer-dimers, PCR off-target amplification and sequencing artifacts. ampliCan has three filters to remove noise from different sources: 1) low quality reads, 2) primer-dimers, and 3) erroneously assigned reads and sequencing artifacts.

Low quality reads

ampliCan offers basic read quality overview with the use of ShortRead (Morgan et al. 2009) package and filters for minimum base quality (default: 0) in a read, average base minimum quality (default avg min: 30) and the presence of ambiguous (N) letters.

Primer-dimers

Filtering primer-dimers is a balance between getting rid of erroneous reads and allowing for longer deletions. For instance, CrispRVariants ignores all alignments with deletions larger than 33 bp (the guide plus a buffer of 5 bp) and is frequently unable to map long insertions (**Fig 2B**, **Supplemental Fig S11**). This effectively removes all primer dimers, but also ignores any bona fide longer indels. ampliCan instead tries to estimate the likely length of a primer dimer deletion by taking the length of the amplicon, subtracting the primer lengths with a small buffer (30 bp) to arrive at maximally allowed deletion length. This results in a more realistic estimate of the length of artificial deletions that would result from primer-dimers. As an example, for an amplicon of size 150 with primers of length 20, the maximum deletion length would be 80 bp (150-(2*20+30)).

Erroneously assigned reads and sequencing artifacts

An assumption of ampliCan is that a CRISPR editing event will result in a low number of indel events resulting in a good alignment with few discrepancies to the reference sequence or (if available) the control experiment. To accomplish this ampliCan uses a two dimensional clustering method based on sequence alignment score and sequence alignment indel events to filter out erroneous reads and sequencing artifacts. This takes all alignments and performs k-means clustering with different 1-3 clusters. It then uses the silhouette criterion to determine the optimal number of clusters. In the case of 1 cluster, all reads are either edited or perfectly matching the reference/control. In the case of 2 you have both edited and unedited reads. In the case of 3 clusters, cluster with center that has the biggest number of events and lowest alignment score (in normalized relation on 0-1 scale) means that you in addition have a group

of sequencing artifacts or reads that poorly align to the loci (example in **Supplemental Fig S19**).

ampliCan provides plots that shows the impact of each of the filtering steps, across the whole library for read quality (**Supplemental Fig S16**) and for each experiments for primer-dimer and assignment/artifacts issues (**Supplemental Fig S15**).

Supplemental Note S9: Read assignment

ampliCan assigns reads to the respective experiment by matching primers used in the amplification of the loci. These region should be immutable and match the reads since an indel spanning a primer would either result in failure to amplify the locus or be “corrected” by the primer when it amplifies the target site. However, since small sequencing and primer synthesis errors could potentially occur in the primer part ampliCan allows for up to 2 mismatches (user customizable) between the primers and reads. During this process it is possible that some reads will be unassigned and not match any of the experiments. While these reads are typically noise from the high-throughput nature of the sequencing experiment, they could in some cases be helpful in troubleshooting failed experiments. ampliCan therefore provides human readable alignments of the top 5 most abundant forward and reverse read pairs aligned to each other (**Supplemental Fig S24**). In some cases these correspond to off-target PCR amplicons and close homologous regions as well as errors in the specification of the experiment.

Supplemental Figures

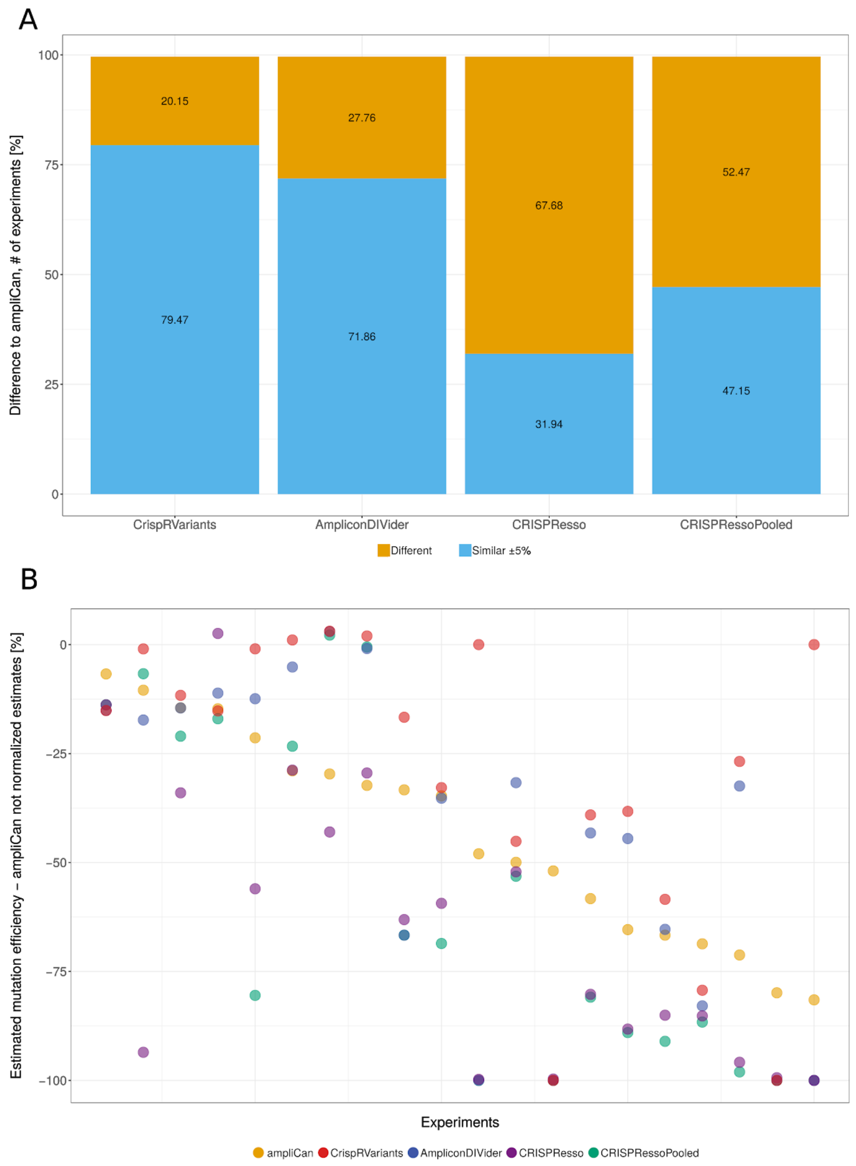

Supplemental Fig S1. Comparison of leading tools on real CRISPR experiments. **A.** Summary of differences between ampliCan and other tools. CrispRVariants reports similar editing efficiencies to ampliCan in ~80% out of 263 experiments. The remaining experiments are due to controls (~5%) and processing (~15%) **B.** Experiments (x axis, sorted) where estimated mutation efficiency differs by at least 5% from non-normalized data. y-axis shows differences in relation

to non-normalized ampliCan estimates. Differences between tools predictions staying above line created by ampliCan prediction are likely to be due to lack of normalization, while the predictions below the ampliCan are likely due to the alignments, processing and filtering of data.

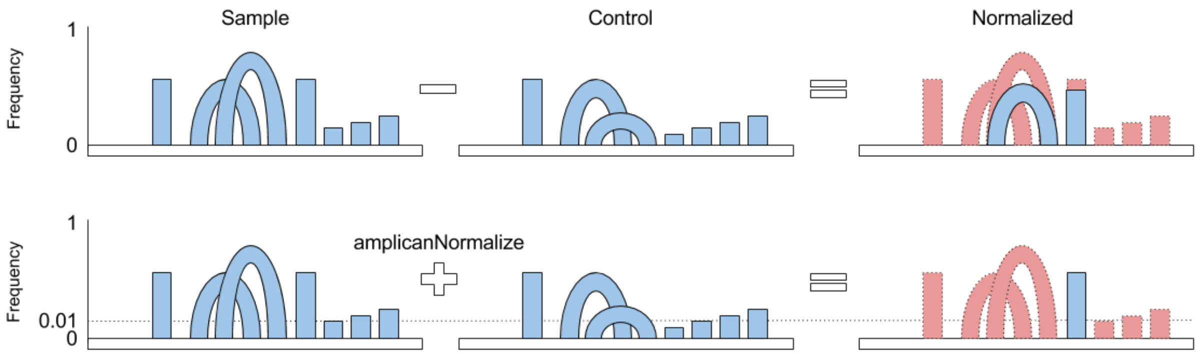

Supplemental Fig S2. Two common methods for normalization using controls. In the first, the frequencies from events in the control sample are subtracted from the frequencies in the treated sample (subtraction method). In the second method, all events occurring in the control above a frequency threshold (ampliCan default: 1%) are removed from the treated sample. The latter is more robust to stochastic differences.

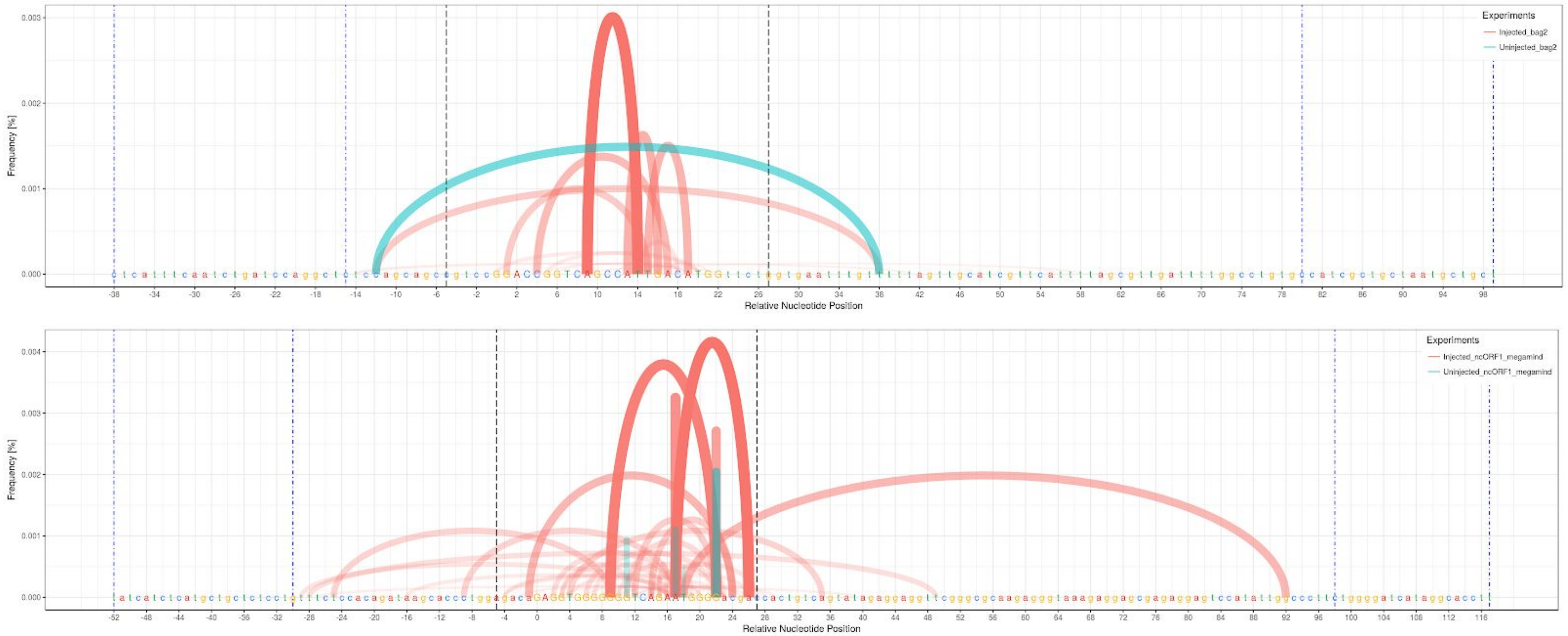

Supplementary Fig S3. Deletion plots produced by ampliCan for two experiments showing deletions in Cas9 injected (red) versus control (cyan) samples. The archs indicate deletions (x-axis, start to end of arch) present in the samples at a frequency indicated by the y-axis and transparency. The blue, vertical dotted lines shows the start and end of the primers. In the top panel experiment normalization using the subtraction method would filter the big deletion also present in the control, but it would not completely get rid of the 1bp long deletion in the lower panel experiment. Both cases would get expunged with the default ampliCan normalization.

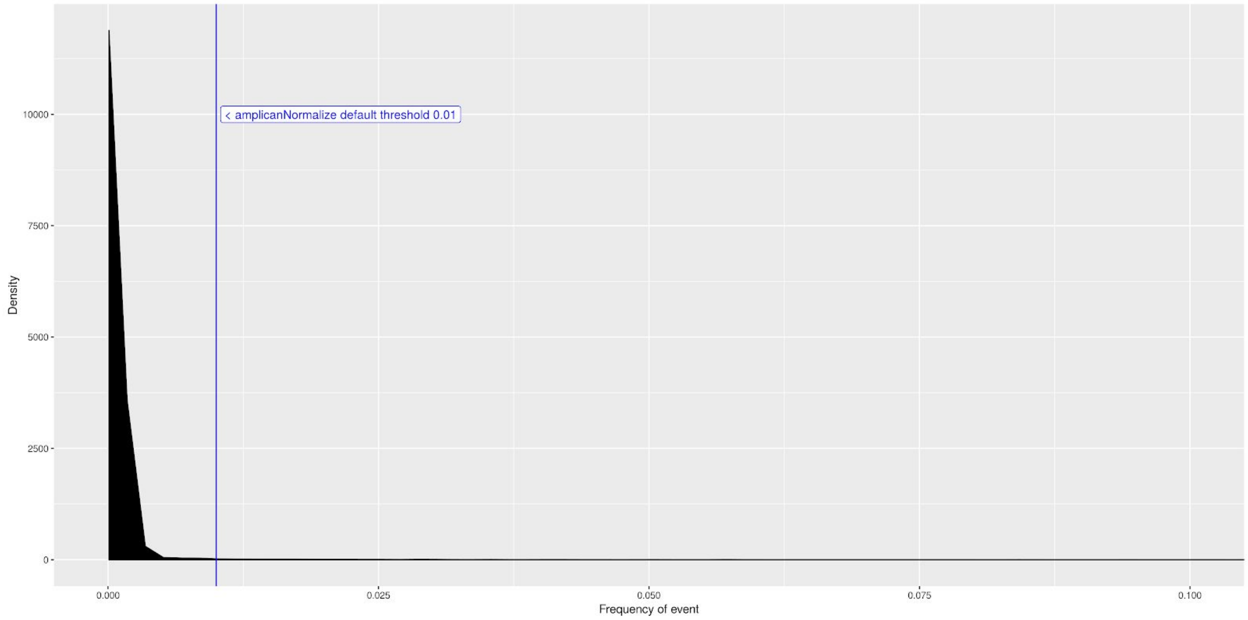

Supplemental Fig S4. Distribution of the frequency of events in the control sample of real experiments. The vast majority are low frequency events likely to be technical and experimental artifacts. ampliCan uses a threshold to exclude these so they are not considered for normalization. The default threshold is a frequency of 0.01 which excludes more than 99% of these events as noise. These low-abundance events are therefore not considered when normalizing the target loci. If the user know the level of variance expected in the controls they can raise the threshold for increased stringency.

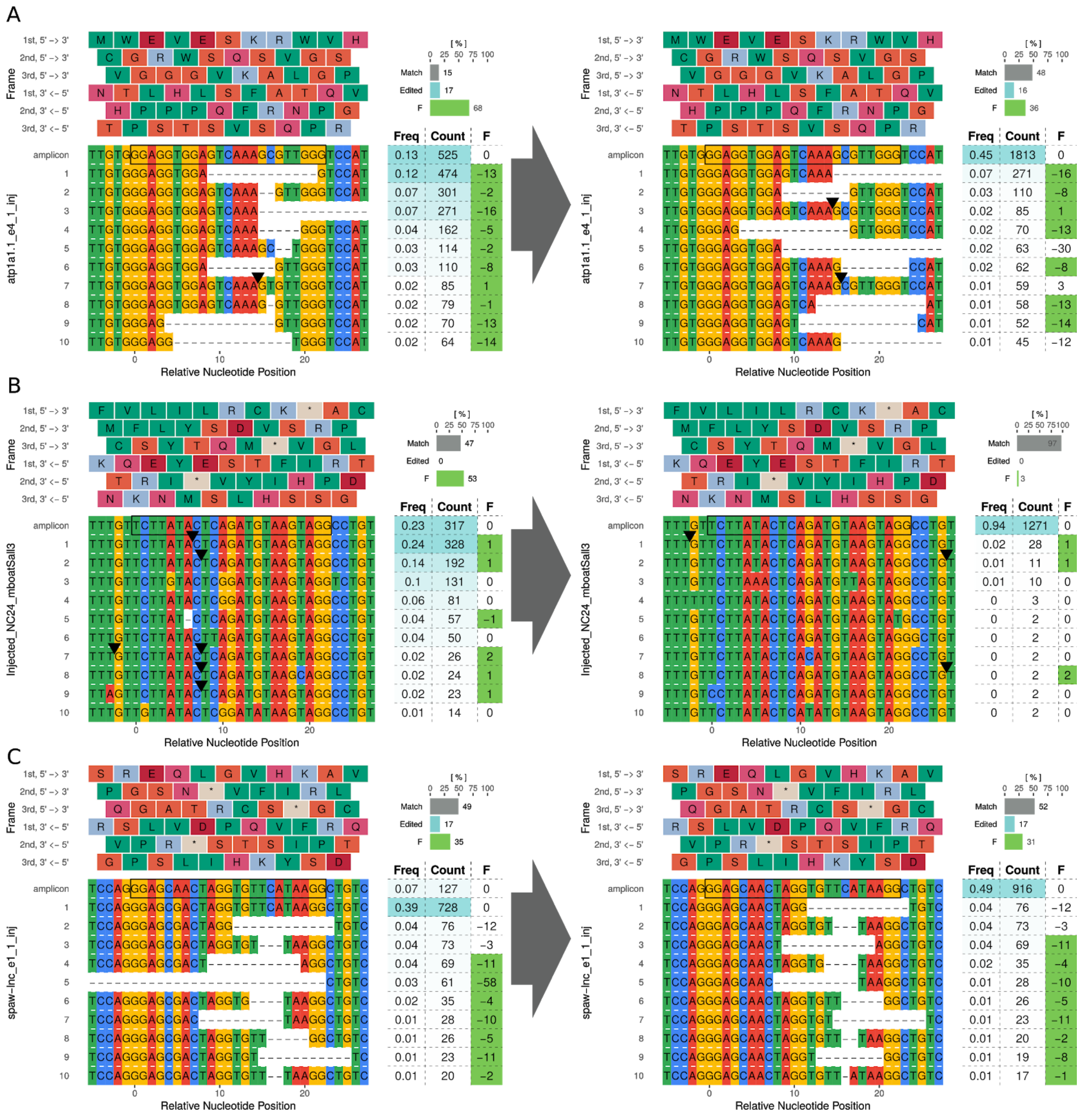

Supplemental Fig S5. Example of normalization with controls on 3 real experiments. In heterogeneous data it can be challenging to derive the true mutation profile. **A.** ampliCan automatically removes wild type mutations and reduce the number of frameshift inducing mutations from 68% to 36%. **B.** A large number of insertions could be mistaken for CRISPR activity reducing the total indel rate from 53 % to 3 %. **C.** A large fraction of reads (39%) carry a G instead of an A. This mismatch also occur at high frequency in the control and is therefore

assumed to represent genetic variance. The mismatch is ignored and the reads are merged with the counts of the other unedited reads.

```
GTGGTCAGGGGGCGTTTATTCTGCCGG--ACT---ATACCCT
||||||||||  |     |  ||  ||  |||   |||||||
GTGGTCAGGGAAC-----TGGTGGAGGTCACTGGGATACCCT
```

```
GTGGTCAGGGGGCGTTTATTCTGCCGGACT----------------ATACCCT
||||||||||                 |||                |||||||
GTGGTCAGGGA----------------ACTGGTGGAGGTCACTGGGATACCCT
```

Supplemental Fig S6. Alignments dependent on multiple parameters and are often a tradeoff between the cost of gaps and the cost of mismatches. The top alignment shows a typical alignment for many aligners that are not optimized for genome editing (gap opening 10, gap extension 2, match 5, mismatch -4 and no end gap penalty). The bottom alignment, illustrates the result with ampliCan parameters (gap opening -25, gap extension 0, match 5, mismatch -4 and no end gap penalty) that are optimized for few events as is expected for CRISPR activity.

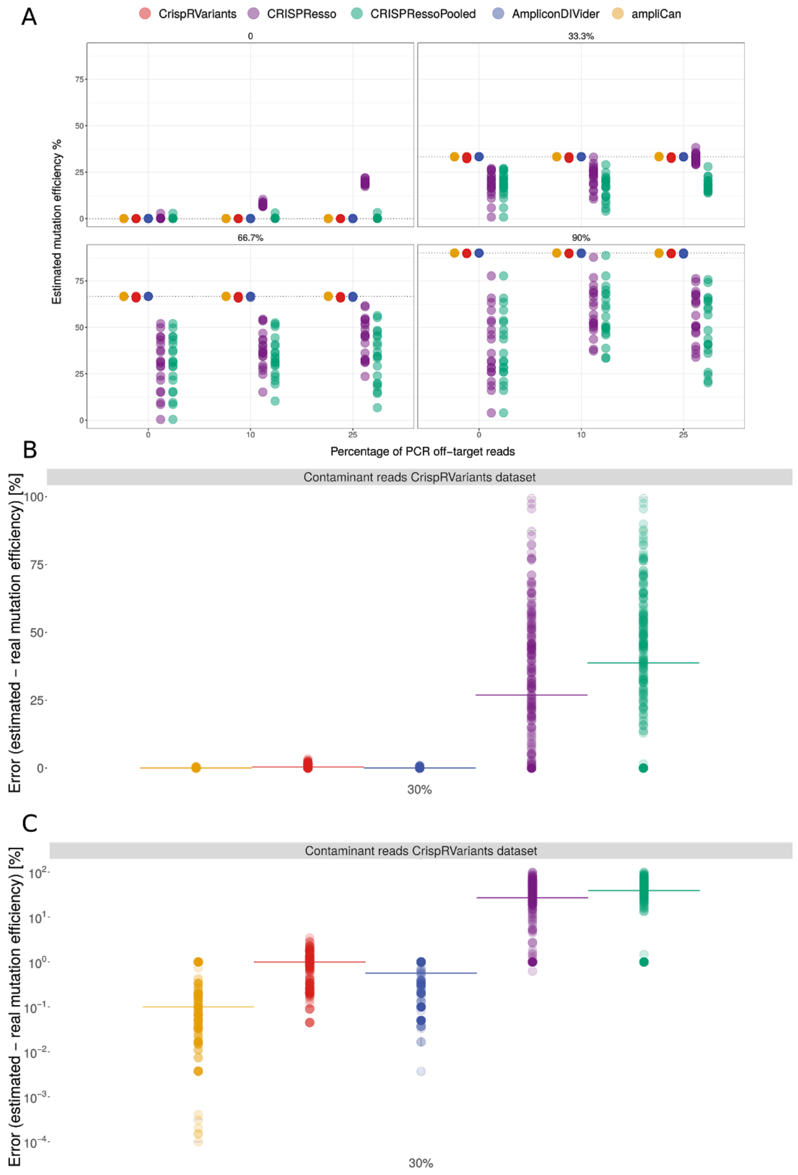

Supplemental Fig S7. ampliCan outperforms CrispRVariants on its own synthetic dataset. **A.** Performance of leading tools when facing different mutation efficiencies and fractions of contaminating reads. The sets are obtained from the benchmark data in (Lindsay et al. 2016) (CrispRVariants), Synthetic Dataset 2 (Supplemental Fig. 15 in (Lindsay et al. 2016). Each dot in the plot correspond to the estimated mutation efficiency calculated by a single tool for a single experiment, while the dotted line shows the true mutation efficiency The fraction of contaminant reads varies on the x-axis. **B.** Error rates (**C.** log10 scaled) of the same experiments. The median error is indicated by the horizontal line.

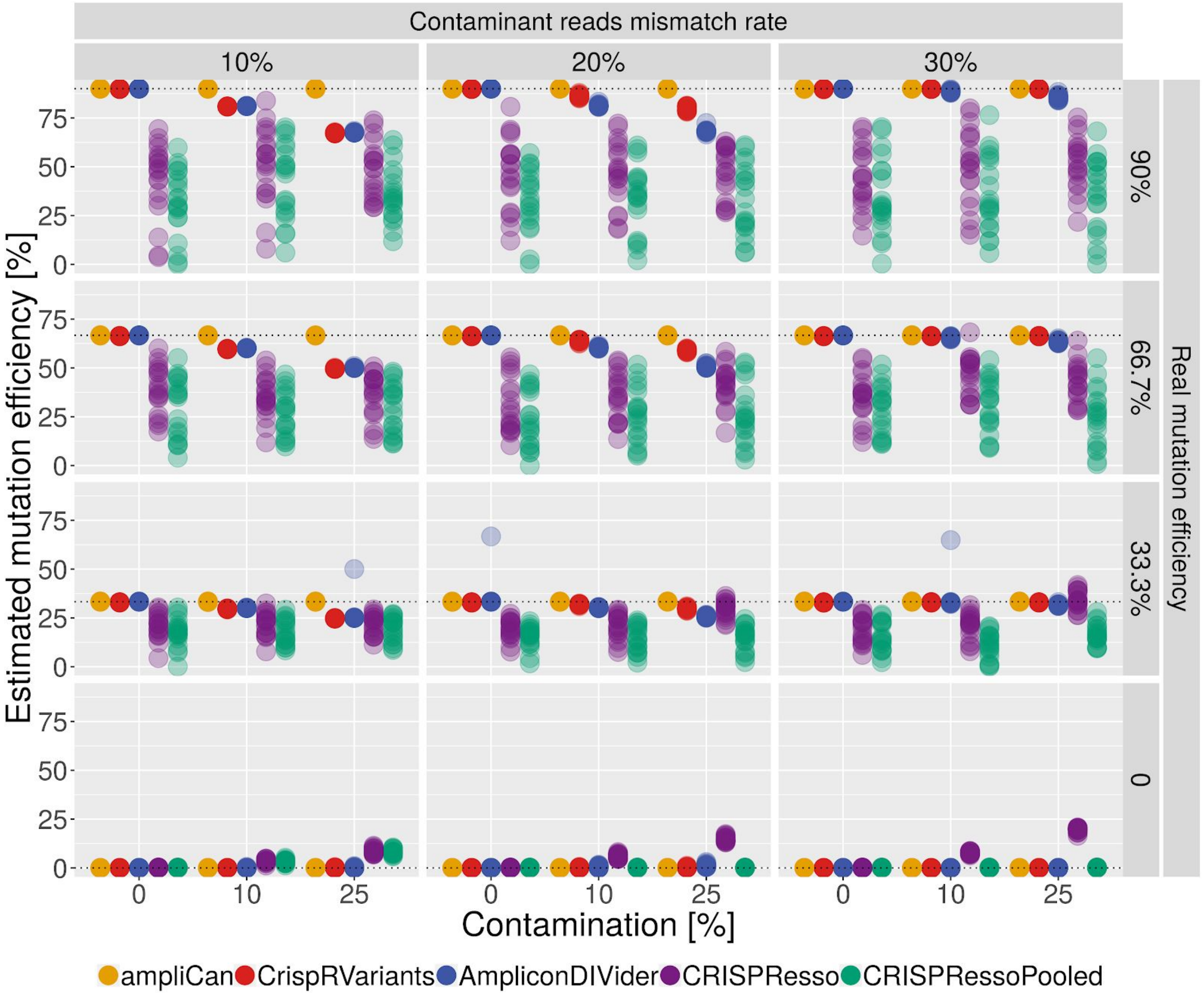

Supplemental Fig S8. The data from left panel of Fig. 2 split by the true mutation efficiency and with a baseline of 0% true mutation efficiency added for comparison. The dots shows estimated mutation efficiency by leading tools on reads with increasing contamination (as 0, 10 and 25 percentage of all reads) with different mismatch rate of the off-target reads (10%, 20%, 30%). Each point corresponds to one experiment. True mutation efficiency is indicated with dotted lines, and labelled to the right of the charts. Contamination is simulated by introducing random mismatches (Contaminant read mismatch rate) in reads mapping to the loci, similar to the benchmark in (Lindsay et al. 2016). The mismatch rate is indicated at the top. Only ampliCan shows robustness to the whole range of different mismatch rates and mutational efficiencies.

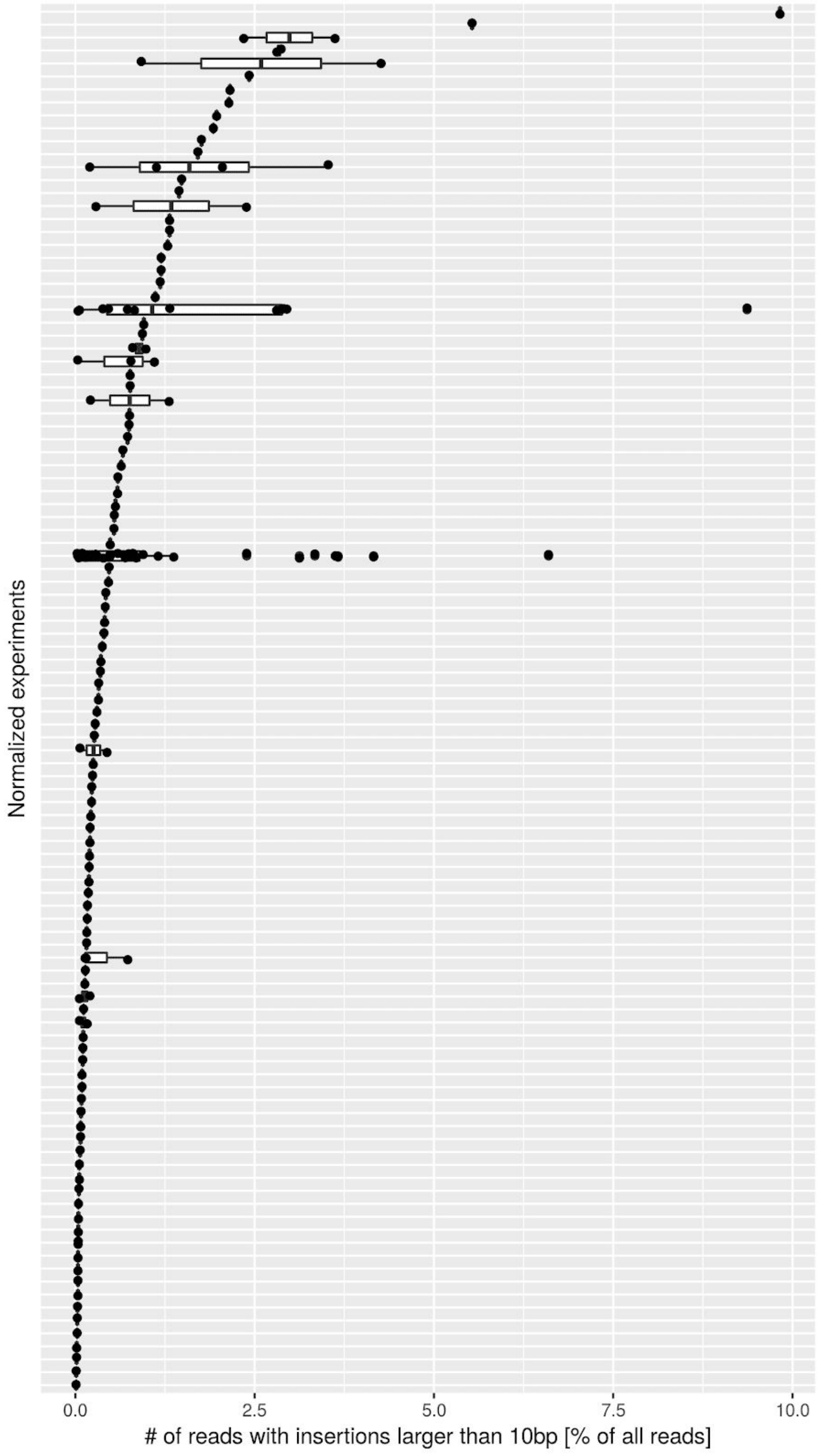

Supplemental Fig S9. Fraction of reads having indels greater than 10 bp across 176 experiments. Each dot represents one experiment and are grouped in rows by having the same gRNA

(replicates). All experiments are normalized using wild type controls ensuring that these are real indel events. The higher mean for some of the replicated experiments suggests that some gRNAs have a higher chance of resulting in long indels.

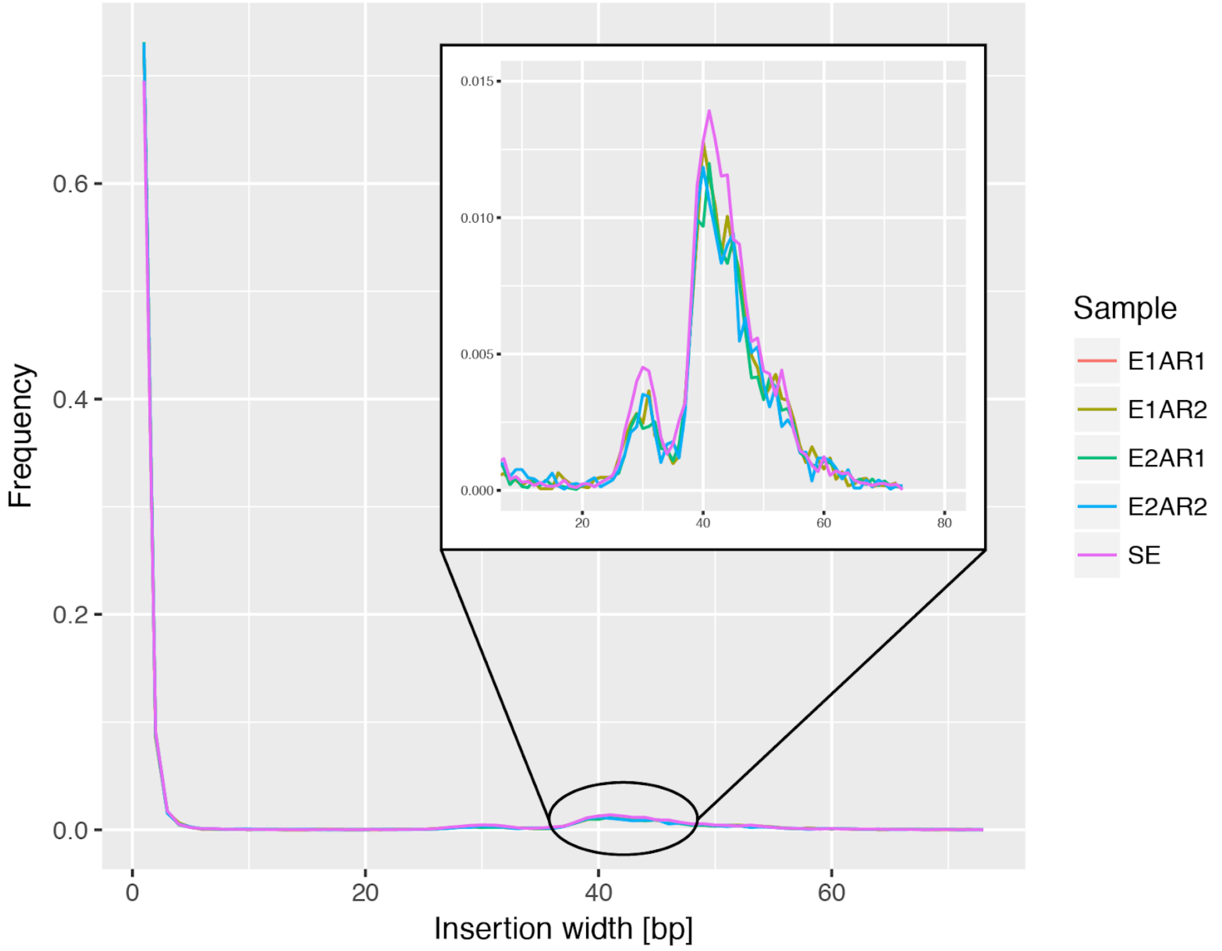

Supplemental Fig S10. Distribution of insertions width for lentiviral samples on Chari et al. 2015 datasets. Shows the proportion (y-axis) of reads with a given insert length (x-axis). The insert shows a population of unintended larger insertions. Around 90% of these originate from the lentiviral vector used to transfect the cells.

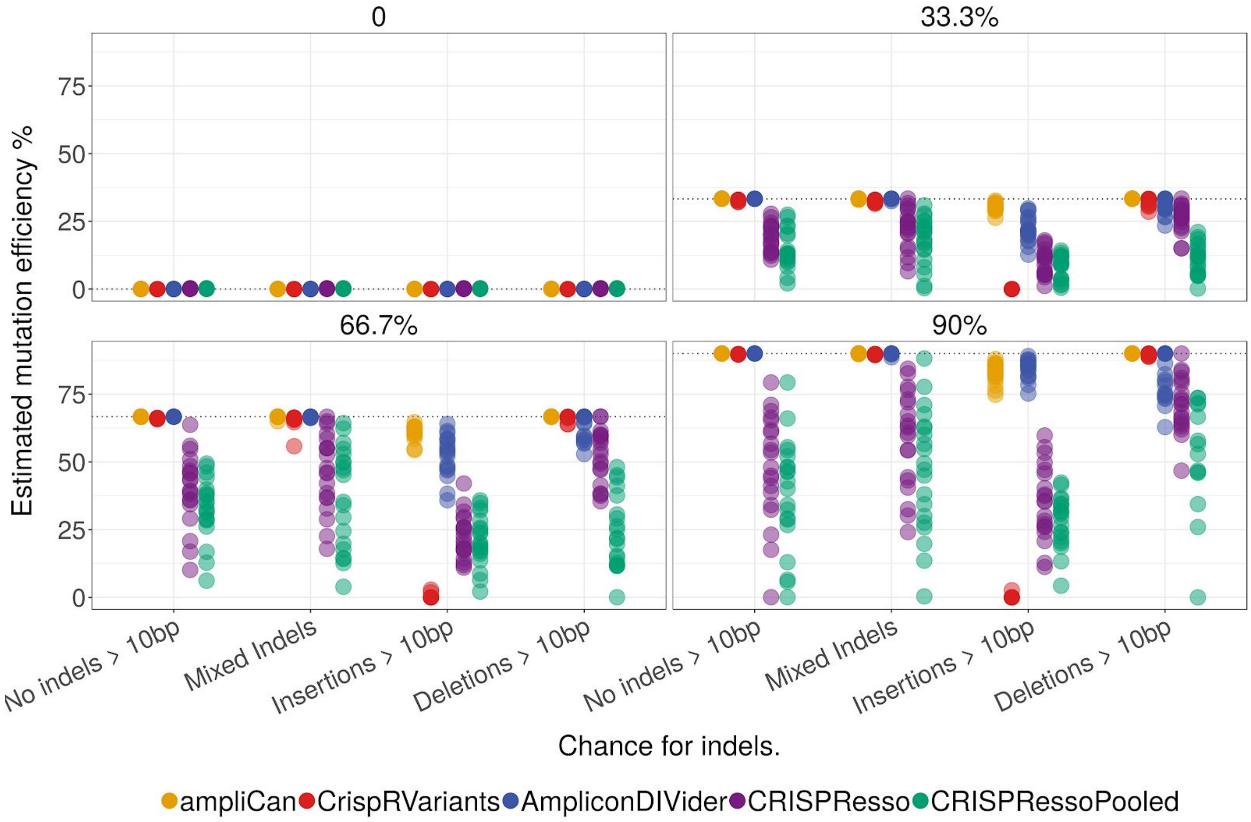

Supplemental Fig S11. Performance of leading tools grouped on simulated data with large indels. Some tools find larger indels challenging. ampliCan consistently returns values closest to the real mutation efficiency.

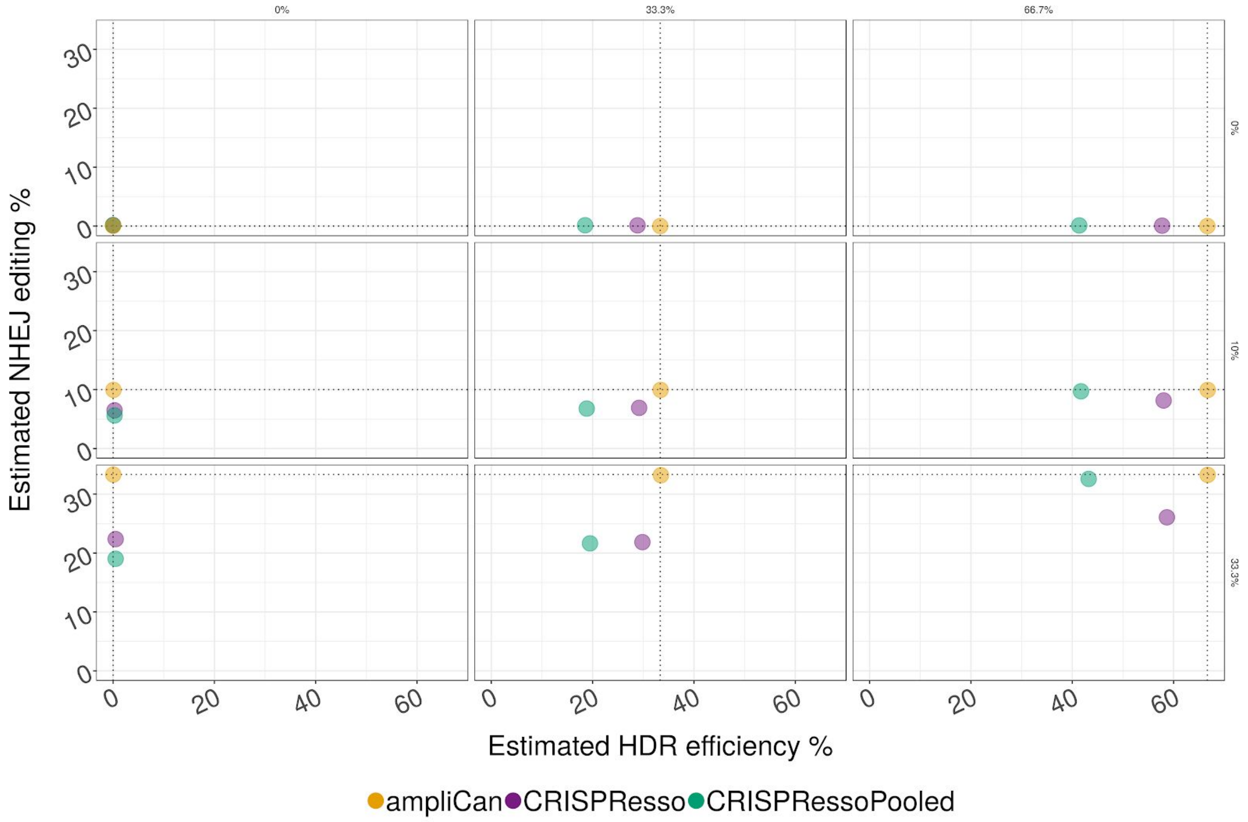

Supplemental Fig S12. Performance of ampliCan, CRISPResso and CRISPRessoPooled on simulated data with variable donor templates, variable true HDR read rate (0, 33, 66 %) and variable NHEJ editing (0, 10, 33 %). The dotted lines represents the real HDR (vertical) and NHEJ (horizontal) efficiencies and their intersection the correct estimate of both. The performance on 20 donors of different types (mismatch, insertions and deletions) and of variable length (from 5-70bp) were averaged into a single dot for visibility. While ampliCan handles donor templates with high accuracy, CRISPResso has difficulties with estimation in situations where alignment is imperfect.

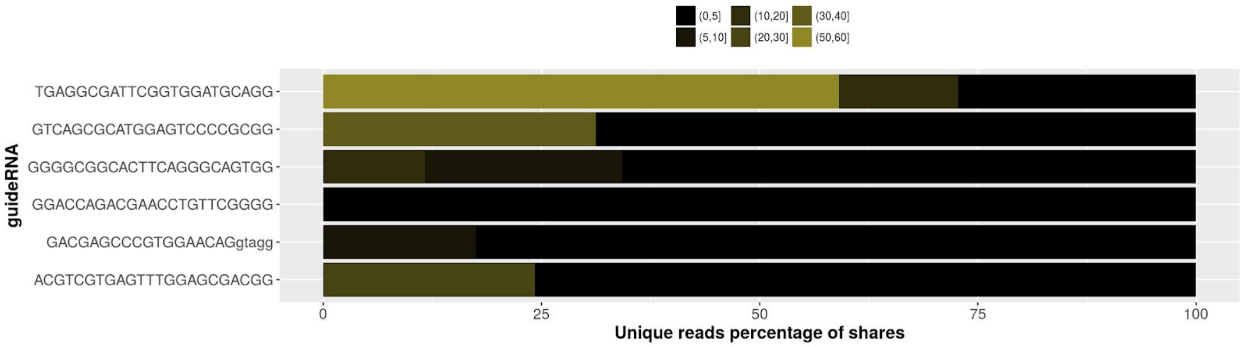

Supplemental Fig S13. Example of a heterogeneity plot produced by ampliCan. In this, identical reads are collapsed together and grouped by gRNA. A stronger shade of yellow indicates a large group of homogeneous reads. This plot can give insight into the heterogeneity of the outcome. A high level of heterogeneity can indicate sequencing problems or mosaicism. Reads can also be aggregated on experiments or any other user selected grouping rather than guides.

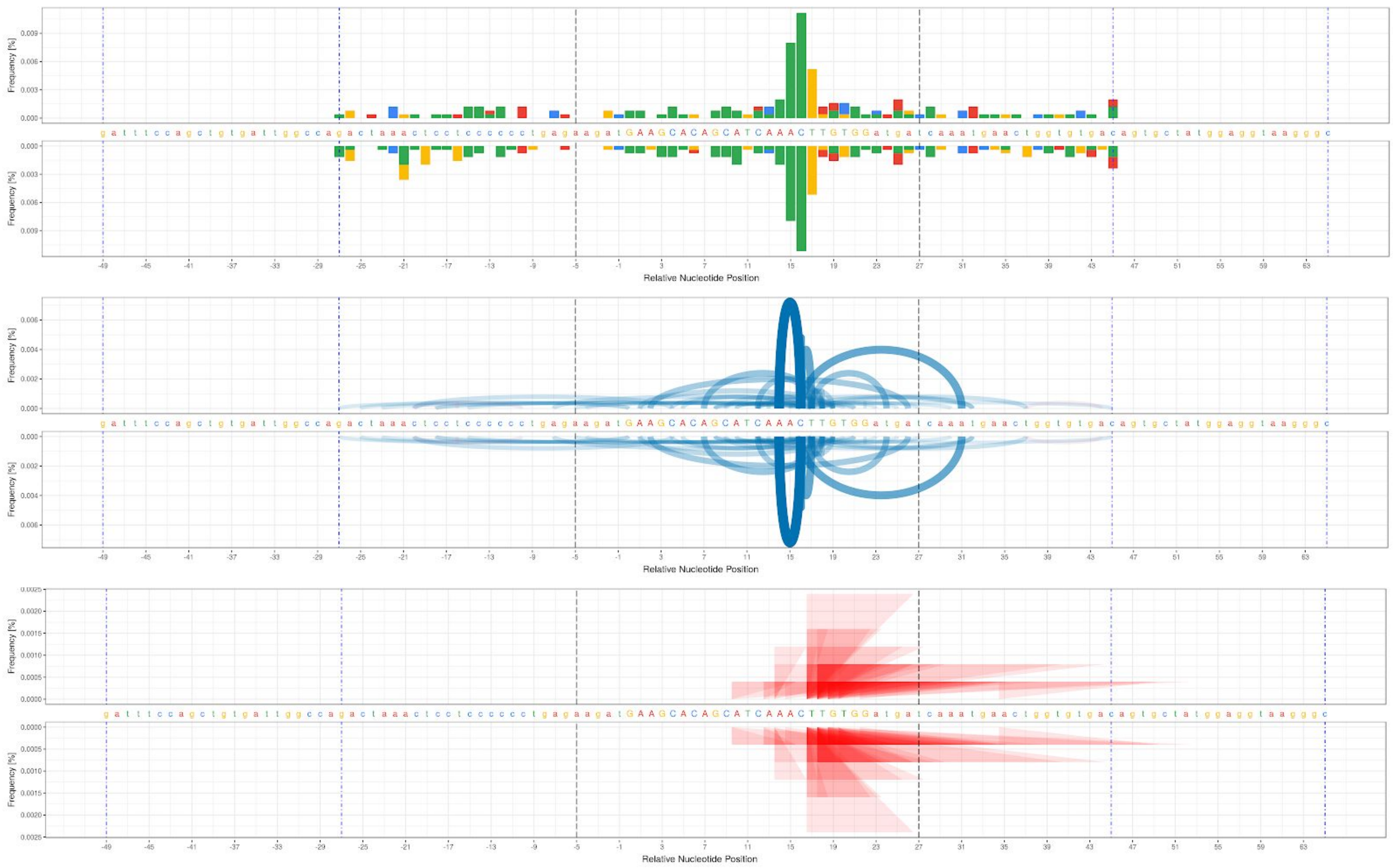

Supplemental Fig S14. A number of plots are produced by ampliCan to investigate editing events: mismatches to the reference (**A**), aggregated deletions (**B**) and aggregated insertions (**C**). Dashed blue vertical lines indicate where the primers start and end. The black

dashed lines mark the region around the cut site where we require a mutation event to overlap to be considered a CRISPR event. The top half of the plots shows the forward reads, while the bottom shows the reverse reads. The amplicon sequence is shown in the middle, with uppercase letters indicating the gRNA. The y axis shows the efficiency of the events summed over all reads. This allows for immediately discerning which bases are edited above the background noise from sequencing.

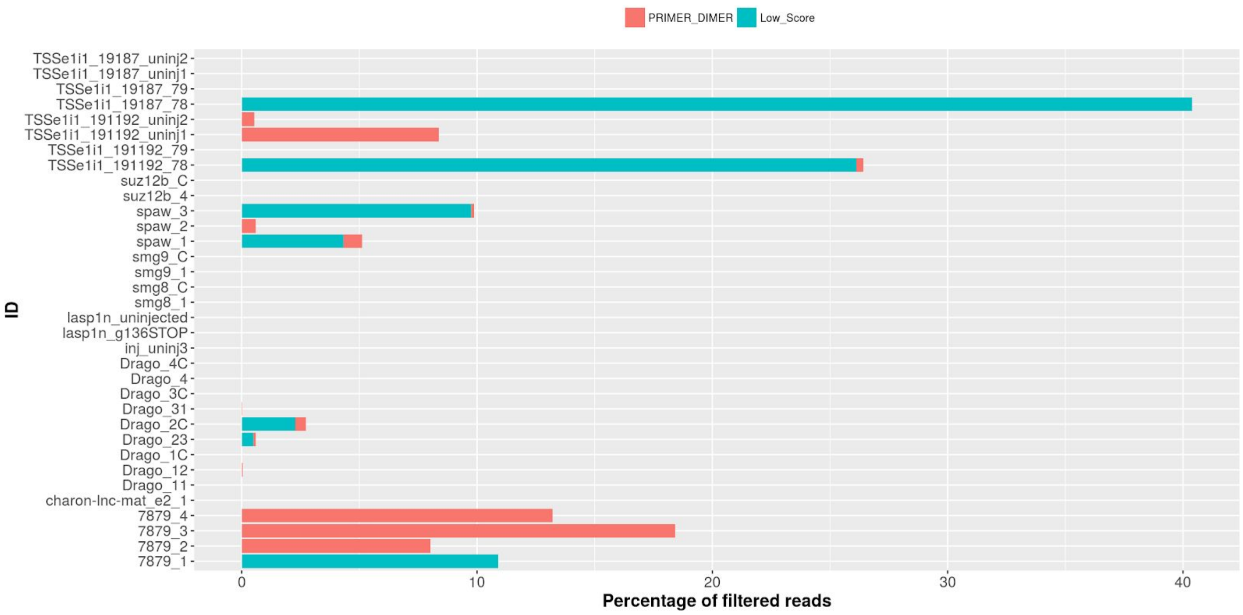

Supplemental Fig S15. Example of bar plot showing fraction of reads that were filtered out of the experiments. Red bars correspond to a primer-dimer filter, and blue bars indicate low quality reads.

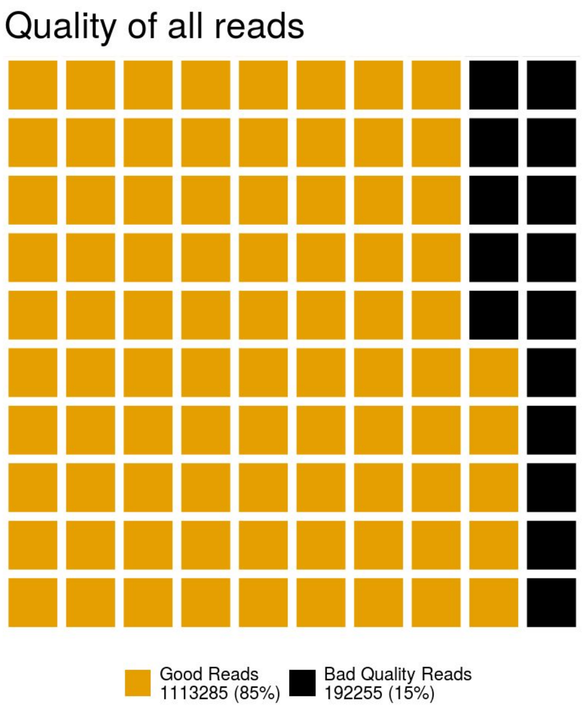

Supplemental Fig S16. Waffle plot of the quality of reads across all experiments.

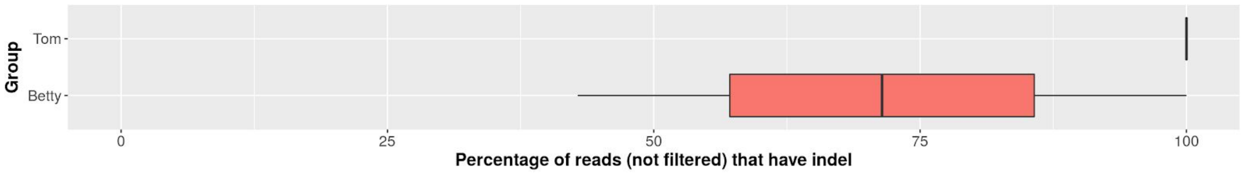

Supplemental Fig S17. Example of comparison plots in ampliCan test data where Indel rates are grouped by the researcher performing the experiments. These can be grouped on any user specified criteria.

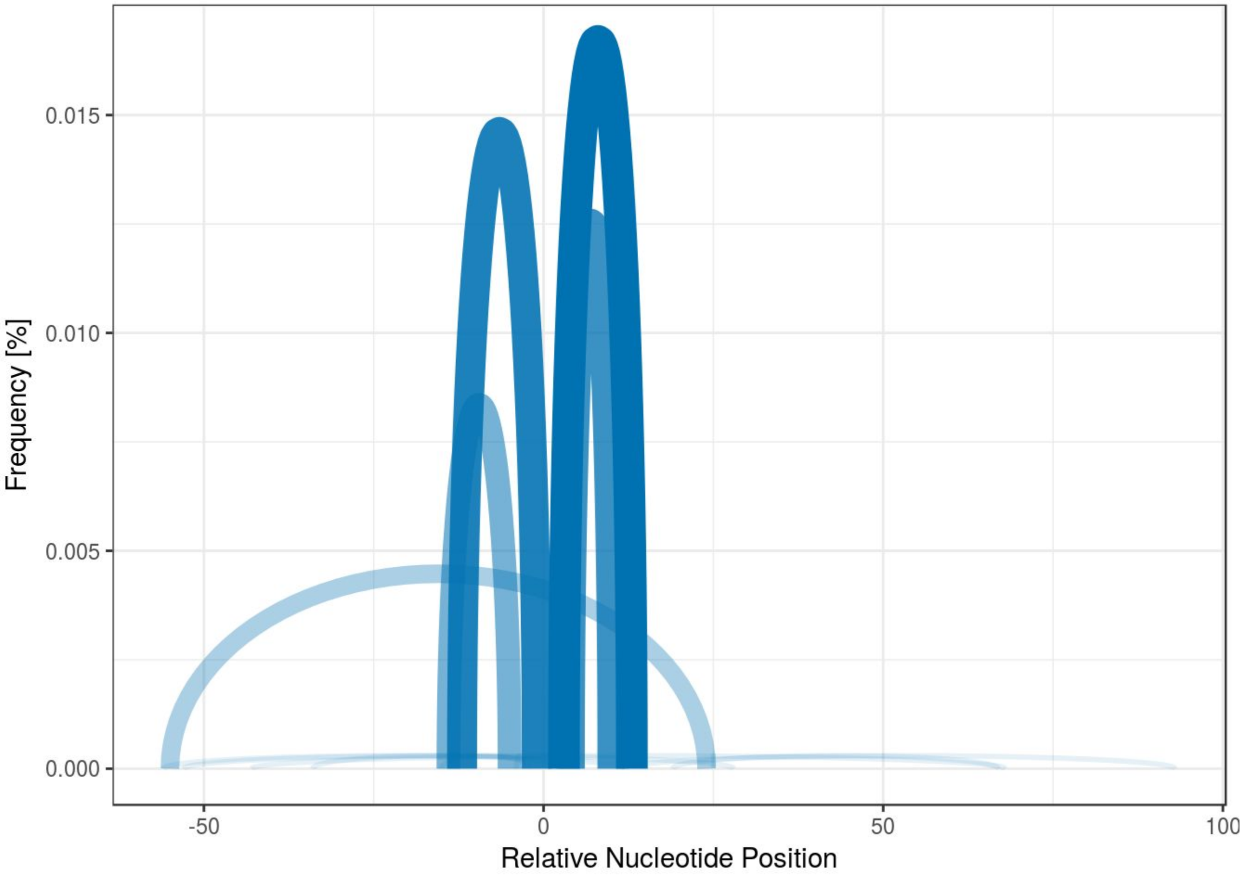

Supplemental Fig S18. Example of a deletion metaplot produced by ampliCan summarizing multiple experiments. Here, an aggregation of editing events from many experiments (many targets) using the same gRNA is presented giving an overall gRNA cut profile. Position 0 is relative to the first 5' base of gRNA.

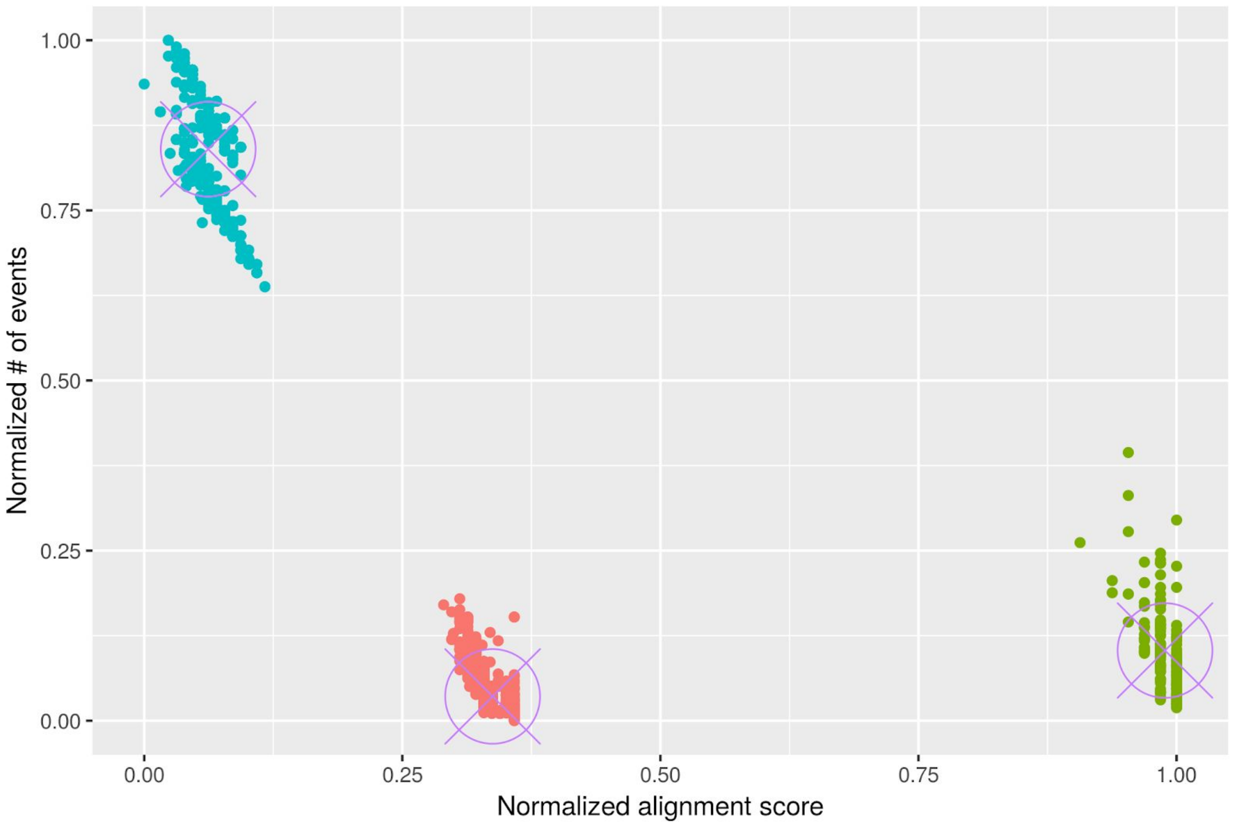

Supplemental Fig S19. Example of k-means clustering of reads during filtering of contaminant reads. A low alignment score (x axis) combined with a high number of events (y axis) indicate erroneous reads. Silhouette criterion is used to determine whether data should be clustered into two (read with no edits and reads with editing events) or three clusters (a noise cluster). In the case of three clusters, the cluster with its center (purple) closest to the upper left corner is filtered.

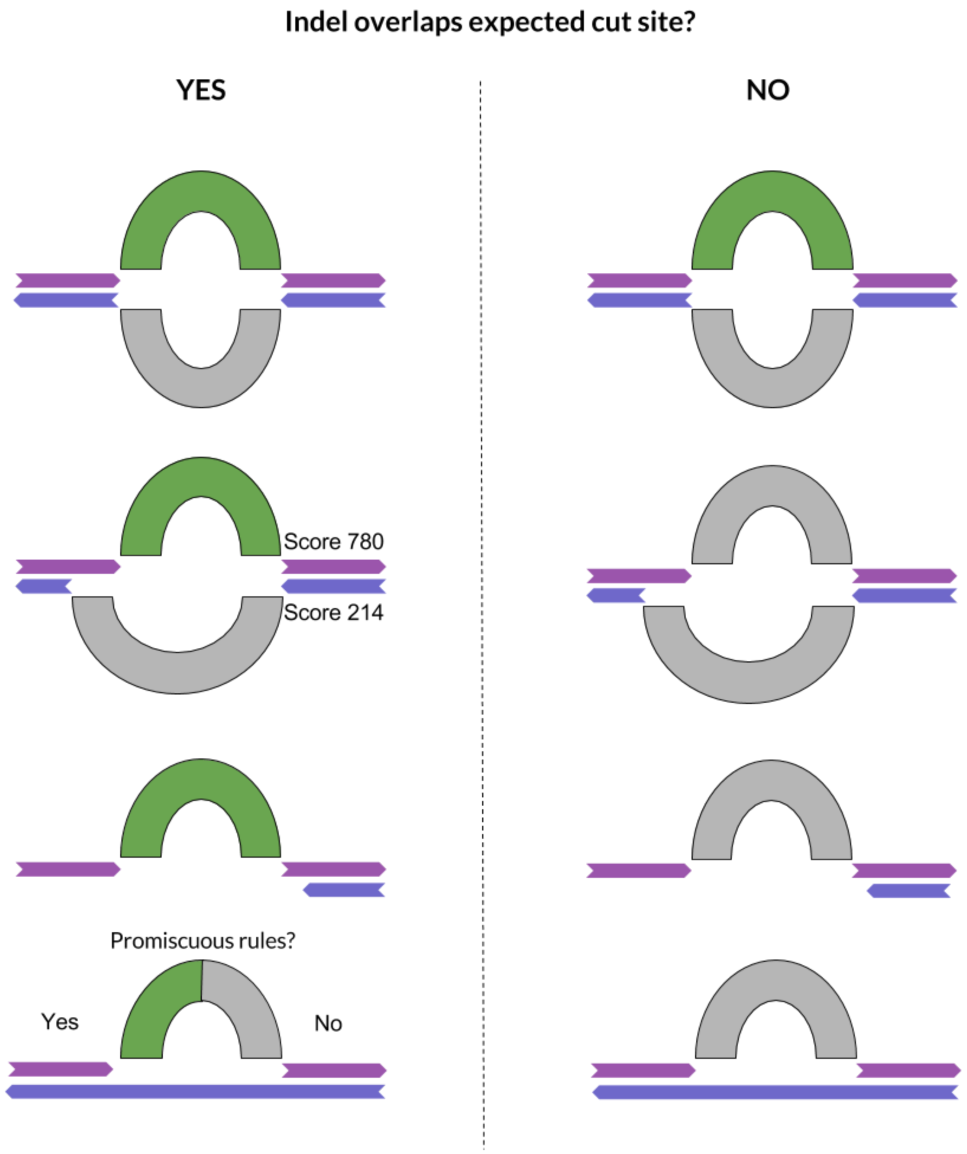

Supplemental Fig S20. The paired-end read consensus rules for ampliCan. Events marked in green are considered real cut sites while those in gray are not. When reads from forward (purple) and reverse (blue) reads are in agreement there is a consensus (top row). When two reads overlap, but disagrees the event from the strand with a higher alignment score is used (row 2). In situations where an event is only covered by one read, that read is preferred (row 3). In rare cases where there are events on one strand and the other has continuous alignment ampliCan allows users to define the behaviour (default is promiscuous rule enabled).

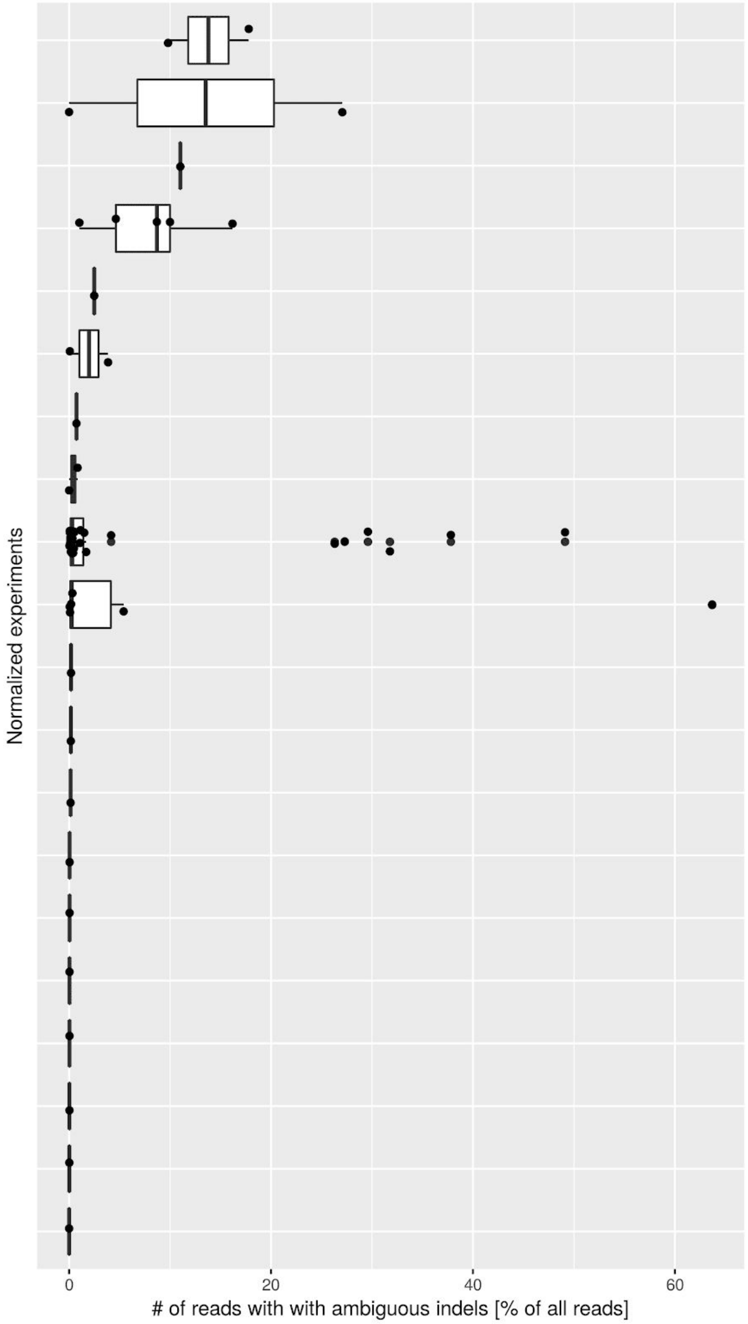

Supplemental Fig S21. Percentage of reads with ambiguous indels caused by disagreement of forward and reverse reads. ampliCan consensus rules help to mitigate mis-estimation that could arise from these events.

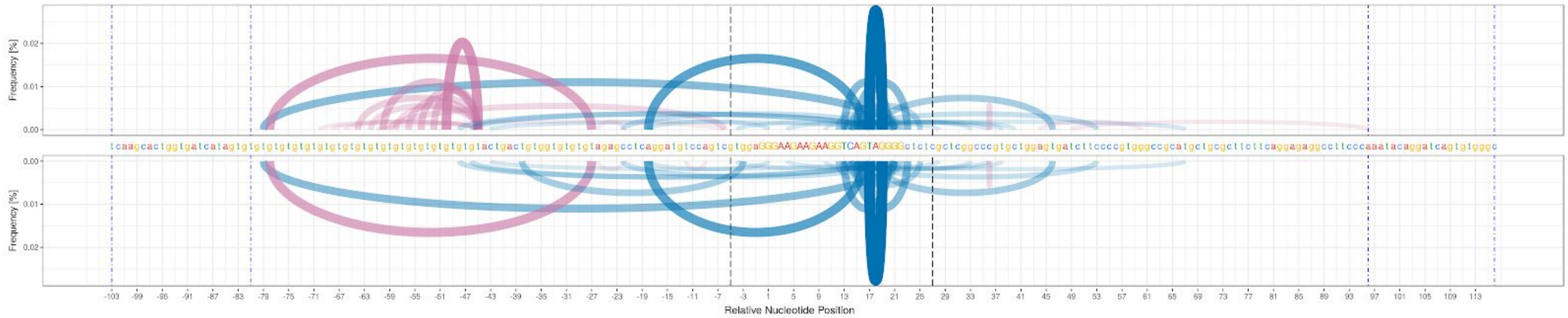

Supplemental Fig S22. ampliCan evaluates forward and reverse reads separately which allows users to spot potential problems. Here, purple colour indicates cuts outside of the gRNA expected cut site, a large majority of those reads comes from problematic alignments, where forward read and reverse read are in disagreement.

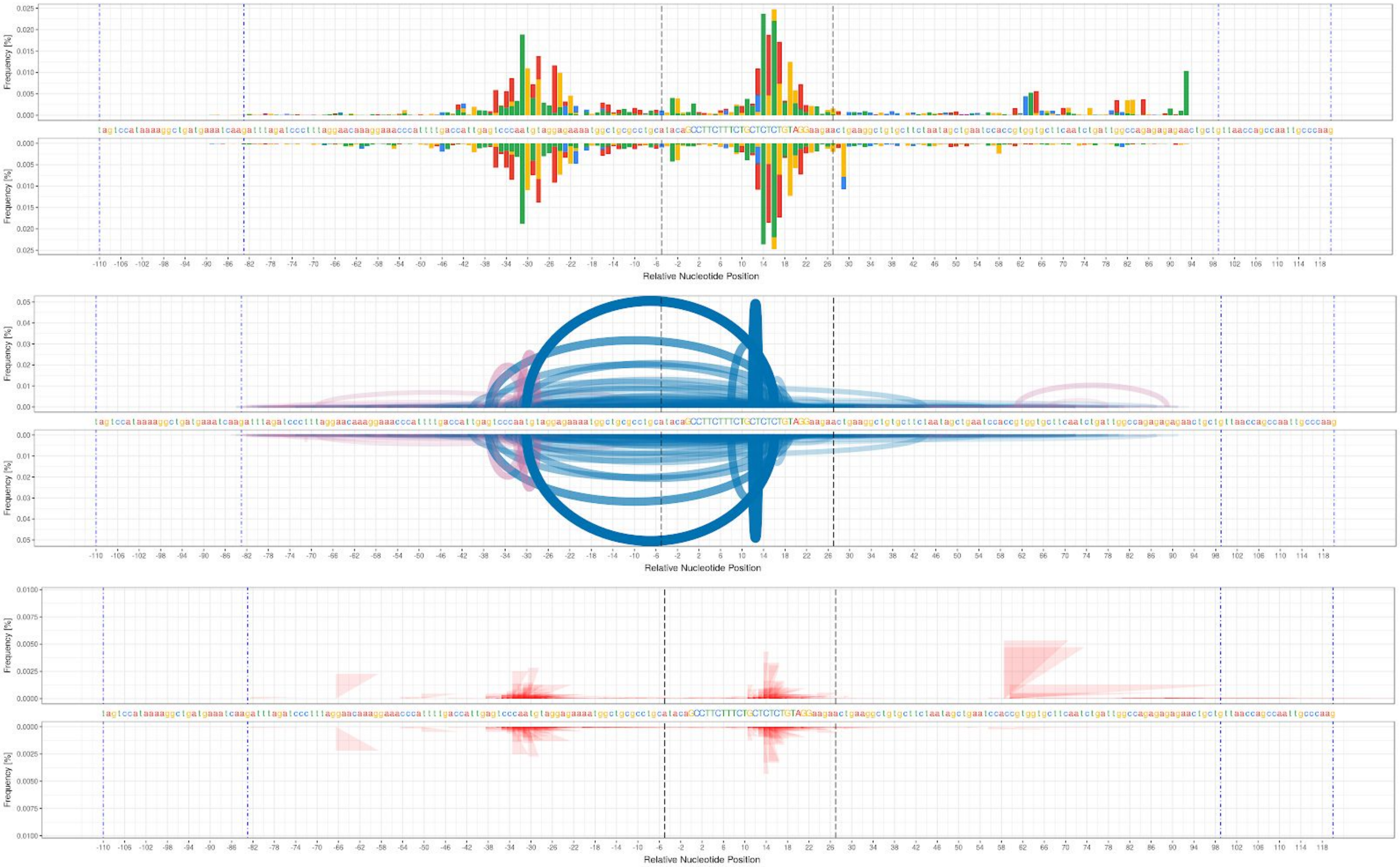

Supplemental Fig S23. Deletion plot (**top**), insertion plot (**middle**), mismatch plot (**bottom**). This presents a case where the user might have mischaracterized their own experiment which can be spotted with these plots. Here nickases were used, but only one of the gRNAs was

specified in the configuration file. A bimodal distribution of mismatch events and insertions is the result. A quick examination allows the user to realize the expected cut site should be extended to include the second gRNA, enabling more precise mutation efficiency estimation.

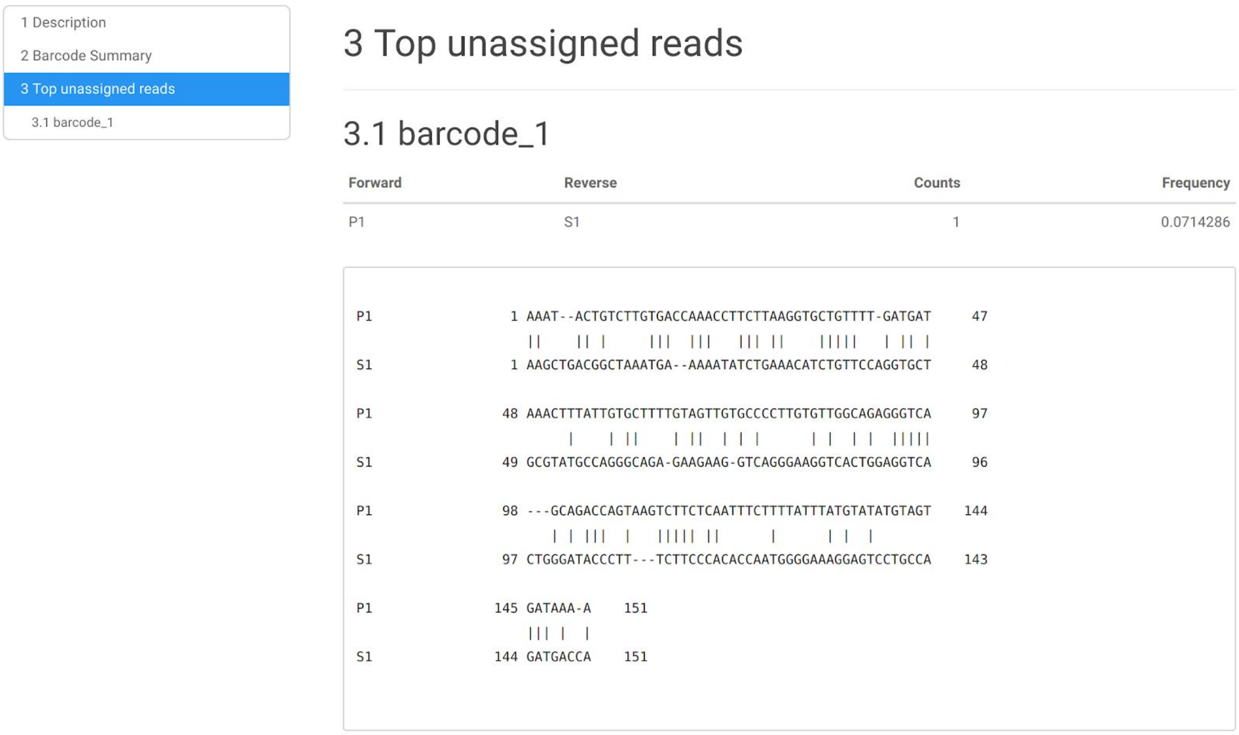

Supplemental Fig S24. Screenshot of the example barcode report, top unassigned read section. Human readable alignment of forward and reverse reads of the top most frequent unassigned reads is presented. Huge fragmentation and poor alignment suggest contamination, while low frequency indicate proper specification of the primers in the config file.

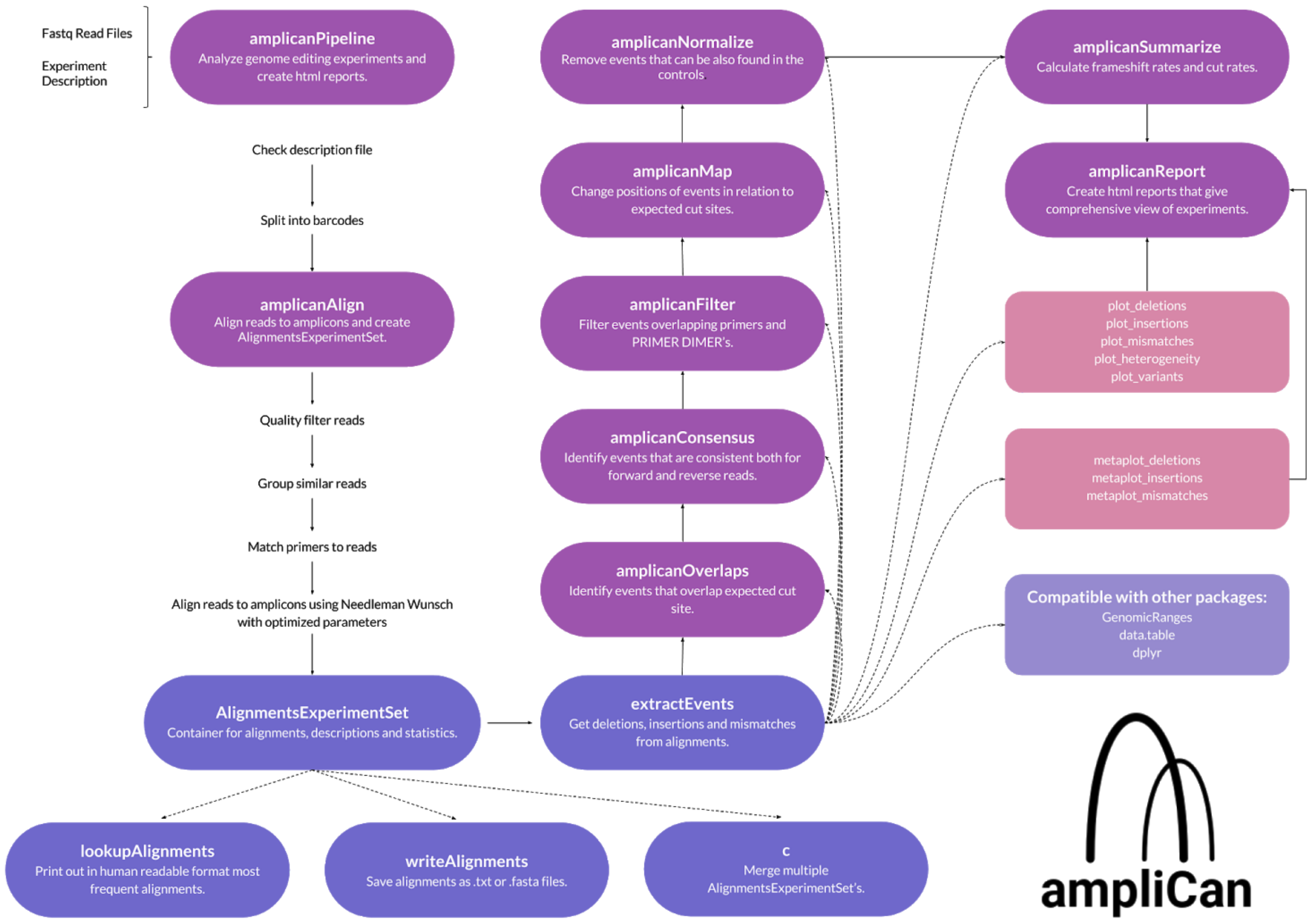

Supplemental Fig S25. Overview of the ampliCan pipeline.

Supplemental Tables

One edited read with variable number of total reads.		Estimated editing efficiency.		
Number of reads	True editing efficiency [%]	Edited [%] with normalization threshold 1% (default)	Edited [%] with Normalization threshold 0%	Control (noise, not edited) [%]
1	100	100	100	0
10	10	10	10	0
100	1	1.001001001	1.001001001	0
1000	0.1	0.1102093979	0.1001903617	0.0100190362
10000	0.01	0.0260724815	0.0100278775	0.016044604
100000	0.001	0.0187490099	0.0010026209	0.0177463891

Supplemental Table S1. Table displaying precision of the ampliCan when detecting editing events with variable normalization threshold. ampliCan can be used to identify extremely low frequency (0.001% and potentially lower) editing, but requires use of no normalization threshold (0%, below Illumina sequencing noise and alignment imperfections). Default normalization is set to 1% (above standard Illumina noise) and is recommended for standard applications as this setting negates the chance of removal of real editing events due to random sequencing and alignment noise and underestimating true editing efficiency at cost of small errors in precision for extremely deep sequencing.

Type	Source	Analysis	Figures
Real Dataset 1, Zebrafish 263 loci with controls	Gagnon et al. 2014 and newly generated	Comparison of discrepancies between tools.	Fig 1. and Supplemental Figs S1, S3, S4, S9, S13, S15, S18, S21, S22, S23, S24.
Real Dataset 2, Human, ~1,400 genomic loci	Chari et al. 2015	Example of large insertions in real data.	Supplemental Fig S10.
Synthetic Dataset 2, 20 loci x 4 efficiency rates x 3 off-target rates = 240 experiments	Lindsay et al. 2016	Reproducing previous benchmark.	Supplemental Fig S7.
Synthetic Dataset 3, As in Lindsay et al. 2016 x 3 mismatch rates = 720 experiments	Simulated based on Lindsay et al. 2016 strategy with variable parameters.	More comprehensive analysis of contaminant reads.	Fig 2. and Supplemental Figs S8, S19.
Synthetic Dataset 4, 20 loci x 4 efficiency rates x 4 types of indels = 320 experiments		Analysis of indel size influence on the efficiency estimates.	Fig 2. And Supplemental Fig S11.
Synthetic Dataset 5, 20 loci x 3 NHEJ rates x 3 HDR rates x 3 donor types = 540 experiments		Homology Directed Repair	Supplemental Fig S12.
Synthetic Dataset 1, 1 loci x 6 efficiency rates x 3 normalization thresholds = 18 experiments		Precision of ampliCan	Supplemental Tab S8.

Supplemental Table S2. Table displays all datasets used and their origin.

Tool	Aligner	Notes
ampliCan	Needleman-Wunsch from Biostrings (Pages et al.) as local alignment	
CrispRVariant (Lindsay et al. 2016)	BWA-MEM(Li 2013) as global alignment	Filters primer dimers by restricting start/end of indel, but may result in missing larger deletions. Larger insertions can be missed through mapping.
ampliconDIVider (Varshney et al. 2015) (ampliconDIV_minimal.sh)	BWA-MEM as global alignment	Sometimes returns estimates above 100%, these values were filtered. We run only the variant counting step of the pipeline. The full pipeline requires the commercial novoAlign.
CRISPResso & CRISPRessoPooled (Pinello et al. 2016)	BWA-MEM + FLASH (in Pooled) as local alignment	Poor performance due to unknown issues. It was installed and run in the same fashion as in Lindsay et al. 2016 analysis. CRISPRessoPooled uses local-global alignment strategy.

Supplemental Table S3. Table of compared tools, with align strategy.

```
ID: ID_1 read_id: 1 Count: 3
AAGCTGACGGCTAAATGAAAAATGTCAAACGTCTGTTCCAG------------------------------------------------------------------AAAAAAAAAAAAAAAAAAAATTCCCACACCAATGGGAAAGGAGTCCTGCCAGATGACCATCC
CAACTGTGTTGCAGCAGCCAGATCCAGGTGTGTTTGCGCTTGTGTAATT
AAGCTGACGGCTAAATGAAAAATGTCAAACATCTGTTCCAGGTGCTGCGTATGCCAGGGCAGAGGAGGTGGTCAGGGAACTGGTGGAGGTCACTGGGATACCCTTTC--------------------TTCCCACACCAATGGGAAAGGAGTCCTGCCAGATGACCATCC
CAACTGTGTTGCTGCAGCCAGATCCAGGTGTGTTTGCGCTTGTGTAATT

AAGCTGACGGCTAAATGAAAAATGTCAAACGTCTGTTCCAG------------------------------------------------------------------AAAAAAAAAAAAAAAAAAAATTCCCACACCAATGGGAAAGGAGTCCTGCCAGATGACCATCC
CAACTGTGTTGCAGCAGCCAGATCCAGGTGTGTTTGCGCTTGTGTAATT
AAGCTGACGGCTAAATGAAAAATGTCAAACATCTGTTCCAGGTGCTGCGTATGCCAGGGCAGAGGAGGTGGTCAGGGAACTGGTGGAGGTCACTGGGATACCCTTTC--------------------TTCCCACACCAATGGGAAAGGAGTCCTGCCAGATGACCATCC
CAACTGTGTTGCTGCAGCCAGATCCAGGTGTGTTTGCGCTTGTGTAATT

ID: ID_1 read_id: 2 Count: 2
AAGCTGACGGCTAAATGAAAAATGTCAAACGTCTGTT--------------------------------------------------------------------------AAAAAAAAAAAAAAAAAAAACACACCAATGGGAAAGGAGTCCTGCCAGATGACCATCC
CAACTGTGTTGCAGCAGCCAGATCCAGGTGTGTTTGCGCTTGTGTAATT
AAGCTGACGGCTAAATGAAAAATGTCAAACATCTGTTCCAGGTGCTGCGTATGCCAGGGCAGAGGAGGTGGTCAGGGAACTGGTGGAGGTCACTGGGATACCCTTTCTTCC--------------------CACACCAATGGGAAAGGAGTCCTGCCAGATGACCATCC
CAACTGTGTTGCTGCAGCCAGATCCAGGTGTGTTTGCGCTTGTGTAATT

AAGCTGACGGCTAAATGAAAAATGTCAAACGTCTGTT--------------------------------------------------------------------------AAAAAAAAAAAAAAAAAAAACACACCAATGGGAAAGGAGTCCTGCCAGATGACCATCC
CAACTGTGTTGCAGCAGCCAGATCCAGGTGTGTTTGCGCTTGTGTAATT
AAGCTGACGGCTAAATGAAAAATGTCAAACATCTGTTCCAGGTGCTGCGTATGCCAGGGCAGAGGAGGTGGTCAGGGAACTGGTGGAGGTCACTGGGATACCCTTTCTTCC--------------------CACACCAATGGGAAAGGAGTCCTGCCAGATGACCATCC
CAACTGTGTTGCTGCAGCCAGATCCAGGTGTGTTTGCGCTTGTGTAATT
```

Supplemental Table S4. Example of human readable output. Aligned reads are assigned to the experiment (ID, read_id) and sorted based on count (Count). For each pair alignment is presented with top part representing forward read aligned to amplicon and bottom presenting reverse read aligned to amplicon.

seqnames	start	end	width	strand	originally	replacement	type	read_id	score	counts
ID_1	108	127	20	+		AAAAAAAAAAAAAAAAAAAA	insertion	1	597	3
ID_1	112	131	20	+		AAAAAAAAAAAAAAAAAAAA	insertion	2	557	2
ID_1	42	107	66	+			deletion	1	597	3
ID_1	38	111	74	+			deletion	2	557	2
ID_1	24	173	150	+			deletion	3	193	1
ID_1	34	117	84	+			deletion	4	532	1
ID_1	31	31	1	+	A	G	mismatch	1	597	3
ID_1	163	163	1	+	T	A	mismatch	1	597	3
ID_1	31	31	1	+	A	G	mismatch	2	557	2
ID_1	163	163	1	+	T	A	mismatch	2	557	2
ID_1	23	23	1	+	T	G	mismatch	3	193	1
ID_1	176	176	1	+	A	T	mismatch	3	193	1
ID_1	177	177	1	+	G	C	mismatch	3	193	1
ID_1	31	31	1	+	A	G	mismatch	4	532	1
ID_1	171	171	1	+	G	A	mismatch	4	532	1
ID_1	108	127	20	-		AAAAAAAAAAAAAAAAAAAA	insertion	1	597	3
ID_1	112	131	20	-		AAAAAAAAAAAAAAAAAAAA	insertion	2	557	2
ID_1	42	107	66	-			deletion	1	597	3
ID_1	38	111	74	-			deletion	2	557	2
ID_1	24	173	150	-			deletion	3	193	1
ID_1	34	117	84	-			deletion	4	532	1
ID_1	31	31	1	-	A	G	mismatch	1	597	3
ID_1	163	163	1	-	T	A	mismatch	1	597	3
ID_1	31	31	1	-	A	G	mismatch	2	557	2
ID_1	163	163	1	-	T	A	mismatch	2	557	2
ID_1	23	23	1	-	T	G	mismatch	3	193	1
ID_1	176	176	1	-	A	T	mismatch	3	193	1
ID_1	177	177	1	-	G	C	mismatch	3	193	1
ID_1	31	31	1	-	A	G	mismatch	4	532	1
ID_1	171	171	1	-	G	A	mismatch	4	532	1
ID_2	115	127	13	-			deletion	1	845	3
ID_2	106	114	9	-			deletion	2	865	2
ID_2	101	105	5	-			deletion	3	885	1
ID_2	115	127	13	+			deletion	1	845	3
ID_2	106	114	9	+			deletion	2	865	2
ID_2	101	105	5	+			deletion	3	885	1
ID_3	171	171	1	+		T	insertion	1	819	5
ID_3	74	89	16	+		CCCCCCCCCCCCCCCC	insertion	3	860	1
ID_3	66	83	18	+			deletion	1	819	5

Supplemental Table S5. Example GenomicRanges table output with additional meta-columns. This representation of alignments allows for efficient manipulation and processing of the data.

ID	Barcode	Forward_Reads	Reverse_Reads	Group	Control	guideRNA	Forward_Primer	Reverse_Primer	Direction	Amplicon
ID_1	barcode_1	R1_001.fastq	R2_001.fastq	Betty	0	AGGTGGTCAGGGAACTGG	AAGCTGACGGCTAAATGA	AATTACACAAGCGCAAACACAC	0	...cagaggAGGTGGTCAGGGAACTGGtgga...
ID_2	barcode_1	R1_001.fastq	R2_001.fastq	Tom	0	TGACCCTCTGCCAACACAAGGGG	TGACCAAACCTTCTTAAGGTGC	CTCTGCTGCAAAATGCAAGG	1	...agttgtgCCCCTTGTGTTGGCAGAGGGTCAg...
ID_3	barcode_2	R1_002.fastq	R2_002.fastq	Tom	0	AGGTGGTCAGGGAACTGG	AAGCTGACGGCTAAATGA	AATTACACAAGCGCAAACACAC	0	...ggAGGTGGTCAGGGAACTGGtgga...
ID_4	barcode_2	R1_002.fastq	R2_002.fastq	Betty	0	GTCCCTGCAACATTAAAGGCCGG	GCTGGCAACATTCCTACCAGT	GAGCGCTGAGGCAGGATTAT	0	...ccaGTCCCTGCAACATTAAAGGCCGGaag...

Supplemental Table S6. Example ampliCan config file. ampliCan requires this file as minimal input, together with relevant fastq files. More precise, up to date description of the file can be found in the ampliCan vignettes.

References

Chari R, Mali P, Moosburner M, Church GM. 2015. Unraveling CRISPR-Cas9 genome engineering parameters via a library-on-library approach. *Nat Methods* **12**: 823–826.

Gagnon JA, Valen E, Thyme SB, Huang P, Akhmetova L, Ahkmetova L, Pauli A, Montague TG, Zimmerman S, Richter C, et al. 2014. Efficient mutagenesis by Cas9 protein-mediated oligonucleotide insertion and large-scale assessment of single-guide RNAs. *PLoS One* **9**: e98186.

Huang W, Li L, Myers JR, Marth GT. 2012. ART: a next-generation sequencing read simulator. *Bioinformatics* **28**: 593–594.

Li H. 2013. Aligning sequence reads, clone sequences and assembly contigs with BWA-MEM. *arXiv [q-bioGN]*. http://arxiv.org/abs/1303.3997.

Lindsay H, Burger A, Biyong B, Felker A, Hess C, Zaugg J, Chiavacci E, Anders C, Jinek M, Mosimann C, et al. 2016. CrispRVariants charts the mutation spectrum of genome engineering experiments. *Nat Biotechnol* **34**: 701–702.

Morgan M, Anders S, Lawrence M, Aboyoun P, Pagès H, Gentleman R. 2009. ShortRead: a bioconductor package for input, quality assessment and exploration of high-throughput sequence data. *Bioinformatics* **25**: 2607–2608.

Pages H, Gentleman R, Aboyoun P, DebRoy S. Biostrings: String objects representing biological sequences, and matching algorithms, 2008. *R package version* **2**: 160.

Pinello L, Canver MC, Hoban MD, Orkin SH, Kohn DB, Bauer DE, Yuan G-C. 2016. Analyzing CRISPR genome-editing experiments with CRISPResso. *Nat Biotechnol* **34**: 695–697.

Ross MG, Russ C, Costello M, Hollinger A, Lennon NJ, Hegarty R, Nusbaum C, Jaffe DB. 2013. Characterizing and measuring bias in sequence data. *Genome Biol* **14**: R51.

Shah AN, Davey CF, Whitebirch AC, Miller AC, Moens CB. 2016. Rapid Reverse Genetic

Screening Using CRISPR in Zebrafish. *Zebrafish* **13**: 152–153.

Varshney GK, Pei W, LaFave MC, Idol J, Xu L, Gallardo V, Carrington B, Bishop K, Jones M, Li M, et al. 2015. High-throughput gene targeting and phenotyping in zebrafish using CRISPR/Cas9. *Genome Res* **25**: 1030–1042.

Wickham H. 2016. *ggplot2: Elegant Graphics for Data Analysis*. Springer.

Minimizing Index Hopping. https://www.illumina.com/science/education/minimizing-index-hopping.html (Accessed January 22, 2019).
